# The microRNA (miRNA) signatures in different phases of the natural history of chronic hepatitis B virus infection

**DOI:** 10.1038/s41598-026-53116-z

**Published:** 2026-05-29

**Authors:** Qiong Wang, Fahong Li, Yurong Yao, Jinyu Wang, Jun Wang, Yong Lin, Ting Wang, Jiming Zhang, Mengji Lu

**Affiliations:** 1 Institute of Virology, University Hospital of Essen, University of Duisburg-Essen, Hufelandstrasse 55, 45122 Essen, Germany; 2 Department of Infectious Diseases, Huashan Hospital, Fudan University, 12 Middle Wulumuqi Road, Shanghai, 200040 China; 3 Department of Infectious Diseases, Jing’an District Center Hospital of Shanghai, Fudan University, Shanghai, China; 4 School of Public Health (Shenzhen), Shenzhen Campus of Sun Yat-sen University, Shenzhen, China; 5 Key Laboratory of Molecular Biology of Infectious Diseases (Chinese Ministry of Education), Chongqing Medical University, Chongqing, China

**Keywords:** MicroRNA, Hepatitis B virus, Liver, Transcriptome analysis, Immune response, Computational biology and bioinformatics, Gastroenterology, Immunology, Molecular biology

## Abstract

Chronic hepatitis B virus (HBV) infection (CHB) progresses through distinct clinical phases characterized by dynamic immune responses and changes in intrahepatic gene expression. While hepatic transcriptomes have been studied, hepatic microRNA (miRNA) profiles across the natural history of CHB remain poorly defined. In this study, we aimed to characterize hepatic miRNA expression and its association with liver cell composition across CHB phases. Liver biopsies from 43 treatment-naïve CHB patients across four clinical phases, immune tolerance (IT), immune active (IA), inactive carrier (IC), and HBeAg-negative hepatitis (ENH), were analyzed using miRNA microarrays and mRNA sequencing. Hepatic cell composition was inferred using CIBERSORT and expression of 92 cell-type marker genes. Differentially expressed miRNAs were identified by Mann–Whitney U test, and clustering and correlation analyses were performed to assess associations with immune and liver cell markers. Consistent with previous studies, immune cell infiltration varied by phase, peaking in IA and ENH phases. Sixty-five miRNAs were significantly differentially expressed (|log₂FC| > 0.585), including immune-associated miRNAs such as miR-155-5p, miR-150-5p, and miR-142-5p. Twenty-two miRNAs elevated in IA phase were associated with macrophage and T cell markers. Hepatocyte-enriched miRNAs (e.g., miR-122-5p, miR-192-5p) remained highly expressed across all phases. Clustering analysis identified five miRNA expression patterns, with immune-associated clusters upregulated in IA and ENH phases. Correlation analysis confirmed strong associations between immune-phase miRNAs and αβ T cell markers. Overall, these findings indicate that hepatic miRNA profiles are largely stable across CHB phases, with immune cell infiltration associated with selective miRNA changes, particularly in IA and ENH phases.

**Supplementary Information:**

The online version contains supplementary material available at 10.1038/s41598-026-53116-z.

## Introduction

MicroRNAs (miRNAs) are ~ 22-nucleotide-long non-coding RNAs that regulate gene expression by binding to the 3′ or 5′ untranslated regions (UTRs) of target mRNAs, leading to mRNA degradation or translational repression^1–3^. MiRNAs act as key post-transcriptional regulators, influencing diverse biological processes such as cell proliferation, differentiation, apoptosis, and other cellular functions^4,5^. Each cell type may show its specific miRNA expression profile^6^. Previously, we characterized the miRNA expression profiles of four major human hepatic cell types—primary human hepatocytes (PHHs), Kupffer cells (KCs), hepatic stellate cells (HSCs), and liver sinusoidal endothelial cells (LSECs)^7^. Due to their broad regulatory functions, miRNAs are considered potential therapeutics for diverse diseases^8^.

A large number of studies using cell culture systems have demonstrated that various host miRNAs play regulatory roles in hepatitis B virus (HBV) replication^9–12^. Rather than directly targeting HBV RNA, most miRNAs modulate host cellular mRNAs involved in pathways such as signal transduction, cell differentiation, growth, and autophagy, thereby indirectly influencing HBV gene expression and replication. Examples include miR-1, members of the miR-99 family, miR-125b, and miR-192-3p^7,10–13^. In hepatoma cell line–based systems, certain miRNAs have been shown to enhance hepatocyte differentiation and upregulate hepatocyte-related gene expression, which in turn facilitates HBV replication^14^. MiRNA expression in liver samples from patients with chronic HBV infection and liver cirrhosis was examined in early studies^15,16^. Distinct differentially expressed miRNAs were found during the different clinical phases and in fibrosis. However, the relationship between mRNA and miRNA expression in the liver is not well understood.

Hepatitis B virus (HBV) infection can cause both acute and chronic hepatitis, potentially leading to liver fibrosis, cirrhosis, and hepatocellular carcinoma (HCC)^17^. According to the American Association for the Study of Liver Diseases (AASLD) practice guidelines^18^, the natural history of chronic HBV infection is divided into 4 phases: immune tolerance (IT) phase, immune active (IA) phase, inactive carrier (IC) phase, and hepatitis B e antigen (HBeAg) negative hepatitis (ENH) phase. Previous transcriptomic analyses have characterized mRNA expression profiles in liver tissues from chronic hepatitis B (CHB) patients, revealing significantly elevated levels of large numbers of immune-related genes, including T-cell and macrophage markers, cytokines, and interferon-stimulated genes (ISGs), in the IA and ENH phases compared to the IT and IC phases^19–21^. Up to 1988 genes were found to be differentially expressed when comparing the transcriptomes of different clinical phases of chronic HBV infection^19^. Our early liver transcriptome analysis identified 109 differentially expressed genes between the IT and IC phases^22^, of which some were examined further and confirmed to contribute to HBV control by cellular signaling and degradation processes^23–25^.

In the present study, we aimed to characterize the intrahepatic miRNA landscape across different phases of chronic HBV infection, and to determine the factors associated with miRNA expression changes. Our findings reveal that intrahepatic miRNA profiles are largely stable throughout the natural history of CHB, in contrast to significant changes in mRNA expression profiles. Only limited, phase-specific alterations in miRNA expression were observed, primarily related to immune cell infiltration and immune activation in the IA and ENH phases. The changes of miRNA expression showed no correlation with serum HBV DNA and antigen loads. Particularly, viral loads in peripheral blood of patients had minimal impact on hepatic miRNA expression, as evidenced by the similarity of profiles between the IT and IC phases.

## Materials and methods

### Patient samples, cluster analysis, and sample exclusion

Patients with chronic HBV infection were recruited from Huashan Hospital, affiliated with Fudan University, Shanghai, China. All enrolled patients were diagnosed with chronic HBV infection, were treatment-naïve, and had not received immunosuppressive therapy within six months prior to liver biopsy. Patients with co-infections (HIV, hepatitis D, or hepatitis C), autoimmune liver diseases, genetic or metabolic liver disorders, or any other primary liver diseases were excluded. The study was approved by the Ethics Committee of Huashan Hospital, Fudan University, and conducted in accordance with the 1975 Declaration of Helsinki. For patients with CHB, liver biopsies were undertaken for determining the grade of liver necroinflammation and fibrosis, as well as excluding concomitant liver diseases. Written informed consent was obtained from all participants for the research use of collected clinical specimens, and the study was approved by the institutional ethics committee (approval No. KY2021-919).

**Fig. 1:**
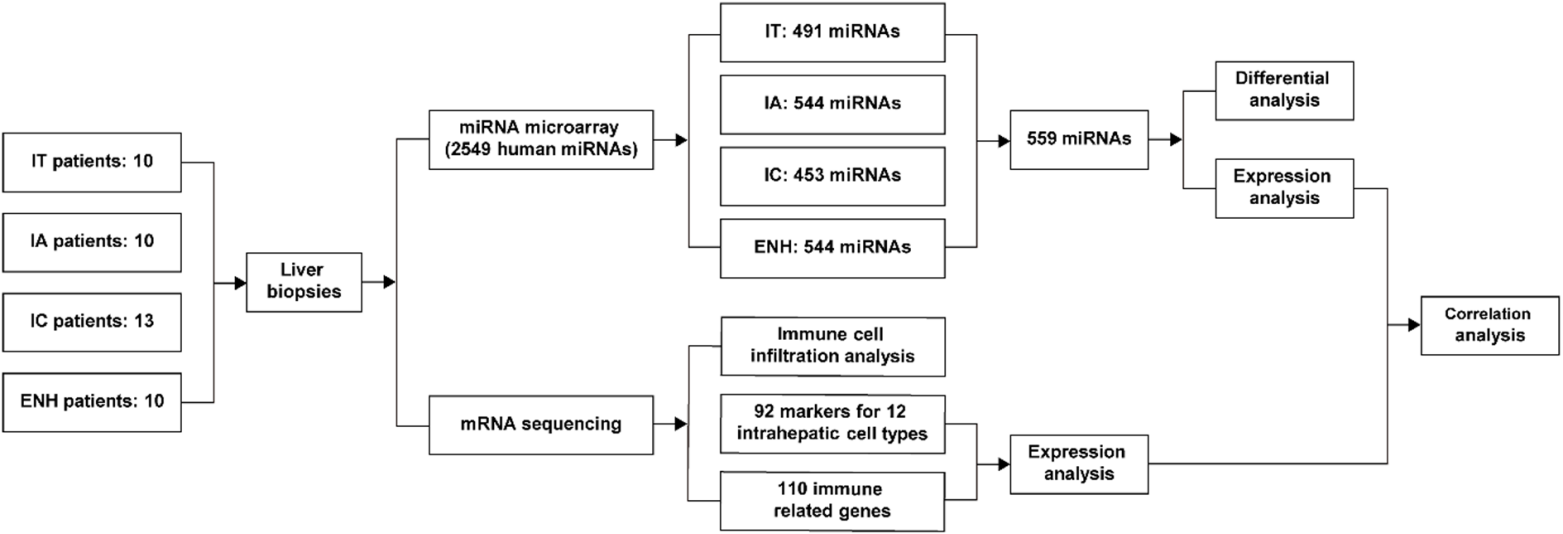
Workflow of study design and data analysis. Liver biopsy samples from 43 patients with chronic HBV infection—classified into IT (*n* = 10), IA (*n* = 10), IC (*n* = 13), and ENH (*n* = 10) phases—underwent miRNA microarray profiling (covering 2,549 human miRNAs) and bulk RNA sequencing analysis. A total of 559 miRNAs (491 in IT, 544 in IA, 453 in IC, and 544 in ENH) were included in differential expression analyses. CIBERSORT was applied to RNA-seq data to estimate immune cell infiltration. Expression of 92 intrahepatic cell-type marker genes and 110 immune-related genes was visualized using heatmaps across clinical phases. Correlation analyses were performed to evaluate associations between miRNA levels and expression of the 92 intrahepatic marker genes. Abbreviations are as defined in the text.

Based on the AASLD practice guidelines, the 43 CHB patients were classified into four phases: immune tolerance (IT, *n* = 10), immune active (IA, *n* = 10), inactive carrier (IC, *n* = 13), and HBeAg-negative hepatitis (ENH, *n* = 10) (Table S1). Liver biopsy was performed as described previously^22^, and histological findings were consistent with serological and virological characteristics. According to the Scheuer criteria, patients in the IT and IC phases had low G and S scores (0–1), while over 50% of IA patients exhibited high scores (2–4). Patient characteristics are summarized in Supplementary Table S1. The 43 collected liver samples were subjected to RNA sequencing analysis and miRNA microarray (Fig. 1).

We performed clustering dendrogram and principal component analysis (PCA) on all 43 samples (Fig. S1). Two samples (IA_10 and ENH_10) showed divergent patterns and were excluded, resulting in 41 samples: IT (*n* = 10), IA (*n* = 9), IC (*n* = 13), and ENH (*n* = 9) (Fig. S1a–c). Further analysis revealed that IA_10 and ENH_10 exhibited abnormal cell compositions with elevated expression of endothelial gene markers (Fig. S1d).

### RNA sequencing and miRNA microarray

Liver biopsy samples were collected and sent to Shanghai Biotechnology Co., Ltd. for RNA sequencing analysis. Total RNA was extracted from liver samples using the mir VanaTM miRNA Isolation Kit (Ambion, USA). RNA libraries were prepared using the VAHTS Total RNA-Seq Library Prep Kit, according to the manufacturer’s protocol, and sequenced on an Illumina platform. The sequencing data have been submitted to a public repository (accession number: GSE298398).

The expression of miRNAs in liver biopsy samples was determined using miRNA microarray^26,27^. Total RNA was extracted and subjected to electrophoresis, with RNA integrity assessed using the RNA Integrity Number (RIN) for quality control. The RNA samples were analyzed using the Agilent Human miRNA (8 × 60 K) array. The arrays were designed based on miRBase v21.0 and included 2,549 annotated human miRNAs. Raw signal intensities from the miRNA microarray were processed using the rmaMicroRna function, which includes background correction and quantile normalization. Normalized expression values were then log₂-transformed prior to downstream analyses. Present (P) and Absent (A) detection flags were assigned according to the default Agilent Feature Extraction detection algorithm, based on detection p-values relative to background signal. Batch effects were not formally assessed, and no batch correction was applied in the miRNA microarray dataset. Fold change (FC) and log₂FC values were calculated from group-wise mean expression levels based on the normalized miRNA expression data. A total of 559 miRNAs were detected across the CHB liver samples analyzed, including 491 in IT, 544 in IA, 453 in IC, and 544 in ENH phase samples.

### Databases and bioinformatics analysis

A panel of 92 liver cell-type marker genes, covering 12 distinct hepatic cell types, including hepatocytes, cholangiocytes, T cells (αβ T cells, γδ T cells 1, γδ T cells 2, NK-like cells), B cells (B cells, plasma cells), macrophages (inflammatory and non-inflammatory), and endothelial cells, was compiled as described in our recent publication^19^. These markers were selected based on published single-cell RNA sequencing datasets of hepatic cells^28,29^. An additional set of 110 markers associated with immune regulation, including immune regulators, chemokine receptors, chemokines, complement components, inflammasomes, and cytokines, was also included for bioinformatic analysis^19^.

Expression and clustered heatmaps were generated for the 559 miRNAs and the selected marker genes. Log₂-normalized values were displayed without additional scaling for baseline expression visualization. For variability analysis, standard deviation (SD) values calculated from log₂-normalized signals were displayed without additional transformation. For hierarchical clustering heatmaps, expression values were standardized by row-wise Z-score transformation prior to clustering using Euclidean distance. For mRNA sequencing data, fragments per kilobase of transcript per million mapped reads (FPKM)-normalized expression values were used and Z-score transformed for clustering heatmap visualization. The full list of markers is provided in Supplementary Tables S2–S3.

Correlation analyses were conducted to assess associations between miRNA and marker gene expression in liver biopsy samples. Normality of each variable was assessed using the Shapiro–Wilk test. Pearson correlation coefficients were calculated when both variables passed the normality test (*p* > 0.05), and Spearman rank correlation coefficients were used otherwise. For the global correlation heatmap (559 miRNAs × 92 marker genes), raw correlation coefficients (r values) were displayed. P-values from all miRNA–marker pairs were adjusted using the Benjamini–Hochberg procedure to control the false discovery rate. For scatter plot visualization, regression lines were shown with 95% confidence intervals. Phase-specific confidence intervals for correlation coefficients were estimated at the 95% level using Fisher’s Z-transformation.

We used FPKM-normalized bulk RNA-seq data as input for the cell-type identification by estimating relative subsets of RNA transcripts (CIBERSORT) algorithm on the CIBERSORTx web platform (https://cibersortx.stanford.edu/)^30^, to estimate immune cell infiltration in liver tissue samples. The LM22 signature matrix, encompassing 22 human immune cell types, was applied in relative mode (absolute mode disabled). Differential expression analysis was conducted on the OmicStudio web platform (https://www.omicstudio.cn/) using the non-parametric Mann–Whitney U test for group comparisons. Raw p-values were adjusted using the Benjamini–Hochberg procedure to control the false discovery rate (FDR), with adjusted *p* < 0.05 considered statistically significant. Data processing, statistical analyses and visualization were performed using R and Python.

## Results

### General description of intrahepatic miRNA expression across clinical phases in CHB patients

**Fig. 2:**
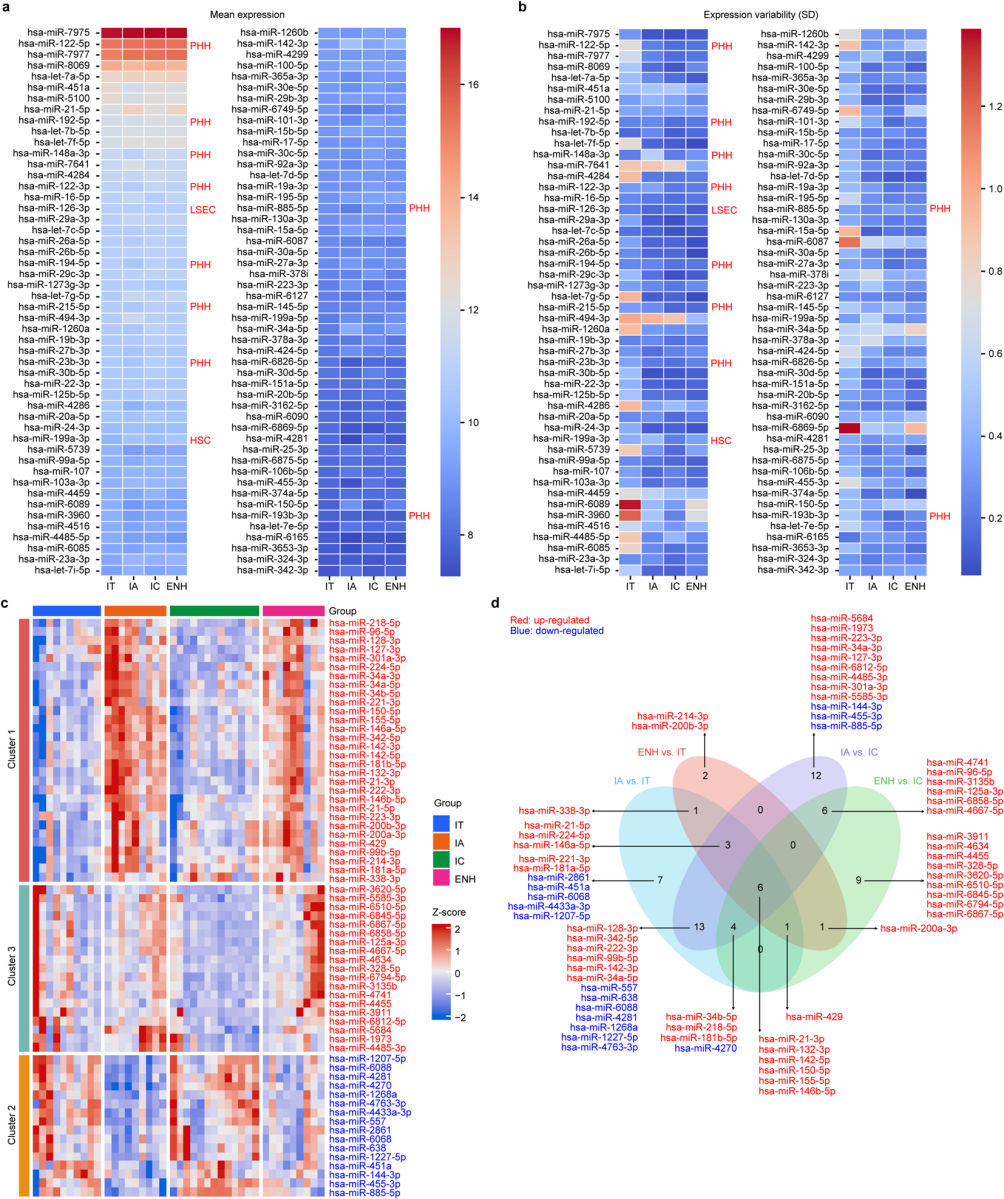
miRNA expression and variability in liver biopsies from CHB patients. (**a**) Heatmap of the top 100 most highly expressed miRNAs across all patient samples. Heatmap showing mean log₂-normalized signal in IT, IA, IC and ENH groups; right-side labels denote representative hepatic cell type–enriched miRNAs; colors indicate expression level (red = high; blue = low). (**b**) Heatmap showing interindividual variability (standard deviation of log₂-normalized signal values) for each miRNA within clinical phases; red = high variance; blue = low variance. (**c**) Hierarchical clustering heatmap of the 65 top differentially expressed miRNAs identified by Mann–Whitney U test (adjusted *p* < 0.05 (FDR), |log₂FC| > 0.585); font color indicates regulation direction (red = upregulated in IA or ENH versus IT/IC; blue = downregulated). Expression values Z-score normalized (red = high; blue = low). (**d**) Venn diagram depicting overlap of these 65 top differentially expressed miRNAs in ENH vs. IT (red), IA vs. IT (cyan), IA vs. IC (purple) and ENH vs. IC (green); arrows mark unique and shared miRNAs, font color as in (**c**). Abbreviations are as defined in the text.

Consistent with our previous analysis, the major changes in hepatic transcriptomes of CHB patients were associated with immune cell infiltration and inflammation during the IA/ENH phases (Fig. S2). In the present study, we additionally quantified hepatic miRNA expression. In total, 559 miRNAs were detected across the CHB liver samples analyzed, with 491, 544, 453, and 544 miRNAs identified in the IT, IA, IC, and ENH phases, respectively (Table S4). Normalized signal intensities ranged from approximately 2.5 to 17. Figure 2a displays the 100 most abundantly expressed hepatic miRNAs, including miR-7975, miR-122-5p, miR-7977, miR-8069, let-7a-5p, miR-451a, miR-5100, miR-21-5p, let-7f-5p, and miR-192-5p. Hepatic cell type–enriched miRNAs were annotated based on our previous study^7^ and used here as a reference framework for interpreting the bulk liver tissue data (Fig. 2a).

To assess expression stability, we calculated the standard deviation of normalized miRNA signal values within each clinical group. Overall, most miRNAs exhibited stable expression across liver samples (Fig. 2b). However, the IA and ENH phases exhibited greater variability in miRNA expression, particularly among PHH-enriched miRNAs such as miR-122-5p, miR-192-5p, miR-194-5p, miR-215-5p, and miR-122-3p (Fig. 2b). Some miRNAs, including miR-6089 and miR-6869-5p, showed high expression variability in IT phase samples; however, their hepatic functions remain undefined.

### Significantly up- and down-regulated miRNAs in IA and ENH phases compared with IT or IC phases

Next, we conducted differential expression analysis of the 559 miRNAs across the CHB clinical phases (Table 1). Group comparisons were performed using the Mann–Whitney U test, and FC and log₂FC values were calculated from group-wise mean expression levels based on the normalized miRNA expression data. Significant differences in miRNA expression were observed in the following group comparisons: ENH vs. IT (37 miRNAs), IA vs. ENH (1 miRNA), IA vs. IT (74 miRNAs), IA vs. IC (224 miRNAs), and ENH vs. IC (148 miRNAs). A fold change threshold of |log₂FC| > 0.585 (corresponding to a 1.5-fold change, which is not a strict biological threshold) was used to formally define a subset of top differentially expressed miRNAs for downstream interpretation. Thereby, we identified 14, 35, 44, and 27 top differentially expressed miRNAs in ENH vs. IT, IA vs. IT, IA vs. IC, and ENH vs. IC comparisons, respectively, resulting in 65 unique miRNAs (Fig. 2c; Table 2). The largest number of differentially expressed miRNAs was observed in the IA vs. IC comparison. No miRNAs were differentially expressed between the IT and IC groups at a p-value cutoff of 0.05.

**Table 1:** Differential-expression analysis of 559 miRNAs across four phases of chronic HBV infection.

Comparison	Number of differentially expressed miRNAs, adj. *p* < 0.05 (up/down)	Number of top differentially expressed miRNAs, adj. *p* < 0.05, |log₂FC| > log₂1.5 (up/down)
IT vs. IC	0	0
ENH vs. IT	37 (37/0)	14 (14/0)
IA vs. ENH	1 (0/1)	0
IA vs. IT	74 (51/23)	35 (22/13)
IA vs. IC	224 (182/42)	44 (33/11)
ENH vs. IC	148 (138/10)	27 (26/1)
Total	273 (224/49)	65 (49/16)

Differential expression was assessed by Mann–Whitney U test with Benjamini–Hochberg FDR correction (adjusted *p* < 0.05). Top differentially expressed miRNAs were defined by adjusted *p* < 0.05 and |log₂FC| > log₂1.5 (≈ 0.585). “Up/down” denotes the number of miRNAs significantly up-/down-regulated in the first group relative to the second. Total counts represent the unique set of all differentially expressed miRNAs across comparisons.

Abbreviations: HBV, hepatitis B virus; IT, immune tolerance; IA, immune active; IC, inactive carrier state; ENH, HBeAg-negative hepatitis; FDR, false discovery rate; FC, fold change.

**Table 2:** Top 65 differentially expressed miRNAs in CHB phase comparisons.

Cluster	miRNA	IA vs. IT		IA vs. IC		ENH vs. IT		ENH vs. IC	
		adj. *p*	log₂FC	adj. *p*	log₂FC	adj. *p*	log₂FC	adj. *p*	log₂FC
Cluster 1	hsa-miR-218-5p	0.023	0.77	0.004	0.71			0.019	0.86
Cluster 1	hsa-miR-96-5p			0.010	0.62			0.002	0.66
Cluster 1	hsa-miR-128-3p	0.027	0.70	0.005	0.80				
Cluster 1	hsa-miR-127-3p			0.000	0.71				
Cluster 1	hsa-miR-301a-3p			0.001	0.71				
Cluster 1	hsa-miR-224-5p	0.023	1.38	0.029	1.20	0.039	1.28		
Cluster 1	hsa-miR-34a-3p			0.001	0.61				
Cluster 1	hsa-miR-34a-5p	0.008	1.30	0.005	0.87				
Cluster 1	hsa-miR-34b-5p	0.006	1.63	0.003	1.52			0.031	1.17
Cluster 1	hsa-miR-221-3p	0.019	0.73						
Cluster 1	hsa-miR-150-5p	0.002	1.11	0.000	0.99	0.039	0.80	0.009	0.68
Cluster 1	hsa-miR-155-5p	0.002	1.66	0.000	1.62	0.039	1.13	0.008	1.08
Cluster 1	hsa-miR-146a-5p	0.002	1.00	0.000	0.87	0.043	0.69		
Cluster 1	hsa-miR-342-5p	0.002	0.71	0.000	0.61				
Cluster 1	hsa-miR-142-3p	0.005	0.94	0.000	0.87				
Cluster 1	hsa-miR-142-5p	0.002	1.09	0.000	1.04	0.043	0.80	0.013	0.75
Cluster 1	hsa-miR-181b-5p	0.010	0.81	0.000	0.97			0.007	0.68
Cluster 1	hsa-miR-132-3p	0.005	0.86	0.000	0.95	0.039	0.60	0.007	0.69
Cluster 1	hsa-miR-21-3p	0.002	0.75	0.000	0.82	0.039	0.60	0.007	0.68
Cluster 1	hsa-miR-222-3p	0.006	0.76	0.000	0.81				
Cluster 1	hsa-miR-146b-5p	0.010	1.23	0.002	1.08	0.043	0.77	0.037	0.62
Cluster 1	hsa-miR-21-5p	0.002	0.89	0.001	0.71	0.039	0.65		
Cluster 1	hsa-miR-223-3p			0.010	0.66				
Cluster 1	hsa-miR-200b-3p					0.043	1.07		
Cluster 1	hsa-miR-200a-3p					0.039	1.16	0.031	0.86
Cluster 1	hsa-miR-429	0.046	1.05			0.039	1.12	0.042	0.76
Cluster 1	hsa-miR-99b-5p	0.032	0.77	0.021	0.63				
Cluster 1	hsa-miR-214-3p					0.039	0.60		
Cluster 1	hsa-miR-181a-5p	0.014	0.59						
Cluster 1	hsa-miR-338-3p	0.027	0.59			0.039	0.89		
Cluster 3	hsa-miR-3620-5p							0.002	0.87
Cluster 3	hsa-miR-5585-3p			0.042	0.59				
Cluster 3	hsa-miR-6510-5p							0.049	0.83
Cluster 3	hsa-miR-6845-5p							0.030	0.65
Cluster 3	hsa-miR-6867-5p							0.034	0.76
Cluster 3	hsa-miR-6858-5p			0.014	0.75			0.049	0.86
Cluster 3	hsa-miR-125a-3p			0.010	0.72			0.023	0.79
Cluster 3	hsa-miR-4667-5p			0.009	0.59			0.035	0.66
Cluster 3	hsa-miR-4634							0.035	1.37
Cluster 3	hsa-miR-328-5p							0.042	0.68
Cluster 3	hsa-miR-6794-5p							0.023	0.62
Cluster 3	hsa-miR-3135b			0.018	0.64			0.023	1.16
Cluster 3	hsa-miR-4741			0.023	0.62			0.035	1.16
Cluster 3	hsa-miR-4455							0.003	0.76
Cluster 3	hsa-miR-3911							0.035	0.61
Cluster 3	hsa-miR-6812-5p			0.012	0.65				
Cluster 3	hsa-miR-5684			0.001	0.60				
Cluster 3	hsa-miR-1973			0.032	0.73				
Cluster 3	hsa-miR-4485-3p			0.037	0.65				
Cluster 2	hsa-miR-1207-5p	0.002	-0.69						
Cluster 2	hsa-miR-6088	0.002	-0.82	0.000	-0.70				
Cluster 2	hsa-miR-4281	0.002	-0.75	0.000	-0.81				
Cluster 2	hsa-miR-4270	0.002	-1.59	0.000	-1.75			0.002	-0.94
Cluster 2	hsa-miR-1268a	0.002	-0.92	0.001	-0.70				
Cluster 2	hsa-miR-4763-3p	0.026	-0.67	0.013	-0.70				
Cluster 2	hsa-miR-4433a-3p	0.002	-0.71						
Cluster 2	hsa-miR-557	0.003	-0.84	0.007	-0.74				
Cluster 2	hsa-miR-2861	0.017	-0.72						
Cluster 2	hsa-miR-6068	0.026	-0.67						
Cluster 2	hsa-miR-638	0.014	-0.59	0.023	-0.72				
Cluster 2	hsa-miR-1227-5p	0.020	-0.74	0.026	-0.74				
Cluster 2	hsa-miR-451a	0.035	-0.70						
Cluster 2	hsa-miR-144-3p			0.042	-0.64				
Cluster 2	hsa-miR-455-3p			0.001	-0.76				
Cluster 2	hsa-miR-885-5p			0.001	-0.62				

Differential expression was assessed by Mann–Whitney U test with Benjamini–Hochberg FDR correction (adjusted *p* < 0.05). The 65 top miRNAs were those with adjusted *p* < 0.05 and |log₂FC| > log₂1.5 (≈ 0.585). Columns show adjusted p-values and log₂ fold changes for each comparison. Blank cells indicate miRNAs that did not simultaneously meet both criteria of adjusted *p* < 0.05 and |log₂FC| > log₂ 1.5 in that comparison and are therefore omitted. miRNA clusters (1–3) derive from hierarchical clustering of the 65 top differentially expressed miRNAs (see Fig. 2c).

Abbreviations: HBV, hepatitis B virus; IT, immune tolerance; IA, immune active; IC, inactive carrier state; ENH, HBeAg-negative hepatitis; FDR, false discovery rate; FC, fold change.

A total of 74 miRNAs were significantly differentially expressed between the IA and IT phases (*p* < 0.05), including 51 upregulated and 23 downregulated miRNAs. By comparison, 224 miRNAs were differentially expressed in IA vs. IC (*p* < 0.05), including 182 upregulated and 42 downregulated miRNAs (Table 1, Table S5). The 65 most significantly altered miRNAs in IA and ENH versus IT and IC (|log₂FC| > 0.585) are summarized in a Venn diagram (Fig. 2d; Table 2). These comparisons revealed both unique and overlapping differentially expressed miRNAs among the clinical groups.

Several miRNAs including hsa-miR-155-5p, hsa-miR-150-5p, hsa-miR-132-3p, hsa-miR-142-5p, hsa-miR-21-3p, and hsa-miR-146b-5p were consistently upregulated across multiple pairwise comparisons. Twenty-two miRNAs significantly upregulated in the IA phase relative to IT and IC are listed in Table 3. Many of these are functionally linked to macrophages and T-cell biology based on previous reports (Table S6). Certain miRNAs appeared uniquely upregulated in either the IA or ENH phase and may play roles in hepatic immune responses: these include miR-21-3p, miR-181a-2-3p, miR-181c-5p, miR-34a-3p, miR-342-5p, miR-625-5p, and miR-511-3p (Table S7).

**Table 3:** 22 significantly up-regulated miRNAs in IA phase compared to IT and IC phases.

miRNA	IA vs. IC		IA vs. IT		Hepatic cell–type enrichment	Reported immune cell associations
	adj. *p*	log₂FC	adj. *p*	log₂FC		
hsa-miR-155-5p	< 0.001	1.62	< 0.005	1.66	KC	DC, macrophage, B cell, T cell, KC
hsa-miR-142-5p	< 0.001	1.04	< 0.005	1.09	KC	T cell, B cell, macrophage
hsa-miR-150-5p	< 0.001	0.99	< 0.005	1.11		T cell, DC
hsa-miR-181b-5p	< 0.001	0.97	< 0.05	0.81		T cell, B cell, macrophage, monocyte
hsa-miR-132-3p	< 0.001	0.95	< 0.01	0.86		Macrophage
hsa-miR-146a-5p	< 0.001	0.87	< 0.005	1.00		macrophage, DC
hsa-miR-142-3p	< 0.001	0.87	< 0.005	0.94		T cell, DC, macrophage
hsa-miR-21-3p	< 0.001	0.82	< 0.005	0.75		T cell, macrophage
hsa-miR-222-3p	< 0.001	0.81	< 0.01	0.76	LSEC	macrophage
hsa-miR-301a-3p	< 0.001	0.71				NK cell, macrophage, T cell
hsa-miR-127-3p	< 0.001	0.71			HSC	macrophage
hsa-miR-342-5p	< 0.001	0.61	< 0.005	0.71		macrophage
hsa-miR-34a-3p	< 0.001	0.61				T cell, DC
hsa-miR-34b-5p	< 0.005	1.52	< 0.01	1.63		B cell, macrophage
hsa-miR-146b-5p	< 0.005	1.08	< 0.05	1.23		Macrophage, DC, T cell
hsa-miR-218-5p	< 0.005	0.71	< 0.05	0.77		NK cell
hsa-miR-21-5p	< 0.005	0.71	< 0.005	0.89	KC	T cell, macrophage, DC
hsa-miR-34a-5p	< 0.01	0.87	< 0.01	1.30		T cell, macrophage
hsa-miR-128-3p	< 0.01	0.80	< 0.05	0.70		DC, T cell
hsa-miR-223-3p	< 0.01	0.66				neutrophils, macrophage, DC
hsa-miR-96-5p	< 0.01	0.62				T cell
hsa-miR-4667-5p	< 0.01	0.59				-

Differential expression was assessed by Mann–Whitney U test with Benjamini–Hochberg correction (adjusted *p* < 0.05). Only miRNAs with |log₂FC| > log₂1.5 (≈ 0.585) in IA vs. IT or IA vs. IC are shown. Blank cells denote comparisons in which a miRNA failed to meet both the adjusted *p* < 0.05 and |log₂FC| > log₂1.5 criteria. “Hepatic cell–type enrichment” indicates the hepatic cell type (PHH, KC, HSC, LSEC) in which each miRNA is most highly expressed (see Fig. 4b). “Reported immune cell associations” lists the immune cell types in which each miRNA has documented regulatory roles (see References in Supplementary Table S6).

Abbreviations: IT, immune tolerance; IA, immune active; IC, inactive carrier state; FC, fold change; PHH, primary human hepatocyte; KC, Kupffer cell; LSEC, liver sinusoidal endothelial cell; HSC, hepatic stellate cell; NK, natural killer cell; DC, dendritic cell.

Our analysis indicates that the miRNA expression profile in the liver shows only limited changes during the natural history, in contrast to significant changes of hepatic gene expression.

### Clustered heatmap analyses of global and hepatic cell type–enriched miRNAs

**Fig. 3:**
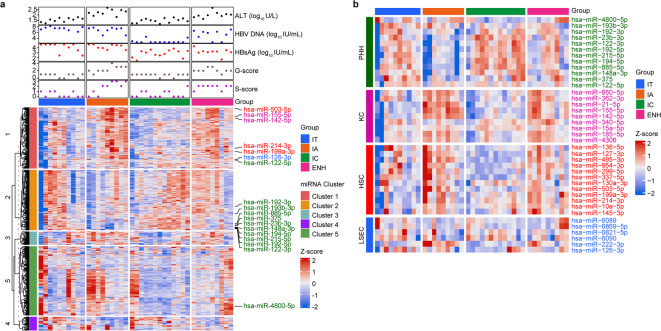
Clustered heatmaps of global and cell type–enriched miRNA expression across CHB phases. (**a**) Euclidean distance–based heatmap of 559 miRNAs. Five distinct expression clusters were identified (left color bar), with patient samples clustered within IT, IA, IC and ENH phases (top bar). Upper tracks show serum ALT (black), HBV DNA (blue) and HBsAg (red) on log₁₀ scales, as well as histologic grade (G-score) and stage (S-score) for each biopsy. Right-side labels highlight 18 representative hepatic cell type–enriched miRNAs: PHH (green), KC (red), HSC (magenta) and LSEC (blue). (**b**) Heatmap of 39 hepatic cell type–enriched miRNAs (including the 18 representative hepatic cell type–enriched miRNAs highlighted in panel a), with rows clustered by cell type (left bars) and columns clustered within each phase; miRNA labels and color scheme as in (**a**). Expression values Z-score normalized (red = high; blue = low). Abbreviations: ALT, alanine aminotransferase; G-score, histologic grade; S-score, histologic stage; others are as defined in the text.

Differential expression analysis identified a number of miRNAs that were significantly upregulated in the IA and ENH phases compared to the IT and IC phases. However, this analysis did not provide a comprehensive overview of miRNA abundance across all patient samples. To address this, Fig. 3a presents a Euclidean distance–based hierarchical clustering heatmap. In this visualization, miRNAs (rows) were clustered into five groups based on their expression profiles, while patient samples (columns) were clustered within each clinical phase (IT, IA, IC, ENH). Clinical annotations for ALT, HBV DNA, and HBsAg levels are displayed above the columns.

The clustering analysis revealed five distinct miRNA expression patterns across the CHB clinical phases. Clusters 1 and 3 consisted of miRNAs that were markedly upregulated in the IA and ENH phases, whereas Clusters 2 and 4 contained miRNAs that were downregulated in these phases.

The 65 top differentially expressed miRNAs identified above were mapped to these five clusters (Fig. 2c; Table 2). Notably, hsa-miR-155-5p, hsa-miR-150-5p, hsa-miR-132-3p, hsa-miR-142-5p, hsa-miR-21-3p, and hsa-miR-146b-5p consistently showed elevated expression in the IA and ENH phases. These miRNAs were distributed among three of the five clusters and exhibited distinct expression patterns across the four clinical phases.

To further investigate hepatic cell type–associated miRNA patterns, a clustered heatmap was generated for 39 hepatic cell type–enriched miRNAs (Fig. 3b, Supplementary Table S8). PHH-enriched miRNAs generally showed a slight decrease in expression in the IA and ENH phases compared to the IT and IC phases. An exception was miR-122, which maintained stable expression across all four CHB phases. Among KC-enriched miRNAs, miR-142-5p and miR-155-5p were significantly upregulated in the IA phase compared to the IT phase, and similarly elevated in the ENH phase relative to the IC phase. This pattern is consistent with the reported high expression of these miRNAs in immune cells. HSC-enriched miRNAs were also upregulated in the IA and ENH phases, although their overall expression levels were low, likely reflecting the relatively small proportion of HSCs in liver tissue. In contrast, LSEC-enriched miRNAs exhibited relatively stable expression across all four phases.

### The expression of selected liver cell markers and their correlations with miRNA expression

**Fig. 4:**
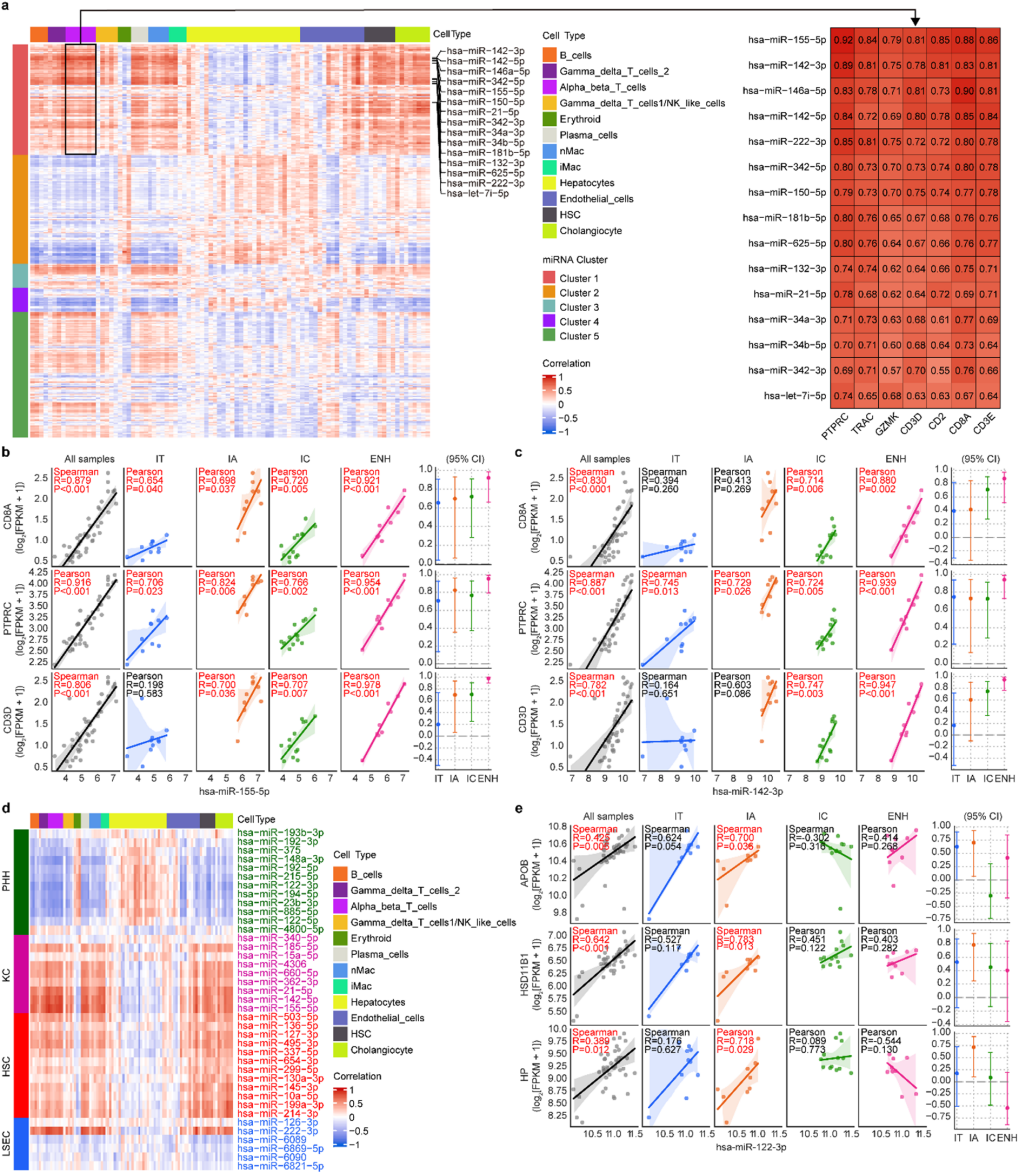
Correlation analyses between miRNA expression and intrahepatic cell-type marker genes across CHB phases. (**a**) Heatmap of correlation coefficients between 559 miRNAs (rows) and 92 marker genes of 12 liver cell types (columns) across 41 samples; miRNAs were hierarchically clustered within their original five clusters (left color bar), and marker genes were clustered within their cell-type groups (top color bar). Inset shows the 15 miRNAs most strongly correlated with αβ T-cell markers (TRAC, PTPRC, GZMK, CD3D, CD3E, CD2). (**b**) Scatter plots of hsa-miR-155-5p versus CD8A, PTPRC, and CD3D expression: leftmost panel shows correlation across all samples (*n* = 41); middle panels show correlation within each phase (IT, IA, IC, ENH) with 95% confidence intervals; rightmost panel compares phase-specific correlation coefficients (95% CIs). (**c**) Same as (**b**) for hsa-miR-142-3p versus the same markers. (**d**) Heatmap of correlation coefficients between 39 hepatic cell type–enriched miRNAs (including the 18 representative hepatic cell type–enriched miRNAs highlighted in Fig. 3a) and the 92 cell-type marker genes; miRNAs and markers were hierarchically clustered within their cell-type groups. (**e**) Scatter plots of PHH-enriched hsa-miR-122-3p versus three PHH marker genes (APOB, HSD11B1, HP), plotted as in (**b–c**). For scatter plots in panels (**b**), (**c**), and (**e**), correlation coefficients were calculated using Pearson correlation when both variables passed the Shapiro–Wilk normality test and Spearman rank correlation otherwise. For phase-specific comparisons, 95% confidence intervals were estimated using Fisher’s Z-transformation. Raw p-values are shown in scatter plots, and *p* < 0.05 is indicated in red. Abbreviations: CI, confidence interval; others are as defined in the text.

We next analyzed the correlations between hepatic expression of the 559 miRNAs and the selected liver cell-type marker genes across all patient samples. The strength of these correlations is visualized as a heatmap in Fig. 4. MiRNAs belonging to Cluster 1 and, to a lesser extent, Cluster 3 showed positive correlations with markers of immune cells, hepatic stellate cells, cholangiocytes, and, in part, endothelial cells. In contrast, miRNAs in Clusters 2 and 4 exhibited negative correlations with these same markers (Fig. 4a, left panel).

The strongest correlations were observed between a set of 15 miRNAs—including miR-155-5p, miR-142-5p, and miR-146a-5p—and markers of immune cells (Fig. 4a, right panel). Key immune markers included GZMK, CD8A, PTPRC (CD45), CD3D, CD3E, CD2, and TRAC, all of which are associated with T cell identity and function. Figure 4a presents a comprehensive correlation matrix that illustrates the relationships between these 15 miRNAs and αβ T cell markers across all clinical samples. While some miRNA–marker pairs showed strong positive associations, others demonstrated weaker or more variable correlations. Importantly, the majority of these miRNAs were also identified as differentially expressed in earlier analyses and were associated with T cell and macrophage signatures (Tables 2 and 3). Together, these findings consistently indicate that the primary changes in miRNA expression are positively associated with immune cell infiltration and activation in CHB.

Figure 4b and c further explore the correlation between hsa-miR-155-5p and hsa-miR-142-3p and three representative αβ T cell markers, presenting data across all 41 samples (left panels) and stratified by CHB clinical phase (right panels). While positive correlations were observed in all phases, the patterns varied. The highest expression levels of hsa-miR-155-5p and hsa-miR-142-3p occurred during the IA phase, consistent with increased lymphocyte infiltration. However, αβ T cell marker expression was also exceptionally elevated in this phase, resulting in reduced correlation coefficients (R) and p-values compared to phases with more moderate immune activity. Additional analysis of other miRNAs confirmed similar correlation trends with αβ T cell markers (data not shown). For instance, although several miRNAs, such as hsa-miR-34a-3p, demonstrated significant correlations with αβ T cell markers in the combined dataset, these associations were diminished when analyzed within individual clinical phases.

Following the global correlation analysis between 559 miRNAs and 92 intrahepatic cell-type markers (Fig. 4a), we focused on the 39 miRNAs previously identified in Fig. 3b as enriched in PHH, KC, HSC, and LSEC lineages. These miRNAs were correlated with the same 92 markers across all 41 liver biopsy samples (Fig. 4d), using Pearson or Spearman correlation coefficients. This cell-type–focused heatmap recapitulated and expanded upon trends from the global analysis. PHH-enriched miRNAs, including hsa-miR-122-3p, miR-192-3p, and miR-193b-3p, exhibited strong positive correlations with hepatocyte markers. KC-enriched miRNAs (e.g., hsa-miR-155-5p, hsa-miR-142-5p) were not only positively correlated with macrophage/KC markers but also showed strong associations with multiple immune cell markers. HSC-enriched miRNAs (e.g., hsa-miR-199a-3p) correlated positively with both HSC markers and immune-related genes. In contrast, LSEC-enriched miRNAs displayed generally weak or no significant correlations with any marker genes; however, hsa-miR-222-3p, classified as LSEC-enriched, showed notable positive correlations with immune cell markers, suggesting broader associations.

Phase-stratified analyses provided additional insight. The PHH-enriched hsa-miR-122-3p showed significant positive correlations with APOB, HSD11B1, and HP, but not in the IC or ENH phases (*P* > 0.05; Fig. 4e). The KC-enriched hsa-miR-155-5p was positively correlated with CD163, with no significant associations in other phases. The HSC-enriched hsa-miR-199a-3p correlated with ACTA2. Conversely, the LSEC-enriched hsa-miR-126-3p did not correlate significantly with CLEC4M in any clinical phase (*P* > 0.05; Fig. S3). For full numerical detail of these phase-stratified correlations, see Supplementary Results. Moreover, box-and-whisker plots across an external human cellular microRNAome resource (UCSC Genome Browser) covering 196 primary human cell types showed that hsa-miR-155-5p, hsa-miR-142-3p, hsa-miR-146a-5p, hsa-miR-142-5p, hsa-miR-342-5p, and hsa-miR-150-5p are markedly enriched in immune lineages (Fig. S4)^34^.

Collectively, these findings suggest that the 39 hepatic cell type–enriched miRNAs quantitatively co-vary with both hepatic cell marker patterns and broader immune dynamics in CHB liver tissue, supporting their potential utility as molecular indicators of intrahepatic cell composition and immune microenvironmental changes.

## Discussion

In this study, we examined hepatic miRNA expression in liver biopsy samples from treatment-naïve CHB patients across distinct clinical phases. Our results demonstrated that hepatic miRNA profiles remain relatively stable throughout the natural course of CHB. Despite substantial changes in immune cell infiltration and immune-related gene expression, the majority of hepatic miRNAs showed consistent expression across the IT, IA, IC, and ENH phases. The primary alterations in miRNA expression were consistently associated with lymphocyte infiltration and immune activation during the immune-active phases. This is consistent with the fact that activated immune cells have high expression levels of hsa-miR-155-5p and a distinct set of immune-related miRNAs and are recruited into the liver during inflammation. However, these associational observations do not establish causality alone.

Our results suggest that the relative stability of hepatic miRNA expression, even under conditions of chronic viral infection and immune activation, may reflect the importance of maintaining cellular miRNA homeostasis. Such stability may help preserve cellular identity and function in the liver despite dynamic inflammatory and virological changes during CHB progression. Previous studies have discussed the potential use of selected miRNAs for liver-directed antiviral treatment or for regulating hepatic functions^31,32^. In this context, our findings support the view that therapeutic manipulation of miRNA levels should be approached with caution, because artificial perturbation of miRNA expression may alter cellular differentiation states and broader regulatory programs.

It is well established that miRNAs exhibit tissue- and cell-type–associated expression patterns^6,7,33,34^. In previous work, we characterized miRNA expression profiles in key hepatic cell types, including PHHs, KCs, LSECs, HSCs, and identified distinct sets of cell type–enriched miRNAs^7^. Accordingly, hepatic miRNAs can serve as molecular markers reflective of cellular identity. In this study, many hepatocyte-enriched miRNAs were found to be highly abundant in liver biopsy samples (Fig. 2a), consistent with the hepatocyte-dominated composition of liver tissue.

Although miRNA levels showed slight variability between patients, interindividual differences were relatively minor. In contrast to the significant variability observed in mRNA expression profiles among patient samples, the expression of miRNAs enriched in PHHs, HSCs, or LSECs exhibited only minor changes across the four clinical phases. Given that miRNAs regulate multiple target genes post-transcriptionally^4^, they play a crucial role in fine-tuning gene expression and maintaining cellular homeostasis in response to environmental stimuli^5^. Therefore, the overall stability of hepatic miRNA profiles likely reflects their function in buffering transcriptional fluctuations, preserving cellular identity, and maintaining liver function despite dynamic changes in viral replication and immune activation during CHB progression.

The most prominent changes in hepatic miRNA expression were observed when comparing liver biopsies from patients with hepatitis to those without hepatitis, both in the current study and in previously published data^15^. Earlier transcriptomic analyses of CHB liver biopsies demonstrated marked differences in hepatic gene expression, primarily attributed to immune cell infiltration^19–22^. Similarly, we found that several intrahepatic miRNAs were upregulated in the IA phase compared to the IT phase. Among them, miR-155—one of the most abundant miRNAs in lymphocytes—was significantly enriched in KCs relative to other hepatic cell types^7^. MiR-155 has been implicated in inflammatory cell–mediated liver injury^35^, and in our analysis, intrahepatic miR-155-5p expression correlated positively with several immune-related genes, including HLA-B, CD53, CXCR4, LCP2, GZMA, HLA-DRA, and HLA-DMA. Other immune-related miRNAs such as miR-142-5p and miR-150 were also found to be enriched in leukocytes and are known to regulate inflammation and T cell differentiation^33,36–38^. These miRNAs were upregulated in the IA phase relative to the IT phase, consistent with increased immune cell infiltration and activation in the liver.

**Fig. 5:**
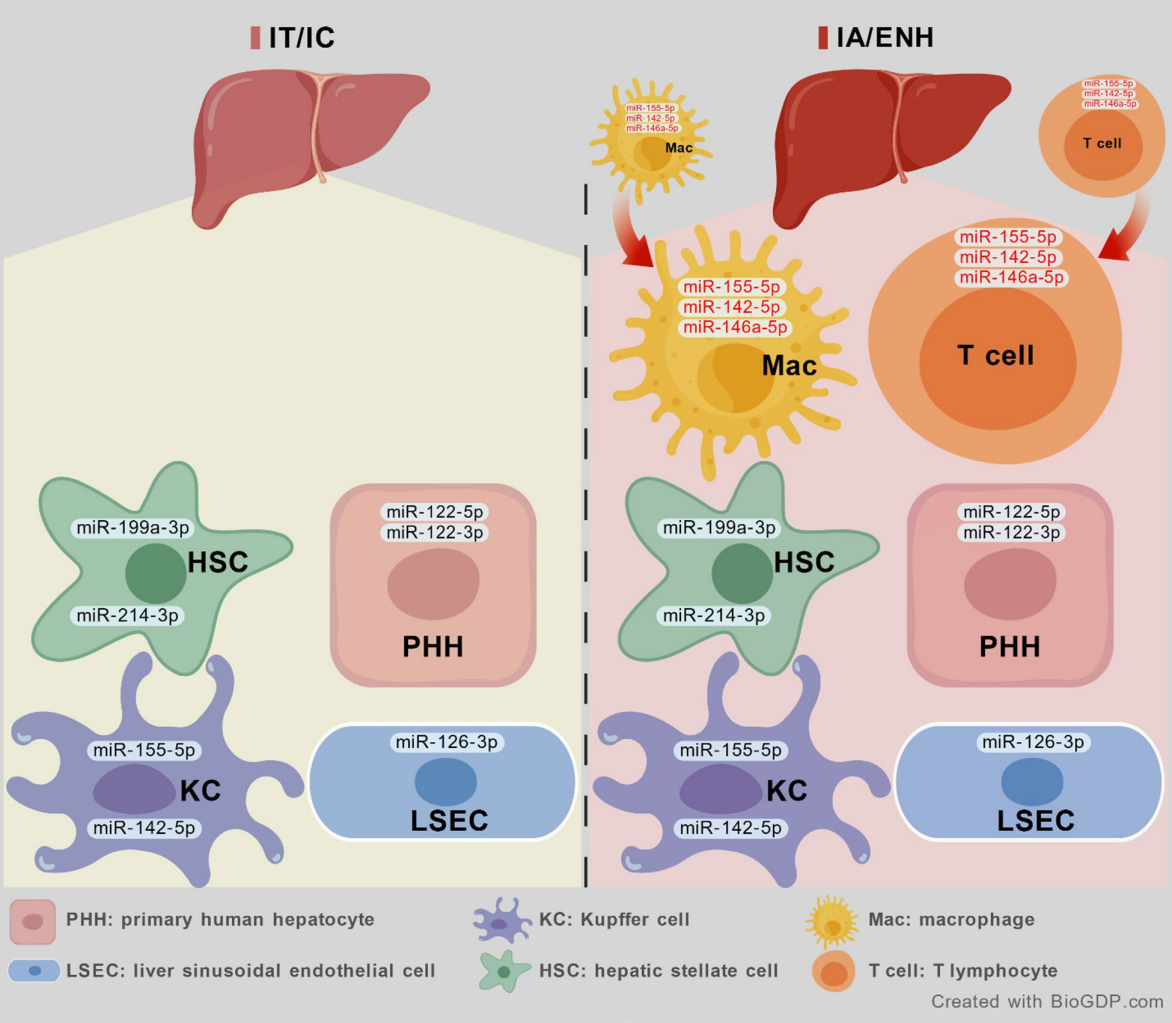
Schematic overview of intrahepatic miRNA dynamics in IT/IC versus active IA/ENH phases. In the IT/IC phase (left), resident PHHs (enriched in miR‑122‑5p, miR‑122‑3p, etc.), LSECs (enriched in miR‑126‑3p, etc.), HSCs (enriched in miR‑199a‑3p, miR‑214‑3p, etc.), and KCs (enriched in miR‑155‑5p, miR‑142‑5p, etc.) are likely to maintain a stable hepatic miRNA landscape. In the IA/ENH phases (right), infiltrating immune cells represented by T cells and macrophages are likely to contribute immune-related miRNAs such as miR-155-5p, miR-142-5p, and miR-146a-5p to the intrahepatic miRNA profile. This figure was created using BioGDP.com (www.biogdp.com). Abbreviations are as defined in the text.

Two mechanisms likely contribute to the observed dynamics of intrahepatic miRNA expression: (1) infiltration of immune cells—such as macrophages, T cells, and B cells—alters the cellular composition of the liver; and (2) miRNA expression within immune cells themselves may be modulated upon activation^5,21^(Fig. S2a and b). As summarized in our schematic model of IT/IC versus IA/ENH states (Fig. 5), resident PHHs, LSECs, HSCs, and KCs are likely to maintain a largely stable miRNA landscape in the IT/IC phases. In the IA/ENH phases, immune cell infiltration represented by T cells and macrophages is likely to contribute immune‑related miRNAs such as miR‑155‑5p, miR‑142‑5p, miR‑146a‑5p to the intrahepatic miRNA profile. This dual mechanism may explain the accumulation of immune-related miRNAs like miR-155, which peaked during the IA phase (Figs. 2c and d and 3a, and 4b). Notably, both miR-155 expression and immune marker gene levels were highest in the IA phase, consistent with enhanced immune activation in this phase (Fig. 4b).

Although the IA and ENH groups differed in serum ALT and HBV DNA levels, their broadly similar intrahepatic miRNA profiles are consistent with the overall pattern observed in our study. The two low-inflammatory phases, IT and IC, also showed highly similar hepatic miRNA profiles despite marked differences in viral replication status, whereas the two inflammatory phases, IA and ENH, shared similarly shaped miRNA profiles despite differences in ALT and serum HBV DNA. Taken together, these findings suggest that peripheral viral replication levels are not closely reflected by hepatic miRNA patterns in CHB. This observation is in line with previous studies showing that intrahepatic inflammatory and transcriptomic patterns across CHB phases correlated more closely with serum ALT than with serum HBV DNA or viral proteins^19,21^.

Circulating serum miRNAs have also been associated with HBV infection and immune activation^39,40^. In CHB patients, HBsAg particles can carry miRNAs such as miR-122, miR-223, and miR-145^41^. Serum miRNA levels are known to correlate with intrahepatic miRNA expression, reflecting the hepatic miRNA profile to a certain extent^42^. Many PHH-enriched miRNAs identified in this study—including those previously reported—are elevated in the serum of HBV-infected patients compared to healthy individuals and are more abundant in HBeAg-positive than HBeAg-negative patients^39,40,43^. In our study, serum levels of miR-122-5p, miR-125b, miR-124, and miR-193b-3p positively correlated with HBV DNA load and HBsAg levels^7,44,45^. In addition, miR-155-5p expression—highly abundant in lymphocytes—tended to be higher in the IA phase than in the IT phase, likely reflecting infiltration of HBV-specific T cells into the liver. HSC-enriched miR-214-3p in serum has also been associated with the severity of liver fibrosis^46^. Collectively, these observations suggest that hepatic cell type–associated miRNAs may have potential as non-invasive biomarkers for HBV-related liver disease, though further validation is warranted.

Singh et al. previously profiled hepatic miRNAs in liver biopsy samples from HBV-infected patients and reported distinct miRNA patterns across different HBV-related disease states in relation to viral replication, liver injury, and fibrosis, with healthy controls included as a reference^15^. In our previous work, we focused primarily on hepatic cell type–enriched miRNA expression^7^. In contrast to these earlier studies, the present study adds a different and more phase-resolved perspective by systematically comparing the intrahepatic miRNA landscape across the four clinically defined CHB phases (IT, IA, IC, and ENH) within a single set of treatment-naïve liver biopsy samples and by integrating paired miRNA microarray and mRNA-seq data from the same biopsies. Thus, the main biological insight provided by this study is not mechanistic proof, but a phase-resolved and integrative view of the intrahepatic miRNA landscape across CHB, showing that the overall hepatic miRNA background is relatively stable, whereas the major phase-associated changes are largely confined to immune-related miRNAs in the inflammatory phases. In addition to identifying differentially expressed miRNAs, this study places the intrahepatic miRNA landscape into the context of phase-specific immune infiltration, hepatic transcriptomic changes, and the broader clinical characteristics of the different CHB phases.

A limitation of this study is the absence of healthy liver controls, because all liver samples were collected for clinical indications. Therefore, the relative stability described here refers to comparisons across CHB clinical phases rather than to uninfected liver. We also considered a published dataset including healthy liver and CHB liver samples as an external point of reference. In that comparison, some immune-associated miRNAs showed higher abundance in CHB than in healthy liver, indicating a broadly similar overall direction to that observed in our study. However, because this dataset was generated using a different platform and was not annotated according to the CHB phase classification used in our study, it could not be directly compared with the phase-associated structure observed in our study and was therefore not directly integrated into the present analysis. The lack of significantly differentially expressed miRNAs between the IT and IC groups may reflect the biological similarity of these two low-inflammatory states, but it may also partly relate to the limited sample size and statistical power of this comparison. Because the analyses were performed on bulk liver biopsy tissue, part of the observed phase-associated differences in immune-related miRNAs may reflect shifts in cellular composition rather than purely cell-intrinsic regulation. Moreover, the measured miRNA signals are likely to reflect a combination of intracellular miRNA expression and extracellular miRNAs present in the tissue microenvironment, including those associated with extracellular vesicles, exosomes, or cell damage. As a result, some of the observed phase-associated miRNA changes may also reflect extracellular accumulation or release within inflamed liver tissue. Although LM22 provides only a relative estimate of immune cell infiltration in bulk liver tissue and does not include major liver-resident populations, our interpretation was not based on LM22 alone, but considered together with the 92 intrahepatic marker genes, histological features, clinical phase information, miRNA–marker correlations, and external human cellular microRNAome reference data. At the same time, the overall phase-associated pattern observed here—namely stronger intrahepatic inflammation and immune activation in the IA and ENH phases than in the IT and IC phases—has also been repeatedly reported in previous transcriptomic and integrative studies of CHB liver tissue, including our recent work^19–21^. In particular, several of the top immune-associated miRNAs identified here showed marked enrichment in immune lineages in a human cellular microRNAome resource covering 196 primary cell types, supporting their association with immune cell–related patterns in CHB liver tissue^34^. Additional orthogonal validation, including immunohistochemistry, would further strengthen these observations; however, the main limitation of the present analysis lies in the bulk-tissue nature of the biopsy samples rather than in the absence of a single conventional validation assay. Future studies using single-cell and other cell-resolved approaches will be important to determine whether the relative stability of hepatic miRNA expression is maintained across specific liver cell populations and to define more directly the immune cell–associated miRNA expression patterns underlying the phase-related changes observed here. In addition, spatial transcriptomic approaches may help clarify how intrahepatic cell distribution and inflammatory microenvironments are related to the observed miRNA patterns. Finally, serum HBV DNA and HBsAg represent peripheral measurements, while intrahepatic viral burden, such as cccDNA or integrated HBV DNA, was not measured in the same biopsies. Accordingly, the relationship between intrahepatic viral activity and hepatic miRNA expression remains unresolved in the present study.

In summary, our findings suggest that hepatic miRNA expression profiles are largely shaped by the composition of liver-resident cells and remain relatively stable across CHB phases. The principal changes are associated with immune cell infiltration and activation, particularly in the immune-active phases, highlighting the close relationship between the immune response and hepatic miRNA regulation in CHB.

## Supplementary Information

Below is the link to the electronic supplementary material.

Supplementary Material 1

Supplementary Material 2

## Data Availability

The RNA-seq data generated in this study have been deposited in the Gene Expression Omnibus (GEO) under accession number GSE298398. The hepatic miRNA expression data used in this study are provided in the Supplementary Tables (Tables S9 and S10).

## Abbreviations

AASLD: American Association for the Study of Liver Diseases
ALT: Alanine aminotransferase
CHB: Chronic hepatitis B
CIBERSORT: Cell-type identification by estimating relative subsets of RNA transcripts
ENH: HBeAg-negative hepatitis
FC: Fold change
FDR: False discovery rate
FPKM: Fragments per kilobase of transcript per million mapped reads
GEO: Gene Expression Omnibus
HBV: Hepatitis B virus
HCC: Hepatocellular carcinoma
HSC: Hepatic stellate cell
IA: Immune active
IC: Inactive carrier
IT: Immune tolerance
KC: Kupffer cell
LSEC: Liver sinusoidal endothelial cell
PCA: Principal component analysis
PHH: Primary human hepatocyte
