# Supplementary material for: The microRNA (miRNA) signatures in different phases of the natural history of chronic hepatitis B virus infection
Source: Sci Rep. 2026 May 29;16:24608. doi: 10.1038/s41598-026-53116-z (PMC13454168; doi:10.1038/s41598-026-53116-z)
Supplement: Supplementary file 1 [file 41598_2026_53116_MOESM1_ESM.pdf]

The microRNA (miRNA) signatures in different phases of the natural history of chronic hepatitis B virus infection

Qiong Wang, [†,1] Fahong Li, [†,2] Yurong Yao, [†,3] Jinyu Wang, [2] Jun Wang, [4] Yong Lin, [5] Ting Wang, [3] Jiming Zhang, [*,2] Mengji Lu[*,1]

Supplementary Tables

Table S1. Clinical and virological features of chronic hepatitis B patients in the study.

Table S2. mRNA expression levels (FPKM) of 92 marker genes for 12 intrahepatic cell types in CHB patients across different phases.

Table S3. mRNA expression levels (FPKM) of 110 immune-related genes in CHB patients across different phases.

Table S4. Medium of normalized expression values of hepatic miRNAs across CHB clinical phases.

Table S5. Differentially expressed miRNAs (n = 273) in pairwise comparisons across CHB clinical phases.

Table S6. 22 significantly up-regulated miRNAs in IA phase compared to IT and IC phases.

Table S7. miRNAs present only in IA and/or ENH phases.

Table S8. Differential expression of 39 hepatic cell type–enriched miRNAs across CHB phases.

Table S9. Normalized expression values of 559 hepatic miRNAs in liver tissue from chronic hepatitis B patients (IT and IA phases).

Table S10. Normalized expression values of 559 hepatic miRNAs in liver tissue from chronic hepatitis B patients (IC and ENH phases).

Table S1. Clinical and virological features of chronic hepatitis B patients in the study.

Group	IT	IA	IC	ENH
N samples	10	10	13	10
Age, years (Median, IQR)	35 (30.75，36)	29 (23.5，32.5)	41 (33，45)	30.5 (27，42.75)
Male, n/N	8/10	8/10	8/13	8/10
HBeAg-positive	10/10	10/10	0/13	0/10
G-score (0–1), n/N	10/10	1/10	13/13	1/10
G-score (2–4), n/N	0/10	9/10	0/13	9/10
S-score (0–1), n/N	10/10	4/10	12/13	3/10
S-score (2–4), n/N	0/10	6/10	1/13	7/10
ALT (U/L) (Median, IQR)	35(21，49)	196 (138.75, 301.25)	30 (17，42)	89 (61.75，115.25)
HBsAg ($\log_{10}$ IU/ml) (Median, IQR)	4.72 (3.64，4.72)	4.39 (3.98，4.65)	3.01 (2.40，3.35)	3.23 (3.08，3.45)
HBV DNA ($\log_{10}$ IU/ml) (Median, IQR)	7.48 (7.08，7.70)	7.39 (6.71，7.48)	2.70 (2.70，2.76)	5.58 (4.67，6.27)

Categorical variables as n/N; continuous variables are shown as median (IQR). G-score and S-score are histological grading and staging scores according to Scheuer criteria.

Abbreviations: IT, immune tolerance; IA, immune active; IC, inactive carrier state; ENH, HBeAg-negative active chronic hepatitis; ALT, alanine aminotransferase; IQR, interquartile range.

Table S2. mRNA expression levels (FPKM) of 92 marker genes for 12 intrahepatic cell types in CHB patients across different phases.

Cell	ID	IT_1	IT_2	IT_3	IT_4	IT_5	IT_6	IT_7	IT_8	IT_9	IT_10	IA_1	IA_2	IA_3	IA_4	IA_5	IA_6	IA_7	IA_8	IA_9	IC_1	IC_2	IC_3	IC_4	IC_5	IC_6	IC_7	IC_8	IC_9	IC_10	IC_11	IC_12	IC_13	ENH_1	ENH_2	ENH_3	ENH_4	ENH_5	ENH_6	ENH_7	ENH_8	ENH_9
B_cells	LTB	0.77	0.83	0.65	0.40	0.41	0.98	0.81	0.97	2.48	0.52	2.34	2.17	0.64	1.42	1.07	1.75	1.69	1.80	0.99	0.76	0.75	1.07	0.94	0.86	0.67	0.52	1.00	0.56	0.50	0.54	0.57	0.77	1.64	1.37	1.08	1.67	2.21	0.94	0.75	1.56	1.03
B_cells	CD37	0.55	0.37	0.57	0.46	0.30	0.78	0.51	0.32	1.11	0.54	1.52	1.36	0.78	0.87	1.24	1.40	1.30	1.00	0.66	0.61	0.36	0.70	0.63	0.35	0.44	0.38	0.74	0.48	0.33	0.32	0.50	0.56	1.29	0.91	0.53	1.17	1.05	0.50	0.37	0.62	0.66
B_cells	MS4A1	0.30	0.15	0.09	0.11	0.13	0.35	0.26	0.09	0.18	0.11	0.56	0.42	0.34	0.45	0.20	0.90	0.20	0.68	0.23	0.17	0.12	0.39	0.27	0.13	0.15	0.28	0.20	0.11	0.17	0.13	0.22	0.24	0.62	0.44	0.13	0.42	0.55	0.21	0.16	0.34	0.30
B_cells	CD79B	0.74	0.72	0.48	1.12	0.65	0.50	0.33	0.60	0.58	0.46	1.10	0.72	0.88	1.03	0.75	1.32	0.83	0.93	0.60	0.51	0.60	0.66	0.63	0.53	0.72	0.70	0.51	0.83	0.68	0.64	0.70	0.73	1.54	0.81	0.57	0.69	1.03	0.49	0.53	0.75	0.57
Gamma_delta_T_cells_2	SLBP	2.45	2.14	3.00	2.53	2.62	2.83	2.49	2.73	1.87	3.76	3.70	2.70	2.43	2.64	3.40	2.81	4.28	2.37	2.76	2.34	2.65	1.93	2.61	2.38	2.51	2.35	3.21	2.70	2.44	2.51	2.18	2.53	3.20	2.93	2.50	2.87	2.30	3.02	2.49	2.77	2.58
Gamma_delta_T_cells_2	STMN1	0.58	0.55	1.03	0.59	0.48	0.34	0.64	0.55	0.91	0.20	1.77	1.37	0.69	0.91	1.22	1.79	2.67	0.74	0.64	0.63	0.54	0.64	0.67	0.54	0.52	0.60	0.57	0.64	0.40	0.48	0.65	0.53	1.44	1.48	0.78	0.68	0.61	0.66	0.58	1.05	0.65
Gamma_delta_T_cells_2	HMGB2	0.78	0.65	0.80	0.63	0.39	0.48	0.94	0.45	0.57	0.36	1.12	0.82	0.45	0.72	0.88	0.92	1.89	0.76	0.43	0.64	0.41	0.49	0.66	0.52	0.51	0.36	0.69	0.58	0.42	0.45	0.61	0.63	1.05	0.74	0.67	0.66	0.66	0.50	0.45	0.65	0.56
Gamma_delta_T_cells_2	TYMS	1.04	0.80	1.47	0.73	0.57	0.60	0.82	0.81	0.37	0.27	6.24	4.31	1.87	1.54	3.70	2.85	8.85	1.19	2.22	0.38	0.80	1.19	1.05	0.24	0.89	0.93	0.96	1.67	0.56	0.59	0.41	0.44	3.99	3.26	1.82	1.20	1.23	2.30	0.56	1.84	0.92
Alpha_beta_T_cells	GZMK	2.80	1.23	1.96	2.17	1.21	2.93	2.48	1.84	3.54	2.00	4.66	6.76	5.12	4.71	6.45	7.71	6.87	4.33	3.07	1.32	1.44	3.75	2.10	2.29	3.53	1.87	2.94	3.88	1.70	2.45	1.67	2.26	6.06	5.27	2.30	6.27	2.50	2.12	1.41	2.83	3.32
Alpha_beta_T_cells	CD8A	0.68	1.00	0.91	0.68	0.46	1.01	1.24	0.78	0.83	0.55	3.64	2.78	2.91	2.46	3.64	4.43	5.32	1.31	1.04	1.23	0.59	1.59	1.25	0.68	1.13	0.54	1.94	1.27	0.42	0.87	0.59	0.55	3.72	2.27	1.20	2.55	1.74	1.62	0.58	1.16	1.02
Alpha_beta_T_cells	PTPRC	8.26	5.44	7.76	7.82	4.77	9.81	8.63	5.25	5.94	3.69	15.59	15.96	9.11	10.82	17.12	16.29	15.05	12.36	9.53	6.31	5.87	9.97	8.21	5.40	7.70	4.94	8.44	5.52	6.14	5.68	5.52	6.44	17.64	12.20	8.83	11.66	10.83	7.84	4.87	8.37	7.17
Alpha_beta_T_cells	CD3D	1.21	1.32	1.23	0.91	0.46	1.05	1.61	1.18	3.37	0.72	4.24	3.17	3.66	2.62	4.70	4.55	5.12	3.08	1.20	0.91	0.62	2.28	1.98	0.69	1.48	1.24	2.02	2.13	1.01	1.04	0.59	0.86	4.50	3.53	1.41	3.02	2.51	1.84	0.61	1.46	1.53
Alpha_beta_T_cells	CD3E	1.19	0.74	1.01	1.01	0.66	1.37	1.09	0.91	1.55	0.55	2.77	2.52	2.49	1.70	3.40	3.23	3.75	2.13	1.34	0.97	0.93	1.59	1.45	0.72	0.95	0.80	1.34	0.94	0.95	0.96	0.72	0.89	2.61	1.96	1.14	2.30	1.78	1.52	0.82	1.52	1.29
Alpha_beta_T_cells	CD2	1.84	1.73	1.66	1.28	1.09	1.73	1.76	1.01	3.07	0.40	3.50	3.67	3.41	2.29	3.56	4.32	4.64	3.06	1.52	1.61	1.54	2.80	2.20	0.82	2.01	0.99	2.68	1.76	1.34	1.23	1.10	1.58	4.89	3.50	2.29	3.27	2.87	1.92	1.58	1.42	1.65
Alpha_beta_T_cells	TRAC	3.14	2.24	3.74	3.06	2.05	4.61	4.04	2.51	2.50	4.46	10.24	9.17	4.40	6.97	10.10	12.48	12.19	5.67	3.36	2.66	2.14	6.03	3.92	1.19	3.03	2.03	5.03	3.00	2.24	1.91	1.65	2.63	10.61	7.36	4.14	8.57	5.51	4.54	1.66	4.67	3.54
Gamma_delta_T_cells1/NK_like_cells	GNLY	0.31	0.20	0.19	0.17	0.18	0.30	0.21	0.13	0.36	0.25	0.34	0.26	0.27	0.29	0.22	0.20	0.38	0.25	0.29	0.20	0.34	0.28	0.46	0.15	0.44	0.36	0.33	0.39	0.32	0.28	0.18	0.21	0.16	0.40	0.20	0.37	0.14	0.21	0.32	0.57	0.44
Gamma_delta_T_cells1/NK_like_cells	NKG7	2.36	2.28	2.51	1.49	0.72	3.15	1.79	1.78	1.90	3.85	5.68	4.40	3.12	3.21	7.03	4.06	7.29	2.73	2.02	1.94	1.60	1.68	2.38	1.43	2.17	1.51	2.15	3.27	1.62	2.16	1.16	1.79	5.09	3.31	1.65	3.92	1.85	2.75	1.41	2.61	1.96
Gamma_delta_T_cells1/NK_like_cells	KLRB1	2.25	2.02	1.72	1.47	1.27	2.67	1.79	1.86	1.12	1.11	3.58	4.14	1.26	2.55	3.67	2.35	5.11	2.59	2.57	3.01	2.17	1.86	2.00	3.14	2.11	1.02	3.20	3.50	1.60	2.20	1.56	1.87	4.37	2.56	2.06	2.82	2.27	2.18	1.01	2.07	1.83
Gamma_delta_T_cells1/NK_like_cells	TRDC	4.74	3.67	3.78	6.26	2.66	5.00	2.64	4.09	1.22	1.47	3.67	5.87	1.96	2.00	3.95	4.40	5.93	5.41	2.68	3.17	3.73	2.52	4.30	1.97	3.06	2.25	2.85	6.07	2.99	3.14	1.99	2.95	3.65	2.96	2.16	4.34	2.54	1.26	1.52	3.43	3.88
Gamma_delta_T_cells1/NK_like_cells	CD7	0.46	0.33	0.38	0.35	0.20	0.55	0.29	0.43	0.50	0.64	0.88	0.92	0.70	0.35	1.00	0.59	0.48	0.45	0.35	0.38	0.34	0.34	0.49	0.36	0.40	0.29	0.29	0.74	0.24	0.32	0.35	0.57	0.31	0.52	0.43	0.89	0.44	0.39	0.27	0.37	0.34
Plasma_cells	MZB1	0.36	0.20	0.24	0.25	0.04	0.51	0.49	0.13	0.42	0.26	1.33	0.40	1.00	0.66	1.03	1.12	1.38	0.81	0.33	0.27	0.34	0.56	0.21	0.13	0.25	0.18	0.48	0.36	0.14	0.07	0.39	0.42	2.00	1.34	0.33	0.42	1.68	0.54	0.16	0.46	0.44
Plasma_cells	IGHG1	92.58	12.14	58.78	23.75	15.14	52.03	105.57	12.98	48.43	52.79	185.37	118.34	481.94	264.68	129.78	189.71	409.89	104.37	78.68	43.97	42.77	59.26	56.98	8.01	32.47	11.17	57.42	56.61	29.26	6.82	32.34	41.40	326.04	376.91	123.06	94.77	674.06	139.44	10.56	30.52	93.19
Plasma_cells	IGLC2	66.94	17.64	36.61	41.57	12.88	36.82	84.08	28.23	49.20	26.99	169.69	53.97	192.64	89.26	97.99	219.00	315.80	106.38	44.67	40.16	41.24	65.36	59.93	18.38	24.29	16.58	67.37	107.57	17.00	5.70	51.70	59.09	339.34	312.57	73.09	67.24	221.21	77.77	23.62	37.79	36.88
Plasma_cells	IGKC	36.88	13.94	22.86	44.55	11.21	47.67	81.79	11.16	46.40	86.14	124.80	52.96	131.44	70.03	94.60	146.01	107.66	139.54	38.43	17.26	37.37	58.02	26.55	10.32	17.39	12.82	54.62	31.06	11.82	5.21	59.47	47.90	276.73	213.58	53.01	49.07	243.19	47.13	20.26	43.69	36.20
nMac	MAFB	22.30	23.08	26.62	23.66	17.15	30.85	19.52	22.97	19.98	24.72	25.82	28.09	26.11	38.06	29.69	22.56	30.42	28.18	21.64	25.42	27.88	17.44	22.42	17.40	23.84	18.94	19.64	26.40	19.89	26.16	18.19	28.41	28.58	30.05	21.61	25.91	23.57	22.96	23.67	20.44	20.08
nMac	CD5L	46.88	43.64	77.47	66.27	30.75	56.99	54.16	49.60	4.96	50.61	76.80	125.84	57.38	89.03	95.51	72.12	136.87	67.37	55.77	35.19	32.37	34.46	66.71	48.33	61.19	28.22	42.99	118.98	38.07	45.74	36.06	37.57	92.49	119.51	48.40	62.18	41.01	42.60	30.14	68.71	28.59
nMac	MARCO	13.51	21.30	44.74	34.62	22.59	19.25	22.57	21.21	1.72	33.33	38.33	53.05	20.41	27.98	74.08	36.42	78.87	29.06	23.94	21.54	27.13	38.98	25.10	16.44	35.84	11.89	16.55	39.59	20.33	17.14	24.53	20.57	63.40	40.47	27.02	31.17	28.86	18.13	15.31	22.16	19.37
nMac	VSIG4	6.17	8.80	14.32	15.62	8.81	11.95	13.61	9.69	0.79	7.31	14.37	17.81	10.34	19.64	24.78	12.26	23.09	13.41	18.42	8.93	13.46	12.81	11.14	8.09	11.44	7.00	10.33	17.63	8.27	9.65	9.71	9.58	20.53	15.14	9.86	12.20	10.97	9.53	7.49	9.96	7.46
nMac	CD163	15.12	16.85	27.22	26.40	17.66	21.61	26.17	16.58	2.97	15.23	35.94	35.41	22.35	35.80	38.20	34.70	34.72	26.05	36.78	14.94	17.27	20.64	19.78	19.73	21.01	15.53	18.98	20.47	22.69	20.24	19.84	20.97	37.87	32.54	22.45	29.46	29.00	24.40	17.39	25.30	17.69
iMac	S100A8	1.22	2.45	2.50	1.64	1.15	1.72	1.18	1.95	0.40	0.08	1.77	2.39	1.34	1.44	1.50	0.57	0.68	2.24	1.92	0.99	1.76	1.68	2.17	2.08	1.33	2.42	1.32	2.10	1.21	1.23	1.50	0.94	0.71	0.86	1.55	1.03	2.02	0.86	0.96	1.77	1.42
iMac	S100A9	1.11	6.34	11.52	4.12	1.11	7.80	3.09	3.48	0.70	2.70	4.49	9.58	2.65	4.40	5.79	1.86	4.50	4.88	4.23	2.96	5.59	2.64	8.49	4.12	4.56	4.17	4.11	7.58	3.29	3.73	2.62	2.58	5.29	3.52	2.91	2.65	4.68	2.78	2.62	4.39	3.23
iMac	LYZ	9.24	9.81	14.68	8.51	7.98	17.12	16.72	8.98	7.63	15.57	39.40	41.25	11.01	15.15	23.62	16.89	20.73	14.00	14.93	10.08	9.58	15.17	13.63	6.00	11.68	7.79	16.42	14.25	10.40	5.13	5.39	13.40	25.86	35.01	10.56	21.07	12.69	5.94	8.20	14.84	14.75
iMac	HLA-DPB1	5.70	4.98	14.51	9.47	5.47	7.32	11.46	8.62	3.76	5.08	27.47	17.74	8.98	15.93	27.23	21.00	32.62	18.65	16.62	8.37	10.63	8.38	13.78	3.71	9.59	7.55	9.85	10.75	10.98	8.87	7.19	7.74	20.43	21.34	11.85	21.61	17.73	13.76	6.90	8.66	10.94
HSC	S100A13	1.14	1.05	1.17	1.38	1.17	1.05	1.78	1.43	1.13	0.69	2.27	1.45	1.41	1.18	1.88	1.94	2.25	1.25	1.97	1.54	1.37	1.21	1.13	1.06	1.08	1.54	0.97	2.23	1.17	1.27	0.88	1.77	1.89	2.23	1.47	1.40	1.71	1.76	1.46	1.00	1.52
HSC	RBP1	2.19	2.30	3.05	2.03	1.90	1.58	3.07	2.14	1.30	2.47	6.70	2.72	2.55	2.24	2.33	2.75	3.64	2.61	3.11	2.19	1.88	2.58	2.67	2.46	2.14	2.29	2.38	4.83	2.12	1.68	2.46	2.83	2.13	4.59	2.58	3.04	3.24	2.33	1.69	3.00	2.19
HSC	SPARCL1	1.63	0.92	1.07	1.90	1.34	1.16	1.68	0.80	1.10	0.44	0.87	0.79	1.15	0.85	2.18	1.79	2.05	1.10	0.79	1.05	1.52	1.71	0.74	1.35	1.07	0.67	1.57	0.80	1.09	1.62	1.80	1.94	1.54	1.19	2.58	0.63	1.02	0.75	0.70	0.67	1.30
HSC	COL3A1	31.25	20.12	20.89	15.52	16.07	19.14	24.64	16.80	20.09	28.86	148.33	40.61	35.77	18.68	32.79	40.90	40.28	24.13	40.12	19.49	17.34	22.77	21.43	18.15	20.76	16.83	18.31	17.53	20.43	17.13	30.83	22.50	32.13	31.26	39.46	23.84	51.69	24.94	13.36	29.68	22.61
HSC	CYGB	1.87	2.87	3.85	2.08	2.33	2.82	3.04	2.31	2.34	5.73	4.65	5.38	5.20	2.42	3.87	3.08	5.10	3.23	4.20	2.28	1.59	3.40	2.27	2.49	2.78	3.34	2.27	4.44	2.32	2.51	3.06	3.47	4.28	5.49	3.12	2.94	3.53	4.24	2.19	2.99	3.30
HSC	ACTA2	9.71	5.86	2.58	3.38	2.49	3.77	9.50	2.46	5.42	2.83	10.09	4.10	6.46	5.15	6.59	8.88	11.28	6.07	5.30	6.42	6.53	5.19	6.53	4.26	4.85	4.57	5.05	4.99	3.30	3.10	6.59	12.11	9.32	6.91	8.95	3.66	6.57	8.06	3.30	4.50	6.78
HSC	COL1A1	9.80	4.64	3.72	3.71	3.34	4.86	6.86	4.03	10.27	10.05	82.22	15.07	17.64	6.65	14.77	13.42	14.30	7.37	14.61	5.28	3.87	4.72	6.06	4.39	4.85	4.55	4.74	4.07	[illegible]	[illegible]	9.16	7.42	9.96	9.18	15.26	7.81	19.01	8.17	3.54	10.47	8.46
Cholangiocyte	RND3	3.69	3.71	2.62	4.23	3.52	3.74	4.10	2.73	0.85	1.96	6.80	4.45	3.99	2.22	3.66	3.56	3.80	4.56	5.23	2.71	4.38	3.50	2.77	6.15	3.42	4.22	5.72	3.50	3.81	2.95	2.48	3.06	3.85	5.76	4.34	2.72	5.66	2.69	3.63	2.93	3.67
Cholangiocyte	KRT19	0.35	0.32	0.51	0.84	0.55	0.19	0.74	0.32	1.01	0.25	2.74	0.53	0.71	0.78	0.92	0.48	0.71	0.31	1.46	0.39	0.32	1.13	0.25	0.14	0.46	0.37	1.69	0.22	0.53	0.42	1.06	0.62	1.34	1.02	2.34	0.43	0.76	0.80	0.13	0.32	0.75
Cholangiocyte	AKR1C1	14.80	19.68	19.53	22.24	18.36	13.91	14.59	18.94	14.18	19.23	22.37	17.16	18.03	26.15	16.68	27.35	14.99	24.59	11.67	16.98	19.17	17.64	20.25	18.83	18.55	12.18	22.33	23.44	13.12	11.29	19.84	20.97	18.50	17.80	14.90	14.61	18.14	18.41	14.05	16.11	25.99
Cholangiocyte	KRT18	20.35	33.49	24.88	13.48	14.94	16.34	17.75	19.61	13.13	19.76	54.72	28.99	32.23	35.24	42.12	17.44	58.61	18.74	36.72	15.18	18.47	21.11	24.59	12.72	17.00	15.01	20.08	25.88	16.69	13.29	17.89	22.85	42.67	26.42	26.65	28.90	26.44	32.18	18.62	24.78	18.87
Cholangiocyte	SOX9	3.05	1.04	1.44	1.22	1.37	2.29	1.74	0.93	4.61	1.12	20.42	3.61	8.27	2.58	5.61	4.89	6.32	1.96	9.74	1.82	0.91	4.67	2.03	1.14	3.08	1.12	2.05	1.39	2.34	0.71	1.71	2.65	6.13	3.86	4.28	2.98	4.65	4.20	1.01	5.48	3.22
Cholangiocyte	EPCAM	0.56	0.14	0.19	0.11	0.07	0.21	0.44	0.03	0.11	0.12	3.49	0.26	0.49	0.24	0.20	0.57	0.53	0.29	1.89	0.30	0.12	0.78	0.30	0.24	0.17	0.17	0.41	0.17	0.41	0.15	0.45	0.46	0.64	0.59	0.94	0.22	2.53	0.59	0.04	0.41	0.43
Cholangiocyte	KRT7	1.11	0.46	0.49	0.26	0.26	0.58	1.04	0.21	1.48	0.31	8.28	1.17	1.69	0.64	0.46	2.03	1.57	0.99	2.98	0.80	0.42	1.85	0.96	0.50	0.46	0.44	1.29	0.49	1.01	0.21	0.82	0.64	1.58	1.31	2.72	0.55	2.15	1.84	0.13	1.16	1.45
Cholangiocyte	CFTR	0.97	0.48	0.74	0.47	0.58	0.57	1.48	0.32	1.59	0.40	9.09	1.88	1.93	0.94	0.81	1.81	0.93	0.90	1.83	0.93	0.57	1.78	0.85	0.42	0.42	0.51	1.65	0.23	0.88	0.35	1.15	1.38	2.07	1.61	2.25	0.92	3.01	1.65	0.48	1.15	1.24
Endothelial_cells	INMT	4.66	3.16	3.26	4.06	2.59	2.40	7.47	5.07	4.57	2.93	13.21	5.12	6.15	2.69	11.15	9.54	8.34	4.18	5.81	5.40	5.32	6.82	6.73	3.41	5.92	2.29	5.49	6.30	8.51	3.51	3.21	4.95	5.93	6.88	4.81	4.13	8.24	4.92	1.87	3.23	4.08
Endothelial_cells	RAMP3	8.65	7.76	7.30	5.93	7.72	3.26	6.93	6.07	7.10	5.09	9.00	7.43	4.98	4.86	7.68	10.57	15.40	7.06	7.01	10.71	8.87	4.40	7.04	8.73	9.08	6.52	7.42	16.19	7.02	7.33	7.09	10.01	8.10	12.49	7.61	6.25	6.27	8.55	4.65	9.68	6.51
Endothelial_cells	LIFR	15.53	9.66	13.79	9.56	9.58	8.95	14.25	12.87	6.53	8.24	20.01	16.94	13.01	7.70	13.86	11.71	12.31	11.87	14.55	13.62	11.84	11.87	13.71	12.71	13.08	9.28	9.26	9.43	12.36	11.12	14.00	14.59	11.93	13.47	12.12	10.96	11.16	10.93	10.36	13.72	13.89
Endothelial_cells	PECAM1	7.25	5.85	6.89	7.31	4.68	4.72	7.23	5.90	2.66	5.14	11.64	10.99	7.97	7.22	12.15	9.92	10.65	8.46	8.79	5.41	6.27	7.18	6.68	5.69	5.71	4.89	6.78	5.89	6.58	5.47	6.38	6.96	11.50	9.22	8.80	8.15	9.86	7.01	4.58	6.75	6.52
Endothelial_cells	VWF	2.77	1.01	1.04	1.62	1.72	1.49	1.91	1.21	2.73	1.13	1.66	1.89	2.39	1.36	6.46	2.56	1.76	1.86	1.67	1.72	1.81	1.90	1.30	1.70	1.18	1.08	1.99	0.64	1.53	1.63	1.68	2.32	1.75	1.55	5.58	1.85	1.92	1.03	1.21	1.31	1.69
Endothelial_cells	CD34	0.72	0.29	0.39	0.51	0.43	0.36	0.30	0.21	0.53	0.37	0.56	0.34	0.53	0.53	0.82	0.61	0.68	0.46	0.41	0.42	0.28	0.49	0.29	0.30	0.31	0.21	0.51	0.26	0.38	0.60	0.50	0.49	0.44	0.30	1.45	0.36	0.60	0.38	0.27	0.45	0.35
Endothelial_cells	TM4SF1	2.85	2.25	2.05	2.13	1.78	1.28	2.13	1.88	1.23	0.90	3.93	2.21	1.99	2.07	2.66	2.52	3.16	2.22	2.24	1.91	2.02	2.35	2.24	2.12	2.31	1.43	2.28	2.43	2.03	2.38	2.67	2.27	2.24	2.77	4.05	2.27	2.45	2.13	1.74	2.15	2.08
Endothelial_cells	CLEC14A	3.57	2.49	2.86	3.83	3.09	3.41	3.00	1.60	3.12	3.46	6.16	3.83	3.84	3.41	3.85	4.17	3.26	2.86	3.44	2.54	2.39	2.74	3.62	3.01	2.82	2.47	3.67	3.10	2.38	3.14	3.35	3.84	3.85	3.69	5.62	3.12	3.92	3.75	1.57	4.21	2.78
Endothelial_cells	CCL14	3.34	2.84	3.21	2.21	2.28	2.33	3.71	3.22	2.85	4.84	2.43	2.30	3.42	2.16	2.55	3.04	2.63	3.52	1.92	5.29	2.83	2.31	3.36	4.68	2.79	2.20	2.50	2.53	3.35	3.06	3.33	3.57	2.94	1.87	3.80	4.24	1.73	3.02	4.16	3.54	2.68
Endothelial_cells	FLT1	3.76	3.55	2.91	3.01	3.22	1.62	4.16	2.45	1.18	1.94	3.55	3.14	2.69	2.38	2.96	2.77	2.51	3.10	3.69	2.55	3.31	2.84	2.81	2.89	2.83	3.52	3.24	1.80	3.42	3.50	3.70	3.31	2.98	2.21	3.70	2.81	3.11	2.93	2.85	3.56	3.13
Endothelial_cells	DNASE1L3	27.58	28.84	27.70	24.43	24.63	15.43	19.65	26.98	8.68	18.41	23.14	25.97	28.06	21.15	26.20	23.15	27.19	28.76	26.27	29.93	29.60	21.75	27.70	26.57	27.49	20.84	25.64	30.49	27.84	29.09	24.20	21.55	23.41	31.44	21.73	25.89	22.21	26.69	26.74	27.04	25.47
Endothelial_cells	FCN2	45.10	46.80	56.51	58.82	64.29	23.42	44.05	41.09	11.81	76.62	63.00	63.21	28.40	26.85	24.57	37.73	45.59	32.95	46.95	62.62	62.06	50.54	56.31	51.63	64.01	18.68	40.96	36.38	32.21	59.22	32.81	40.80	48.53	37.36	32.36	35.12	48.01	40.17	36.25	42.75	31.15
Endothelial_cells	CLEC4M	3.12	2.45	4.37	3.16	3.53	3.87	1.66	2.31	1.31	5.91	2.17	3.59	3.12	2.26	3.27	2.13	2.87	3.20	1.98	3.57	2.06	3.47	3.14	2.48	2.41	2.73	2.02	2.98	2.05	2.50	1.79	2.55	3.98	3.98	2.97	3.13	1.28	2.61	2.72	2.71	1.62
Endothelial_cells	CLEC1B	3.08	3.97	3.18	3.48	3.15	0.99	1.21	3.56	1.19	3.20	2.26	2.83	2.41	2.48	1.53	2.93	3.00	3.01	4.06	3.41	3.68	3.59	2.95	2.79	3.64	2.83	3.57	3.16	3.11	3.57	2.49	2.01	2.45	2.81	2.82	3.01	3.06	3.00	2.88	3.57	2.92
Endothelial_cells	CLEC4G	16.29	16.07	20.98	15.55	16.66	7.83	8.79	16.81	8.31	14.65	16.72	17.17	20.83	12.63	19.45	14.43	21.76	18.26	17.79	18.50	16.78	14.00	17.55	20.25	17.83	12.40	14.97	24.80	15.97	16.60	15.79	14.34	18.29	20.50	14.79	16.54	11.96	18.03	13.76	16.75	12.88
Erythroid	CA1	0.00	0.00	0.00	0.00	0.00	0.00	0.02	0.00	0.00	0.00	0.00	0.00	0.00	0.00	0.00	0.00	0.00	0.00	0.02	0.01	0.01	0.02	0.00	0.00	0.00	0.01	0.02	0.00	0.00	0.00	0.01	0.00	0.00	0.00	0.00	0.00	0.00	0.00	0.04	0.00	0.00
Erythroid	HBB	11.83	21.99	13.12	16.08	17.83	13.31	3.50	12.22	8.18	11.66	3.89	8.88	3.69	6.38	5.36	2.52	13.63	5.73	5.96	15.02	16.93	5.37	7.29	18.99	11.30	13.14	7.73	49.62	6.90	11.42	4.71	5.03	4.48	13.42	9.47	5.50	3.72	10.23	14.16	7.64	5.61
Erythroid	ALAS2	0.08	0.10	0.10	0.17	0.18	0.06	0.11	0.11	0.02	0.07	0.03	0.03	0.07	0.06	0.00	0.00	0.03	0.04	0.03	0.12	0.08	0.04	0.00	0.16	0.15	0.08	0.08	0.14	0.03	0.02	0.04	0.05	0.01	0.15	0.02	0.04	0.04	0.12	0.23	0.03	0.05
Hepatocytes	BCHE	25.84	31.72	46.75	27.82	26.46	27.80	25.89	32.22	21.57	33.55	9.75	17.18	17.63	18.09	11.37	20.42	20.80	30.29	28.68	34.61	43.63	34.55	20.35	46.66	29.47	29.39	32.77	24.36	23.60	36.50	33.11	26.90	25.12	18.69	28.52	25.25	23.88	21.62	35.06	28.94	19.70
Hepatocytes	PCK1	13.11	9.17	26.33	27.01	22.83	7.91	206.63	12.93	12.58	19.64	8.39	20.05	25.59	20.59	9.41	14.42	4.39	68.11	21.77	14.12	20.13	14.08	9.48	36.55	15.04	23.19	4.71	16.09	15.38	14.13	19.17	45.16	11.12	10.11	23.68	21.78	7.14	9.86	7.45	11.55	6.61
Hepatocytes	GSTA2	524.26	274.07	154.19	602.48	176.58	144.30	113.08	413.64	184.13	104.75	216.10	140.88	21.79	529.82	51.77	78.50	306.84	55.45	521.67	5.68	159.85	165.66	281.27	182.81	100.71	226.16	45.61	339.13	337.37	225.42	166.31	98.94	41.21	107.55	81.13	1.65	112.34	151.33	49.62	182.56	337.35
Hepatocytes	CYP3A4	405.39	363.27	701.99	582.23	551.75	307.39	1142.92	507.82	417.38	258.92	518.42	531.79	270.29	547.92	318.29	282.08	515.41	687.48	467.35	846.63	517.87	590.25	669.84	582.87	608.69	521.47	409.16	608.82	1498.07	750.54	947.06	494.50	386.33	696.33	346.65	427.38	495.53	338.37	332.78	365.14	540.22
Hepatocytes	G6PC	48.54	41.13	98.26	85.70	69.09	92.45	316.29	51.98	37.95	84.22	28.28	45.87	40.94	44.31	22.33	41.22	21.21	166.52	26.99	60.15	50.73	35.49	42.46	34.62	32.70	60.62	44.29	91.38	22.04	50.89	52.10	92.22	42.12	49.50	117.13	42.11	36.62	29.94	33.68	61.80	41.03
Hepatocytes	SEC16B	16.56	12.20	12.80	12.02	13.08	10.29	10.95	16.47	11.89	6.67	13.89	13.60	13.35	9.48	11.31	7.98	11.83	11.16	13.90	10.39	10.30	14.17	9.94	10.23	8.56	14.15	8.71	9.62	16.14	7.92	10.80	12.54	15.10	15.32	9.63	13.78	7.69	18.00	13.60	10.58	13.28
Hepatocytes	ALDH6A1	92.32	77.89	92.72	96.84	91.49	87.82	105.89	98.30	57.66	95.45	61.75	82.17	68.16	98.67	60.68	97.81	67.99	85.24	77.85	89.41	102.47	96.56	85.45	92.15	101.69	85.84	79.26	67.33	53.59	87.51	96.30	79.33	76.59	105.68	66.63	60.50	67.14	66.68	80.20	72.47	80.85
Hepatocytes	APOB	1405.25	1549.12	1557.98	1353.88	1518.86	1456.09	1488.73	1529.76	855.61	1743.02	1367.84	1429.11	1367.13	1533.24	1282.32	1548.30	953.94	1422.46	1375.41	1468.51	1566.33	1457.64	1514.93	1478.74	1483.22	1672.08	1576.54	929.36	1561.85	1600.01	1704.48	1535.15	1352.42	1118.12	1495.70	1490.34	1675.03	1642.70	1751.47	1627.77	1405.94
Hepatocytes	HSD11B1	86.71	99.60	100.73	109.65	95.61	97.77	91.02	100.09	42.34	79.82	39.62	92.84	81.88	95.99	73.23	88.25	78.13	79.78	92.67	83.93	119.42	94.18	86.35	96.67	106.97	90.05	84.50	135.84	112.69	93.84	95.70	98.42	87.50	101.94	72.80	87.91	96.59	103.40	105.52	85.18	76.63
Hepatocytes	HPR	47.85	45.88	39.70	56.01	56.14	50.70	52.07	27.76	24.79	37.98	25.41	45.06	38.00	42.58	23.90	44.35	36.24	44.74	41.23	61.81	60.83	43.58	54.88	42.80	52.18	43.66	23.86	68.06	55.12	52.24	44.76	37.50	33.38	55.95	30.09	34.56	30.72	37.94	30.96	37.45	205.03
Hepatocytes	CYP2E1	392.30	441.28	464.61	493.76	501.13	402.30	444.98	643.34	401.45	449.31	424.72	571.58	616.83	566.80	373.62	539.28	557.05	499.96	540.09	386.66	534.11	553.43	552.55	534.39	482.09	557.92	411.39	475.36	508.73	595.32	657.98	524.88	497.91	902.35	412.92	558.53	557.48	776.59	618.17	481.58	515.74
Hepatocytes	TF	858.90	850.67	978.16	996.13	866.30	905.55	929.18	963.17	726.05	673.50	1013.83	980.66	816.22	869.05	865.97	957.26	944.42	927.22	908.35	858.74	964.97	978.13	953.56	946.62	1020.45	871.79	1087.30	788.32	831.97	822.75	827.90	893.28	853.98	832.16	959.89	781.67	894.66	881.24	793.83	1072.63	1049.13
Hepatocytes	HAMP	15.47	13.43	27.01	5.13	9.14	8.99	0.14	15.73	21.38	13.17	15.74	23.15	11.32	20.22	3.88	23.57	3.27	9.10	27.28	9.53	23.03	17.16	6.83	33.96	15.78	24.12	6.88	21.34	21.87	13.38	11.78	0.00	19.44	9.86	14.57	18.51	3.97	28.05	21.71	11.79	12.29
Hepatocytes	MASP2	23.97	28.41	27.51	23.55	28.37	28.76	32.89	30.38	25.84	24.72	26.54	28.33	29.74	29.61	21.91	24.92	27.03	24.42	24.65	28.18	28.37	27.36	25.58	36.52	26.74	32.09	22.52	38.99	27.62	24.98	28.00	29.29	23.21	27.10	22.65	29.38	26.22	25.76	30.11	27.84	25.49
Hepatocytes	HP	549.92	819.45	778.81	861.54	674.58	658.98	766.73	548.03	301.71	395.43	448.61	597.91	431.99	911.26	403.14	454.16	281.91	646.31	528.71	599.59	883.56	617.16	604.38	922.21	587.67	695.27	612.05	979.90	694.25	682.49	729.95	563.47	732.14	689.06	607.82	576.17	571.62	697.23	420.01	581.06	403.04
Hepatocytes	ASGR1	24.30	30.41	23.13	24.30	25.04	20.17	25.94	29.70	39.55	34.29	21.55	24.07	27.71	23.68	24.98	23.77	31.02	26.62	23.99	27.66	22.96	25.27	24.73	26.88	26.36	27.69	23.71	31.92	25.19	26.14	22.74	26.64	23.88	31.46	20.98	27.61	24.68	26.65	26.24	23.00	26.95
Hepatocytes	GHR	55.99	71.58	50.76	49.73	53.57	56.47	30.57	50.65	34.42	25.36	33.20	32.71	23.49	33.50	29.49	30.94	28.88	49.94	46.33	41.79	36.42	44.09	47.60	41.71	38.65	51.57	69.98	20.80	34.77	56.96	40.57	40.68	23.30	20.34	44.66	50.08	59.13	49.42	47.99	64.20	38.74
Hepatocytes	ASS1	245.45	310.40	496.67	168.62	403.92	284.52	370.21	295.96	186.51	231.85	252.11	296.33	434.91	466.99	143.92	455.53	247.74	359.37	282.61	337.55	406.75	354.86	355.89	339.53	439.87	328.17	234.90	466.69	258.01	237.51	455.67	227.85	336.19	463.35	248.93	238.38	384.67	169.34	273.73	184.25	232.85
Hepatocytes	APOE	499.62	709.67	755.27	444.40	614.12	618.00	597.73	630.87	608.93	351.05	572.23	598.18	455.89	812.08	466.01	463.06	695.45	580.94	544.84	515.67	561.71	588.43	618.03	601.72	596.10	492.08	640.55	1032.03	437.78	601.40	592.09	749.71	555.66	649.28	538.65	580.71	474.72	515.22	476.64	535.04	511.18
Hepatocytes	SCD	359.86	494.30	423.69	207.90	361.97	538.64	229.36	349.77	137.06	229.32	376.62	174.42	677.46	272.61	421.21	272.39	368.27	174.84	285.94	97.06	285.27	135.83	273.64	166.58	206.43	289.45	746.56	256.45	264.79	592.48	61.72	284.36	309.14	160.01	184.72	226.71	623.85	316.33	933.16	318.54	396.25
Hepatocytes	CYP2A6	372.91	266.25	374.48	262.52	168.54	117.20	248.95	73.08	275.67	272.86	43.50	150.86	236.33	188.71	216.18	226.90	155.43	425.79	162.48	299.48	158.39	65.98	166.61	275.89	335.24	305.41	293.10	325.21	287.55	273.78	323.28	471.64	170.67	311.71	62.23	203.37	278.27	303.08	430.90	225.99	420.83
Hepatocytes	TM7SF2	5.68	10.53	10.24	5.95	10.48	4.72	7.53	7.96	7.19	12.25	7.02	6.69	7.83	7.21	6.55	5.70	7.06	5.81	7.16	6.66	6.85	7.39	8.23	8.00	6.38	6.89	8.01	11.35	8.72	10.52	5.79	5.87	7.15	8.73	5.52	5.01	9.14	6.89	9.99	8.81	9.10
Hepatocytes	CYP2A7	7.57	14.31	10.23	16.40	136.04	43.49	12.83	46.59	7.75	23.12	2.50	4.14	12.99	6.29	9.85	6.69	20.65	5.93	8.42	15.63	18.07	22.48	60.40	12.65	34.22	4.42	13.13	11.99	118.61	13.42	18.60	18.15	22.31	16.94	27.08	4.44	32.53	12.63	21.08	43.09	10.36
Hepatocytes	HMGCS1	28.75	40.85	85.55	94.99	52.18	19.65	43.83	35.92	38.68	16.88	36.76	44.82	77.93	28.50	84.61	29.93	23.92	29.21	103.01	39.90	77.82	27.08	45.78	85.15	49.25	19.74	32.96	25.60	42.53	37.34	12.89	30.15	53.05	10.24	59.14	37.46	62.11	22.04	39.59	32.95	112.27
Hepatocytes	CYP3A7	5.81	7.72	11.41	7.55	5.18	5.70	9.59	7.24	4.45	3.95	57.73	6.58	9.01	9.01	7.15	34.53	32.00	8.08	11.28	19.89	6.08	5.75	3.10	7.22	5.18	6.99	20.70	16.66	12.01	8.10	16.23	16.59	20.08	10.46	10.30	32.66	32.01	6.11	7.19	10.61	11.61
Hepatocytes	ACSS2	13.77	24.93	17.36	19.80	16.52	12.57	14.54	14.06	20.76	18.73	16.97	11.26	18.74	11.82	28.54	14.65	14.65	7.97	18.33	14.21	17.98	8.88	18.49	18.31	18.83	8.75	19.03	14.94	22.68	18.61	7.86	12.05	16.80	9.18	12.19	13.06	15.88	8.68	19.11	14.13	31.78

Abbreviations: FPKM, fragments per kilobase of transcript per million mapped reads; CHB, chronic hepatitis B; IT, immune tolerance; IA, immune active; IC, inactive carrier; ENH, HBeAg-negative hepatitis.

Table S3. mRNA expression levels (FPKM) of 110 immune-related genes in CHB patients across different phases.

Gene Group	ID	IT_1	IT_2	IT_3	IT_4	IT_5	IT_6	IT_7	IT_8	IT_9	IT_10	IA_1	IA_2	IA_3	IA_4	IA_5	IA_6	IA_7	IA_8	IA_9	IC_1	IC_2	IC_3	IC_4	IC_5	IC_6	IC_7	IC_8	IC_9	IC_10	IC_11	IC_12	IC_13	ENH_1	ENH_2	ENH_3	ENH_4	ENH_5	ENH_6	ENH_7	ENH_8	ENH_9
Im_regulators	TNFRSF14	4.16	3.59	3.83	3.59	2.85	3.47	3.00	3.51	6.13	5.32	5.65	5.25	4.83	4.78	5.29	5.07	4.66	3.86	3.93	4.10	2.91	3.18	3.48	3.42	3.54	3.71	3.31	4.14	3.08	3.63	3.30	4.15	4.50	4.12	3.83	5.20	3.68	3.89	3.60	4.14	3.08
Im_regulators	BTLA	0.45	0.14	0.20	0.35	0.28	0.66	0.36	0.27	0.64	0.18	0.67	0.81	0.46	0.57	0.44	1.04	0.76	0.81	0.18	0.18	0.23	0.38	0.42	0.26	0.41	0.21	0.33	0.14	0.13	0.16	0.16	0.24	0.80	0.74	0.36	0.54	0.86	0.28	0.19	0.39	0.37
Im_regulators	IDO1	0.39	0.21	0.23	0.22	0.13	0.10	0.23	0.11	0.58	0.23	0.49	0.57	0.33	0.33	0.58	0.53	0.66	0.41	0.25	0.21	0.06	0.25	0.19	0.14	0.27	0.09	0.19	0.33	0.06	0.14	0.08	0.11	0.37	0.43	0.57	0.24	0.24	0.15	0.08	0.19	0.21
Im_regulators	CD274	0.53	0.56	0.66	0.61	0.83	0.60	0.34	0.41	0.41	0.37	1.08	1.10	0.46	0.59	1.17	0.88	0.99	0.81	0.60	0.44	0.59	0.76	0.90	0.48	0.40	0.39	0.36	0.49	0.53	0.39	0.53	0.50	1.40	0.96	0.84	0.64	0.88	0.59	0.45	0.65	0.27
Im_regulators	EOMES	0.53	0.39	0.56	0.42	0.35	0.91	0.39	0.40	0.49	0.54	1.01	1.21	1.11	0.75	1.63	1.43	1.21	0.75	0.56	0.46	0.39	0.76	0.74	0.35	0.66	0.38	0.61	0.46	0.48	0.34	0.30	0.34	1.03	0.62	0.43	1.09	0.66	0.69	0.26	0.66	0.47
Im_regulators	CD48	2.46	2.12	2.64	2.88	1.22	3.50	2.57	2.07	1.54	1.37	5.05	5.85	2.53	3.42	5.66	5.65	5.88	4.08	2.82	2.28	1.78	2.66	1.92	1.60	2.39	1.29	3.19	3.32	1.64	2.07	1.76	1.90	5.85	5.07	2.52	4.06	3.47	2.10	1.34	2.51	2.26
Im_regulators	HAVCR2	1.23	1.02	2.27	1.90	1.08	1.64	1.33	1.08	0.41	0.94	2.86	3.14	1.24	2.21	3.52	2.30	3.31	2.19	1.78	1.30	1.40	1.80	1.01	1.52	1.54	1.10	1.31	1.98	0.92	1.09	1.21	1.30	3.16	2.11	1.43	1.84	1.78	1.45	1.17	1.47	1.39
Im_regulators	CD86	0.92	0.98	1.33	1.58	0.70	1.67	1.44	1.00	0.34	0.82	2.39	2.54	1.55	1.94	3.33	2.34	2.28	1.76	1.43	1.09	0.89	1.72	1.21	1.13	1.15	0.87	1.27	1.44	1.06	1.05	1.11	1.27	2.82	2.24	1.32	1.52	1.71	1.15	0.62	1.27	1.03
Im_regulators	LGALS3	1.11	1.13	1.85	1.04	0.87	1.55	1.43	1.04	0.23	0.71	3.89	2.67	0.70	1.44	1.03	1.83	3.33	1.76	1.61	1.09	0.87	1.02	1.00	0.84	1.05	0.58	1.29	1.75	0.71	0.68	0.78	0.71	3.40	2.94	1.58	1.65	1.93	1.16	0.59	1.05	1.07
Im_regulators	CD244	1.48	1.31	2.44	1.64	0.87	1.42	1.95	1.09	0.55	0.88	2.19	2.76	2.50	1.83	3.45	2.91	3.45	1.58	1.47	1.34	1.34	1.82	1.63	0.94	1.86	1.10	1.42	2.18	1.19	1.36	1.36	1.16	2.23	2.14	1.28	2.29	1.56	1.46	1.10	1.54	0.97
Im_regulators	TIGIT	0.37	0.06	0.39	0.24	0.06	0.36	0.34	0.07	0.40	0.14	0.64	1.03	0.46	0.19	1.00	0.95	1.03	0.79	0.29	0.29	0.15	0.40	0.53	0.18	0.31	0.05	0.31	0.20	0.20	0.10	0.13	0.33	0.90	0.59	0.49	0.79	0.35	0.53	0.07	0.38	0.11
Im_regulators	CTLA4	0.16	0.08	0.08	0.15	0.14	0.18	0.14	0.00	0.32	0.00	0.67	0.65	0.50	0.49	1.03	0.56	0.81	0.54	0.12	0.12	0.08	0.18	0.22	0.05	0.10	0.13	0.09	0.02	0.10	0.05	0.09	0.09	0.64	0.53	0.44	0.33	0.30	0.15	0.04	0.17	0.19
Im_regulators	LAG3	2.50	2.17	2.61	1.74	1.42	3.45	2.25	1.93	1.59	4.49	2.34	2.34	2.17	2.70	2.36	1.93	2.43	1.75	2.11	1.80	1.55	1.87	1.58	1.55	1.85	1.77	2.08	1.68	1.87	2.20	1.30	2.78	2.75	1.74	1.65	2.43	2.40	2.12	3.48	2.64	2.37
Im_regulators	LGALS9	1.51	1.52	1.99	1.91	0.94	2.12	1.85	1.30	0.72	1.67	3.36	2.81	1.35	1.95	3.51	2.19	2.73	2.13	1.77	1.22	0.85	1.47	1.33	1.03	1.19	0.84	1.16	1.74	1.08	1.06	1.09	1.31	2.71	2.71	1.52	2.05	1.90	1.14	1.24	1.64	1.11
Im_regulators	PDCD1LG2	3.04	2.80	3.64	4.04	1.74	2.40	3.11	2.45	1.20	2.35	5.10	4.93	3.70	3.19	5.91	4.94	6.09	3.47	3.26	3.33	2.95	4.03	3.78	2.93	3.39	2.66	2.72	4.57	2.89	2.96	2.47	2.94	6.08	5.07	3.08	3.16	3.93	2.58	1.77	2.73	2.24
Im_regulators	PDCD1	0.23	0.14	0.30	0.12	0.11	0.14	0.28	0.03	0.04	0.14	1.04	1.18	0.57	0.60	0.70	1.13	1.28	1.02	0.09	0.14	0.09	0.36	0.09	0.14	0.09	0.09	0.23	0.11	0.10	0.17	0.15	0.05	1.23	0.71	0.46	0.53	0.37	0.15	0.05	0.12	0.31
Chemokines receptors	XCR1	0.60	0.31	0.43	0.44	0.39	0.66	0.25	0.21	0.58	0.55	0.38	1.22	0.81	0.59	0.94	0.56	0.78	0.61	0.37	0.30	0.24	0.41	0.66	0.39	0.81	0.02	0.49	0.18	0.24	0.29	0.49	0.34	0.88	0.70	0.62	0.34	0.55	0.30	0.02	0.58	0.19
Chemokines receptors	CXCR3	0.21	0.11	0.13	0.21	0.10	0.31	0.46	0.15	0.60	0.19	1.09	0.85	0.86	0.55	1.10	1.03	1.04	0.82	0.41	0.24	0.19	0.29	0.39	0.12	0.28	0.20	0.24	0.46	0.13	0.19	0.25	0.26	0.88	0.71	0.36	0.59	0.83	0.28	0.18	0.25	0.39
Chemokines receptors	CXCR6	0.52	0.44	0.36	0.47	0.26	0.63	0.48	0.46	0.30	0.45	1.25	1.35	0.91	0.65	1.40	1.20	1.72	0.81	0.44	0.49	0.27	0.69	0.43	0.39	0.55	0.25	0.53	0.57	0.29	0.20	0.25	0.29	1.78	1.25	0.52	1.27	0.62	0.30	0.28	0.46	0.35
Chemokines receptors	CCR5	1.30	0.58	0.76	0.98	0.37	1.48	1.60	0.74	0.41	0.45	2.08	1.46	1.28	0.95	2.56	2.07	2.35	1.58	1.35	0.97	0.62	1.48	1.35	0.71	1.06	0.84	1.75	0.64	0.69	0.61	0.70	0.82	1.96	1.41	0.96	1.39	1.73	1.11	0.74	1.11	0.91
Chemokines receptors	CXCR4	0.81	0.59	0.58	0.77	0.62	1.90	1.10	0.49	0.45	0.34	3.15	2.66	1.45	2.23	2.99	2.49	1.77	2.41	1.76	0.89	0.99	0.98	0.69	0.66	0.53	0.60	0.84	0.66	1.09	0.63	0.67	0.79	2.32	2.60	1.17	1.29	2.33	1.04	0.41	0.67	0.93
Chemokines receptors	CCR2	0.59	0.38	0.22	0.59	0.26	0.59	0.83	0.32	0.40	0.36	1.30	0.61	0.50	0.66	0.70	0.92	0.69	0.90	0.60	0.43	0.56	0.48	0.42	0.32	0.36	0.19	0.80	0.29	0.27	0.39	0.43	0.52	1.04	0.59	0.78	0.54	1.47	0.49	0.25	0.52	0.42
Chemokines receptors	CCR6	0.14	0.01	0.03	0.16	0.16	0.03	0.01	0.13	0.00	0.01	0.40	0.00	0.01	0.03	0.11	0.02	0.20	0.07	0.12	0.02	0.08	0.13	0.00	0.02	0.01	0.01	0.16	0.01	0.00	0.04	0.00	0.01	0.14	0.12	0.11	0.07	0.15	0.01	0.11	0.09	0.01
Chemokines receptors	CCR7	0.38	0.19	0.17	0.26	0.25	0.21	0.37	0.19	1.06	0.47	0.64	0.65	0.54	0.63	0.56	0.74	0.20	1.02	0.39	0.08	0.19	0.43	0.23	0.15	0.20	0.16	0.24	0.13	0.14	0.14	0.19	0.14	0.41	0.33	0.31	0.43	0.61	0.16	0.07	0.26	0.22
Chemokines receptors	CCR1	4.88	5.94	8.88	6.75	3.53	7.48	5.79	5.69	0.84	6.87	9.62	8.77	3.98	7.20	10.94	7.02	8.57	7.70	6.68	4.67	5.77	6.06	5.00	5.03	4.71	4.24	4.84	7.68	4.92	3.61	4.23	4.55	10.60	8.66	5.17	6.14	7.15	5.62	4.13	5.25	3.56
Chemokines receptors	CX3CR1	1.18	0.80	1.18	1.21	0.66	1.51	1.28	1.00	0.75	0.32	1.49	1.58	0.89	0.64	1.60	0.89	0.71	1.36	1.06	0.86	1.10	0.98	1.18	0.46	0.95	0.48	1.37	0.84	0.73	1.04	1.37	0.93	1.19	1.01	0.76	1.30	1.27	0.90	0.95	1.42	0.91
Chemokines receptors	CXCR2	0.20	0.32	0.36	0.22	0.10	0.24	0.07	0.28	0.00	0.18	0.35	0.25	0.22	0.29	0.07	0.04	0.21	0.22	0.49	0.33	0.28	0.30	0.36	0.23	0.33	0.23	0.13	0.17	0.17	0.25	0.23	0.16	0.19	0.17	0.08	0.20	0.23	0.20	0.08	0.25	0.16
Chemokines receptors	CCR4	0.22	0.27	0.27	0.30	0.16	0.23	0.32	0.22	0.83	0.23	0.50	0.33	0.30	0.35	0.64	0.38	0.23	0.58	0.32	0.18	0.24	0.32	0.24	0.10	0.15	0.22	0.36	0.12	0.20	0.18	0.22	0.17	0.40	0.34	0.38	0.33	0.31	0.19	0.13	0.27	0.46
Chemokines receptors	CCR10	0.02	0.07	0.00	0.01	0.03	0.00	0.05	0.07	0.00	0.17	0.08	0.03	0.02	0.00	0.01	0.03	0.07	0.02	0.05	0.00	0.02	0.01	0.03	0.06	0.04	0.03	0.08	0.06	0.02	0.08	0.08	0.03	0.08	0.04	0.04	0.03	0.01	0.03	0.02	0.03	0.06
Chemokines receptors	CXCR1	0.79	0.65	0.30	0.19	0.14	0.54	0.49	0.51	0.00	0.67	0.87	0.71	0.64	0.72	0.17	0.31	0.28	0.60	0.84	0.40	0.65	0.66	0.64	0.77	0.44	0.51	0.53	0.33	0.32	0.32	0.50	0.47	0.49	0.49	0.25	0.08	0.47	0.17	0.71	0.40	0.60
Chemokines receptors	CCR3	0.02	0.03	0.02	0.01	0.02	0.02	0.01	0.03	0.00	0.05	0.03	0.04	0.01	0.03	0.10	0.02	0.04	0.01	0.02	0.01	0.02	0.01	0.05	0.01	0.03	0.02	0.00	0.02	0.01	0.03	0.01	0.01	0.02	0.00	0.00	0.05	0.02	0.01	0.01	0.04	0.03
Chemokines receptors	CCR8	0.00	0.02	0.06	0.02	0.00	0.04	0.07	0.04	0.00	0.00	0.21	0.24	0.20	0.02	0.03	0.04	0.06	0.06	0.02	0.00	0.00	0.03	0.00	0.07	0.02	0.00	0.05	0.01	0.00	0.03	0.03	0.09	0.03	0.09	0.00	0.11	0.00	0.02	0.00	0.00	0.00
Chemokines receptors	CCR9	0.02	0.04	0.00	0.01	0.05	0.01	0.01	0.05	0.00	0.01	0.01	0.01	0.01	0.00	0.00	0.06	0.02	0.01	0.01	0.02	0.01	0.03	0.04	0.04	0.04	0.02	0.03	0.00	0.04	0.02	0.04	0.02	0.01	0.07	0.01	0.02	0.03	0.02	0.00	0.04	0.02
Chemokines	CX3CL1	3.13	2.69	2.45	2.37	1.64	2.24	4.17	2.32	4.14	6.12	2.53	1.83	2.67	3.40	2.44	2.02	2.34	1.69	2.45	2.41	2.51	2.73	2.58	3.38	2.47	2.40	2.21	2.14	2.03	3.02	2.84	3.61	2.69	1.54	2.48	2.87	2.04	2.85	2.33	3.63	2.76
Chemokines	CXCL2	8.24	6.18	7.49	5.75	10.73	16.90	8.46	9.64	12.99	10.16	2.13	3.22	1.44	9.31	4.15	10.04	2.28	23.32	8.19	8.14	8.76	8.50	4.58	53.46	4.05	9.52	8.90	8.29	26.41	6.71	5.94	9.35	4.22	7.91	12.44	5.01	7.03	5.75	6.92	4.34	4.37
Chemokines	CXCL16	5.09	6.09	7.45	6.84	5.71	4.49	6.14	6.34	1.44	8.26	13.86	10.46	10.20	8.17	12.01	8.78	12.01	8.79	7.58	6.57	5.90	6.71	6.21	6.00	6.55	4.93	5.44	9.40	6.90	6.97	6.19	5.92	10.16	12.81	6.34	6.96	8.45	6.64	6.20	5.28	5.51
Chemokines	CCL4	0.96	1.39	0.93	0.86	0.63	1.62	1.00	0.95	0.45	0.86	2.68	2.61	1.51	2.75	4.39	2.42	4.15	1.86	1.09	0.60	0.90	1.07	0.71	0.77	1.29	0.57	1.08	1.90	0.45	0.91	0.53	0.58	3.45	2.54	1.40	2.41	1.29	1.66	0.46	1.15	0.71
Chemokines	CCL5	1.98	2.37	2.91	2.50	1.63	2.83	2.94	0.39	2.50	4.31	2.07	5.47	2.51	4.77	8.54	9.17	10.15	1.61	0.62	2.41	2.21	3.87	3.47	1.59	3.13	2.47	3.76	4.06	2.18	2.15	1.43	2.19	6.95	5.91	2.34	6.29	2.85	3.09	2.52	3.54	3.53
Chemokines	CXCL9	10.94	8.05	18.82	7.43	6.78	8.11	12.66	2.84	4.65	4.04	88.06	70.22	31.26	36.40	78.28	58.66	98.46	40.04	11.22	10.27	2.64	19.05	10.60	6.05	11.45	1.58	13.84	10.42	4.35	5.90	3.40	13.18	90.04	55.22	43.33	54.85	26.96	15.61	4.31	11.40	14.21
Chemokines	CXCL10	12.60	8.43	15.15	5.73	6.99	12.70	16.49	2.00	1.55	5.35	111.37	43.31	24.88	23.69	69.94	46.62	123.32	31.73	14.16	5.64	2.43	13.43	10.71	4.13	5.01	2.30	13.75	10.01	1.29	3.98	3.11	8.85	91.11	50.59	34.21	30.21	44.70	15.23	3.95	12.08	13.57
Chemokines	CXCL11	1.03	0.38	0.70	0.18	0.29	0.33	0.64	0.20	0.19	0.15	4.15	1.50	0.35	0.58	2.62	1.25	3.61	1.19	0.77	0.55	0.10	0.51	0.34	0.19	0.34	0.16	0.85	0.33	0.08	0.13	0.17	0.56	3.43	1.66	1.29	1.03	0.86	0.59	0.12	0.33	0.56
Chemokines	XCL1	0.43	0.34	0.55	0.43	0.52	0.66	0.84	0.32	0.20	0.45	1.33	1.05	0.47	0.55	1.13	0.82	1.84	0.86	0.28	0.30	0.30	0.47	0.69	0.41	0.62	0.11	0.50	1.68	0.25	0.20	0.88	0.39	0.91	0.89	0.45	0.98	0.97	0.63	0.80	0.53	0.79
Chemokines	CXCL13	0.13	0.09	0.14	0.18	0.13	0.50	0.43	0.12	0.00	0.17	1.31	1.33	0.77	1.15	1.38	1.12	1.15	1.17	0.13	0.13	0.31	0.44	0.19	0.06	0.13	0.24	0.20	0.59	2.49	0.04	0.07	0.33	1.63	0.44	0.46	1.07	0.64	0.23	0.39	0.17	0.58
Chemokines	CCL8	0.17	0.11	0.18	0.62	0.11	0.50	0.50	0.08	0.00	0.26	0.99	0.15	0.23	0.69	0.78	0.42	0.80	0.74	0.24	0.12	0.36	0.14	0.24	0.04	0.11	0.16	0.21	0.39	0.03	0.11	0.06	0.08	0.79	0.52	0.15	0.20	0.36	0.34	0.08	0.07	0.17
Chemokines	CCL18	0.21	0.32	1.00	0.40	0.59	1.32	1.24	0.19	0.66	0.05	1.32	2.18	1.04	1.30	1.82	2.36	1.19	4.26	0.59	0.33	0.36	0.86	0.64	0.30	0.23	0.10	0.49	0.48	0.06	0.20	0.43	0.62	0.85	1.87	0.70	0.91	0.85	0.52	0.18	0.21	0.46
Chemokines	CCL19	2.36	0.58	1.51	2.34	0.93	1.12	2.42	1.73	9.17	0.65	3.91	6.06	2.92	4.50	2.69	5.17	1.67	9.78	1.98	0.36	0.88	2.42	1.79	0.77	1.47	0.78	1.09	1.11	1.69	0.28	0.78	1.39	4.56	4.27	3.43	2.02	7.14	1.55	0.40	1.58	3.66
Chemokines	CCL21	7.30	2.16	4.77	3.50	1.92	5.01	6.84	1.66	14.71	3.60	22.60	8.45	12.06	11.02	8.60	20.39	7.94	11.89	5.09	1.60	3.17	8.58	4.70	2.74	2.11	1.51	3.25	4.37	6.89	1.26	2.18	4.79	9.88	8.97	11.92	8.11	13.52	3.46	0.98	4.24	6.46
Chemokines	CCL2	0.91	0.53	0.61	0.77	0.58	0.68	0.61	0.44	0.43	0.39	2.31	0.89	1.13	0.47	1.95	0.95	1.73	0.75	1.11	0.44	0.57	1.30	0.80	0.58	0.64	0.46	0.62	1.02	0.38	0.32	0.40	0.96	1.15	0.95	0.90	0.61	1.30	0.80	0.30	0.77	0.88
Chemokines	CCL20	0.08	0.05	0.08	0.02	0.00	0.00	0.10	0.03	0.33	0.00	0.68	0.15	0.00	0.14	0.17	0.07	0.26	0.08	0.11	0.00	0.04	0.12	0.00	0.04	0.01	0.05	0.17	0.00	0.01	0.06	0.03	0.00	0.14	0.13	0.00	0.13	0.29	0.09	0.02	0.03	0.03
Chemokines	CXCL6	0.10	0.10	0.39	0.25	0.21	0.17	0.60	0.07	0.13	0.02	1.70	1.11	0.57	0.39	0.32	0.44	0.64	0.33	1.12	0.48	0.17	0.50	0.28	0.15	0.08	0.31	0.20	0.16	0.38	0.10	0.25	0.24	0.56	0.26	0.46	0.21	0.85	0.18	0.04	0.27	0.37
Chemokines	CXCL8	0.13	0.16	0.08	0.07	0.00	0.25	0.22	0.12	0.00	0.00	1.76	0.57	0.57	0.38	0.28	0.27	0.29	0.15	0.58	0.07	0.07	0.12	0.12	0.05	0.06	0.03	0.13	0.02	0.18	0.05	0.08	0.06	0.51	0.34	0.22	0.22	0.49	0.09	0.09	0.16	0.15
Chemokines	CXCL1	0.10	0.21	0.39	0.22	0.41	0.06	0.45	0.00	0.26	0.11	0.93	0.45	0.42	0.37	0.36	0.38	0.40	0.18	0.91	0.07	0.13	0.23	0.23	0.16	0.16	0.13	0.10	0.11	0.33	0.13	0.24	0.21	0.51	0.52	0.40	0.21	0.59	0.59	0.03	0.14	0.32
Chemokines	CCL13	0.11	0.20	0.00	0.00	0.08	0.00	0.00	0.10	0.00	0.00	0.15	0.10	0.06	0.06	0.33	0.10	0.09	0.10	0.18	0.00	0.13	0.25	0.27	0.00	0.00	0.11	0.06	0.11	0.00	0.15	0.00	0.00	0.04	0.00	0.13	0.00	0.09	0.17	0.34	0.00	0.23
Chemokines	CXCL5	0.00	0.04	0.00	0.00	0.00	0.07	0.05	0.04	0.00	0.00	0.17	0.09	0.00	0.02	0.00	0.04	0.00	0.02	0.08	0.02	0.03	0.00	0.08	0.00	0.01	0.00	0.12	0.03	0.05	0.01	0.07	0.06	0.08	0.04	0.10	0.00	0.08	0.02	0.00	0.00	0.11
Chemokines	CXCL3	0.05	0.02	0.08	0.07	0.07	0.09	0.04	0.05	0.02	0.09	0.13	0.09	0.03	0.21	0.19	0.00	0.13	0.07	0.07	0.05	0.05	0.01	0.04	0.00	0.04	0.01	0.05	0.00	0.02	0.06	0.03	0.00	0.06	0.03	0.05	0.07	0.04	0.07	0.05	0.03	0.04
Chemokines	CXCL17	0.06	0.00	0.00	0.00	0.00	0.10	0.00	0.00	0.00	0.00	0.00	0.00	0.00	0.00	0.13	0.00	0.00	0.00	0.00	0.00	0.04	0.03	0.00	0.00	0.00	0.00	0.00	0.00	0.05	0.00	0.00	0.00	0.00	0.00	0.00	0.00	0.00	0.00	0.00	0.00	0.04
Chemokines	CCL17	0.00	0.12	0.00	0.00	0.00	0.00	0.00	0.13	0.00	0.00	0.06	0.00	0.00	0.00	0.00	0.00	0.00	0.00	0.00	0.00	0.00	0.00	0.07	0.13	0.00	0.00	0.14	0.00	0.17	0.05	0.00	0.00	0.00	0.00	0.00	0.00	0.11	0.00	0.00	0.00	0.00
Chemokines	CCL1	0.00	0.00	0.00	0.00	0.00	0.00	0.00	0.15	0.00	0.00	0.08	0.00	0.10	0.00	0.00	0.00	0.00	0.08	0.00	0.00	0.07	0.00	0.00	0.00	0.00	0.00	0.00	0.00	0.00	0.00	0.00	0.00	0.12	0.00	0.00	0.00	0.00	0.00	0.00	0.00	0.00
Chemokines	CCL16	7.12	15.08	15.36	9.66	7.69	14.06	11.04	12.36	15.43	11.88	14.21	12.07	15.09	13.53	14.44	9.09	14.20	10.25	10.57	15.33	9.35	12.32	15.24	18.63	8.83	13.67	8.12	12.60	6.66	7.71	13.20	10.40	12.73	15.84	12.63	8.88	10.92	8.64	14.29	11.14	11.26
Chemokines	CXCL14	7.29	11.42	9.16	9.82	7.33	7.90	11.15	8.70	4.02	5.69	4.90	12.43	10.74	12.78	12.35	10.83	13.06	7.82	10.67	8.02	5.77	8.98	14.48	7.56	11.13	8.39	8.99	13.03	5.81	8.50	6.22	6.79	9.03	4.58	11.30	11.15	10.22	9.38	9.50	11.09	7.54
Chemokines	CXCL12	22.31	17.40	24.08	23.47	21.95	18.67	34.47	20.84	17.11	22.76	55.15	25.09	28.58	24.51	29.82	24.66	37.48	26.24	26.80	28.77	22.20	25.16	26.85	17.22	25.13	15.92	26.32	24.20	22.31	24.15	26.99	33.58	26.73	27.51	30.61	22.16	32.12	24.69	21.91	24.22	23.94
Complements	C1QC	84.08	85.28	171.35	152.06	67.12	133.99	137.20	92.23	8.58	179.71	265.10	243.80	92.37	182.77	224.68	185.91	364.08	148.57	147.43	87.58	63.31	130.94	117.32	83.33	106.19	62.52	71.13	212.37	98.74	99.14	81.08	100.63	287.59	271.79	98.18	162.90	150.75	103.55	60.75	115.19	77.04
Complements	C1QB	14.22	22.10	36.62	25.69	14.35	32.71	29.85	16.33	1.40	39.28	47.06	51.93	21.06	39.17	56.71	45.61	107.43	33.87	30.26	18.31	21.06	27.45	19.26	21.07	21.89	12.21	17.95	56.85	22.33	21.35	22.87	21.23	54.59	68.70	20.79	31.41	45.69	22.93	15.75	22.59	15.12
Complements	C1QA	44.80	51.04	92.01	77.08	37.16	53.37	74.51	52.99	5.18	161.36	144.60	123.21	30.30	104.75	89.12	89.05	198.45	82.35	64.03	49.96	44.76	76.45	61.30	52.67	58.25	36.10	43.39	144.04	49.22	49.03	50.93	49.56	144.88	145.69	46.45	59.35	69.04	48.57	36.15	44.59	38.76
Inflammasomes	NLRC3	1.09	0.51	0.54	0.68	0.66	0.74	0.75	0.75	1.02	0.69	1.76	1.50	0.73	1.12	1.55	1.81	0.87	1.14	0.89	0.69	0.59	0.92	1.03	0.63	0.81	0.69	1.11	0.48	0.63	0.59	0.58	0.68	1.34	0.82	0.75	1.45	0.96	0.89	0.64	0.84	0.93
Inflammasomes	NLRC5	3.04	1.82	1.96	2.12	1.37	2.26	1.90	1.39	4.31	2.04	5.19	4.72	4.53	3.48	6.89	4.92	3.12	3.03	2.18	1.60	1.35	2.69	2.78	1.24	1.66	1.33	2.04	1.14	1.09	1.05	1.13	1.63	4.66	2.14	2.50	3.96	2.11	2.20	1.62	2.17	2.22
Inflammasomes	NLRC4	0.61	0.38	1.07	0.57	0.56	0.86	0.73	0.47	0.48	0.35	1.34	1.55	0.66	0.80	1.34	1.02	1.17	0.93	1.12	0.44	0.63	0.62	0.77	0.26	0.53	0.43	0.66	0.68	0.66	0.51	0.38	0.42	1.08	1.04	0.75	1.13	0.95	0.46	0.51	0.74	0.49
Inflammasomes	NLRP3	0.39	0.22	0.30	0.34	0.17	0.26	0.48	0.34	0.20	0.20	0.52	0.48	0.18	0.38	0.54	0.29	0.32	0.34	0.32	0.24	0.30	0.25	0.19	0.19	0.25	0.16	0.32	0.30	0.25	0.20	0.31	0.34	0.38	0.52	0.34	0.22	0.34	0.24	0.26	0.25	0.29
Inflammasomes	NLRP11	0.25	0.11	0.04	0.07	0.23	0.18	0.26	0.22	0.23	0.26	0.12	0.43	0.25	0.05	0.20	0.06	0.24	0.27	0.41	0.20	0.29	0.18	0.05	0.11	0.29	0.09	0.17	0.21	0.17	0.31	0.11	0.42	0.22	0.17	0.24	0.20	0.15	0.37	0.20	0.23	0.15
Inflammasomes	NLRP5	0.00	0.00	0.00	0.00	0.00	0.00	0.00	0.00	0.00	0.00	0.01	0.00	0.00	0.00	0.00	0.00	0.00	0.02	0.01	0.00	0.01	0.00	0.00	0.00	0.00	0.01	0.00	0.00	0.01	0.00	0.00	0.00	0.00	0.00	0.00	0.00	0.00	0.00	0.00	0.00	0.00
Inflammasomes	NLRP4	0.00	0.00	0.00	0.01	0.00	0.00	0.00	0.01	0.00	0.00	0.01	0.00	0.00	0.01	0.00	0.00	0.00	0.00	0.00	0.00	0.00	0.00	0.00	0.00	0.00	0.00	0.01	0.00	0.00	0.00	0.01	0.00	0.01	0.00	0.00	0.00	0.00	0.02	0.00	0.00	0.00
Inflammasomes	NLRP12	0.06	0.06	0.11	0.05	0.09	0.11	0.04	0.06	0.09	0.15	0.14	0.24	0.02	0.07	0.12	0.09	0.04	0.09	0.16	0.10	0.11	0.05	0.13	0.07	0.04	0.08	0.09	0.05	0.05	0.06	0.07	0.14	0.14	0.15	0.03	0.04	0.12	0.04	0.13	0.09	0.06
Inflammasomes	NLRP1	1.22	0.83	0.92	0.96	0.83	0.96	1.04	0.75	1.79	1.21	1.84	1.59	1.46	1.25	1.38	1.57	0.80	1.11	1.04	0.85	0.79	0.93	1.09	0.85	0.97	0.71	1.01	0.54	0.91	0.73	0.92	0.99	1.25	1.00	1.29	1.56	1.09	0.91	0.77	1.10	1.09
Inflammasomes	NLRP8	0.00	0.00	0.00	0.00	0.00	0.00	0.00	0.00	0.00	0.00	0.03	0.00	0.00	0.00	0.00	0.00	0.00	0.02	0.02	0.00	0.00	0.00	0.00	0.00	0.01	0.02	0.00	0.00	0.02	0.00	0.00	0.00	0.00	0.00	0.00	0.00	0.00	0.00	0.01	0.00	0.00
Inflammasomes	NLRP6	0.75	1.77	0.74	0.83	0.86	3.03	0.67	1.25	2.18	1.39	0.37	0.73	1.03	0.49	0.47	0.14	0.30	1.09	0.53	1.40	1.08	0.31	0.91	0.64	0.60	1.52	2.22	0.93	0.45	0.65	0.68	1.49	0.79	0.71	2.45	0.93	0.92	1.76	1.70	0.68	0.66
Inflammasomes	NLRP14	0.76	0.31	0.37	0.40	0.61	1.04	0.30	0.39	0.35	0.37	0.32	0.32	0.26	0.43	0.27	1.17	0.18	0.56	0.37	0.40	0.24	0.29	0.29	0.33	0.36	0.39	0.25	0.33	0.33	0.56	0.48	0.34	0.28	0.66	0.24	0.40	0.32	0.12	0.94	0.45	0.32
Inflammasomes	NLRP13	0.00	0.00	0.00	0.00	0.00	0.00	0.00	0.00	0.00	0.00	0.01	0.00	0.00	0.00	0.00	0.00	0.00	0.01	0.02	0.00	0.01	0.00	0.00	0.00	0.00	0.01	0.00	0.00	0.00	0.00	0.01	0.00	0.00	0.00	0.00	0.00	0.00	0.00	0.00	0.00	0.00
Inflammasomes	NLRP7	0.01	0.00	0.02	0.00	0.00	0.01	0.01	0.00	0.00	0.00	0.04	0.01	0.01	0.02	0.00	0.03	0.01	0.01	0.00	0.02	0.01	0.00	0.01	0.01	0.02	0.01	0.01	0.01	0.03	0.00	0.01	0.02	0.02	0.02	0.02	0.03	0.04	0.01	0.04	0.00	0.00
Inflammasomes	NLRP2	0.05	0.00	0.04	0.04	0.00	0.02	0.20	0.05	0.14	0.07	0.18	0.05	0.07	0.06	0.07	0.05	0.09	0.06	0.17	0.09	0.04	0.17	0.10	0.03	0.02	0.10	0.06	0.03	0.04	0.07	0.09	0.07	0.10	0.08	0.14	0.11	0.10	0.08	0.05	0.08	0.02
Cytokines	IL23A	0.03	0.07	0.00	0.17	0.07	0.32	0.21	0.00	0.00	0.03	0.05	0.12	0.06	0.06	0.06	0.05	0.07	0.07	0.24	0.03	0.11	0.06	0.05	0.10	0.15	0.13	0.16	0.05	0.07	0.05	0.17	0.14	0.09	0.08	0.06	0.07	0.13	0.08	0.05	0.05	0.11
Cytokines	IL17C	0.00	0.00	0.00	0.02	0.00	0.00	0.00	0.00	0.00	0.00	0.00	0.00	0.00	0.00	0.00	0.00	0.08	0.05	0.00	0.00	0.00	0.00	0.00	0.00	0.02	0.02	0.03	0.00	0.04	0.02	0.00	0.00	0.03	0.00	0.00	0.00	0.02	0.00	0.00	0.00	0.00
Cytokines	IL10	0.08	0.15	0.27	0.27	0.17	0.18	0.09	0.27	0.00	0.00	0.32	0.17	0.12	0.14	0.39	0.06	0.42	0.21	0.22	0.19	0.72	0.09	0.04	0.06	0.16	0.35	0.08	0.34	0.30	0.08	0.09	0.08	0.16	0.06	0.07	0.18	0.10	0.15	0.12	0.16	0.09
Cytokines	IL12A	0.04	0.07	0.05	0.02	0.03	0.02	0.01	0.03	0.00	0.00	0.08	0.04	0.01	0.02	0.07	0.05	0.07	0.06	0.03	0.00	0.00	0.04	0.02	0.01	0.04	0.01	0.01	0.01	0.04	0.01	0.02	0.02	0.06	0.04	0.07	0.03	0.02	0.01	0.03	0.01	0.02
Cytokines	TGFB2	0.46	0.14	0.20	0.20	0.17	0.06	0.39	0.09	0.32	0.12	0.82	0.43	0.13	0.38	0.70	0.41	0.40	0.13	0.58	0.18	0.10	0.29	0.32	0.18	0.26	0.05	0.23	0.05	0.22	0.19	0.31	0.23	0.12	0.36	0.56	0.26	0.50	0.18	0.16	0.23	0.26
Cytokines	TNF	0.35	1.02	1.17	0.67	0.25	0.97	0.41	0.61	1.29	0.66	1.58	1.27	0.74	1.03	2.62	1.02	1.21	1.15	0.84	0.59	0.37	0.56	0.42	0.60	0.51	0.35	0.69	0.80	0.43	0.49	0.29	0.79	1.48	1.39	0.85	0.69	0.67	0.99	0.40	0.78	0.34
Cytokines	IL7	0.50	0.48	0.49	0.35	0.38	0.71	0.32	0.44	0.33	0.21	0.60	0.40	0.46	0.35	0.27	0.56	0.43	0.42	0.39	0.47	0.34	0.29	0.38	0.38	0.29	0.45	0.32	0.25	0.33	0.52	0.31	0.45	0.31	0.47	0.63	0.36	0.49	0.46	0.55	0.34	0.37
Cytokines	IL15	0.28	0.15	0.41	0.37	0.21	0.25	0.53	0.16	0.35	0.09	0.71	0.63	0.15	0.47	0.68	0.33	0.39	0.42	0.23	0.44	0.36	0.18	0.19	0.27	0.11	0.23	0.19	0.22	0.20	0.31	0.38	0.20	0.66	0.36	0.31	0.20	0.45	0.15	0.24	0.28	0.18
Cytokines	IL32	1.37	2.13	3.69	1.70	1.64	1.41	2.01	1.08	1.17	0.86	13.86	8.09	5.83	5.98	7.77	3.74	12.73	5.42	6.06	1.58	1.03	2.52	2.93	1.87	1.52	2.22	1.89	4.34	2.21	2.42	0.96	1.47	8.99	8.59	4.99	5.55	7.04	4.85	2.64	2.88	2.39
Cytokines	IL18	0.69	0.95	0.82	0.94	0.61	1.01	0.82	0.74	0.21	0.38	2.73	2.41	0.95	1.29	2.58	1.80	1.90	1.30	1.11	1.16	0.62	0.91	1.04	0.92	0.93	0.51	1.06	0.87	0.83	0.90	0.83	0.91	2.47	1.68	1.28	1.67	1.38	1.31	0.61	1.22	0.72
Cytokines	IL16	0.82	0.76	1.00	0.79	0.72	0.85	1.00	0.61	1.28	0.83	1.71	1.05	1.44	1.29	1.15	1.81	1.42	0.94	0.94	0.99	0.73	1.08	1.20	0.85	0.91	0.72	0.88	0.80	0.74	0.67	0.84	0.95	1.53	1.05	1.41	1.37	0.88	1.02	0.76	0.90	0.99
Cytokines	IL1B	0.31	0.20	0.42	0.35	0.14	0.40	0.29	0.24	0.13	0.32	0.92	0.67	0.33	0.54	1.16	0.32	0.92	0.41	0.38	0.33	0.64	0.30	0.27	0.19	0.11	0.18	0.38	0.45	0.55	0.20	0.21	0.34	0.82	0.53	0.32	0.46	0.40	0.30	0.18	0.44	0.25
Cytokines	CSF1	3.10	3.26	3.31	3.37	2.81	3.34	2.76	2.83	3.39	3.37	6.69	6.11	5.14	3.87	6.73	5.18	5.77	4.69	4.45	3.11	3.04	4.00	3.51	2.85	3.10	2.85	3.87	2.86	2.75	3.66	2.88	4.10	5.32	3.85	3.87	4.57	3.79	4.24	2.52	3.59	2.76
Cytokines	IL13	0.00	0.00	0.00	0.00	0.01	0.00	0.00	0.00	0.00	0.00	0.00	0.00	0.00	0.00	0.00	0.01	0.01	0.00	0.01	0.00	0.00	0.00	0.03	0.00	0.00	0.00	0.00	0.00	0.00	0.00	0.00	0.00	0.03	0.00	0.02	0.00	0.00	0.02	0.00	0.00	0.00
Cytokines	IL36G	0.02	0.02	0.00	0.04	0.03	0.04	0.03	0.02	0.00	0.00	0.00	0.02	0.03	0.08	0.00	0.00	0.00	0.04	0.02	0.02	0.02	0.03	0.13	0.09	0.05	0.07	0.00	0.03	0.17	0.00	0.03	0.00	0.09	0.00	0.00	0.00	0.04	0.00	0.02	0.00	0.06
Cytokines	CSF3	0.00	0.00	0.00	0.01	0.00	0.00	0.00	0.00	0.00	0.00	0.00	0.00	0.00	0.00	0.01	0.00	0.01	0.00	0.00	0.00	0.00	0.00	0.00	0.00	0.01	0.00	0.00	0.00	0.00	0.02	0.00	0.00	0.00	0.00	0.00	0.00	0.00	0.00	0.00	0.00	0.00
Cytokines	IL2	0.00	0.00	0.00	0.00	0.10	0.00	0.00	0.00	0.18	0.00	0.00	0.03	0.00	0.00	0.00	0.03	0.00	0.00	0.00	0.00	0.00	0.00	0.00	0.00	0.00	0.00	0.00	0.00	0.00	0.00	0.00	0.00	0.02	0.00	0.00	0.00	0.06	0.00	0.00	0.02	0.00
Cytokines	IL34	0.64	0.16	0.42	0.36	0.34	0.24	0.61	0.53	0.57	0.48	1.07	0.46	0.63	0.32	0.60	0.70	0.36	0.53	0.50	0.23	0.34	0.43	0.35	0.43	0.57	0.30	0.46	0.59	0.34	0.26	0.31	0.59	0.46	0.63	0.43	0.55	0.47	0.38	0.12	0.40	0.36
Cytokines	IL27	4.22	2.53	5.87	3.19	8.48	5.61	5.03	4.63	3.68	11.02	2.18	3.51	2.38	4.28	2.68	4.12	1.79	6.24	4.28	4.57	3.73	3.19	2.73	3.96	6.04	7.73	2.62	4.60	7.21	4.31	5.15	6.50	2.83	3.45	3.39	4.33	2.44	2.32	5.33	3.37	5.01
Cytokines	IL21	0.09	0.00	0.00	0.00	0.00	0.00	0.00	0.00	0.00	0.00	0.08	0.16	0.15	0.10	0.22	0.04	0.46	0.04	0.00	0.00	0.00	0.05	0.00	0.00	0.03	0.00	0.00	0.03	0.00	0.00	0.00	0.05	0.25	0.00	0.00	0.06	0.15	0.09	0.00	0.03	0.00
Cytokines	IL1A	0.00	0.00	0.00	0.00	0.00	0.00	0.03	0.00	0.00	0.00	0.05	0.07	0.00	0.00	0.03	0.00	0.04	0.00	0.00	0.00	0.00	0.00	0.00	0.00	0.02	0.00	0.05	0.00	0.00	0.00	0.02	0.00	0.00	0.00	0.00	0.00	0.00	0.00	0.00	0.03	0.00
Cytokines	IL12B	0.00	0.00	0.03	0.04	0.00	0.00	0.06	0.00	0.29	0.00	0.07	0.02	0.04	0.02	0.02	0.02	0.02	0.12	0.03	0.00	0.00	0.00	0.02	0.00	0.00	0.05	0.02	0.00	0.03	0.00	0.04	0.00	0.01	0.06	0.05	0.02	0.03	0.08	0.02	0.05	0.00
Cytokines	IL5	0.03	0.00	0.00	0.00	0.00	0.05	0.06	0.03	0.00	0.00	0.03	0.02	0.03	0.03	0.00	0.00	0.00	0.02	0.00	0.03	0.02	0.03	0.03	0.08	0.02	0.03	0.03	0.02	0.02	0.02	0.02	0.00	0.04	0.03	0.00	0.03	0.02	0.00	0.03	0.03	0.04
Cytokines	IL20	0.00	0.00	0.00	0.00	0.00	0.03	0.00	0.00	0.00	0.00	0.00	0.00	0.00	0.00	0.00	0.00	0.00	0.00	0.00	0.00	0.01	0.00	0.00	0.00	0.01	0.00	0.00	0.00	0.00	0.00	0.00	0.00	0.00	0.00	0.00	0.00	0.00	0.00	0.00	0.00	0.00
Cytokines	CSF2	0.00	0.00	0.00	0.00	0.00	0.00	0.00	0.00	0.00	0.00	0.00	0.00	0.00	0.15	0.00	0.00	0.00	0.06	0.00	0.07	0.00	0.00	0.00	0.00	0.05	0.06	0.00	0.13	0.00	0.00	0.10	0.00	0.05	0.13	0.00	0.08	0.00	0.00	0.00	0.00	0.00
Cytokines	IL11	0.00	0.00	0.00	0.00	0.00	0.00	0.00	0.00	0.00	0.00	0.03	0.01	0.00	0.00	0.00	0.00	0.00	0.01	0.00	0.03	0.00	0.00	0.00	0.06	0.02	0.00	0.00	0.00	0.00	0.00	0.00	0.00	0.01	0.00	0.00	0.00	0.03	0.00	0.00	0.00	0.00
Cytokines	IL6	0.01	0.00	0.00	0.00	0.00	0.00	0.00	0.00	0.00	0.00	0.00	0.00	0.00	0.02	0.03	0.02	0.00	0.00	0.00	0.00	0.02	0.00	0.00	0.02	0.01	0.01	0.00	0.00	0.00	0.01	0.00	0.00	0.00	0.02	0.00	0.00	0.01	0.02	0.00	0.00	0.00
Cytokines	IL17D	0.00	0.03	0.04	0.03	0.52	0.00	0.02	0.00	0.00	0.03	0.07	0.13	0.03	0.06	0.60	0.01	0.33	0.06	0.02	0.04	0.04	0.05	0.03	0.01	0.01	0.00	0.00	0.02	0.02	0.35	0.05	0.00	0.06	0.04	0.12	0.09	0.01	0.03	0.01	0.00	0.14
Cytokines	IL17B	0.00	0.00	0.00	0.00	0.00	0.00	0.00	0.00	0.00	0.00	0.00	0.00	0.00	0.00	0.00	0.00	0.00	0.00	0.00	0.00	0.00	0.00	0.00	0.00	0.05	0.00	0.00	0.00	0.00	0.00	0.00	0.00	0.02	0.00	0.04	0.00	0.00	0.00	0.00	0.00	0.00
Cytokines	IL4	0.04	0.00	0.06	0.00	0.00	0.00	0.00	0.00	0.00	0.00	0.00	0.00	0.00	0.05	0.00	0.00	0.00	0.04	0.00	0.00	0.00	0.00	0.00	0.00	0.00	0.00	0.00	0.00	0.03	0.00	0.03	0.00	0.03	0.00	0.00	0.00	0.00	0.00	0.00	0.02	0.06
Cytokines	IL33	6.94	6.18	5.90	5.27	5.72	3.47	4.94	5.83	1.82	3.97	8.38	8.22	4.18	4.83	4.80	4.75	6.12	6.02	6.50	4.88	5.98	6.89	6.78	6.11	5.74	4.42	5.75	5.33	6.04	5.40	6.86	6.99	6.59	7.34	6.38	6.34	6.10	5.16	5.45	6.39	5.82

Abbreviations: FPKM, fragments per kilobase of transcript per million mapped reads; CHB, chronic hepatitis B; IT, immune tolerance; IA, immune active; IC, inactive carrier; ENH, HBeAg-negative hepatitis.

Table S4. Medium of normalized expression values of 559 hepatic miRNAs across CHB clinical phases.

Systematic name	Medium of normalized signal value				IT phase	IA phase	IC phase	ENH phase
	IT phase	IA phase	IC phase	ENH phase	(n = 491)	(n = 544)	(n = 453)	(n = 544)
hsa-miR-7975	16.9	17	17	17	p	p	p	P
hsa-miR-122-5p	15.6	15.3	15.6	15.3	p	p	p	P
hsa-miR-7977	15.3	15.6	15.3	15.6	p	p	p	P
hsa-miR-8069	14.2	13.7	14.1	13.8	p	p	p	P
hsa-let-7a-5p	12.8	12.9	12.9	12.9	p	p	p	P
hsa-miR-451a	12.7	12.1	12.1	12	p	p	p	P
hsa-miR-5100	12.3	11.9	12.3	12.1	p	p	p	P
hsa-miR-21-5p	12.2	12.9	12.3	12.6	p	p	p	P
hsa-let-7f-5p	12.2	12.2	12.1	12.3	p	p	p	P
hsa-miR-192-5p	11.9	11.8	12	11.9	p	p	p	P
hsa-let-7b-5p	11.8	11.9	11.9	11.9	p	p	p	P
hsa-miR-148a-3p	11.5	11.2	11.7	11.4	p	p	p	P
hsa-miR-4284	11.5	11.3	11.4	11.4	p	p	p	P
hsa-miR-122-3p	11.2	10.9	11.3	10.9	p	p	p	P
hsa-let-7g-5p	11.2	11.3	11.2	11.3	p	p	p	P
hsa-miR-7641	11	11.6	11.1	11.3	p	p	p	P
hsa-miR-16-5p	11	11.3	10.9	11.1	p	p	p	P
hsa-miR-29a-3p	11	11.2	11.1	11.2	p	p	p	P
hsa-miR-29c-3p	11	11	11	11.1	p	p	p	P
hsa-miR-26b-5p	11	10.9	11	11	p	p	p	P
hsa-miR-126-3p	10.9	10.9	10.8	10.8	p	p	p	P
hsa-miR-194-5p	10.9	10.7	11	10.7	p	p	p	P
hsa-let-7c-5p	10.9	10.9	10.9	10.9	p	p	p	P
hsa-miR-215-5p	10.9	10.7	10.9	10.8	p	p	p	P
hsa-miR-1260a	10.9	10.6	10.9	10.8	p	p	p	P
hsa-miR-26a-5p	10.9	10.8	11.1	10.9	p	p	p	P
hsa-miR-125b-5p	10.8	10.6	10.9	10.7	p	p	p	P
hsa-miR-22-3p	10.7	10.7	10.6	10.7	p	p	p	P
hsa-miR-1273g-3p	10.7	10.8	10.7	10.6	p	p	p	P
hsa-miR-19b-3p	10.7	10.6	10.9	10.8	p	p	p	P
hsa-miR-30b-5p	10.7	10.6	10.9	10.6	p	p	p	P
hsa-miR-494-3p	10.7	10.9	10.5	10.8	p	p	p	P
hsa-miR-23b-3p	10.7	10.4	10.9	10.6	p	p	p	P
hsa-miR-27b-3p	10.6	10.5	10.7	10.6	p	p	p	P
hsa-miR-20a-5p	10.5	10.4	10.6	10.4	p	p	p	P
hsa-miR-4286	10.2	9.8	10	10	p	p	p	P
hsa-miR-24-3p	10.1	10.2	10.2	10.2	p	p	p	P
hsa-miR-107	9.9	9.9	9.9	9.9	p	p	p	P
hsa-miR-99a-5p	9.9	9.7	9.9	9.8	p	p	p	P
hsa-miR-103a-3p	9.9	10	10	10	p	p	p	P
hsa-miR-4459	9.8	9.2	9.9	9.3	p	p	p	P
hsa-miR-199a-3p	9.8	10.1	10.2	10.4	p	p	p	P
hsa-miR-6085	9.7	9.4	9.6	9.4	p	p	p	P
hsa-miR-4516	9.6	9.2	9.6	9.6	p	p	p	P
hsa-miR-5739	9.6	9.2	9.4	9.5	p	p	p	P
hsa-miR-23a-3p	9.6	10	9.7	9.9	p	p	p	P
hsa-let-7i-5p	9.5	9.7	9.4	9.5	p	p	p	P
hsa-miR-3960	9.4	9.2	9.4	9.5	p	p	p	P
hsa-miR-6089	9.4	8.8	9.3	9.1	p	p	p	P
hsa-miR-4485-5p	9.4	9.2	9.2	9	p	p	p	P
hsa-miR-142-3p	9.3	10.3	9.6	9.8	p	p	p	P
hsa-miR-100-5p	9.3	9.5	9.5	9.5	p	p	p	P
hsa-miR-1260b	9.3	9.1	9.3	9.2	p	p	p	P
hsa-miR-101-3p	9.2	8.8	9.3	9	p	p	p	P
hsa-miR-365a-3p	9.2	8.9	9.2	9.1	p	p	p	P
hsa-miR-30e-5p	9.1	8.8	9.3	9	p	p	p	P
hsa-let-7d-5p	9.1	9.1	9.1	9	p	p	p	P
hsa-miR-15a-5p	9.1	9.2	9.3	9.1	p	p	p	P
hsa-miR-29b-3p	9.1	9.3	9.1	9.1	p	p	p	P
hsa-miR-15b-5p	9.1	9.4	9.2	9.1	p	p	p	P
hsa-miR-30c-5p	9	8.9	9.3	9	p	p	p	P
hsa-miR-4299	9	8.9	9.2	8.9	p	p	p	P
hsa-miR-19a-3p	9	8.7	9.1	8.9	p	p	p	P
hsa-miR-17-5p	9	8.8	9.2	8.9	p	p	p	P
hsa-miR-92a-3p	9	8.7	9.1	8.7	p	p	p	P
hsa-miR-195-5p	9	9.2	9.3	9.4	p	p	p	P
hsa-miR-885-5p	8.9	8.5	9.1	8.7	p	p	p	P
hsa-miR-130a-3p	8.8	9.1	9	8.9	p	p	p	P

hsa-miR-27a-3p	8.8	9.1	8.8	9	p	p	p	P
hsa-miR-30a-5p	8.8	8.8	8.9	8.8	p	p	p	P
hsa-miR-6749-5p	8.7	8.3	8.6	8.6	p	p	p	P
hsa-miR-378i	8.6	8.4	8.8	8.6	p	p	p	P
hsa-miR-34a-5p	8.6	9.7	8.8	9.1	p	p	p	P
hsa-miR-223-3p	8.5	8.9	8.5	8.7	p	p	p	P
hsa-miR-6087	8.4	8.1	8.2	8.3	p	p	p	P
hsa-miR-424-5p	8.4	8.7	8.5	8.6	p	p	p	P
hsa-miR-378a-3p	8.4	8.1	8.7	8.3	p	p	p	P
hsa-miR-6127	8.4	8.3	8.6	8.2	p	p	p	P
hsa-miR-151a-5p	8.3	8.2	8.3	8.1	p	p	p	P
hsa-miR-145-5p	8.3	8.4	8.8	8.6	p	p	p	P
hsa-miR-199a-5p	8.2	8.6	8.7	8.9	p	p	p	P
hsa-miR-30d-5p	8.2	8	8.5	8.1	p	p	p	P
hsa-miR-6090	8.2	7.7	8.1	8.1	p	p	p	P
hsa-miR-20b-5p	8.2	8	8.2	8.1	p	p	p	P
hsa-miR-455-3p	8.1	7.5	8.4	7.7	p	p	p	P
hsa-miR-6826-5p	8.1	7.7	7.9	7.8	p	p	p	P
hsa-miR-106b-5p	8	8.3	8.2	8.1	p	p	p	P
hsa-miR-3162-5p	8	7.8	8	7.9	p	p	p	P
hsa-miR-25-3p	8	8.3	8.2	8.2	p	p	p	P
hsa-miR-4281	8	7.2	8.1	7.5	p	p	p	P
hsa-miR-374a-5p	8	8.1	8.1	8.1	p	p	p	P
hsa-miR-6875-5p	7.9	8	8	8	p	p	p	P
hsa-miR-193b-3p	7.9	7.8	7.7	7.8	p	p	p	P
hsa-miR-150-5p	7.8	8.8	7.9	8.6	p	p	p	P
hsa-let-7e-5p	7.8	8.2	8.1	8.1	p	p	p	P
hsa-miR-6869-5p	7.8	7.4	7.4	7.6	p	p	p	P
hsa-miR-3653-3p	7.7	7.3	7.9	7.5	p	p	p	P
hsa-miR-6165	7.6	7.3	7.3	7.3	p	p	p	P
hsa-miR-342-3p	7.6	8	7.7	7.8	p	p	p	P
hsa-miR-324-3p	7.6	7.7	7.5	7.7	p	p	p	P
hsa-miR-193a-3p	7.5	7.2	7.4	7.1	p	p	p	P
hsa-miR-4763-3p	7.4	6.6	7.3	6.8	p	p	p	P
hsa-miR-10a-5p	7.3	7.6	7.6	7.8	p	p	p	P
hsa-miR-197-5p	7.3	7.5	7.6	7.4	p	p	p	P
hsa-miR-6125	7.3	7.1	7	7.2	p	p	p	P
hsa-miR-497-5p	7.3	7.6	7.6	7.8	p	p	p	P
hsa-miR-4270	7.2	5.6	7.4	6.5	p	p	p	P
hsa-miR-642a-3p	7.2	7.2	7.1	7.3	p	p	p	P
hsa-miR-331-3p	7.2	7.3	7.2	7.3	p	p	p	P
hsa-miR-146b-5p	7.2	8.1	7.2	7.8	p	p	p	P
hsa-miR-1202	7.2	6.9	7	7.2	p	p	p	P
hsa-miR-6088	7.1	6.2	7	6.6	p	p	p	P
hsa-miR-181a-5p	7.1	7.8	7.4	7.5	p	p	p	P
hsa-miR-144-3p	7.1	6.4	7	6.9	p	p	p	P
hsa-miR-7114-5p	7	6.9	7.1	7	p	p	p	P
hsa-miR-1225-5p	6.9	6.6	7.1	6.6	p	p	p	P
hsa-miR-93-5p	6.9	7.4	7.1	7.1	p	p	p	P
hsa-miR-6821-5p	6.9	6.7	6.8	6.9	p	p	p	P
hsa-miR-1207-5p	6.8	6	6.5	6.2	p	p	p	P
hsa-miR-374b-5p	6.8	7.1	6.8	7	p	p	p	P
hsa-miR-574-3p	6.6	6.5	6.8	6.6	p	p	p	P
hsa-miR-361-5p	6.6	6.5	6.8	6.6	p	p	p	P
hsa-miR-28-5p	6.6	6.6	6.6	6.7	p	p	p	P
hsa-miR-30e-3p	6.6	6.6	6.7	6.6	p	p	p	P
hsa-miR-1973	6.5	6.8	6.1	6.6	p	p	p	P
hsa-miR-340-5p	6.5	6.3	6.5	6.6	p	p	p	P
hsa-miR-574-5p	6.5	6.5	6.4	6.7	p	p	p	P
hsa-miR-6879-5p	6.5	6.6	6.4	6.6	p	p	p	P
hsa-miR-4530	6.5	6.9	6.5	6.7	p	p	p	P
hsa-miR-4485-3p	6.4	6.6	6	6.5	p	p	p	P
hsa-miR-320b	6.4	6.5	6.6	6.5	p	p	p	P
hsa-miR-146a-5p	6.4	7.3	6.5	7	p	p	p	P
hsa-miR-214-3p	6.3	7	6.8	7	p	p	p	P
hsa-miR-151b	6.3	6.4	6.4	6.5	p	p	p	P
hsa-miR-140-3p	6.3	6.7	6.5	6.6	p	p	p	P
hsa-miR-320d	6.3	6.5	6.5	6.6	p	p	p	P
hsa-miR-376c-3p	6.3	6.4	6	6.3	p	p	p	P
hsa-miR-455-5p	6.2	5.9	6	6	p	p	p	P
hsa-miR-425-5p	6.2	6.5	6.2	6.4	p	p	p	P
hsa-miR-142-5p	6.2	7.3	6.3	6.9	p	p	p	P

hsa-miR-6800-5p	6.1	6.3	6.1	6.3	p	p	p	P
hsa-miR-6893-5p	6.1	6.2	6.1	6.1	p	p	p	P
hsa-miR-320a	6.1	6.2	6.3	6.2	p	p	p	P
hsa-miR-2861	6	5.4	5.7	5.7	p	p	p	P
hsa-miR-185-5p	6	6.2	5.8	6.2	p	p	p	P
hsa-miR-3651	6	6.3	6.1	6.3	p	p	p	P
hsa-miR-1915-3p	6	5.7	6	6.1	p	p	p	P
hsa-miR-638	6	5.6	5.9	5.8	p	p	p	P
hsa-miR-376a-3p	6	6.2	5.8	6.2	p	p	p	P
hsa-miR-210-3p	6	5.6	6	5.6	p	p	p	P
hsa-miR-6803-5p	5.9	6	6.1	6	p	p	p	P
hsa-miR-6132	5.9	5.9	5.9	5.8	p	p	p	P
hsa-miR-320e	5.9	6	5.9	6.1	p	p	p	P
hsa-miR-200b-3p	5.9	6.6	6.6	7.2	p	p	p	P
hsa-miR-505-3p	5.9	5.7	5.9	6	p	p	p	P
hsa-miR-940	5.9	5.8	5.9	5.9	p	p	p	P
hsa-miR-3665	5.9	5.9	5.8	6.1	p	p	p	P
hsa-miR-4721	5.9	5.9	5.9	5.8	p	p	p	P
hsa-miR-6740-5p	5.8	6.1	5.8	6	p	p	p	P
hsa-miR-126-5p	5.8	5.7	5.5	5.8	p	p	p	P
hsa-miR-6785-5p	5.8	6.1	6.1	6.1	p	p	p	P
hsa-miR-6780b-5p	5.7	5.9	5.6	5.8	p	p	p	P
hsa-miR-4465	5.7	6.2	5.8	5.9	p	p	p	P
hsa-miR-30a-3p	5.7	6	6.1	5.9	p	p	p	P
hsa-miR-125a-5p	5.7	6.3	6	6.2	p	p	p	P
hsa-miR-3195	5.6	5.7	5.5	5.8	p	p	p	P
hsa-miR-6763-5p	5.6	5.3	5.3	5.5	p	p	p	P
hsa-miR-423-5p	5.6	5.7	5.6	5.8	p	p	p	P
hsa-miR-5006-5p	5.6	5.4	5.3	5.5	p	p	p	P
hsa-miR-4713-3p	5.6	5.9	5.6	5.7	p	p	p	P
hsa-miR-3196	5.6	5.4	5.2	5.6	p	p	p	P
hsa-miR-660-5p	5.6	6	5.5	5.8	p	p	p	P
hsa-miR-939-5p	5.6	5.3	5.3	5.6	p	p	p	P
hsa-miR-1268a	5.5	4.7	5.4	4.9	p	p	p	P
hsa-miR-4291	5.5	5.6	5.5	5.7	p	p	p	P
hsa-miR-98-5p	5.5	5.8	5.4	5.8	p	p	p	P
hsa-miR-7704	5.5	5.6	5.4	5.6	p	p	p	P
hsa-miR-6124	5.5	5.3	5.4	5.4	p	p	p	P
hsa-miR-4653-3p	5.5	5.6	5.6	5.5	p	p	p	P
hsa-miR-5787	5.5	5.2	4.9	5.2	p	p	p	P
hsa-miR-4507	5.4	5.5	5.4	5.5	p	p	p	P
hsa-miR-4672	5.4	5.5	5.3	5.5	p	p	p	P
hsa-miR-324-5p	5.4	5.5	5.1	5.3	p	p	p	P
hsa-miR-4466	5.4	5.5	5.3	5.6	p	p	p	P
hsa-miR-148b-3p	5.3	5.6	5.2	5.5	p	p	p	P
hsa-miR-4428	5.3	5.6	5.3	5.4	p	p	p	P
hsa-miR-320c	5.3	5.5	5.4	5.5	p	p	p	P
hsa-miR-4505	5.3	5.3	5.3	5.4	p	p	p	P
hsa-miR-874-3p	5.3	5.3	5.2	5.3	p	p	p	P
hsa-miR-211-3p	5.3	4.9	4.7	5.1	p	p	p	P
hsa-miR-486-5p	5.3	5	5.3	5.2	p	p	p	P
hsa-miR-483-5p	5.3	5.2	5	5.4	p	p	p	P
hsa-miR-4665-3p	5.2	5	5.2	5.1	p	p	p	P
hsa-miR-762	5.2	5.4	5.2	5.4	p	p	p	P
hsa-miR-6131	5.2	5.5	5.2	5.3	p	p	p	P
hsa-miR-186-5p	5.2	5.5	5	5.5	p	p	p	P
hsa-miR-4687-3p	5.2	5.4	5	5.6	p	p	p	P
hsa-miR-4728-5p	5.2	5.4	5.1	5.4	p	p	p	P
hsa-miR-152-3p	5.2	5.2	5.1	5.3	p	p	p	P
hsa-miR-151a-3p	5.2	5.5	5.3	5.5	p	p	p	P
hsa-miR-483-3p	5.2	4.9	5	5.1	p	p	p	P
hsa-miR-128-3p	5.1	5.8	4.8	5.4	p	p	p	P
hsa-miR-1228-3p	5.1	4.8	5	4.9	p	p	p	P
hsa-miR-590-5p	5.1	5.2	5.1	5.3	p	p	p	P
hsa-miR-130b-3p	5.1	5.6	5.3	5.5	p	p	p	P
hsa-miR-6717-5p	5.1	5.4	5.1	5.4	p	p	p	P
hsa-miR-937-5p	5.1	4.4	4.8	4.6	p	p	p	P
hsa-miR-3135b	5.1	5.2	4.7	5.4	p	p	p	P
hsa-miR-1249-3p	5.1	4.7	5.2	4.8	p	p	p	P
hsa-miR-8485	5.1	4.7	4.4	5	p	p	p	P
hsa-miR-4515	5	5	4.8	5.1	p	p	p	P
hsa-miR-143-3p	5	5.6	5.3	5.4	p	p	p	P

hsa-miR-4443	5	5.1	4.8	5.3	p	p	p	P
hsa-miR-29c-5p	5	4.9	4.8	5.1	p	p	p	P
hsa-miR-642b-3p	5	5	4.7	5	p	p	p	P
hsa-miR-4787-5p	5	4.9	4.7	5	p	p	p	P
hsa-miR-1275	5	4.9	4.9	4.9	p	p	p	P
hsa-miR-1246	5	4.9	4.5	4.9	p	p	p	P
hsa-miR-6068	5	4.3	4.6	4.7	p	p	p	P
hsa-miR-1268b	4.9	4.8	4.7	5	p	p	p	P
hsa-miR-204-5p	4.9	5.1	5.2	5.2	p	p	p	P
hsa-miR-378a-5p	4.9	4.8	5	5	p	p	p	P
hsa-miR-4442	4.9	5.1	4.7	5	p	p	p	P
hsa-miR-664a-3p	4.9	4.8	4.8	4.9	p	p	p	P
hsa-miR-1234-3p	4.9	4.7	5	4.8	p	p	p	P
hsa-miR-362-3p	4.9	5.2	4.8	5.2	p	p	p	P
hsa-miR-4313	4.9	4.7	4.9	4.9	p	p	p	P
hsa-miR-3198	4.9	5.3	5	5.2	p	p	p	P
hsa-miR-378d	4.9	4.7	4.9	5	p	p	p	P
hsa-miR-6812-5p	4.9	4.7	4.4	5.1	p	p	p	P
hsa-miR-6069	4.9	4.5	4.9	4.8	p	p	p	P
hsa-miR-664b-3p	4.8	4.8	4.6	4.8	p	p	p	P
hsa-miR-3656	4.8	4.5	4.6	4.8	p	p	p	P
hsa-miR-4261	4.8	4.7	4.4	4.7	p	p	p	P
hsa-miR-335-5p	4.8	5.4	5.2	5	p	p	p	P
hsa-miR-99b-5p	4.8	5.5	5	5.3	p	p	p	P
hsa-miR-200a-3p	4.8	5.4	5.1	6.1	p	p	p	P
hsa-miR-18a-5p	4.8	4.9	4.4	4.9	p	p	p	P
hsa-miR-3663-3p	4.8	4.6	4.5	4.7	p	p	p	P
hsa-miR-361-3p	4.7	5.2	4.5	4.9	p	p	p	P
hsa-miR-1238-3p	4.7	4.4	4.7	4.6	p	p	p	P
hsa-miR-4741	4.7	4.9	4.4	5.2	p	p	p	P
hsa-miR-7107-5p	4.7	4.6	4.2	4.8	p	p	p	P
hsa-miR-17-3p	4.7	5.1	5	4.9	p	p	p	P
hsa-miR-1305	4.7	5.1	4.7	5.1	p	p	p	P
hsa-miR-6800-3p	4.7	4.4	4.6	4.5	p	p	p	P
hsa-miR-4669	4.7	4.6	4.6	4.7	p	p	p	P
hsa-miR-6508-5p	4.7	4.4	4.6	4.5	p	p	p	P
hsa-miR-34b-5p	4.7	5.9	4.3	5.3	p	p	p	P
hsa-miR-4484	4.7	4.6	4.6	4.6	p	p	p	P
hsa-miR-193a-5p	4.7	4.6	4.9	4.8	p	p	p	P
hsa-miR-1304-3p	4.7	4.4	4.7	4.7	p	p	p	P
hsa-miR-3162-3p	4.6	4.5	4.6	4.5	p	p	p	P
hsa-miR-487b-3p	4.6	4.9	4.4	4.8	p	p	p	P
hsa-miR-6737-3p	4.6	4.4	4.7	4.4	p	p	p	P
hsa-miR-4649-3p	4.6	4.5	4.6	4.5	p	p	p	P
hsa-miR-4787-3p	4.6	4.4	4.5	4.6	p	p	p	P
hsa-miR-4739	4.6	5.3	4.6	5.1	p	p	p	P
hsa-miR-484	4.6	4.7	4.4	4.6	p	p	p	P
hsa-miR-6734-5p	4.6	5.1	4.6	4.9	p	p	p	P
hsa-miR-6889-3p	4.6	4.4	4.5	4.5	p	p	p	P
hsa-miR-6819-3p	4.6	4.3	4.6	4.4	p	p	p	P
hsa-miR-6515-3p	4.6	4.3	4.6	4.4	p	p	p	P
hsa-miR-6797-3p	4.6	4.4	4.6	4.5	p	p	p	P
hsa-miR-4478	4.6	4.8	4.5	4.7	p	p	p	P
hsa-miR-1224-5p	4.6	4.2	4.2	4.6	p	p	p	P
hsa-miR-4725-5p	4.6	4.3	4.4	4.4	p	p	p	P
hsa-miR-4497	4.5	4.6	4.6	4.7	p	p	p	P
hsa-miR-191-3p	4.5	4.3	4.5	4.4	p	p	p	P
hsa-miR-3607-3p	4.5	4.1	4.2	4.4	p	p	p	P
hsa-miR-6820-5p	4.5	4.5	4.5	4.6	p	p	p	P
hsa-miR-1281	4.5	4.2	4.5	4.4	p	p	p	P
hsa-miR-6724-5p	4.4	4.7	4.4	4.7	p	p	p	P
hsa-miR-6076	4.4	4.9	4.5	5.1	p	p	p	P
hsa-miR-1825	4.4	4.2	4.4	4.3	p	p	p	P
hsa-miR-7150	4.4	4.5	4.3	4.6	p	p	p	P
hsa-miR-6126	4.4	4.6	4.1	4.6	p	p	p	P
hsa-miR-95-3p	4.4	4.7	4.2	4.6	p	p	p	P
hsa-miR-377-3p	4.4	4.7	4.2	4.6	p	p	p	P
hsa-miR-4788	4.3	4.4	4.1	4.4	p	p	p	P
hsa-miR-8063	4.3	4.5	4.1	4.6	p	p	p	P
hsa-miR-22-5p	4.3	4.8	4.1	4.6	p	p	p	P
hsa-miR-6865-3p	4.3	4.1	4.3	4.3	p	p	p	P
hsa-miR-766-3p	4.3	4.3	4.3	4.3	p	p	p	P

hsa-miR-197-3p	4.3	4.5	4.4	4.5	p	p	p	P
hsa-miR-1539	4.3	4	4.2	4.1	p	p	p	P
hsa-miR-125a-3p	4.2	4.9	4.1	4.6	p	p	p	P
hsa-miR-338-3p	4.2	4.9	5.1	5.1	p	p	p	P
hsa-miR-4310	4.2	4.1	4.2	4.1	p	p	p	P
hsa-miR-7108-5p	4.2	4.1	3.9	4.2	p	p	p	P
hsa-miR-450a-5p	4.2	4.8	4.2	4.7	p	p	p	P
hsa-miR-425-3p	4.2	4	4.3	4.3	p	p	p	P
hsa-miR-2116-3p	4.2	4	4.1	4.1	p	p	p	P
hsa-miR-3679-5p	4.2	4.5	4	4.5	p	p	p	P
hsa-miR-1914-3p	4.2	4.5	4.3	4.4	p	p	p	P
hsa-miR-139-3p	4.2	4.1	4.1	4.1	p	p	p	P
hsa-miR-8072	4.1	4.1	4	4	p	p	p	P
hsa-miR-6723-5p	4.1	4.1	3.8	4.2	p	p	p	P
hsa-let-7b-3p	4.1	4	4.1	4.1	p	p	p	P
hsa-miR-4758-3p	4.1	4.1	4.1	4.1	p	p	p	P
hsa-miR-4746-3p	4.1	4.6	4.1	4.3	p	p	p	P
hsa-miR-6813-3p	4.1	3.9	4.2	4.1	p	p	p	P
hsa-let-7f-1-3p	4.1	3.9	4	4	p	p	p	P
hsa-miR-1587	4.1	4	3.8	3.9	p	p	p	P
hsa-miR-4436b-5p	4	3.9	3.9	4	p	p	p	P
hsa-miR-550a-5p	4	3.8	4	4	p	p	p	P
hsa-miR-181b-5p	4	5.1	3.9	4.6	p	p	p	P
hsa-miR-4664-3p	4	4	4.2	4.2	p	p	p	P
hsa-miR-203a-3p	4	4.2	4.1	4.4	p	p	p	P
hsa-miR-127-3p	4	4.4	3.8	4.3	p	p	p	P
hsa-miR-6891-5p	4	3.9	3.8	4.3	p	p	p	P
hsa-miR-3940-5p	4	3.8	4	4	p	p	p	P
hsa-miR-4433a-5p	4	3.8	3.9	3.9	p	p	p	P
hsa-miR-139-5p	3.9	4	4.1	4	p	p	p	P
hsa-miR-33b-3p	3.9	3.9	4	4	p	p	p	P
hsa-miR-32-5p	3.9	4.2	3.9	4	p	p	p	P
hsa-miR-193b-5p	3.9	3.9	3.9	4	p	p	p	P
hsa-miR-4749-3p	3.9	3.8	3.9	3.9	p	p	p	P
hsa-miR-7974	3.9	3.8	3.8	3.9	p	p	p	P
hsa-miR-6777-3p	3.9	3.6	3.8	3.8	p	p	p	P
hsa-miR-5001-5p	3.9	4	3.7	4.1	p	p	p	P
hsa-miR-6763-3p	3.8	3.8	3.9	3.9	p	p	p	P
hsa-miR-378g	3.8	3.8	3.7	4.1	p	p	p	P
hsa-miR-6851-3p	3.8	3.7	3.9	3.9	p	p	p	P
hsa-miR-532-3p	3.8	4.3	3.8	4.1	p	p	p	P
hsa-miR-296-5p	3.8	3.6	3.8	3.7	p	p	p	P
hsa-miR-654-3p	3.7	4	3.6	4	p	p	p	P
hsa-miR-362-5p	3.7	4.2	3.8	4.1	p	p	p	P
hsa-miR-221-3p	3.6	4.5	4	4.3	p	p	p	P
hsa-miR-6870-3p	3.5	3.3	3.5	3.4	p	p	p	P
hsa-miR-933	3.3	3.3	3.3	3.3	p	p	p	P
hsa-miR-4324	3.2	3.2	3.2	3.3	p	p	p	P
hsa-miR-140-5p	6.3	6.7	6.5	6.6	p	p	p	P
hsa-miR-4306	6.1	6.3	5.9	6.3	p	p	p	P
hsa-miR-155-5p	4.9	6.4	4.9	5.7	p	p	p	P
hsa-miR-10b-5p	4.9	5.3	4.8	5.3	p	p	p	P
hsa-miR-532-5p	4.8	5.3	4.8	5.1	p	p	p	P
hsa-miR-136-5p	4.2	4.5	4.1	4.4	p	p	p	P
hsa-miR-1181	4.1	4.6	3.9	4.5	p	p	p	P
hsa-miR-149-5p	4.1	4	4.1	4.1	p	p	p	P
hsa-miR-6752-3p	4	3.8	3.9	3.9	p	p	p	P
hsa-miR-6858-3p	3.9	3.8	4	3.9	p	p	p	P
hsa-miR-6812-3p	3.8	3.6	3.7	3.7	p	p	p	P
hsa-miR-125b-2-3p	3.8	3.9	3.6	3.9	p	p	p	P
hsa-miR-6752-5p	3.8	4	3.6	4	p	p	p	P
hsa-miR-23c	3.8	3.7	3.8	3.9	p	p	p	P
hsa-miR-20a-3p	3.7	3.8	3.7	3.9	p	p	p	P
hsa-miR-454-3p	3.5	4	3.5	3.7	p	p	p	P
hsa-miR-374c-5p	3.4	3.7	3.4	3.7	p	p	p	P
hsa-miR-18b-5p	3	3.1	2.9	3.1	p	p	p	P
hsa-miR-340-3p	4.1	4.1	4.1	4.2	p	p	p	P
hsa-miR-24-1-5p	3.7	3.6	3.6	3.8	p	p	p	P
hsa-miR-513a-5p	5.3	5.5	5.4	5.5	p	p	p	P
hsa-miR-564	4.4	4.4	4.2	4.3	p	p	p	P
hsa-miR-513b-5p	4.2	4.5	4.1	4.4	p	p	p	P
hsa-miR-4656	3.8	3.8	3.7	3.8	p	p	p	P

hsa-miR-6760-3p	3.8	3.6	3.9	3.7	p	p	p	P
hsa-miR-4430	4.5	4.8	4.6	4.6	p	p	p	P
hsa-miR-6766-3p	3.9	3.6	3.8	3.7	p	p	p	P
hsa-miR-575	4.5	4.7	4.1	4.7	p	p	p	P
hsa-miR-363-3p	4.2	4.9	4.1	4.5	p	p	p	P
hsa-miR-4716-3p	4.1	4.5	4	4.4	p	p	p	P
hsa-miR-5581-5p	4.1	4.5	4	4.5	p	p	p	P
hsa-miR-6767-5p	3.9	4.4	4	4.3	p	p	p	P
hsa-miR-381-3p	3.9	4.1	3.6	3.9	p	p	p	P
hsa-miR-619-5p	3.7	3.9	3.4	3.8	p	p	p	P
hsa-miR-6791-5p	4.6	4.5	4.4	4.4	p	p	p	P
hsa-miR-7110-5p	4.4	4.4	4.2	4.4	p	p	p	P
hsa-miR-3907	4	3.8	3.6	4	p	p	p	P
hsa-miR-542-3p	4	4.5	4	4.4	p	p	p	P
hsa-miR-4698	3.8	3.9	3.8	3.9	p	p	p	P
hsa-miR-1237-3p	3.8	3.6	3.8	3.8	p	p	p	P
hsa-miR-6848-3p	3.7	3.6	3.8	3.8	p	p	p	P
hsa-miR-301a-3p	3.6	4.2	3.4	3.9	p	p	p	P
hsa-miR-4323	3.2	3.2	3.3	3.3	p	p	p	P
hsa-miR-6880-3p	3.9	3.7	3.8	3.8	p	p	p	P
hsa-miR-6731-3p	3.7	3.6	3.7	3.8	p	p	p	P
hsa-miR-3180-5p	3.5	3.5	3.6	3.5	p	p	p	P
hsa-miR-188-5p	4.4	4	4.2	4.2	p	p	p	P
hsa-miR-144-5p	4	3.7	3.6	3.8	p	p	p	P
hsa-miR-4769-3p	3.6	3.5	3.6	3.7	p	p	p	P
hsa-miR-92b-3p	3.5	3.6	3.5	3.5	p	p	p	P
hsa-miR-129-2-3p	3.4	3.4	3.5	3.5	p	p	p	P
hsa-miR-513c-5p	3.3	3.6	3.3	3.5	p	p	p	P
hsa-miR-6795-3p	3.7	3.5	3.7	3.7	p	p	p	P
hsa-miR-4271	4.5	3.9	4.2	4.1	p	p	p	P
hsa-miR-6756-5p	4.4	4	4	4.1	p	p	p	P
hsa-miR-6858-5p	4.1	3.7	3.4	4	p	p	p	P
hsa-miR-4532	3.8	4.1	3.7	4	p	p	p	P
hsa-miR-6769b-5p	4.8	4.7	4.6	4.6	p	p	p	P
hsa-miR-6824-3p	3.8	3.6	3.8	3.8	p	p	p	P
hsa-miR-1908-3p	3.7	3.7	3.7	3.9	p	p	p	P
hsa-miR-6757-3p	3.8	3.5	3.7	3.7	p	p	p	P
hsa-miR-222-3p	3.2	3.8	3.2	3.6	p	p	p	P
hsa-miR-3646	3.7	3.9	3.6	3.9	p	p	p	P
hsa-miR-1-3p	3.6	4	4.3	4.3	p	p	p	P
hsa-miR-1229-5p	4.1	4.2	3.9	4.2	p	p	p	P
hsa-miR-602	3.7	3.6	3.5	3.7	p	p	p	P
hsa-miR-218-5p	3.7	4.7	3.7	4.5	p	p	p	P
hsa-miR-563	3.6	3.4	3.7	3.6	p	p	p	P
hsa-miR-145-3p	3.4	3.8	3.6	3.8	p	p	p	P
hsa-miR-214-5p	3.3	3.9	3.4	3.9	p	p	p	P
hsa-miR-5571-5p	3.7	3.5	3.6	3.7	p	p	p	P
hsa-miR-133b	3.5	3.5	3.7	3.7	p	p	p	P
hsa-miR-6785-3p	3.6	3.5	3.6	3.6	p	p	p	P
hsa-miR-1290	3.4	3.7	3.3	3.6	p	p	p	P
hsa-miR-3151-3p	3.6	3.8	3.7	3.8	p	p	p	P
hsa-miR-154-5p	3.5	3.8	3.4	3.7	p	p	p	P
hsa-miR-6861-3p	3.5	3.5	3.6	3.6	p	p	p	P
hsa-miR-5088-5p	3.5	4	3.7	3.8	p	p	p	P
hsa-miR-5194	3.7	4.1	3.7	4	p	p	p	P
hsa-miR-6765-3p	3.7	3.7	3.6	3.7	p	p	p	P
hsa-miR-378f	3.4	3.3	3.3	3.4	p	p	p	P
hsa-miR-4745-5p	3.3	3.6	3.3	3.6	p	p	p	P
hsa-miR-6829-5p	4	4.1	3.8	4.2	p	p	p	P
hsa-miR-4462	3.5	3.4	3.1	3.5	p	p	p	P
hsa-miR-1249-5p	3.2	3.6	3.5	3.5	p	p	p	P
hsa-miR-1273e	3.7	3.8	3.5	3.7	p	p	p	P
hsa-miR-4666b	3.6	3.5	3.6	3.7	p	p	p	P
hsa-miR-7847-3p	3.9	3.8	3.5	3.9	p	p	p	P
hsa-miR-135a-3p	3.4	3.8	3.3	3.6	p	p	p	P
hsa-miR-1233-5p	4	4	3.5	4.2	p	p	p	P
hsa-miR-16-2-3p	2.6	2.7	2.6	2.7	p	p	p	P
hsa-miR-129-1-3p	3.4	3.4	3.5	3.4	p	p	p	P
hsa-miR-6872-3p	3.2	3.2	3.1	3.3	p	p	p	P
hsa-miR-212-3p	3.6	3.8	3.3	4	p	p	A	P
hsa-miR-1185-1-3p	3.4	3.7	3.4	3.7	p	p	A	P
hsa-miR-500a-3p	3.2	3.6	3.2	3.5	p	p	A	P

hsa-miR-652-3p	3	3.6	3.1	3.4	p	p	A	P
hsa-miR-98-3p	3.6	3.5	3.5	3.7	p	p	A	P
hsa-miR-299-5p	3.5	3.8	3.4	3.6	p	p	A	P
hsa-miR-132-3p	3.4	4.4	3.4	4.1	p	p	A	P
hsa-miR-3125	3.7	4	3.5	4	p	p	A	P
hsa-miR-4455	3.9	3.8	3.4	4.1	p	p	A	P
hsa-miR-6727-5p	3.7	4	3.4	4.1	p	p	A	P
hsa-miR-6840-3p	3.5	3.6	3.4	3.7	p	p	A	P
hsa-miR-136-3p	3.5	3.7	3.3	3.7	p	p	A	P
hsa-miR-502-3p	3.3	3.7	3.1	3.7	p	p	A	P
hsa-miR-495-3p	3.3	3.7	3.1	3.5	p	p	A	P
hsa-miR-582-5p	3.8	4.5	4	4.3	p	p	p	P
hsa-miR-505-5p	3.7	3.9	3.7	4	p	p	p	P
hsa-miR-192-3p	6	5.8	5.9	5.9	p	p	p	P
hsa-miR-375	4.9	4.1	4.6	4.5	p	p	p	P
hsa-miR-194-3p	3.4	3.2	3.3	3.3	p	p	p	P
hsa-miR-19b-1-5p	2.6	2.7	2.7	2.8	p	p	p	P
hsa-miR-148a-5p	2.7	2.7	2.8	2.8	p	p	p	P
hsa-miR-5701	3.8	3.8	3.7	4	p	p	p	P
hsa-miR-429	3.6	4.2	4.1	4.9	p	p	p	P
hsa-miR-199b-5p	3.5	4.3	3.7	4.3	p	p	p	P
hsa-miR-4257	3.5	3.7	3.5	3.7	p	p	p	P
hsa-miR-3141	4.4	3.8	4	4.1	p	p	p	P
hsa-miR-5585-3p	3.7	3.9	3.5	4.2	p	p	p	P
hsa-miR-4634	4.3	4.1	3.6	4.4	p	p	p	P
hsa-miR-6775-5p	3.6	3.6	3.3	3.7	p	p	p	P
hsa-miR-6808-5p	3.9	3.8	3.6	4	p	p	p	P
hsa-miR-3190-5p	3.7	3.4	3.6	3.6	p	p	p	P
hsa-miR-3613-3p	3.4	3.5	3.5	3.6	p	p	p	P
hsa-miR-5684	3.4	3.9	3.3	3.5	p	p	A	P
hsa-miR-6850-5p	3.8	4	3.4	4	p	p	A	P
hsa-miR-4701-5p	3.5	3.5	3.5	3.5	p	p	A	P
hsa-miR-219a-5p	3.7	3.6	3.4	3.6	p	p	A	P
hsa-miR-4800-5p	3.8	3.8	3.4	3.8	p	p	A	P
hsa-miR-6794-5p	3.6	3.7	3.2	3.9	p	p	A	P
hsa-miR-4728-3p	3.4	3.3	3.3	3.5	p	p	A	P
hsa-miR-1972	3.1	3.3	3	3.4	p	p	A	P
hsa-miR-4499	4.1	3.8	3.7	4.1	p	p	p	P
hsa-miR-634	3.4	3.3	3.4	3.3	p	p	p	P
hsa-miR-499a-5p	3.7	3.6	3.6	3.9	p	p	p	P
hsa-miR-4298	3.8	3.8	3.4	3.8	p	p	p	P
hsa-miR-3614-5p	3.6	3.4	3.4	3.6	p	p	A	P
hsa-miR-28-3p	2.6	2.7	2.6	2.6	p	p	A	P
hsa-miR-6757-5p	3.5	3.6	3.1	3.5	p	p	A	P
hsa-miR-338-5p	2.5	2.6	2.5	2.6	p	p	A	P
hsa-miR-6798-5p	3.2	3.3	3.1	3.2	p	p	A	P
hsa-miR-1273f	3.1	3.1	2.8	3.1	p	p	A	P
hsa-miR-4667-5p	3.4	3.4	3	3.5	p	p	A	P
hsa-miR-345-5p	2.9	3	2.8	3	p	p	A	P
hsa-miR-3911	3.7	3.3	3	3.6	p	p	A	P
hsa-miR-4449	3.1	3.1	3	3.1	p	p	A	P
hsa-miR-6515-5p	3.4	3.6	3.3	3.6	p	p	p	P
hsa-miR-6786-5p	3.5	3.5	3.4	3.7	p	p	p	P
hsa-miR-1227-5p	3.8	3.1	3.7	3.5	p	A	p	P
hsa-miR-628-3p	3.2	3.3	3.3	3.4	p	p	A	P
hsa-miR-1229-3p	3.4	3.3	3.4	3.4	p	p	A	P
hsa-miR-6855-3p	3.5	3.4	3.4	3.5	p	p	A	P
hsa-miR-4433a-3p	4	3.4	3.7	3.6	p	A	p	P
hsa-miR-7152-5p	3.8	3.6	3.6	3.7	p	A	p	P
hsa-miR-378e	3.1	3	3	3.2	p	p	A	P
hsa-miR-337-5p	3.4	3.5	3	3.4	p	p	A	P
hsa-miR-4652-3p	3.5	3.4	3.5	3.4	p	p	A	A
hsa-miR-557	3.8	2.9	3.5	3.3	p	A	A	A
hsa-miR-5010-3p	3.4	3.4	3.5	3.6	A	p	A	P
hsa-miR-7-1-3p	3.3	3.6	3.3	3.5	A	p	A	P
hsa-miR-1288-3p	3.5	3.9	3.4	3.7	A	p	A	P
hsa-miR-7-5p	3.2	3.5	3.1	3.4	A	p	A	P
hsa-miR-342-5p	3.1	3.9	3.3	3.7	A	p	A	P
hsa-miR-6512-5p	3.5	3.9	3.4	3.8	A	p	A	P
hsa-miR-542-5p	3.4	3.7	3.3	3.4	A	p	A	P
hsa-miR-5699-5p	3.4	3.7	3.3	3.6	A	p	A	P
hsa-miR-6507-3p	3.5	3.3	3.5	3.5	A	p	A	P

hsa-miR-30c-1-3p	2.7	2.6	2.6	2.7	A	p	A	P
hsa-miR-3156-5p	3.3	3.8	3.2	3.6	A	p	A	P
hsa-miR-409-3p	3.4	3.6	3.3	3.6	A	p	A	P
hsa-miR-224-5p	3.1	4.4	3.3	4.4	A	p	p	P
hsa-miR-4646-3p	3.5	3.4	3.4	3.4	A	p	A	P
hsa-miR-4767	3.3	3.4	3.2	3.4	A	p	A	P
hsa-miR-150-3p	3.3	3.4	3	3.3	A	p	A	P
hsa-miR-21-3p	3	3.6	2.9	3.6	A	p	A	P
hsa-miR-181a-2-3p	2.8	3	2.9	3.1	A	p	A	P
hsa-miR-6759-3p	3.6	3.6	3.6	3.6	A	p	A	P
hsa-miR-7111-3p	3.6	3.4	3.4	3.5	A	p	A	P
hsa-miR-1307-5p	3.2	3.4	3	3.1	A	p	A	P
hsa-miR-6807-5p	3.5	3.8	3.3	3.7	A	p	A	P
hsa-miR-4697-5p	2.8	2.9	2.7	3	A	A	A	P
hsa-miR-6867-5p	3.4	3.5	3.1	3.8	A	p	A	P
hsa-miR-1271-5p	3.1	3.3	3.1	3.3	A	A	A	P
hsa-miR-181c-5p	3.1	3.4	2.9	3.4	A	p	A	P
hsa-miR-34a-3p	2.8	3.5	2.8	3.2	A	p	A	P
hsa-miR-30c-2-3p	3.1	3.2	3	3.2	A	p	A	P
hsa-miR-3620-5p	3.2	3.3	3	3.6	A	A	A	P
hsa-miR-6798-3p	3.2	3.2	3.3	3.2	A	A	A	P
hsa-miR-6728-5p	3.3	3.5	3.3	3.7	A	p	A	P
hsa-miR-1227-3p	3	3.1	3.1	3.2	A	A	A	P
hsa-miR-4793-5p	3.2	3.7	3.1	3.6	A	p	A	P
hsa-miR-6789-5p	3	3.4	3	3.3	A	p	A	P
hsa-miR-4312	3.3	3.3	3.4	3.4	A	p	A	P
hsa-miR-3648	3.1	3.5	3.1	3.4	A	p	A	P
hsa-miR-4651	3	3.7	3	3.5	A	p	A	P
hsa-miR-8060	3.4	3.4	3.3	3.6	A	p	A	P
hsa-miR-4695-5p	3.3	3.3	3	3.5	A	A	A	P
hsa-miR-6780a-5p	3.3	3.8	3.2	3.5	A	p	A	P
hsa-miR-379-5p	3.2	3.4	3.1	3.3	A	p	A	P
hsa-miR-500a-5p	2.8	3.1	2.8	2.9	A	p	A	P
hsa-miR-625-5p	2.8	3.4	2.8	3.2	A	p	A	P
hsa-miR-4317	2.8	3	2.7	3	A	A	A	P
hsa-miR-6756-3p	3.3	3.2	3.2	3.3	A	A	A	P
hsa-miR-665	3.3	3.1	3.1	3.2	A	p	A	P
hsa-miR-511-3p	3.3	3.8	3.2	3.6	A	p	A	P
hsa-miR-572	3	2.9	3	3	A	A	A	P
hsa-miR-7152-3p	3.3	3.6	3.2	3.6	A	p	A	P
hsa-miR-3652	3.2	3.3	3.1	3.3	A	p	A	P
hsa-miR-6086	3.3	3.5	3.2	3.4	A	p	A	P
hsa-miR-423-3p	3	3	2.9	3	A	p	A	P
hsa-miR-503-5p	2.8	3.1	2.9	3.1	A	p	A	P
hsa-miR-4633-5p	3	2.9	2.8	3.1	A	A	A	P
hsa-miR-23b-5p	2.7	2.7	2.6	2.8	A	A	A	P
hsa-miR-3679-3p	3.4	3.2	3.3	3.3	A	p	A	A
hsa-miR-663a	3	3.3	2.9	3.2	A	p	A	A
hsa-miR-96-5p	2.9	3.6	2.8	3.4	A	p	A	A
hsa-miR-328-5p	3	3.1	2.8	3.1	A	p	A	A
hsa-miR-6845-5p	3	3.3	2.9	3.3	A	p	A	A
hsa-miR-6510-5p	2.8	3.4	2.9	3.2	A	p	A	A
hsa-miR-664a-5p	3	3.2	3	3.2	A	p	A	A
hsa-miR-6516-3p	2.9	3.3	2.9	3.2	A	p	A	A
hsa-miR-551b-3p	2.9	3.3	2.9	3.1	A	p	A	A
hsa-miR-6075	2.9	3	2.8	2.9	A	p	A	A
hsa-miR-135b-5p	3.1	3.6	3.1	3.3	A	p	A	A
hsa-miR-424-3p	2.8	2.9	2.7	2.9	A	p	A	A
hsa-miR-452-5p	2.8	3.3	2.8	3	A	p	A	A

Abbreviations: IT, immune tolerant; IA, immune active; IC, inactive carrier; ENH, HBeAg-negative hepatitis; P, present; A, absent. Values represent the medium of normalized signal values for each CHB phase.

Table S5. Differentially expressed miRNAs (n = 273) in pairwise comparisons across CHB clinical phases.

miRNA	IA vs. IT		IA vs. IC		ENH vs. IT		ENH vs. IC		IA vs. ENH	
	adj. p	log$_2$FC	adj. p	log$_2$FC	adj. p	log$_2$FC	adj. p	log$_2$FC	adj. p	log$_2$FC
hsa-miR-155-5p	0.002	1.66	0.000	1.62	0.039	1.13	0.008	1.08		
hsa-miR-34b-5p	0.006	1.63	0.003	1.52			0.031	1.17		
hsa-miR-224-5p	0.023	1.38	0.029	1.20	0.039	1.28				
hsa-miR-146b-5p	0.010	1.23	0.002	1.08	0.043	0.77	0.037	0.62		
hsa-miR-142-5p	0.002	1.09	0.000	1.04	0.043	0.80	0.013	0.75		
hsa-miR-150-5p	0.002	1.11	0.000	0.99	0.039	0.80	0.009	0.68		
hsa-miR-181b-5p	0.010	0.81	0.000	0.97			0.007	0.68		
hsa-miR-132-3p	0.005	0.86	0.000	0.95	0.039	0.60	0.007	0.69		
hsa-miR-146a-5p	0.002	1.00	0.000	0.87	0.043	0.69	0.037	0.57		
hsa-miR-142-3p	0.005	0.94	0.000	0.87						
hsa-miR-34a-5p	0.008	1.30	0.005	0.87						
hsa-miR-21-3p	0.002	0.75	0.000	0.82	0.039	0.60	0.007	0.68		
hsa-miR-222-3p	0.006	0.76	0.000	0.81			0.008	0.44		
hsa-miR-128-3p	0.027	0.70	0.005	0.80			0.004	0.56		
hsa-miR-6858-5p			0.014	0.75			0.049	0.86		
hsa-miR-1973			0.032	0.73						
hsa-miR-125a-3p			0.010	0.72			0.023	0.79		
hsa-miR-218-5p	0.023	0.77	0.004	0.71			0.019	0.86		
hsa-miR-21-5p	0.002	0.89	0.001	0.71	0.039	0.65	0.008	0.48		
hsa-miR-301a-3p	0.038	0.52	0.001	0.71			0.005	0.51		
hsa-miR-127-3p			0.000	0.71			0.008	0.52		
hsa-miR-223-3p			0.010	0.66			0.042	0.35		
hsa-miR-4485-3p			0.037	0.65						
hsa-miR-6812-5p			0.012	0.65						
hsa-miR-3135b			0.018	0.64			0.023	1.16		
hsa-miR-99b-5p	0.032	0.77	0.021	0.63			0.019	0.42		
hsa-miR-96-5p			0.010	0.62			0.002	0.66		
hsa-miR-4741			0.023	0.62			0.035	1.16		
hsa-miR-342-5p	0.002	0.71	0.000	0.61	0.039	0.56	0.005	0.46		
hsa-miR-34a-3p	0.006	0.58	0.001	0.61	0.043	0.45	0.009	0.48		
hsa-miR-5684			0.001	0.60						
hsa-miR-4667-5p			0.009	0.59			0.035	0.66		
hsa-miR-5585-3p			0.042	0.59						
hsa-miR-1181			0.019	0.56						
hsa-miR-361-3p			0.004	0.56			0.019	0.44		
hsa-miR-511-3p			0.004	0.56			0.011	0.40		
hsa-miR-619-5p			0.029	0.56						
hsa-miR-4651			0.018	0.56			0.034	0.27		
hsa-miR-221-3p	0.019	0.73	0.048	0.55	0.039	0.48	0.009	0.31		
hsa-miR-6510-5p			0.019	0.55			0.049	0.83		
hsa-miR-495-3p			0.000	0.55			0.003	0.36		
hsa-miR-4800-5p			0.029	0.54						
hsa-miR-513b-5p			0.015	0.54						
hsa-miR-6867-5p			0.014	0.53			0.034	0.76		
hsa-miR-6780a-5p			0.004	0.53			0.013	0.37		
hsa-miR-542-3p	0.019	0.55	0.003	0.53			0.027	0.34		
hsa-miR-363-3p			0.021	0.53						
hsa-miR-4716-3p			0.000	0.52			0.004	0.44		
hsa-miR-377-3p			0.004	0.52						
hsa-miR-18a-5p			0.021	0.52			0.003	0.48		
hsa-miR-1288-3p	0.007	0.39	0.000	0.52			0.004	0.37		
hsa-miR-135a-3p			0.014	0.51						
hsa-miR-8063			0.015	0.51			0.042	0.35		
hsa-miR-575			0.014	0.51			0.023	0.54		
hsa-miR-4746-3p			0.015	0.51						
hsa-miR-3679-5p			0.012	0.51			0.035	0.52		
hsa-miR-1246			0.038	0.50			0.041	0.25		
hsa-miR-450a-5p			0.041	0.50			0.037	0.41		
hsa-miR-4739	0.032	0.56	0.026	0.50						
hsa-miR-6807-5p			0.005	0.50			0.049	0.46		

hsa-miR-337-5p			0.001	0.50			0.013	0.30
hsa-miR-5581-5p	0.020	0.33	0.000	0.50			0.004	0.42
hsa-miR-3156-5p			0.005	0.50			0.008	0.39
hsa-miR-409-3p			0.001	0.50			0.009	0.31
hsa-miR-5699-5p			0.014	0.49				
hsa-miR-502-3p	0.027	0.36	0.001	0.49			0.004	0.41
hsa-miR-6734-5p			0.001	0.49			0.002	0.39
hsa-miR-503-5p	0.038	0.48	0.021	0.48				
hsa-miR-6767-5p	0.007	0.42	0.000	0.48			0.007	0.31
hsa-miR-1972			0.023	0.48				
hsa-miR-4478			0.015	0.48				
hsa-miR-342-3p	0.005	0.57	0.004	0.47				
hsa-miR-454-3p	0.046	0.44	0.001	0.47			0.003	0.33
hsa-miR-6086			0.019	0.47				
hsa-miR-4728-5p			0.023	0.47				
hsa-miR-4793-5p			0.019	0.46			0.049	0.38
hsa-miR-3125			0.000	0.45			0.000	0.49
hsa-miR-185-5p			0.021	0.45			0.031	0.40
hsa-miR-4672			0.048	0.45				
hsa-miR-181c-5p	0.010	0.40	0.002	0.45			0.019	0.31
hsa-miR-542-5p			0.005	0.45			0.023	0.27
hsa-miR-154-5p			0.003	0.44			0.002	0.38
hsa-miR-136-3p			0.000	0.44			0.005	0.39
hsa-miR-16-5p	0.032	0.34	0.000	0.44			0.006	0.27
hsa-miR-513c-5p			0.026	0.44				
hsa-miR-6512-5p			0.001	0.44			0.002	0.42
hsa-miR-582-5p	0.027	0.54	0.029	0.44	0.043	0.43	0.023	0.33
hsa-miR-3648			0.019	0.44				
hsa-miR-1185-1-3p			0.029	0.43			0.049	0.20
hsa-miR-6845-5p			0.026	0.43			0.030	0.65
hsa-miR-7-5p	0.006	0.34	0.000	0.42	0.039	0.30	0.001	0.38
hsa-miR-6727-5p			0.029	0.42				
hsa-miR-1273e			0.023	0.42				
hsa-miR-4306			0.015	0.42	0.043	0.42	0.008	0.44
hsa-miR-625-5p	0.008	0.41	0.000	0.42	0.039	0.37	0.003	0.39
hsa-miR-7847-3p			0.037	0.42				
hsa-miR-6780b-5p			0.023	0.42				
hsa-miR-660-5p	0.032	0.41	0.007	0.41			0.027	0.34
hsa-miR-642b-3p			0.026	0.41				
hsa-miR-381-3p			0.012	0.41				
hsa-miR-6850-5p			0.029	0.41				
hsa-miR-4428			0.023	0.41				
hsa-miR-6794-5p			0.023	0.41			0.023	0.62
hsa-miR-663a			0.010	0.41			0.030	0.45
hsa-miR-4462			0.015	0.41			0.049	0.20
hsa-miR-452-5p	0.005	0.42	0.001	0.41	0.039	0.34	0.009	0.33
hsa-miR-1273f			0.048	0.40				
hsa-miR-181a-5p	0.014	0.59	0.021	0.40				
hsa-miR-98-5p			0.007	0.40			0.007	0.41
hsa-miR-532-3p			0.010	0.40			0.008	0.37
hsa-miR-3646			0.014	0.40				
hsa-miR-379-5p			0.000	0.39			0.031	0.21
hsa-miR-6724-5p			0.042	0.39				
hsa-miR-487b-3p			0.019	0.39			0.011	0.32
hsa-miR-7110-5p			0.019	0.38			0.027	0.33
hsa-miR-1229-5p			0.037	0.38				
hsa-miR-376a-3p			0.035	0.38				
hsa-miR-362-3p			0.007	0.38			0.009	0.39
hsa-miR-532-5p	0.038	0.56	0.021	0.38				
hsa-miR-299-5p			0.008	0.38			0.011	0.22
hsa-miR-6516-3p	0.008	0.31	0.000	0.38	0.039	0.25	0.001	0.32
hsa-miR-6126			0.022	0.37				
hsa-miR-652-3p	0.023	0.35	0.005	0.37			0.008	0.26
hsa-miR-186-5p			0.015	0.36			0.008	0.45
hsa-miR-135b-5p			0.021	0.36			0.019	0.30

hsa-miR-150-3p			0.029	0.36				
hsa-miR-136-5p			0.029	0.35				
hsa-miR-7152-3p			0.014	0.35			0.023	0.51
hsa-miR-4430			0.042	0.35				
hsa-miR-424-5p	0.046	0.50	0.048	0.34				
hsa-miR-6740-5p			0.004	0.34			0.035	0.32
hsa-miR-5194	0.020	0.27	0.001	0.34			0.027	0.25
hsa-miR-4695-5p			0.029	0.34				
hsa-miR-1290			0.005	0.33			0.049	0.23
hsa-miR-27a-3p			0.048	0.33				
hsa-miR-3652			0.023	0.33				
hsa-miR-3198			0.004	0.32				
hsa-miR-500a-5p	0.007	0.24	0.000	0.32			0.013	0.21
hsa-let-7i-5p	0.005	0.42	0.001	0.32				
hsa-miR-4532			0.023	0.32				
hsa-miR-1307-5p			0.013	0.32				
hsa-miR-4697-5p			0.032	0.31				
hsa-miR-6791-5p			0.042	0.31				
hsa-miR-425-5p			0.029	0.31				
hsa-miR-7150			0.048	0.31				
hsa-miR-500a-3p			0.008	0.31	0.043	0.24	0.007	0.25
hsa-miR-4788			0.026	0.30				
hsa-miR-4713-3p			0.004	0.30				
hsa-miR-5088-5p			0.001	0.30				
hsa-miR-1914-3p			0.015	0.29				
hsa-miR-6752-5p			0.023	0.29				
hsa-miR-362-5p			0.029	0.29				
hsa-miR-6717-5p			0.009	0.29				
hsa-miR-18b-5p			0.009	0.28			0.008	0.21
hsa-miR-1305	0.043	0.28	0.011	0.28			0.047	0.24
hsa-miR-4465			0.042	0.27				
hsa-miR-6789-5p	0.038	0.28	0.021	0.27				
hsa-miR-6879-5p			0.001	0.27			0.042	0.23
hsa-miR-374c-5p	0.038	0.27	0.005	0.27	0.039	0.32	0.002	0.33
hsa-miR-654-3p			0.041	0.27			0.016	0.22
hsa-miR-4745-5p			0.007	0.26			0.038	0.19
hsa-miR-374b-5p			0.029	0.26			0.037	0.23
hsa-miR-4530			0.032	0.25				
hsa-miR-148b-3p			0.029	0.25			0.009	0.34
hsa-miR-4633-5p			0.029	0.24			0.003	0.38
hsa-miR-7-1-3p			0.010	0.24			0.013	0.24
hsa-miR-424-3p			0.019	0.23				
hsa-miR-4317			0.000	0.23			0.001	0.27
hsa-miR-4767			0.023	0.22			0.035	0.20
hsa-miR-345-5p			0.005	0.22			0.035	0.10
hsa-miR-4449			0.041	0.21			0.008	0.23
hsa-miR-664a-5p			0.029	0.21			0.003	0.44
hsa-miR-4442			0.032	0.20			0.049	0.22
hsa-miR-6808-5p			0.037	0.20				
hsa-miR-6798-5p			0.032	0.19				
hsa-miR-3151-3p			0.029	0.18				
hsa-miR-6131			0.029	0.18				
hsa-miR-4687-3p			0.048	0.18				
hsa-let-7g-5p			0.010	0.16	0.043	0.30	0.005	0.14
hsa-miR-423-3p			0.029	0.12				
hsa-miR-28-3p			0.048	0.08				
hsa-miR-338-5p			0.041	0.06				
hsa-miR-4634							0.035	1.37
hsa-miR-3620-5p							0.002	0.87
hsa-miR-200a-3p					0.039	1.16	0.031	0.86
hsa-miR-4455							0.003	0.76
hsa-miR-429	0.046	1.05			0.039	1.12	0.042	0.76
hsa-miR-328-5p							0.042	0.68
hsa-miR-3911							0.035	0.61
hsa-miR-211-3p							0.002	0.58

hsa-miR-214-5p					0.039	0.54	0.006	0.51		
hsa-miR-22-5p							0.008	0.50		
hsa-miR-212-3p							0.012	0.45		
hsa-miR-3907							0.013	0.45		
hsa-miR-6829-5p							0.042	0.37		
hsa-miR-6723-5p							0.003	0.37		
hsa-miR-1233-5p							0.046	0.36		
hsa-miR-324-5p							0.013	0.33		
hsa-miR-145-3p							0.042	0.33		
hsa-miR-6840-3p							0.013	0.32		
hsa-miR-505-5p							0.011	0.32		
hsa-miR-126-5p							0.023	0.30		
hsa-miR-3196							0.046	0.30		
hsa-miR-4787-5p							0.042	0.29		
hsa-miR-125b-2-3p							0.006	0.26		
hsa-miR-4443							0.035	0.22		
hsa-miR-29c-5p							0.023	0.21		
hsa-miR-6872-3p							0.033	0.20		
hsa-miR-152-3p							0.042	0.20		
hsa-miR-1271-5p					0.039	0.23	0.031	0.19		
hsa-miR-181a-2-3p					0.039	0.23	0.005	0.17		
hsa-miR-4698							0.035	0.15		
hsa-miR-23b-5p							0.011	0.15		
hsa-let-7f-5p					0.043	0.35	0.008	0.13		
hsa-miR-16-2-3p					0.039	0.14	0.042	0.12		
hsa-miR-200b-3p					0.043	1.07				
hsa-miR-338-3p	0.027	0.59			0.039	0.89				
hsa-miR-214-3p					0.039	0.60				
hsa-miR-199a-3p					0.039	0.54				
hsa-miR-125a-5p					0.043	0.53				
hsa-miR-10a-5p					0.043	0.49				
hsa-miR-199a-5p					0.039	0.47				
hsa-miR-195-5p					0.043	0.45				
hsa-miR-23a-3p	0.027	0.44			0.043	0.36				
hsa-miR-3679-3p			0.037	-0.12						
hsa-miR-296-5p			0.011	-0.21						
hsa-miR-99a-5p			0.010	-0.24						
hsa-miR-30b-5p			0.003	-0.26			0.015	-0.22		
hsa-miR-6766-3p			0.005	-0.26						
hsa-miR-6515-3p			0.003	-0.28						
hsa-miR-192-5p			0.004	-0.30						
hsa-miR-27b-3p			0.005	-0.30						
hsa-miR-1228-3p			0.033	-0.30						
hsa-miR-6085	0.035	-0.31	0.004	-0.32						
hsa-miR-365a-3p	0.035	-0.38	0.033	-0.32						
hsa-miR-8069			0.002	-0.33						
hsa-miR-215-5p			0.003	-0.33						
hsa-miR-1260b			0.037	-0.33						
hsa-miR-19a-3p			0.023	-0.36						
hsa-miR-30e-5p	0.035	-0.38	0.001	-0.37			0.046	-0.24		
hsa-miR-92a-3p			0.004	-0.38						
hsa-miR-194-5p	0.035	-0.28	0.001	-0.40						
hsa-miR-30c-5p			0.001	-0.41			0.031	-0.30		
hsa-miR-23b-3p			0.001	-0.42						
hsa-miR-125b-5p			0.001	-0.43					0.016	-0.27
hsa-miR-122-3p	0.035	-0.32	0.001	-0.47						
hsa-miR-1225-5p			0.003	-0.47			0.013	-0.43		
hsa-miR-1260a	0.041	-0.35	0.002	-0.47						
hsa-miR-3653-3p			0.032	-0.48						
hsa-miR-4433a-3p	0.002	-0.71	0.030	-0.48						
hsa-miR-30d-5p			0.001	-0.50			0.009	-0.36		
hsa-miR-1249-3p	0.031	-0.38	0.000	-0.52			0.002	-0.42		
hsa-miR-148a-3p			0.004	-0.55						
hsa-miR-1207-5p	0.002	-0.69	0.003	-0.55						
hsa-miR-101-3p			0.001	-0.58						

hsa-miR-885-5p	0.035	-0.48	0.001	-0.62						
hsa-miR-144-3p			0.042	-0.64						
hsa-miR-1268a	0.002	-0.92	0.001	-0.70						
hsa-miR-4763-3p	0.026	-0.67	0.013	-0.70						
hsa-miR-6088	0.002	-0.82	0.000	-0.70						
hsa-miR-638	0.014	-0.59	0.023	-0.72						
hsa-miR-557	0.003	-0.84	0.007	-0.74						
hsa-miR-1227-5p	0.020	-0.74	0.026	-0.74						
hsa-miR-455-3p			0.001	-0.76						
hsa-miR-4281	0.002	-0.75	0.000	-0.81			0.022	-0.49		
hsa-miR-4270	0.002	-1.59	0.000	-1.75			0.002	-0.94		
hsa-miR-4299							0.035	-0.24		
hsa-miR-6127							0.025	-0.31		
hsa-miR-3656	0.004	-0.37								
hsa-miR-1224-5p	0.026	-0.48								
hsa-miR-6068	0.026	-0.67								
hsa-miR-451a	0.035	-0.70								
hsa-miR-2861	0.017	-0.72								

Differential expression was assessed by Mann–Whitney U test with Benjamini–Hochberg FDR correction (adjusted $p < 0.05$). This table lists all 273 miRNAs with adjusted $p < 0.05$ in at least one of the five shown comparisons; columns report adjusted p-values and $\log_2$ fold changes. Blank cells denote comparisons in which FDR-adjusted $p \geqslant 0.05$. The IT vs. IC comparison is omitted because it yielded no significant miRNAs.

Abbreviations: CHB, chronic hepatitis B; IT, immune tolerance; IA, immune active; IC, inactive carrier; ENH, HBeAg-negative hepatitis; FDR, false discovery rate; FC, fold change.

Table S6. 22 significantly up-regulated miRNAs in IA phase compared to IT and IC phases.

miRNA	IA vs. IC		IA vs. IT		Hepatic cell–type enrichment	Previous reports	
	adj. p	$\log_2$FC	adj. p	$\log_2$FC		Reported immune cell associations	References
hsa-miR-155-5p	<0.001	1.62	<0.005	1.66	KC	DC, macrophage, B cell, T cell, KC	(1-5)
hsa-miR-142-5p	<0.001	1.04	<0.005	1.09	KC	T cell, B cell, macrophage	(6-9)
hsa-miR-150-5p	<0.001	0.99	<0.005	1.11		T cell, DC	(9-12)
hsa-miR-181b-5p	<0.001	0.97	<0.05	0.81		T cell, B cell, macrophage, monocyte	(13-16)
hsa-miR-132-3p	<0.001	0.95	<0.01	0.86		macrophage	(17, 18)
hsa-miR-146a-5p	<0.001	0.87	<0.005	1.00		macrophage, DC	(19-21)
hsa-miR-142-3p	<0.001	0.87	<0.005	0.94		T cell, DC, macrophage	(7, 12, 22, 23)
hsa-miR-21-3p	<0.001	0.82	<0.005	0.75		T cell, macrophage	(24-27)
hsa-miR-222-3p	<0.001	0.81	<0.01	0.76	LSEC	macrophage	(19, 28, 29)
hsa-miR-301a-3p	<0.001	0.71				NK cell, macrophage, T cell	(30-33)
hsa-miR-127-3p	<0.001	0.71			HSC	macrophage	(34-36)
hsa-miR-342-5p	<0.001	0.61	<0.005	0.71		macrophage	(37)
hsa-miR-34a-3p	<0.001	0.61				T cell, DC	(38-40)
hsa-miR-34b-5p	<0.005	1.52	<0.01	1.63		B cell, macrophage	(41, 42)
hsa-miR-146b-5p	<0.005	1.08	<0.05	1.23		Macrophage, DC, T cell	(21, 43)
hsa-miR-218-5p	<0.005	0.71	<0.05	0.77		NK cell	(44)
hsa-miR-21-5p	<0.005	0.71	<0.005	0.89	KC	T cell, macrophage, DC	(45-47)
hsa-miR-34a-5p	<0.01	0.87	<0.01	1.30		T cell, macrophage	(48-50)
hsa-miR-128-3p	<0.01	0.80	<0.05	0.70		DC, T cell	(51)
hsa-miR-223-3p	<0.01	0.66				neutrophils, macrophage, DC	(47, 52-58)
hsa-miR-96-5p	<0.01	0.62				T cell	(59)
hsa-miR-4667-5p	<0.01	0.59				-	(-)

Differential expression was assessed by Mann–Whitney U test with Benjamini–Hochberg correction (adjusted $p < 0.05$). Only miRNAs with $|\log_2 FC| > \log_2 1.5$ (≈ 0.585) in IA vs. IT or IA vs. IC are shown. Blank cells denote comparisons in which a miRNA failed to meet both the adjusted $p < 0.05$ and $|\log_2 FC| > \log_2 1.5$ criteria. "Hepatic cell–type enrichment" indicates the hepatic cell type (PHH, KC, HSC, LSEC) in which each miRNA is most highly expressed (see Fig. 4B). "Reported immune cell associations" lists the immune cell types in which each miRNA has documented regulatory roles (see References). Abbreviations: IT, immune tolerance; IA, immune active; IC, inactive carrier state; FC, fold change; PHH, primary human hepatocyte; KC, Kupffer cell; LSEC, liver sinusoidal endothelial cell; HSC, hepatic stellate cell; NK, natural killer cell; DC, dendritic cell.

References

1. Yao R, Ma YL, Liang W, Li HH, Ma ZJ, Yu X, Liao YH. MicroRNA-155 modulates Treg and Th17 cells differentiation and Th17 cell function by targeting SOCS1. PLoS One 2012;7:e46082.
2. Lind EF, Millar DG, Dissanayake D, Savage JC, Grimshaw NK, Kerr WG, Ohashi PS. miR-155 Upregulation in Dendritic Cells Is Sufficient To Break Tolerance In Vivo by Negatively Regulating SHIP1. J Immunol 2015;195:4632-4640.
3. O'Connell RM, Taganov KD, Boldin MP, Cheng G, Baltimore D. MicroRNA-155 is induced during the macrophage inflammatory response. Proc Natl Acad Sci U S A 2007;104:1604-1609.
4. Rodriguez A, Vigorito E, Clare S, Warren MV, Couttet P, Soond DR, van Dongen S, et al. Requirement of bic/microRNA-155 for normal immune function. Science 2007;316:608-611.
5. Blaya D, Aguilar-Bravo B, Hao F, Casacuberta-Serra S, Coll M, Perea L, Vallverdu J, et al. Expression of microRNA-155 in inflammatory cells modulates liver injury. Hepatology 2018;68:691-706.
6. Wang WL, Ouyang C, Graham NM, Zhang Y, Cassady K, Reyes EY, Xiong M, et al. microRNA-142 guards against autoimmunity by controlling Treg cell homeostasis and function. PLoS Biol 2022;20:e3001552.
7. Talebi F, Ghorbani S, Chan WF, Boghozian R, Masoumi F, Ghasemi S, Vojgani M, et al. MicroRNA-142 regulates inflammation and T cell differentiation in an animal model of multiple sclerosis. J Neuroinflammation 2017;14:55.
8. Ding S, Liang Y, Zhao M, Liang G, Long H, Zhao S, Wang Y, et al. Decreased microRNA-142-3p/5p expression causes CD4+ T cell activation and B cell hyperstimulation in systemic lupus erythematosus. Arthritis Rheum 2012;64:2953-2963.
9. Shi J, Chen M, Ouyang L, Wang Q, Guo Y, Huang L, Jiang S. miR-142-5p and miR-130a-3p regulate pulmonary macrophage polarization and asthma airway remodeling. Immunol Cell Biol 2020;98:715-725.
10. Smith NL, Wissink EM, Grimson A, Rudd BD. miR-150 Regulates Differentiation and Cytolytic Effector Function in CD8+ T cells. Sci Rep 2015;5:16399.
11. Li Y, Yu J, Wang F, Guo R, Xing H, Chen Y, Chen D, et al. MiR-150-5p regulate T cell activation in severe aplastic anemia by targeting Bach2. Cell Tissue Res 2021;384:423-434.

12. Tung SL, Boardman DA, Sen M, Letizia M, Peng Q, Cianci N, Dioni L, et al. Regulatory T cell-derived extracellular vesicles modify dendritic cell function. Sci Rep 2018;8:6065.
13. Ghorbani S, Talebi F, Chan WF, Masoumi F, Vojgani M, Power C, Noorbakhsh F. MicroRNA-181 Variants Regulate T Cell Phenotype in the Context of Autoimmune Neuroinflammation. Front Immunol 2017;8:758.
14. Di Marco M, Veschi S, Lanuti P, Ramassone A, Pacillo S, Pagotto S, Pepe F, et al. Enhanced Expression of miR-181b in B Cells of CLL Improves the Anti-Tumor Cytotoxic T Cell Response. Cancers (Basel) 2021;13.
15. Ma J, Chen S, Liu Y, Han H, Gong M, Song Y. The role of exosomal miR-181b in the crosstalk between NSCLC cells and tumor-associated macrophages. Genes Genomics 2022;44:1243-1258.
16. Wang Z, Li C, Sun X, Li Z, Li J, Wang L, Sun Y. Hypermethylation of miR-181b in monocytes is associated with coronary artery disease and promotes M1 polarized phenotype via PIAS1-KLF4 axis. Cardiovasc Diagn Ther 2020;10:738-751.
17. Fang H, Li HF, Pan Q, Jin HL, Yang M, Wang RR, Wang QY, et al. MiR-132-3p Modulates MEKK3-Dependent NF-kappaB and p38/JNK Signaling Pathways to Alleviate Spinal Cord Ischemia-Reperfusion Injury by Hindering M1 Polarization of Macrophages. Front Cell Dev Biol 2021;9:570451.
18. Renaudineau Y, Berindan-Neagoe I, Stanciu LA. Editorial: Role of Macrophage MicroRNAs in Inflammatory Diseases and Cancer. Front Immunol 2021;12:764525.
19. Li Y, Zhao L, Shi B, Ma S, Xu Z, Ge Y, Liu Y, et al. Functions of miR-146a and miR-222 in Tumor-associated Macrophages in Breast Cancer. Sci Rep 2015;5:18648.
20. Galbiati V, Lefevre MA, Maddalon A, Vocanson M, Iulini M, Marinovich M, Corsini E. Role of miR-24-3p and miR-146a-5p in dendritic cells' maturation process induced by contact sensitizers. Arch Toxicol 2023;97:2183-2191.
21. Ding J, Zhang Y, Cai X, Zhang Y, Yan S, Wang J, Zhang S, et al. Extracellular vesicles derived from M1 macrophages deliver miR-146a-5p and miR-146b-5p to suppress trophoblast migration and invasion by targeting TRAF6 in recurrent spontaneous abortion. Theranostics 2021;11:5813-5830.
22. Naqvi AR, Fordham JB, Ganesh B, Nares S. miR-24, miR-30b and miR-142-3p interfere with antigen processing and presentation by primary macrophages and dendritic cells. Sci Rep 2016;6:32925.
23. Sun Y, Varambally S, Maher CA, Cao Q, Chockley P, Toubai T, Malter C, et al. Targeting of microRNA-142-3p in dendritic cells regulates endotoxin-induced mortality. Blood 2011;117:6172-6183.
24. Han Z, Estephan RJ, Wu X, Su C, Yuan YC, Qin H, Kil SH, et al. MicroRNA Regulation of T-Cell Exhaustion in Cutaneous T Cell Lymphoma. J Invest Dermatol 2022;142:603-612 e607.
25. Huang Y, Huang Y, Cai Z, Ferrari MW, Li C, Zhang T, Lyu G, et al. MiR-21-3p inhibitor exerts myocardial protective effects by altering macrophage polarization state and reducing excessive mitophagy. Commun Biol 2024;7:1371.
26. Guo W, Wu Z, Chen J, Guo S, You W, Wang S, Ma J, et al. Nanoparticle delivery of miR-21-3p sensitizes melanoma to anti-PD-1 immunotherapy by promoting ferroptosis. J Immunother Cancer 2022;10.
27. Xi J, Huang Q, Wang L, Ma X, Deng Q, Kumar M, Zhou Z, et al. miR-21 depletion in macrophages promotes tumoricidal polarization and enhances PD-1 immunotherapy. Oncogene 2018;37:3151-3165.
28. Njock MS, O'Grady T, Nivelles O, Lion M, Jacques S, Cambier M, Herkenne S, et al. Endothelial extracellular vesicles promote tumour growth by tumour-associated macrophage reprogramming. J Extracell Vesicles 2022;11:e12228.
29. Ying X, Wu Q, Wu X, Zhu Q, Wang X, Jiang L, Chen X, et al. Epithelial ovarian cancer-secreted exosomal miR-222-3p induces polarization of tumor-associated macrophages. Oncotarget 2016;7:43076-43087.
30. Zhang J, Yang Y, Wei Y, Li L, Wang X, Ye Z. Hsa-miR-301a-3p inhibited the killing effect of natural killer cells on non-small cell lung cancer cells by regulating RUNX3. Cancer Biomark 2023;37:249-259.
31. Shou Y, Wang X, Chen C, Liang Y, Yang C, Xiao Q, Li H, et al. Exosomal miR-301a-3p from esophageal squamous cell carcinoma cells promotes angiogenesis by inducing M2 polarization of macrophages via the PTEN/PI3K/AKT signaling pathway. Cancer Cell Int 2022;22:153.
32. Luo S, Wu R, Li Q, Zhang G. MiR-301a-3p Advances IRAK1-Mediated Differentiation of Th17 Cells to Promote the Progression of Systemic Lupus Erythematosus via Targeting PELI1. J Healthc Eng 2021;2021:2982924.
33. Wang X, Luo G, Zhang K, Cao J, Huang C, Jiang T, Liu B, et al. Hypoxic Tumor-Derived Exosomal miR-301a Mediates M2 Macrophage Polarization via PTEN/PI3Kgamma to Promote Pancreatic Cancer Metastasis. Cancer Res 2018;78:4586-4598.
34. Liu Y, Wu Y, Wang C, Hu W, Zou S, Ren H, Zuo Y, et al. MiR-127-3p enhances macrophagic proliferation via disturbing fatty acid profiles and oxidative phosphorylation in atherosclerosis. J Mol Cell Cardiol 2024;193:36-52.
35. Wu X, Wu J, Dai T, Wang Q, Cai S, Wei X, Chen J, et al. beta-elemene promotes miR-127-3p maturation, induces NSCLCs autophagy, and enhances macrophage M1 polarization through exosomal communication. J Pharm Anal 2024;14:100961.
36. Ying H, Kang Y, Zhang H, Zhao D, Xia J, Lu Z, Wang H, et al. MiR-127 modulates macrophage polarization and promotes lung inflammation and injury by activating the JNK pathway. J Immunol 2015;194:1239-1251.
37. Wei Y, Nazari-Jahantigh M, Chan L, Zhu M, Heyll K, Corbalan-Campos J, Hartmann P, et al. The microRNA-342-5p fosters inflammatory macrophage activation through an Akt1- and microRNA-155-dependent pathway during atherosclerosis. Circulation 2013;127:1609-1619.
38. Taheri F, Ebrahimi SO, Shareef S, Reiisi S. Regulatory and immunomodulatory role of miR-34a in T cell immunity. Life Sci 2020;262:118209.
39. Hart M, Walch-Ruckheim B, Friedmann KS, Rheinheimer S, Tanzer T, Glombitza B, Sester M, et al. miR-34a: a new player in the regulation of T cell function by modulation of NF-kappaB signaling. Cell Death Dis 2019;10:46.
40. Huang A, Yang Y, Chen S, Xia F, Sun D, Fang D, Xiong S, et al. MiR-34a promotes DCs development and inhibits their function on T cell activation by targeting WNT1. Oncotarget 2017;8:17191-17201.
41. Qian CS, Li LJ, Huang HW, Yang HF, Wu DP. MYC-regulated lncRNA NEAT1 promotes B cell proliferation and lymphomagenesis via the miR-34b-5p-GLI1 pathway in diffuse large B-cell lymphoma. Cancer Cell Int 2020;20:87.
42. Duan C, Liu H, Yang X, Liu J, Deng Y, Wang T, Xing J, et al. Sirtuin1 inhibits calcium oxalate crystal-induced kidney injury by regulating TLR4 signaling and macrophage-mediated inflammatory activation. Cell Signal 2023;112:110887.
43. Testa U, Pelosi E, Castelli G, Labbaye C. miR-146 and miR-155: Two Key Modulators of Immune Response and Tumor Development. Noncoding RNA 2017;3.
44. Yang Q, Li J, Hu Y, Tang X, Yu L, Dong L, Chen D. MiR-218-5p Suppresses the Killing Effect of Natural Killer Cell to Lung Adenocarcinoma by Targeting SHMT1. Yonsei Med J 2019;60:500-508.
45. Yu H, Pan J, Zheng S, Cai D, Luo A, Xia Z, Huang J. Hepatocellular Carcinoma Cell-Derived Exosomal miR-21-5p Induces Macrophage M2 Polarization by Targeting RhoB. Int J Mol Sci 2023;24.
46. Pu J, Xu Z, Nian J, Fang Q, Yang M, Huang Y, Li W, et al. M2 macrophage-derived extracellular vesicles facilitate CD8+T cell exhaustion in hepatocellular carcinoma via the miR-21-5p/YOD1/YAP/beta-catenin pathway. Cell Death Discov 2021;7:182.
47. Reis M, Mavin E, Nicholson L, Green K, Dickinson AM, Wang XN. Mesenchymal Stromal Cell-Derived Extracellular Vesicles Attenuate Dendritic Cell Maturation and Function. Front Immunol 2018;9:2538.
48. Yang P, Li QJ, Feng Y, Zhang Y, Markowitz GJ, Ning S, Deng Y, et al. TGF-beta-miR-34a-CCL22 signaling-induced Treg cell recruitment promotes venous metastases of HBV-positive hepatocellular carcinoma. Cancer Cell 2012;22:291-303.
49. McCubbrey AL, Nelson JD, Stolberg VR, Blakely PK, McCloskey L, Janssen WJ, Freeman CM, et al. MicroRNA-34a Negatively Regulates Efferocytosis by Tissue Macrophages in Part via SIRT1. J Immunol 2016;196:1366-1375.

50. Li J, Che L, Xu C, Lu D, Xu Y, Liu M, Chai W. XIST/miR-34a-5p/PDL1 axis regulated the development of lung cancer cells and the immune function of CD8(+) T cells. J Recept Signal Transduct Res 2022;42:469-478.
51. Liang X, Shangguan W, Zhang M, Mei S, Wang L, Yang R. miR-128 enhances dendritic cell-mediated anti-tumor immunity via targeting of p38. Mol Med Rep 2017;16:1307-1313.
52. Jimenez Calvente C, Del Pilar H, Tameda M, Johnson CD, Feldstein AE. MicroRNA 223 3p Negatively Regulates the NLRP3 Inflammasome in Acute and Chronic Liver Injury. Mol Ther 2020;28:653-663.
53. Kim GD, Ng HP, Patel N, Mahabeleshwar GH. Kruppel-like factor 6 and miR-223 signaling axis regulates macrophage-mediated inflammation. FASEB J 2019;33:10902-10915.
54. Calvente CJ, Tameda M, Johnson CD, Del Pilar H, Lin YC, Adronikou N, De Mollerat Du Jeu X, et al. Neutrophils contribute to spontaneous resolution of liver inflammation and fibrosis via microRNA-223. J Clin Invest 2019;129:4091-4109.
55. Tang S, Zhang X, Duan Z, Xu M, Kong M, Zheng S, Bai L, et al. The novel hepatoprotective mechanisms of silibinin-phospholipid complex against d-GalN/LPS-induced acute liver injury. Int Immunopharmacol 2023;116:109808.
56. Lu FB, Chen DZ, Chen L, Hu ED, Wu JL, Li H, Gong YW, et al. Attenuation of Experimental Autoimmune Hepatitis in Mice with Bone Mesenchymal Stem Cell-Derived Exosomes Carrying MicroRNA-223-3p. Mol Cells 2019;42:906-918.
57. Johnnidis JB, Harris MH, Wheeler RT, Stehling-Sun S, Lam MH, Kirak O, Brummelkamp TR, et al. Regulation of progenitor cell proliferation and granulocyte function by microRNA-223. Nature 2008;451:1125-1129.
58. Fazi F, Rosa A, Fatica A, Gelmetti V, De Marchis ML, Nervi C, Bozzoni I. A minicircuitry comprised of microRNA-223 and transcription factors NFI-A and C/EBPalpha regulates human granulopoiesis. Cell 2005;123:819-831.
59. Ichiyama K, Gonzalez-Martin A, Kim BS, Jin HY, Jin W, Xu W, Sabouri-Ghomi M, et al. The MicroRNA-183-96-182 Cluster Promotes T Helper 17 Cell Pathogenicity by Negatively Regulating Transcription Factor Foxo1 Expression. Immunity 2016;44:1284-1298.

Table S7. miRNAs present only in IA and/or ENH phases.

miRNA	Phase(s) present
hsa-miR-3679-3p	IA
hsa-miR-663a	IA
hsa-miR-96-5p	IA
hsa-miR-328-5p	IA
hsa-miR-6845-5p	IA
hsa-miR-6510-5p	IA
hsa-miR-664a-5p	IA
hsa-miR-6516-3p	IA
hsa-miR-551b-3p	IA
hsa-miR-6075	IA
hsa-miR-135b-5p	IA
hsa-miR-424-3p	IA
hsa-miR-452-5p	IA
hsa-miR-4697-5p	ENH
hsa-miR-1271-5p	ENH
hsa-miR-3620-5p	ENH
hsa-miR-6798-3p	ENH
hsa-miR-1227-3p	ENH
hsa-miR-4695-5p	ENH
hsa-miR-4317	ENH
hsa-miR-6756-3p	ENH
hsa-miR-572	ENH
hsa-miR-4633-5p	ENH
hsa-miR-23b-5p	ENH
hsa-miR-5010-3p	IA and ENH
hsa-miR-7-1-3p	IA and ENH
hsa-miR-1288-3p	IA and ENH
hsa-miR-7-5p	IA and ENH
hsa-miR-342-5p	IA and ENH
hsa-miR-6512-5p	IA and ENH
hsa-miR-542-5p	IA and ENH
hsa-miR-5699-5p	IA and ENH
hsa-miR-6507-3p	IA and ENH
hsa-miR-30c-1-3p	IA and ENH
hsa-miR-3156-5p	IA and ENH
hsa-miR-409-3p	IA and ENH
hsa-miR-4646-3p	IA and ENH
hsa-miR-4767	IA and ENH
hsa-miR-150-3p	IA and ENH
hsa-miR-21-3p	IA and ENH
hsa-miR-181a-2-3p	IA and ENH
hsa-miR-6759-3p	IA and ENH
hsa-miR-7111-3p	IA and ENH
hsa-miR-1307-5p	IA and ENH
hsa-miR-6807-5p	IA and ENH
hsa-miR-6867-5p	IA and ENH
hsa-miR-181c-5p	IA and ENH
hsa-miR-34a-3p	IA and ENH
hsa-miR-30c-2-3p	IA and ENH
hsa-miR-6728-5p	IA and ENH
hsa-miR-4793-5p	IA and ENH
hsa-miR-6789-5p	IA and ENH
hsa-miR-4312	IA and ENH
hsa-miR-3648	IA and ENH

hsa-miR-4651	IA and ENH
hsa-miR-8060	IA and ENH
hsa-miR-6780a-5p	IA and ENH
hsa-miR-379-5p	IA and ENH
hsa-miR-500a-5p	IA and ENH
hsa-miR-625-5p	IA and ENH
hsa-miR-665	IA and ENH
hsa-miR-511-3p	IA and ENH
hsa-miR-7152-3p	IA and ENH
hsa-miR-3652	IA and ENH
hsa-miR-6086	IA and ENH
hsa-miR-423-3p	IA and ENH
hsa-miR-503-5p	IA and ENH

This table lists miRNAs detected exclusively in IA, exclusively in ENH, or in both IA and ENH, and not detected in IT or IC phases.

Abbreviations: IA, immune active; ENH, HBeAg-negative active chronic hepatitis.

Table S8. Differential expression of 39 hepatic cell type–enriched miRNAs across CHB phases.

Cell	miRNA	IA vs. IT		IA vs. IC		ENH vs. IT		ENH vs. IC	
		adj. p	log_2FC	adj. p	log_2FC	adj. p	log_2FC	adj. p	log_2FC
PHH	hsa-miR-4800-5p			0.029	0.54				
PHH	hsa-miR-193b-3p								
PHH	hsa-miR-192-3p								
PHH	hsa-miR-23b-3p			0.001	-0.42				
PHH	hsa-miR-122-3p	0.035	-0.32	0.001	-0.47				
PHH	hsa-miR-192-5p			0.004	-0.30				
PHH	hsa-miR-215-5p			0.003	-0.33				
PHH	hsa-miR-194-5p	0.035	-0.28	0.001	-0.40				
PHH	hsa-miR-885-5p	0.035	-0.48	0.001	-0.62				
PHH	hsa-miR-148a-3p			0.004	-0.55				
PHH	hsa-miR-375								
PHH	hsa-miR-122-5p								
KC	hsa-miR-660-5p	0.032	0.41	0.007	0.41			0.027	0.34
KC	hsa-miR-362-3p			0.007	0.38			0.009	0.39
KC	hsa-miR-21-5p	0.002	0.89	0.001	0.71	0.039	0.65	0.008	0.48
KC	hsa-miR-155-5p	0.002	1.66	0.000	1.62	0.039	1.13	0.008	1.08
KC	hsa-miR-142-5p	0.002	1.09	0.000	1.04	0.043	0.80	0.013	0.75
KC	hsa-miR-340-5p								
KC	hsa-miR-15a-5p								
KC	hsa-miR-185-5p			0.021	0.45			0.031	0.40
KC	hsa-miR-4306			0.015	0.42	0.043	0.42	0.008	0.44
HSC	hsa-miR-136-5p			0.029	0.35				
HSC	hsa-miR-127-3p			0.000	0.71			0.008	0.52
HSC	hsa-miR-495-3p			0.000	0.55			0.003	0.36
HSC	hsa-miR-654-3p			0.041	0.27			0.016	0.22
HSC	hsa-miR-299-5p			0.008	0.38			0.011	0.22
HSC	hsa-miR-337-5p			0.001	0.50			0.013	0.30
HSC	hsa-miR-130a-3p								
HSC	hsa-miR-503-5p	0.038	0.48	0.021	0.48				
HSC	hsa-miR-199a-3p					0.039	0.54		
HSC	hsa-miR-214-3p					0.039	0.60		
HSC	hsa-miR-10a-5p					0.043	0.49		
HSC	hsa-miR-145-3p							0.042	0.33
LSEC	hsa-miR-6089								
LSEC	hsa-miR-6869-5p								
LSEC	hsa-miR-6821-5p								
LSEC	hsa-miR-6090								
LSEC	hsa-miR-222-3p	0.006	0.76	0.000	0.81			0.008	0.44
LSEC	hsa-miR-126-3p								

Differential expression was assessed by Mann–Whitney U test with Benjamini–Hochberg FDR correction (adjusted $p < 0.05$). Columns show FDR-adjusted p-values and log_2FC; blank cells indicate non-significant comparisons (adjusted $p \geqslant 0.05$). The miRNA order matches that of the corresponding heatmap (see Fig. 4B).

Abbreviations: CHB, chronic hepatitis B; IT, immune tolerance; IA, immune active; IC, inactive carrier state; ENH, HBeAg-negative active chronic hepatitis; FDR, false discovery rate; FC, fold change.

Table S9. Normalized expression values of 559 hepatic miRNAs in liver tissue from chronic hepatitis B patients (IT and IA phases).

Samples	IT_1	IT_2	IT_3	IT_4	IT_5	IT_6	IT_7	IT_8	IT_9	IT_10	IA_1	IA_2	IA_3	IA_4	IA_5	IA_6	IA_7	IA_8	IA_9	IA_10
GEO_ID	IT-TXX_S20	IT-WJF_S1	IT-CMJ_S9	IT-MYC_S19	IT-CHS_S3	IT-HZP_S4	IT-ZKQ_S8	IT-LZ_S5	IT-ZC_S21	IT-LDW_S6	IA-XHK_S15	IA-BRG_S11	IA-HXL_S16	IA-ZWX_S13	IA-QYC_S15	IA-ZJL_S6	IA-WF_S7	IA-QBS_S16	IA-WHJ2_S17	IA-SYB_S18
hsa-let-7a-5p	12.75210111	12.82881819	12.41772948	12.83535215	12.58799918	12.77611026	12.93244835	13.20440231	12.20423909	11.56710425	13.07817938	12.88578597	13.12691608	12.9598486	12.48689113	12.83057293	12.65821471	12.55177276	13.19433182	13.17290753
hsa-let-7b-3p	4.162048253	4.269713152	4.093141435	3.945458307	4.34921528	3.463000795	4.305135584	4.118595664	3.12088228	4.242519116	4.086791739	3.969205495	4.079149909	4.009820573	3.726652681	3.793930473	4.158800297	3.733608144	4.244920084	3.901931733
hsa-let-7b-5p	11.88808617	12.01712227	11.60982447	11.84189482	11.68879701	11.80381112	12.04401425	11.89138322	11.60585163	11.82069279	12.3623124	11.93918696	12.20252185	11.99587199	11.66344627	11.94267628	11.84078929	11.67981431	12.32661501	12.08752244
hsa-let-7c-5p	10.92594337	11.11236951	10.82898955	10.8809962	10.97057371	10.91158171	10.94172659	11.04542717	10.49751429	10.69096911	10.93347549	10.89836037	10.93009652	10.88116285	10.55447454	10.82030843	10.75913186	10.75312215	11.00468299	11.05628388
hsa-let-7d-5p	8.974649666	9.203935717	8.864502237	8.9819625	9.117115164	8.881636636	9.13972474	9.183935496	7.759007544	9.104754005	9.425584755	9.081935691	9.204565544	8.994552201	9.055777423	9.106685699	9.318703087	8.891535155	9.165217072	9.852677431
hsa-let-7e-5p	7.957991598	7.999781228	7.525424332	7.792533266	7.764117544	7.723653603	8.286042513	8.049743037	6.69859992	6.494123922	8.953294439	8.042537173	8.472680122	8.029358699	7.72348341	8.304386938	8.15564705	7.809477992	8.541430714	9.309715415
hsa-let-7f-1-3p	3.798990412	4.080644196	3.8341654	3.712132961	4.260098305	3.472680831	4.264601281	4.197162066	3.040373557	4.215394882	3.882359592	3.85713546	4.108442442	3.9124124	3.588269893	3.808188806	3.943213269	3.608632848	4.246350066	3.75881002
hsa-let-7f-5p	12.08564844	12.19221811	11.5512808	12.18046198	11.90199473	12.25773805	12.16924805	12.19532171	11.40555214	9.815555084	12.47127144	12.27212288	12.25787501	12.17526445	12.00842634	12.20716294	12.13794816	12.0524721	12.33422879	12.52927597
hsa-let-7g-5p	11.24591487	11.24696573	10.8263048	11.20605516	11.01689243	11.24063172	11.16549322	11.22387386	10.54858998	8.128827561	11.4169957	11.31698595	11.33561714	11.26482184	11.06041425	11.41196008	11.19939487	11.19090553	11.322234	10.90498854
hsa-let-7i-5p	9.54695878	9.481671996	9.269429079	9.539523509	9.33949318	9.474791672	9.660469916	9.482933121	8.998938495	7.802480384	9.978653107	9.880555786	9.746692961	9.586815298	9.648553996	9.762885226	9.867847358	9.522080342	9.746130144	10.66362736
hsa-miR-100-5p	9.428196393	9.371373892	9.282633772	9.100884222	9.495748385	9.188659165	9.488490532	9.810933778	8.68918834	8.112722815	9.413852732	9.663109627	9.622945046	9.521448588	9.176727211	9.657567059	9.336997744	9.299818729	9.515943443	9.819100242
hsa-miR-101-3p	9.141982966	9.246730899	9.09036177	9.196324227	9.527061935	9.140706895	9.566908279	9.452056259	8.003276836	7.698398728	8.066514327	8.803326779	8.849053012	8.928488179	8.579667363	8.629397779	8.285337135	9.255424524	8.837962544	9.555972761
hsa-miR-103a-3p	9.636939062	10.0433484	9.846101115	9.908485143	9.715197758	9.86649306	10.01925034	10.19271074	9.112008031	9.187857307	10.15186601	10.27302795	10.06056629	10.04245162	9.834157374	10.07741724	9.852871635	9.729310258	10.10747962	8.836756137
hsa-miR-106b-5p	7.99247628	8.143472335	8.04274714	7.974021107	8.048013805	7.843724179	8.277334613	8.273691815	6.673483577	7.937921379	8.462417625	8.465748287	8.540411727	7.975808621	8.004970374	8.271855107	8.423449071	7.994698973	8.222640222	7.936850331
hsa-miR-107	9.826926359	10.01952423	9.825660782	10.01864815	9.81560614	9.90381899	10.00859508	10.14816445	9.042646809	9.421140922	9.907692145	10.14653749	10.01566424	9.871807167	9.643237598	9.959886283	9.799468672	9.689388442	10.03007503	8.880876544
hsa-miR-10a-5p	7.412486026	7.593780168	6.961576693	7.47133945	7.311698926	7.027178702	7.81614492	7.624564924	6.720649227	4.695313868	7.918898307	7.464109474	8.021908368	7.565261804	7.302071481	7.592283777	7.538654029	7.325783191	7.95976911	8.582810119
hsa-miR-10b-5p	4.736752366	5.03042062	3.959720012	5.496558445	5.317734691	4.164130606	5.393135859	4.893568231	3.766248019	3.050186322	5.345941823	4.71063764	5.541266027	4.953423352	6.223395391	5.268024203	6.366403871	4.569770604	5.26510017	8.967379632
hsa-miR-1181	3.9309612	4.394823831	5.609793286	4.079300013	3.685894805	4.272571246	3.992637942	3.961941084	3.301595937	4.979334473	4.569819387	4.23194826	4.37881101	4.774816178	5.34358801	5.246350568	4.636905732	5.508349211	4.292459074	3.428625121
hsa-miR-1185-1-3p	3.523316068	3.344965	4.679973748	3.33538296	3.16514244	3.848136295	3.238762314	3.388926232	3.887766038	3.949499093	3.678643156	3.514837673	3.345449754	3.887578322	4.409716702	4.323661753	3.675041937	4.713371362	3.390052803	3.634639322
hsa-miR-1202	7.305964598	7.212013695	7.112412921	7.024902031	7.015975021	7.408616975	6.945741162	7.172023074	7.889138284	7.490703112	6.778628268	7.442885475	6.874729925	7.301946667	6.844620186	6.861217659	6.817312443	6.858631394	7.034631295	6.278229548
hsa-miR-1207-5p	6.542641292	6.815736511	6.022146626	6.237283638	6.328196354	6.747924869	6.893214753	6.788302028	7.057114386	7.100971124	5.964827928	6.146690122	5.840660815	6.021411345	6.11628351	5.617435918	6.002311525	6.317386501	5.853967786	5.601684527
hsa-miR-122-3p	11.02165043	11.18718613	11.23104413	10.95608659	11.17504575	11.21886652	11.14517545	11.39262684	10.26388228	11.1286412	10.18647774	10.87834176	10.86919103	11.12048487	10.32209413	10.92682233	10.39096665	11.05513033	10.94016024	6.372472388
hsa-miR-1224-5p	4.663290007	4.769472441	4.561400009	4.061064718	3.974769565	4.658149044	4.164911499	4.484641335	4.890031202	5.583662831	3.881686429	4.056957203	3.932794168	4.319905214	4.410856664	4.277339745	3.834665229	4.477771563	4.22441824	3.582514018
hsa-miR-1225-5p	6.964784753	7.191729301	7.160312014	6.479224456	6.518257742	6.97816412	6.913539614	6.94539787	6.555251537	7.6916664	6.295687348	6.657409299	6.401082756	6.673352975	6.848608766	6.668843668	6.350257385	7.090964947	6.560238918	5.160961724
hsa-miR-122-5p	15.54077635	15.54014588	15.61329182	15.60675495	15.58953431	15.76505522	15.61329182	15.61329182	15.28575159	13.2839983	15.00308734	15.34243705	15.58953431	15.61329182	15.4971752	15.61329182	15.18866238	15.34243705	15.61329182	10.10569217
hsa-miR-1227-3p	3.098979218	3.039236943	3.170387745	2.970726631	3.106575042	3.011228462	3.543770182	2.971108559	2.799909921	3.17417102	3.247162535	3.117470824	3.099164973	3.149137645	3.029445867	3.075507785	3.141664651	2.972398966	3.419788648	3.015421525
hsa-miR-1227-5p	3.623063674	4.153805525	3.593471763	3.658906023	3.295510657	3.794069313	5.486302398	3.787691373	4.232928911	4.428025581	2.998155967	3.060569998	2.98636453	3.343752339	3.78022097	3.050567561	3.45144234	4.302115139	3.128331913	4.420361643
hsa-miR-1228-3p	4.806955189	5.249073192	4.858384471	4.579555037	5.128946936	4.348922201	5.234317058	5.198800387	4.095881579	5.375646586	4.797637924	4.795516647	4.813807119	4.785680113	4.461323685	4.55554125	4.787059183	4.704510062	5.066773434	4.404140284
hsa-miR-1229-3p	3.397414157	3.381072075	3.424708056	3.316040675	3.643365809	3.144910699	3.427849107	3.540197763	2.916512487	3.678172	3.380161332	3.262371097	3.262486405	3.424335836	3.347736249	3.401564987	3.313508645	3.382440097	3.3866806	3.322789243
hsa-miR-1229-5p	4.3953572	3.981063761	4.407211641	3.572237319	4.215122012	5.552438402	3.94339402	3.849480937	5.905292535	4.070643899	4.033882476	4.202145274	3.973013886	4.792081287	4.591710614	4.17849423	4.015167583	4.035576739	4.042059688	5.094819494
hsa-miR-1233-5p	4.193830268	3.77733059	4.208215336	3.977045832	3.493569446	4.18839626	3.367701438	4.049148818	3.478477719	5.542339157	3.761859164	4.036926086	4.156808916	4.147134827	3.899794307	3.982436837	4.015962595	4.434891445	3.858702071	3.904389933
hsa-miR-1234-3p	4.823466659	5.056211513	4.806484021	4.529044192	5.138300328	4.753964774	4.974023742	4.910326579	3.759685867	5.234169494	4.721411409	4.780044111	4.782164091	4.852270088	4.536532149	4.604451154	4.660059153	4.647580951	4.984642308	4.452952054
hsa-miR-1237-3p	3.701039783	3.888273498	3.802192411	3.628235696	4.122183489	3.359830075	4.095669597	3.713439699	3.106081701	4.013526221	3.861944631	3.853447217	3.716980126	3.720621355	3.447511705	3.61127229	3.619038881	3.533462799	4.048515657	3.587408832
hsa-miR-1238-3p	4.382514929	4.809418783	4.33440342	4.289279911	5.027833538	3.906849383	4.897979628	4.744914104	3.367875374	4.806006161	4.592262308	4.48849463	4.506595221	4.594598589	4.186528841	4.217681543	4.441808319	4.221044887	4.841448554	4.194491443
hsa-miR-1246	4.887414944	5.337633262	5.398216967	4.652235314	6.178943186	4.84023656	4.801491734	4.681749077	7.573008065	4.957537183	4.947941859	5.35009405	4.704642658	4.671867505	5.355925242	5.063397259	5.000503466	5.852774163	4.75902466	4.72868826
hsa-miR-1249-3p	4.883634687	5.449683231	5.096342966	4.626251271	4.844074431	4.668837528	5.362219597	5.285097787	5.017544064	5.744209802	4.490390759	4.753059951	4.598728948	4.87189611	4.605679189	4.686729538	4.769447681	5.1202319	4.797478421	3.975564152
hsa-miR-1249-5p	3.169928224	3.377855513	4.174731506	3.225584349	3.016424487	3.37068469	3.211979599	3.227613318	3.239545502	4.329376455	3.415485191	3.644536642	3.424628179	3.888808156	3.989716752	3.991165073	3.583419349	3.932259992	3.521846106	2.634037077
hsa-miR-125a-3p	4.605073606	4.243480397	5.27545999	4.023830483	3.45222473	4.953295741	3.971531148	4.031404696	8.792768805	4.831909099	4.638204984	4.990472348	4.377375904	5.421442474	5.392047693	4.997764928	4.424877372	5.027465734	4.279021459	3.609681208
hsa-miR-125a-5p	5.963044826	5.892404328	5.312063893	5.713680826	5.679048077	5.604495853	6.302556249	6.073611284	4.697864732	4.33168958	6.964069171	6.149851097	6.571474225	6.058612784	5.656775304	6.461556899	6.294890579	5.470756525	6.584807812	7.168741132
hsa-miR-125b-2-3p	3.803953473	4.084341063	3.802723872	3.827906445	3.832714506	3.647553236	4.083948358	4.346039147	3.415617671	3.359565382	3.748875351	3.934946785	4.068630387	3.880649456	3.547087832	3.754172796	4.057619533	3.567736243	3.600333599	4.657751605
hsa-miR-125b-5p	10.6586704	10.83596507	10.81665817	10.6562722	10.79304785	10.70539028	11.07080496	11.04787932	10.12302087	9.47817875	10.19825281	10.64251707	10.65267336	10.64823157	10.28064824	10.60142188	10.34684001	10.55098388	10.66371619	11.7412891
hsa-miR-1260a	10.93997019	11.2141782	10.81643913	10.87171287	10.80235024	11.18955902	10.87766088	10.98232742	8.243135362	11.72585745	10.6315943	10.73583771	10.36817943	10.73893354	9.8851352	10.52365503	10.64618759	10.97051055	10.65683904	10.15768949
hsa-miR-1260b	9.460581739	9.672018729	9.323588509	9.289562207	9.269543768	9.022784098	9.096598463	9.255941426	7.39279233	10.28002367	9.359900224	9.093484283	8.79030001	9.000560044	8.340137072	8.876854951	9.32371914	9.305098445	9.026496164	9.081101384
hsa-miR-126-3p	11.0554921	11.06492948	10.73595329	10.80949662	10.74695814	11.15390337	11.18299281	10.99641472	10.46548925	10.93480282	10.95228642	11.06910635	10.97702167	10.8900278	10.55807451	10.82517736	10.75160816	10.87950396	10.94300037	11.100509
hsa-miR-126-5p	6.055570229	5.8733167	4.962695986	5.836773963	5.83969576	5.786714961	5.91553072	6.058636279	4.707885292	5.72555298	5.490069243	5.834452551	5.974568799	5.707570653	5.333582874	5.55567075	5.6613797	5.406181985	5.729446727	6.57862066
hsa-miR-1268a	4.912357009	5.786667652	4.815328858	5.155394134	5.539555301	5.303001978	6.82153302	5.511659737	6.063435644	5.817529511	4.709606627	4.679052347	4.557300796	4.76505238	5.178882846	4.536230268	4.76800862	5.164728529	4.328982619	4.963919854
hsa-miR-1268b	4.974707511	5.031139842	4.94849954	4.732856119	4.809137085	4.847157891	5.304294149	4.920525049	7.417813917	5.340058703	4.66843686	4.809194276	4.596447512	5.06910403	4.785526861	4.70126109	4.761630944	4.986301334	4.755459047	4.924061743
hsa-miR-1271-5p	3.092608818	3.088320816	3.029910922	3.252526977	3.10868695	3.017928038	3.21031326	3.301299281	2.787138838	3.246209413	3.175195007	3.0978852	3.459139183	3.021946891	3.634835217	3.201177345	3.830570572	3.170682538	3.277830099	5.377944379
hsa-miR-1273e	3.213615486	3.710629867	5.073656097	3.355844078	2.971346923	4.277984895	3.164550831	3.250737828	3.826173287	3.654399057	3.654399057	3.702019656	3.787774051	4.055675569	4.697152963	4.673997536	3.773378	4.716505373	3.678760092	2.663222582
hsa-miR-1273f	3.106561482	2.820210742	3.645062727	3.057176061	2.93546599	3.862838863	2.802822685	2.726395057	5.463734205	4.222808885	3.194370972	3.109020002	2.953960208	3.606190872	3.900407855	3.692321816	2.51078654	3.67507253	2.984234266	3.061602633
hsa-miR-1273g-3p	10.52053513	10.75165806	11.3825344	10.73112704	10.68280927	11.04018087	10.67127705	10.47228908	10.42212529	11.25030819	10.85305965	10.6879848	10.51674657	11.18777606	10.72438774	10.78975409	11.25581667	11.04681648	10.70327367	10.20002723
hsa-miR-127-3p	4.219170808	4.621714398	3.921984694	3.965268143	3.996255213	3.990979548	4.068575431	4.716890479	3.0783203	3.604534046	5.42563806	4.354537683	4.484192578	4.311347353	4.093033469	4.209693013	4.731292712	4.001129382	4.359344718	5.038895952
hsa-miR-1275	4.64185547	5.034636863	4.892805753	4.636361446	4.825266675	4.365384047	4.957598192	4.988518147	6.273869145	5.333594878	5.034529508	4.992550258	5.050699927	4.644038709	4.679485953	4.962636795	4.591044244	4.848760455	5.094585959	4.033069698
hsa-miR-1281	4.375745882	4.450302399	4.176901302	4.154583618	4.738147125	3.854049017	4.577376753	4.572792044	3.434683296	4.596608943	4.291744263	4.372472776	4.458756098	4.204974743	4.040389319	4.13952125	4.19181429	4.107623521	4.6098975	4.234907878
hsa-miR-128-3p	4.972235684	5.310659572	4.382206179	5.280192854	5.370492494	4.739613438	5.137239623	5.269765028	3.734440387	3.494685649	5.913490194	5.518086302	5.806445057	5.05114191	5.840256783	5.772025541	5.95357756	4.505182006	5.409972674	7.068636848
hsa-miR-1288-3p	3.54467098	3.067246607	3.932058305	3.675080123	3.526344835	3.58050206	3.383837361	3.502274369	3.150958668	3.361047649	3.797665037	3.76461829	3.899313477	3.717120977	3.891444146	3.988695711	4.01537956	3.974890243	3.823932931	3.650154028
hsa-miR-1290	3.690912787	3.378027607	3.820462633	3.334395812	3.32182153	3.26417564	3.53730915	3.660589859	4.329120399	3.311876759	3.631567326	3.911558779	3.670567119	3.667509639	3.756199885	3.692448475	3.628991898	3.804958542	3.331132166	3.350790814
hsa-miR-129-1-3p	3.228780633	3.412141718	3.361831194	3.313461807	3.520224085	2.964868038	3.417908943	3.227342529	2.888768585	3.470637877	3.473881152	3.340757582	3.436900553	3.474569406	3.139914841	3.387552496	3.442226722	3.026925291	3.500430137	3.439493871
hsa-miR-129-2-3p	3.435999031	3.511134553	3.422774425	3.3070947	3.435557294	3.00402775	3.58161233	3.593695796	2.910518496	3.481924507	3.374500183	3.529129403	3.261629527	3.471807113	3.306399331	3.302878608	3.522602686	3.160166535	3.57586687	3.463380229
hsa-miR-1304-3p	4.409453964	4.67511156	4.545175084	4.353369542	4.788177858	4.047552626	4.852461566	4.658855181	3.430759123	4.853703648	4.575333479	4.481436831	4.615818106	4.481642865	4.13192483	4.206116675	4.413671931	4.247031425	4.811594721	4.222002024
hsa-miR-1305	5.109501229	4.622129013	5.12595789	5.098475804	4.810842135	4.936253573	4.565086263	4.730511809	4.540799413	4.313009288	5.160940864	5.131739823	5.191530601	4.976611153	4.981142977	5.21316642	4.906594078	5.245985676	4.977717446	4.636280967
hsa-miR-1307-5p	3.154351571	3.043120668	3.482259184	3.194699099	3.358492558	2.98747445	3.176262052	3.286451978	2.782913669	3.00426199	3.328129027	3.407621439	2.921770834	3.062822336	3.389193216	3.385701285	3.242136708	3.553564413	3.364258849	3.270777208
hsa-miR-130a-3p	8.850748601	8.846564736	8.848112467	8.725001964	8.79848266	8.69919764	9.379811294	8.926762291	8.054460425	9.388898368	9.397953101	8.830133781	9.152271383	8.819789995	8.538494439	8.948526464	9.10085603	8.70513558	9.083833291	9.393945116
hsa-miR-130b-3p	4.680793981	5.886213475	4.696693477	5.505424165	5.118283174	5.086105238	5.663136965	5.545542367	4.020659454	3.832014874	5.717713874	5.638458028	5.658919803	4.989213684	4.580209728	5.793435997	5.482288215	4.905324915	5.842017231	4.386677382
hsa-miR-132-3p	3.581179325	3.730876138	3.250857688	3.574915645	3.428632292	3.269574352	3.937898025	4.024289707	3.020772634	3.404759761	4.875540008	4.6246999	4.719542077	4.142872782	4.296755738	4.36462222	4.47608206	3.564787549	4.232865012	4.5988073
hsa-miR-133b	3.715859116	3.984102788	2.880757205	10.90891659	11.65545605	2.822453799	4.201501941	3.138224311	3.157187524	3.511159934	3.239142671	2.866414994	4.507992825	3.316785846	12.74335136	3.498796611	12.20894111	2.849371761	3.428305535	15.57932133

hsa-miR-135a-3p	3.189550748	3.443628347	4.622906717	3.377601321	3.15245327	3.499021377	3.050728074	3.345100642	3.47984524	4.084205636	3.780525163	3.710824357	3.514464909	3.9245444	4.406568574	4.383963297	4.041574286	4.361939558	3.401360324	3.118147008
hsa-miR-135b-5p	3.217528421	3.34092319	2.923894315	3.087024918	3.625918775	3.054824767	3.124715235	3.292863474	2.617929053	2.87804234	3.413676979	3.754198949	3.719335551	3.326867489	3.0573948	3.807368593	3.160258779	3.118983626	3.558251127	4.422674023
hsa-miR-136-3p	3.696141095	3.561876409	3.231093764	3.407305462	3.86002773	3.245814339	3.733575003	3.837241404	2.766337586	3.53970277	4.163418825	3.72969676	3.76008658	3.575150781	3.429302781	3.494049429	3.773958952	3.429188884	3.661728168	4.674510862
hsa-miR-136-5p	4.474909794	4.252537286	3.855039853	4.212253673	5.009760609	3.988222123	4.887425952	4.489937917	2.991589375	3.133805699	5.050311026	4.501934442	4.502971247	4.179965591	3.953566649	4.225139109	4.502819043	4.233718399	4.035433753	5.675822163
hsa-miR-139-3p	4.172130649	4.174708153	3.872721184	4.223992141	4.167231646	3.935946331	4.279586869	4.066436991	3.661571836	4.183599357	3.973289509	4.197479374	4.162072148	4.10402518	3.730779343	4.146878337	3.969015712	3.887818013	4.167075586	3.732964003
hsa-miR-139-5p	3.965271765	4.213391493	3.768739537	3.936607584	4.019253507	3.899358533	4.188740552	4.300076642	3.257794959	3.608328139	3.868768934	4.023345574	4.135105498	4.090777202	3.687096184	3.83493645	4.201078304	3.907919172	4.049176375	4.210875652
hsa-miR-1-3p	4.037841891	4.791786569	2.61457758	11.65005476	12.17046566	2.935491052	4.982740964	3.432490379	3.642134356	2.471378963	3.593313619	3.157853868	5.100819142	3.87541541	13.82139338	4.007427945	12.73221701	3.144827465	3.912828688	15.95843556
hsa-miR-140-3p	6.409860852	6.327657429	6.081360888	6.322816504	6.335136079	6.032126564	6.899863428	6.503693836	4.903042496	5.976657074	6.66542697	6.842645123	6.65589495	6.456054615	6.474425944	6.634529024	6.72631157	6.053619156	6.53393834	7.697668986
hsa-miR-140-5p	6.561161888	6.339661382	5.802615092	6.371329887	6.339313571	6.220596308	6.911123332	6.631460116	4.903885621	3.405138503	6.67185209	6.825009799	6.787770034	6.396513295	6.3874795	6.589909306	6.597476882	6.071519389	6.671542371	7.882099989
hsa-miR-142-3p	9.908977346	9.400790213	9.310687031	9.615600801	9.345207916	9.551762519	10.14106955	9.345241929	8.458816401	6.966890442	10.66247511	10.72253177	10.24244122	9.876435968	10.47333094	10.33969638	10.21986735	10.35432845	9.837977796	9.285333974
hsa-miR-142-5p	6.777308453	6.148392607	5.821086307	6.585707012	6.186096332	6.340546342	7.047988277	6.216467802	5.237086716	4.16731242	7.456547095	7.657455057	7.430028779	7.007746354	7.349320032	7.453778023	7.272159838	7.21876161	6.870887385	6.369934145
hsa-miR-143-3p	5.640314478	5.445779562	4.657295499	4.997371064	5.116456389	4.668132782	6.482671673	5.125481351	4.376889984	5.015154895	5.782965596	5.131801478	5.958304011	5.243348968	5.274671022	5.747070823	5.648320994	4.887018496	5.731433697	7.854746441
hsa-miR-144-3p	7.581558682	7.517375741	7.535305121	7.790654691	8.433826407	6.853254214	7.072186563	6.745280531	4.70100379	6.542797116	6.344031373	6.615058516	7.060215487	6.281967928	6.44006494	5.341005402	7.277009111	6.076315663	6.434624051	9.42184107
hsa-miR-144-5p	4.386204082	4.369772541	3.992166571	4.270302064	4.570689368	3.905275493	3.464760774	3.950664237	2.930171348	3.344821169	3.168009569	3.479681809	3.828437306	3.511237065	3.7714057	3.017834853	3.995585127	3.285327293	3.665249867	5.437547996
hsa-miR-145-3p	4.024248114	3.560362361	2.962670728	3.609441751	3.408686928	3.076136769	4.51977127	3.764939218	2.944370083	3.089466858	3.977870683	3.423899486	4.038324111	3.706262897	3.460424221	3.778321726	3.82935691	3.302956123	3.973475476	5.980081795
hsa-miR-145-5p	8.909355385	8.587875134	7.815126904	8.210224043	8.306792897	8.037830373	9.397697644	8.371310798	7.917540058	9.017194455	8.903759064	8.040650151	8.861860937	8.39220116	8.088288653	8.665139477	8.371792047	8.134135345	8.95055845	10.78728122
hsa-miR-146a-5p	6.646143746	6.400494498	6.357729615	6.555708563	6.170050195	6.42616854	6.963272542	6.76297453	5.335438011	5.476801765	7.489341676	7.747536235	7.444438582	7.05831277	7.095480472	7.59798204	7.778056898	6.986405783	6.952285556	5.536065637
hsa-miR-146b-5p	7.347575611	7.52110675	6.933722823	7.176758922	6.948604415	7.218642825	7.338952566	7.175935745	5.319736668	5.557081943	8.669009864	8.72082843	8.383875486	7.950608663	8.086823682	8.091541524	8.44000452	6.831765862	8.118880372	5.761153269
hsa-miR-148a-3p	11.70628371	11.49407629	11.41680065	11.54524814	11.39412536	11.6051243	11.66146213	11.38824544	10.96800278	11.66007142	10.2217815	11.12044701	11.20923699	11.62704334	10.60604809	11.22753182	10.17914022	11.54162979	11.46409611	8.789013281
hsa-miR-148a-5p	2.882190975	2.850892848	2.702320415	2.753557979	2.850481723	2.586375814	2.747821957	2.941117123	2.6568045	2.722733349	2.780226516	2.600853981	2.74675917	2.799399818	2.630373938	2.697312189	2.557846941	2.607005889	2.783696803	2.627563453
hsa-miR-148b-3p	5.416214284	5.478988595	4.972951575	5.343123976	5.333267251	5.114537999	5.511226904	5.653865423	4.133468917	5.050721325	5.497210534	5.59025408	5.764750784	5.548629529	5.002267333	5.599241169	5.468612841	5.018915636	5.573541912	5.636479238
hsa-miR-149-5p	3.76694839	4.059909117	3.923959772	3.849975413	4.324559175	3.307438353	4.468076489	4.306080236	3.13959602	4.402848627	4.205496837	4.004768362	4.144695424	4.046973419	3.760930454	3.822128096	4.118772129	3.669370702	4.232353582	4.498331457
hsa-miR-150-3p	3.230191033	3.260720202	3.954612668	2.885290018	3.220030993	3.671309581	3.097317422	3.148375047	3.903698369	4.496833246	3.40359431	3.368522147	3.049928893	3.937700517	3.885153003	3.966408996	3.242534774	3.702651472	3.072217867	3.282181085
hsa-miR-150-5p	8.183984545	7.731311549	7.133444094	7.867142906	7.523764693	7.829836215	8.553132799	8.005610097	7.855837035	6.964353676	9.234106135	9.512582461	8.825872167	8.698162243	8.341713904	9.230923517	8.462456922	8.871730515	8.886946805	8.370754507
hsa-miR-151a-3p	5.328289849	5.435706945	4.947049926	5.175190726	5.199816718	5.129762087	5.723489619	5.708891151	3.814974737	4.204278142	5.609743994	5.39658537	5.70496192	5.380941293	4.87615067	5.441975743	5.628605331	4.980497375	5.685972598	5.5229025
hsa-miR-151a-5p	8.042956621	8.349850756	8.419932994	8.048316114	8.13668403	8.115930149	8.438977544	8.513246857	6.998231763	8.377793747	7.962660485	8.019902958	8.191641151	8.160886926	7.654421086	8.008009619	7.935467671	8.150228031	8.338290206	8.213427079
hsa-miR-151b	6.368366483	6.593536833	6.293761913	6.216573824	6.430904414	6.334292532	6.598151009	6.831940002	5.421315225	4.582080008	6.24492139	6.296470745	6.557863282	6.471925708	5.95898468	6.37091842	6.383869583	6.348054725	6.604057794	6.73456109
hsa-miR-152-3p	5.032670314	5.385463543	4.517252022	5.204289166	5.314628169	5.06738422	5.181474792	5.49795033	4.226284117	4.270670687	5.069982492	5.236775093	5.416014167	5.183592443	4.885025945	5.192027936	5.135433814	4.744110588	5.130370366	5.458310308
hsa-miR-1539	4.011584328	4.254743862	4.138257463	3.866981648	4.487810043	3.621667693	4.268900829	4.255224956	3.51470061	4.366142671	4.150126982	4.093044239	4.015072816	4.078687894	3.748676668	3.863101838	4.072777255	3.813347019	4.331636564	3.919311381
hsa-miR-154-5p	3.669688171	3.67706929	3.079709465	3.544082359	3.668483421	3.500552349	3.761549135	3.937004403	2.826892035	3.009353711	4.32278096	3.671628691	3.892792482	3.62005834	3.458327941	3.772425124	3.896732242	3.360239218	3.836872944	4.37441628
hsa-miR-155-5p	5.380895137	4.987769276	4.915813377	4.88331798	4.369770545	5.017591764	5.894325869	5.398640425	4.32776233	3.396847482	7.10064155	7.280231149	6.365525223	6.228097663	6.769182228	6.759131435	6.76107137	6.238695024	5.803305885	4.931597023
hsa-miR-1587	3.602463464	3.764079311	4.216799525	3.642862764	3.821165158	5.086353897	4.05858802	3.759360919	4.965384592	4.050070704	3.978182398	3.949652121	3.927478532	4.574403587	4.310147648	4.217373289	3.790203145	4.219033242	3.931559495	3.978056749
hsa-miR-15a-5p	9.052524377	9.139523479	9.028508224	9.218036072	8.978637524	9.073818086	9.415242341	9.26674352	8.090561805	6.244446263	9.108211606	9.495426016	9.363235192	9.20582366	9.159219106	9.242338584	9.197492318	9.15596731	9.229078909	9.519125663
hsa-miR-15b-5p	9.04586169	9.252962177	9.088340844	9.05047082	8.978517053	9.022228765	9.123808581	9.375708688	8.246714596	8.814705475	9.52373946	9.531320451	9.564584408	9.243223939	9.065333296	9.498500897	9.389305765	8.886387316	9.223750024	9.275693847
hsa-miR-16-2-3p	2.768603694	2.583876661	2.573494492	2.697271826	2.660388554	2.627788231	2.748228728	2.597784289	2.500438432	2.4755702	2.667269389	2.692836892	2.630571768	2.846622908	2.571720843	2.638383051	2.81461748	2.591788627	2.677006368	2.720916873
hsa-miR-16-5p	11.03830142	11.13671997	11.00444423	11.1886617	10.62190789	11.18777522	10.92585909	10.93930932	10.46004756	11.17919824	11.55580495	11.54812427	11.44854826	11.10134244	11.24492493	11.27582431	11.4676359	10.99376585	11.14547554	11.12779487
hsa-miR-17-3p	4.839515335	5.213065427	4.593348218	4.733393788	4.941951732	4.521163657	5.230492628	5.312671176	3.618065372	3.224461067	5.110861482	5.090613325	5.304437875	4.699780176	4.175195844	5.228283991	5.053470389	4.402875915	4.780427656	4.246246124
hsa-miR-17-5p	8.837558546	9.31029043	9.004366981	8.823999984	8.850496383	8.882525544	9.087484618	9.285468016	8.170786182	9.505924183	8.819269688	8.908980478	9.314252397	8.903767885	8.430483183	9.317720207	8.810958037	8.83361964	8.783663464	7.526769429
hsa-miR-181a-2-3p	2.744555595	2.813529954	2.703399895	3.105631298	2.954404126	2.807508265	2.815417591	2.915533101	2.630890305	2.66914221	3.151636986	2.936403877	3.120661492	2.835618721	2.913028956	2.855295836	2.964195027	2.720718943	3.085032887	3.835669208
hsa-miR-181a-5p	7.333432268	7.328772461	7.104757764	7.141213206	6.844201942	7.102696215	7.70302627	7.558593169	6.270161853	7.310269447	8.243664579	7.84796213	7.939260816	7.529484083	7.534659659	7.955623402	7.805278807	7.160038807	7.978774553	7.506291132
hsa-miR-181b-5p	4.473599776	4.118099253	4.02106208	4.328873769	3.634296596	4.014166775	4.704856339	4.473382366	3.356908882	3.525659659	5.588927654	5.105626385	5.09844003	4.464582566	4.639457132	4.877758906	5.062988059	4.141068327	4.918511762	5.055350965
hsa-miR-181c-5p	3.207025515	2.847113159	2.7611534	3.097576526	3.056081166	2.982364803	3.285006012	3.124723382	2.774460021	3.118696623	3.870783701	3.440649659	3.642936482	3.311966652	3.228427072	3.496238092	3.369894986	2.956783713	3.392836982	4.628268669
hsa-miR-1825	4.194261675	4.519402778	4.101348544	4.014942977	4.686844643	3.790773873	4.522288648	4.690136303	3.312600532	4.42340131	4.359476655	4.191244612	4.399903021	4.256853927	3.892117453	4.226554337	4.343843073	3.923786141	4.59217045	4.061163409
hsa-miR-185-5p	6.023973591	6.091794303	5.561837129	6.020874543	5.992277753	5.837050769	6.047748263	6.275683569	4.565478258	4.129475435	6.490258548	6.613097545	6.480281463	5.966364395	6.173827986	6.155697814	6.550547853	5.649006849	6.235028731	5.781279828
hsa-miR-186-5p	5.226731297	5.455166843	4.762954156	5.229937033	5.203625774	5.074352865	5.579955557	5.529231225	3.942900837	4.163095067	5.58657127	5.491752054	5.495340296	5.274507032	5.12248611	5.393308234	5.655952141	4.928598713	5.348447832	5.970028527
hsa-miR-188-5p	4.487091747	4.438146304	4.593647542	3.930630102	3.751321615	4.45619342	4.278722841	4.042172927	4.67707159	4.582268008	3.592655741	3.92210823	3.573815075	4.035214599	4.531944412	4.412913417	3.966083208	5.22984044	4.165792145	3.407379108
hsa-miR-18a-5p	4.738885465	5.031404931	4.197215775	4.681801963	4.825447206	4.460631345	4.765332889	5.185572144	3.270122271	4.82173454	5.146579301	4.89715095	5.304161105	4.645133891	4.407546806	5.13103735	5.340590821	4.434187943	4.458830624	4.200173038
hsa-miR-18b-5p	2.846471637	3.111785244	2.659455829	3.004024747	2.993831549	2.951076999	3.102393041	3.152199943	2.546283042	2.652384888	3.213193916	3.046897347	3.501863803	3.058669175	3.241848415	3.149524024	3.207477811	2.767834263	3.002439556	2.936796156
hsa-miR-1908-3p	3.722452668	3.68867902	3.656556646	3.6168362	4.061793673	3.312041325	4.064385756	3.929941753	3.004032974	3.871924886	3.956812765	3.693450371	3.774049067	3.924521281	3.497346317	3.544643823	3.638761365	3.51543271	4.038426285	3.726797632
hsa-miR-191-3p	4.318428855	4.592144476	4.209134923	4.110247828	4.756357938	3.790059962	4.655846536	4.494605257	3.296200554	4.583595066	4.398583868	4.367368934	4.271761389	4.393541884	4.068397601	4.153110069	4.312987171	4.068845354	4.612068475	4.037019858
hsa-miR-1914-3p	4.589290123	4.027125432	4.665508034	4.571983719	4.180497866	4.577610959	3.794217275	4.03106412	4.851096144	4.104377528	4.784881257	4.678118859	4.436382902	4.542655129	4.368376208	4.531757263	4.363977562	4.944884697	4.638772177	4.456810926
hsa-miR-1915-3p	5.978216649	5.95241663	5.978546683	5.627628668	5.872340288	6.070852934	6.406201079	5.946128963	7.758815891	6.060604049	5.72240993	5.810375842	5.641492085	6.10203965	5.898688033	5.748021973	6.140466132	5.598120175	5.744129371	6.169738656
hsa-miR-192-3p	5.882208571	6.028631757	5.749490308	5.76191543	6.066210505	5.60733947	6.044416452	6.46815175	4.166069593	6.262840853	5.416208521	5.888117415	6.007670009	5.851698259	4.99055459	5.833196921	5.585388737	5.384966477	5.938148267	3.091867322
hsa-miR-192-5p	11.88588285	11.94801709	12.06332765	11.72645931	11.85513511	11.95886311	12.0908672	12.17296901	11.3810909	11.65036729	11.56905887	11.88135849	11.9509257	12.03734722	11.27619844	11.7934393	11.44332333	11.70155519	11.89556805	7.010469357
hsa-miR-193a-3p	6.908537391	7.591457854	7.21363633	7.006481721	7.450485216	6.772855823	7.632801432	7.768126394	5.738002975	7.940722004	7.188184938	7.367465369	7.367667746	7.286288666	6.565296724	7.144174772	6.865409325	6.599157753	7.203116662	6.769453527
hsa-miR-193a-5p	4.681991539	5.303786871	4.518811707	4.520126879	5.041310484	4.661513981	4.937554916	5.329860032	3.578093312	3.920897663	4.285201155	4.567257676	4.927039866	4.747027383	4.245957611	4.61540824	4.358294574	4.426392259	5.064470746	4.323128098
hsa-miR-193b-3p	7.692818804	7.919770807	7.766888185	7.991885589	8.142903853	7.545632731	7.944808604	7.832525388	6.384684416	8.090814759	7.836697956	7.707934069	7.730744576	7.79649053	7.837313484	7.495575041	8.181112411	7.212546503	7.891329294	8.420845346
hsa-miR-193b-5p	3.839718722	4.016994876	3.774346307	3.8916294	4.222012697	3.725791549	3.969124544	4.329051707	3.230238687	3.680995669	3.764943035	3.837608064	3.965188456	3.925205567	3.771966293	3.850820083	4.043660849	3.641661783	4.346505738	4.237101214
hsa-miR-194-3p	3.313839893	3.396124398	3.311739509	3.195792136	3.504548803	3.087132602	3.3670241	3.650862279	2.726088477	3.353799814	3.154511682	3.268631645	3.358008824	3.310520662	2.854221897	3.269761309	3.247523781	3.054310353	3.234373767	2.530646751
hsa-miR-194-5p	10.89246279	11.00708301	10.996572	10.82764791	10.80176296	10.9925537	10.91699675	11.17292077	10.2808478	10.65816716	10.2633525	10.70113528	10.76200352	10.76358933	10.18595083	10.62932868	10.33464843	10.69181696	10.82221785	5.905747805
hsa-miR-195-5p	9.12086558	9.090665874	8.957654891	9.046082667	8.75299454	8.880576304	9.802728932	9.215753375	8.530250135	7.390266731	9.594678706	9.056738339	9.5540271	9.086057	8.862201422	9.509027067	9.192773305	8.993909156	9.525685003	10.39296081
hsa-miR-1972	3.289491913	3.114943526	4.057828447	3.088858554	2.939706072	3.655837371	2.970536447	3.075583076	4.815328111	3.471441326	3.263919432	3.339794801	3.253820863	3.651600775	4.159645758	4.087023609	2.94909337	4.264311761	3.290743039	2.844386662
hsa-miR-1973	6.235962379	6.441123046	8.375691456	6.426163689	6.154249271	6.521864598	5.802703749	6.548870816	7.661720618	7.741273309	6.82628196	6.623495536	6.608311169	7.17215417	8.250931738	8.086132758	6.759418096	8.497643387	6.339537152	5.280063185
hsa-miR-197-3p	4.317339453	4.433944441	4.134085485	4.275048231	4.466493733	3.950194708	4.647976464	4.566989551	3.295716144	4.194673827	4.367378942	4.44689272	4.485886595	4.348470967	4.080195815	4.52169711	4.766383846	4.211676734	4.517410307	5.345830787
hsa-miR-197-5p	7.304752912	7.278452294	8.485286529	7.40800193	7.138727814	7.430624397	7.193009459	7.227995717	7.277398039	7.960058297	7.158106237	7.504654512	7.333943993	7.498537069	7.912119793	7.967917031	7.119539218	8.051868807	7.518083451	5.601392911
hsa-miR-199a-3p	10.29381061	10.13279775	9.83237935	9.768996294	9.60690663	10.00265884	10.48519727	10.04927821	9.573554318	9.144715142	11.25025176	9.985287966	10.6124765	10.11068201	9.894230886	10.50749874	10.12834383	10.10862973	10.8319203	10.64648029
hsa-miR-199a-5p	8.584636398	8.507644756	8.078431701	8.150534969	8.240968209	8.095270325	8.938188798	8.706678324	7.931622109	8.537906348	9.745332561	8.368178653	9.080094337	8.415030016	8.003654389	8.902007635	8.446048701	8.233959424	9.348401759	8.887164683
hsa-miR-199b-5p	3.965598725	3.758668499	2.788596409	4.781366129	4.693488118	2.952293098	5.020362707	3.451831075	3.018331118	2.938381279	4.280117013	3.975729881	4.781935318	3.665051175	5.157254005	4.014422397	5.869965098	3.814687966	4.501301373	9.020555488
hsa-miR-19a-3p	8.973488858	9.168959266	9.01218953	8.849737267	9.237425277	9.003665308	9.460963708	9.144801371	7.994172097	8.314607533	8.676872644	8.61738287	9.250631923	8.822279124	8.368488208	9.070063522	8.738686609	9.215581558	8.398994724	8.188833229
hsa-miR-19b-1-5p	2.537857216	2.562933821	2.529890561	2.643254233	2.736248611	2.557614407	2.62770453	2.765165841	2.55127016	2.746546122	2.68785278	2.74465256	2.8384015	2.702779079	2.425344537	2.716894593	2.656852097	2.649566513	2.753384851	2.597938597
hsa-miR-19b-3p	10.67945861	10.88390303	10.82220521	10.4669036	10.65522242	10.72852974	10.96746729	10.80216259	10.16543372	10.56641393	10.57490527	10.61040304	11.02520964	10.70777167	10.35604515	11.0111063	10.55599093	10.87234163	10.35595348	9.645597979
hsa-miR-200a-3p	5.438280618	4.939713883	4.140744146	4.710651412	4.775843693	4.38475955	6.100300439	4.789136205	3.950264065	4.856950926	7.080032034	5.538737669	6.06023909	5.399124362	4.513316109	5.719593156	5.263485174	5.017600248	6.897494877	4.032961679

hsa-miR-200b-3p	6.679767609	6.252343438	5.520019083	5.882843902	6.009461781	5.726611088	7.258397942	5.921721947	5.522901151	4.978297444	8.195592634	6.632715895	7.16238589	6.585973661	5.764659445	7.013754853	6.229626493	6.225975751	8.182181083	4.439137976
hsa-miR-203a-3p	4.384465414	4.29535403	3.795977428	4.354852129	4.000820292	3.85007735	4.376258539	4.896265211	3.084502005	3.476951093	3.976669877	4.239135663	4.386692849	4.190119895	3.539312887	3.978795132	4.080709404	4.29057975	4.599024966	3.871445018
hsa-miR-204-5p	5.097489399	5.196695444	4.947939228	4.771373942	5.067000487	4.6197671	5.864066275	5.600809851	3.626481993	4.869644513	5.171121569	4.603952356	5.865871618	4.878566751	4.449187501	5.058634773	5.458558854	4.627620119	5.072594709	5.970155595
hsa-miR-20a-3p	3.888732961	4.005397407	3.403288028	3.691876569	3.855881079	3.459084233	4.176005691	4.066255165	2.84617956	2.802056023	3.788602662	3.808921437	4.012448881	3.834588062	3.467424521	3.946478789	3.683894839	3.536378316	3.777927353	3.972485325
hsa-miR-20a-5p	10.47584623	10.65735223	10.46435559	10.26881301	10.28014251	10.49136535	10.61452911	10.55698178	9.879626536	10.79473062	10.24070193	10.36076661	10.76909132	10.47712311	9.970981784	10.8022155	10.19047712	10.48080087	10.2032487	9.151542621
hsa-miR-20b-5p	8.161393713	8.356350597	7.997545682	8.003438579	8.011116251	8.163715208	8.315703389	8.401010876	7.245925646	8.245352364	7.892154395	8.125306336	8.469760722	8.130138229	7.57028695	8.378822604	7.85408739	7.97503506	8.024960823	7.087636819
hsa-miR-210-3p	5.554308609	6.610344725	5.435362755	6.152377595	6.256471787	5.354297427	5.965185641	6.334789279	4.754670776	5.89012218	5.934777403	5.976568876	5.506114028	5.51486356	5.570904423	5.70678616	5.278290124	4.694449208	6.090859905	5.520586959
hsa-miR-211-3p	5.469226814	5.356070002	4.572176084	4.823509636	4.940748115	5.457602878	4.835367523	5.265081187	7.9885623	5.448333858	4.577686039	5.077276011	4.912612948	5.578429635	4.603530799	4.711680351	4.864725045	4.658462128	4.779990571	5.007862938
hsa-miR-2116-3p	4.020961297	4.279591102	4.243220334	3.703548682	4.370838161	3.518056627	4.210098773	4.104015526	3.143013494	4.205331986	4.12389498	4.021543816	4.205450714	4.024079136	3.712754054	3.737077967	3.957571674	3.752161716	4.282499945	3.995887931
hsa-miR-212-3p	3.833912371	3.582944999	3.424411258	3.839190633	3.402801833	3.549159567	3.488697476	4.085971189	4.208432685	3.844770229	3.743092856	4.055297191	3.990483386	4.221700157	3.503675355	3.580281233	3.888604159	3.627610218	3.783002823	3.804118929
hsa-miR-21-3p	3.162133585	2.97974564	2.857411725	3.095129531	2.953073637	2.890867442	3.333032609	3.301961156	2.892349408	2.708704449	4.476748596	4.122628994	3.590142776	3.632041973	3.534127133	3.381163394	3.903419322	3.198289558	3.700085789	3.172344019
hsa-miR-214-3p	6.780897654	6.702303029	6.370390747	6.337695511	6.140711966	6.322854041	7.114385829	6.714850083	5.376320428	5.462228212	7.830513951	6.781714851	7.41692329	6.534760465	6.415101594	7.048964419	7.016377753	6.267920473	7.449264638	7.813737095
hsa-miR-214-5p	3.684649853	3.26804459	3.083149663	3.418033489	3.364028297	3.125103555	3.880651122	3.548344497	2.850265729	2.951372537	4.445327724	3.469015078	4.350079007	3.596231825	3.276736682	3.862739354	3.589920304	3.155354855	4.153501997	4.324498814
hsa-miR-215-5p	10.8101688	11.07347769	11.00565155	10.71926649	10.86489588	11.00097479	10.88509756	11.02288457	10.15573446	10.28477437	10.28802125	10.66682119	10.74794457	10.86507425	10.15116958	10.62627033	10.22357406	10.78918613	10.69587278	6.027281347
hsa-miR-21-5p	12.00743067	12.2244115	12.18228751	12.13421314	11.80200238	12.17328908	12.35208854	12.35885363	11.85046254	11.17076533	13.47322702	13.20599578	13.01189576	12.79196313	12.85728446	12.88977276	12.85185419	12.27840844	12.86985854	10.78174217
hsa-miR-218-5p	3.955546892	3.741629503	3.100897371	4.411447275	3.861631718	3.542505015	4.562201841	3.750476606	3.131932812	2.86650461	4.95918635	4.540204185	4.889885544	3.968164133	4.094831008	4.850825295	4.90873957	3.724426275	4.56621365	7.505527697
hsa-miR-219a-5p	3.475926001	3.808895089	3.527477169	3.742749564	4.487546833	3.108343411	4.424904706	4.150491336	2.860952636	3.682887052	3.483747261	3.605403585	3.838516793	3.569080457	3.209186356	3.564115856	3.924226921	3.708537227	3.616840245	3.681872423
hsa-miR-221-3p	3.854229747	3.58467924	3.611314702	3.789477183	3.920497868	3.647705436	4.174936551	4.193768273	3.055932579	3.549198768	4.695672435	4.402649685	4.492321976	4.002945305	3.94937489	4.424219173	5.529843027	3.622048784	4.474176429	4.760836622
hsa-miR-222-3p	3.513164741	3.069147611	3.386450935	3.49507865	2.998601536	3.187778334	3.759715264	3.184603262	2.783208435	3.003175952	4.546552453	4.107373726	3.840758029	3.721448009	3.657643248	3.970338928	4.746251468	3.453527697	3.660415826	4.003127283
hsa-miR-223-3p	8.613552399	8.618051137	8.451257471	8.210320662	7.956603049	8.226708275	8.971057639	8.660155328	8.151982082	8.923403375	9.769170171	9.820498408	8.928113394	8.556142928	8.860289832	8.472773165	8.546608528	8.83774589	9.183593704	8.146782102
hsa-miR-22-3p	10.65286657	10.8286535	10.75663896	10.83398624	11.22067461	10.62714101	10.67849104	10.73910219	9.76770799	10.11398332	10.72455835	10.73823545	10.57831461	10.70380714	10.90773125	10.49194084	10.7599449	10.50005116	10.61819796	11.94172489
hsa-miR-224-5p	3.80144594	2.879021795	3.090768294	3.302186851	2.769504774	2.964232159	3.197521734	4.5388924	2.901543004	3.076169706	5.96161214	4.427296711	4.511264961	3.104684888	4.263079121	4.384196117	5.590926614	3.291561223	4.794444585	4.065334857
hsa-miR-22-5p	4.709976481	4.98348763	3.92929903	5.00903415	5.235274342	4.331131224	4.209366499	4.741297472	3.131170867	4.222113502	4.748683563	5.178964092	4.251669065	4.508984192	5.174643227	4.24620808	5.165047597	3.987633366	4.849175774	6.836342492
hsa-miR-23a-3p	9.581079134	9.694101422	9.266237103	9.58126243	9.632647133	9.494070636	9.96719965	9.664103567	9.174428804	9.082431807	10.41225901	9.903163328	10.00666197	9.655646455	9.983715956	9.909293368	10.01270617	9.470670663	10.19711292	11.14137081
hsa-miR-23b-3p	10.49808933	11.11598307	10.5863556	10.74940981	10.92983194	10.58763925	10.66256221	10.9343671	9.851935802	10.5745344	10.27288634	10.62840547	10.55643329	10.54565344	10.3082733	10.38610417	10.07491897	10.50035073	10.75287644	10.34591535
hsa-miR-23b-5p	2.720732144	2.719658068	2.579325273	2.999233096	2.67169448	2.745930647	2.782852123	2.853688433	2.64503288	2.506960656	2.693893352	2.702353106	2.814526856	2.664633108	2.686841058	2.677331327	2.571227062	2.629050428	2.764629844	2.776717971
hsa-miR-23c	3.80471132	3.711566926	3.7331256	3.809912843	4.189668279	3.230456849	4.259861797	4.046672848	3.048048916	4.132093843	3.933479084	3.933024537	4.086691997	3.88500543	3.645773102	3.455810694	3.738250624	3.454608834	3.86017441	3.659187195
hsa-miR-24-1-5p	3.408767752	3.99727228	3.379183475	3.678406735	4.435289618	3.382819846	3.66773634	4.205414034	2.785917577	3.384256892	3.442121452	3.597910272	3.840381138	3.505929542	3.398777522	3.511580105	3.811871326	3.461038292	3.741616458	5.030112554
hsa-miR-24-3p	10.06259074	10.46314211	10.04841298	10.07308885	10.28130186	10.02875639	10.12983911	10.26975071	9.305282298	10.79455309	10.41243399	10.24756377	10.22194759	10.11918891	10.23938804	10.10524719	10.20283496	10.05286251	10.32102896	10.82345531
hsa-miR-25-3p	7.977948552	8.2585766	8.055107546	8.005246984	8.020850527	7.915518674	8.23602137	8.475422453	7.250284107	7.737862522	8.347925711	8.472357662	8.511318022	8.089778597	7.931598542	8.291121151	8.272694963	8.025729819	8.32981623	7.481089835
hsa-miR-26a-5p	10.96899833	10.90983817	10.86024015	10.81183199	10.70700798	10.94745931	11.12114985	10.86110378	10.11004434	11.44918944	10.76351	10.75982113	10.88960428	10.89126137	10.67922184	10.89022645	10.59788683	10.79314949	11.07674985	10.81339663
hsa-miR-26b-5p	10.98819387	11.06822585	10.95009761	10.79749049	10.6983208	11.00647937	11.17324672	11.11964691	10.48915615	10.43065984	10.80785037	11.01776085	10.99621109	10.97534402	10.69211611	10.89561496	10.65143563	10.94097196	11.07054552	10.4236829
hsa-miR-27a-3p	8.722662845	8.790322615	8.374768923	8.789933429	9.000890915	8.424706865	9.24537733	8.82437567	7.985314515	8.704921275	9.562076606	9.054516006	9.122674241	8.682552618	9.382610438	8.966385503	9.248889778	8.528100978	9.164503264	10.74984025
hsa-miR-27b-3p	10.61451587	11.20062975	10.47570544	10.6403048	10.88287553	10.57573848	10.53543622	10.93552695	10.11104563	10.68175533	10.28350495	10.4935101	10.63251569	10.43210228	10.25131448	10.2907909	10.18433425	10.4772911	10.66407447	10.4767833
hsa-miR-28-3p	2.61171759	2.80371108	2.613412069	2.750217833	2.617657368	2.609055245	2.759682264	2.741599339	2.636122868	2.363567487	2.798125201	2.656215869	2.633786693	2.716641155	2.572548008	2.65639705	2.79820291	2.703251277	2.80429765	2.53350979
hsa-miR-28-5p	6.555467804	6.673804383	6.213140675	6.458778498	6.665780806	6.60394697	6.998784792	6.980014826	5.066517358	4.370838676	6.42266993	6.667885922	6.80680881	6.698188121	5.874046251	6.544008748	6.567749562	6.441772717	6.723826447	6.323467858
hsa-miR-2861	5.658499679	6.110855417	5.81097073	5.258773524	5.519146443	6.105140016	6.26406617	5.838010452	7.467620292	6.019010697	5.17711973	5.312068848	4.768575378	5.791210791	5.661498555	5.268338884	5.410935668	5.71582633	5.374170348	6.162122756
hsa-miR-296-5p	3.605434361	4.010789106	3.775608766	3.352552102	3.516936919	3.517342008	3.95413903	3.95770099	3.427078617	4.040901049	3.425900022	3.45109522	3.572429221	3.623181231	3.495604182	3.543137097	3.639377924	3.843952277	3.700953767	3.525789408
hsa-miR-299-5p	3.556943863	3.68621638	3.307879073	3.506101771	4.063737136	3.35825721	3.651707214	3.910007206	2.745258845	3.412115317	4.351929809	3.414280616	3.866856362	3.580878309	3.784511829	3.673599363	4.06687792	3.439052598	3.764976546	4.736695862
hsa-miR-29a-3p	10.83392082	10.78079727	11.01912559	10.98234273	11.01730417	10.93175848	11.48381594	11.27925857	10.39989871	10.3582346	11.09391312	11.2140973	11.35989175	10.94963727	11.20686315	11.13619879	11.1631871	11.06400166	11.16962123	11.75145792
hsa-miR-29b-3p	8.917690058	9.019849572	9.051554453	9.214825642	9.693998531	8.993488894	9.702444674	9.311656715	8.256533618	8.720788003	9.296169429	9.406496705	9.357024938	8.943121189	9.375657925	9.153392938	9.326427924	9.206655048	9.10105136	10.30809463
hsa-miR-29c-3p	11.00253262	10.92660658	10.84537515	11.03539577	11.37361225	10.95862516	11.21441466	11.01276388	10.26303004	9.903259541	10.68745775	10.88247869	11.12606658	10.91210078	11.31495222	10.91104885	11.08720163	10.97281763	10.9991831	12.36476966
hsa-miR-29c-5p	4.867962392	4.996737828	4.556709171	5.016254623	5.28631838	4.50190215	5.288956111	5.201329001	3.837692398	4.942082254	4.566899898	4.781484458	4.98682997	4.809503243	5.20005098	4.772417137	5.386795106	4.466687881	4.934369169	6.726097738
hsa-miR-301a-3p	3.71382316	3.86770332	3.577741423	3.638240183	3.816443263	3.387376637	4.281532099	3.615925135	2.670555571	3.505855359	4.575326689	4.188279245	4.585036781	3.702220135	3.980660077	4.152791756	4.540899132	3.622824514	3.841902445	5.214941576
hsa-miR-30a-3p	5.715200589	5.824276904	4.6699793	5.905131916	5.807640699	5.373911582	6.136443905	5.709754395	4.050848726	3.788525621	6.066319452	5.374010011	5.979131856	5.588157412	5.866339407	5.856270985	6.201177864	5.363349322	6.207548213	7.129535193
hsa-miR-30a-5p	8.786536048	8.832589171	8.264091119	8.922350816	8.768775267	8.784735451	9.122430094	8.617414522	7.843795092	9.387163365	9.098805419	8.254120208	8.786946396	8.665687093	8.743270761	8.780776577	8.863585143	8.562220942	9.101786049	9.832166038
hsa-miR-30b-5p	10.75959053	10.68438937	10.64168055	10.74701438	10.58837086	10.72087593	11.02391398	10.85626855	9.365530851	10.91913064	10.48140461	10.49155627	10.62542539	10.63502628	10.40734759	10.60386568	10.51326673	10.62462129	10.64848801	10.70263771
hsa-miR-30c-1-3p	2.671680104	2.812392077	2.495162852	2.68051315	2.724378371	2.580443977	2.684744684	2.667124063	2.553421573	2.575962942	2.613140683	2.580987663	2.618217105	2.645159765	2.600797297	2.804560775	2.757301984	2.587170577	2.556544061	2.636792163
hsa-miR-30c-2-3p	2.919742245	3.024041199	2.881972475	3.151701083	3.148993038	3.17160872	3.250536708	3.086227612	3.19358885	2.877174712	3.398564123	3.000984741	3.227871245	3.205799188	2.938560875	3.156162755	3.382252631	3.068479686	3.325752421	3.798152881
hsa-miR-30c-5p	9.130079474	9.227650352	8.978406097	9.033331938	9.011673188	9.089355661	9.316580365	9.328839848	7.976078225	8.512519788	8.690432096	8.717565147	8.987515363	9.010697606	8.582462258	8.903986424	8.741293971	8.777388382	9.043746342	9.175842543
hsa-miR-30d-5p	8.119538245	8.360541442	8.197423601	8.022939473	8.175848368	7.986293339	8.444966508	8.603877713	7.358472469	8.823118576	7.941340777	7.9173814	8.1023071	8.036592779	7.688244894	8.056382494	7.96722103	7.948561843	8.145255751	8.955339605
hsa-miR-30e-3p	6.624472265	6.695237943	6.353072345	6.55723268	6.707141877	6.442303509	6.93554892	7.115666935	5.033309663	4.965523141	6.218175082	6.630469076	6.842319887	6.571543508	6.302382462	6.557066699	6.605944632	6.256539565	6.648304457	7.265393102
hsa-miR-30e-5p	9.107208486	9.140281625	9.095539829	8.908604048	9.234836501	9.033208811	9.442545686	9.288401819	8.078419824	9.982624931	8.605284837	8.786810442	9.022531821	8.954404005	8.621156755	8.756891226	8.747607374	9.017707703	8.821173036	9.341194405
hsa-miR-3125	4.070223407	3.394066532	4.045833934	3.902437087	3.639278573	3.980772025	3.435377342	3.69269196	4.217189936	3.36537595	3.92290036	4.013826166	4.089309885	3.840601873	3.966743621	4.053301773	3.858334371	4.096977276	3.751071603	3.77153454
hsa-miR-3135b	5.578826226	5.514847896	5.103729923	4.271732929	4.956163409	6.383957572	4.509792323	4.487223033	9.69594386	6.346380185	5.388208365	5.862387795	5.072013744	5.929820557	5.961803298	5.132256821	4.585372513	5.158319197	5.062343621	5.455851744
hsa-miR-3141	4.030885375	4.4437222	3.875934601	3.8354621	3.718784369	4.813263411	4.60027623	4.13078352	6.194374315	4.405371292	3.468950172	3.903007559	3.634481499	4.223044061	4.27368355	3.668486964	3.368112672	4.383133717	3.619509713	3.772645405
hsa-miR-3151-3p	3.683110716	3.506767175	4.154019634	3.732316554	3.597884653	3.600229681	3.482817708	3.695428788	3.258432596	4.281697061	3.657029485	3.992679742	3.741280709	3.996515293	3.869086034	3.973212928	3.748206375	3.83116517	3.76901509	3.343043613
hsa-miR-3156-5p	3.568858937	2.969537904	3.921001537	3.52113061	3.218784135	3.852095796	3.263685621	3.245383784	4.291990363	3.173598159	3.892009673	3.62028223	3.566899138	3.766324908	3.976772636	3.871135273	3.304281814	3.983454554	3.481633318	3.41458001
hsa-miR-3162-3p	4.412025482	4.759770831	4.484532518	4.322820898	4.976279623	4.01307923	4.894686316	4.645221286	3.266847435	4.81257021	4.51281936	4.489105212	4.592246183	4.492636866	4.136452727	4.400332517	4.550724313	4.248119764	4.87592637	4.36631818
hsa-miR-3162-5p	7.979983149	8.017951229	8.242498598	7.783098659	7.835974163	8.09482796	7.780902602	8.037373358	8.409861496	8.703333796	7.637412945	8.221856091	7.831741045	7.810464207	7.651338694	7.678166401	7.598705323	7.915128941	7.852170251	6.514783551
hsa-miR-3180-5p	3.584469671	3.437641758	3.478111943	3.455598763	3.71424994	3.115275944	3.696750827	3.493193092	2.949954945	3.522545931	3.830929227	3.54938425	3.605543422	3.454487075	3.244581866	3.46090663	3.334896374	3.2060306	3.578061661	3.595148221
hsa-miR-3190-5p	3.578284819	3.698422097	3.610586136	3.427243006	3.854517495	3.223046855	3.876258435	3.704107439	2.939612057	3.793870495	3.462836703	3.272805796	3.677479364	3.606623147	3.239437004	3.362637477	3.516138844	3.293424793	3.820079182	3.441853458
hsa-miR-3195	5.534796353	5.75096804	6.27385064	5.138686032	5.616201422	5.570705732	5.273803536	5.682644659	5.001805842	6.330777728	5.378070871	5.379631352	5.294208807	6.072399099	5.834654318	5.815897077	5.702587889	6.399774854	5.356155371	5.677772644
hsa-miR-3196	5.420103074	5.560969501	5.588181792	4.803461383	5.42144727	5.578243753	5.380946304	5.537490441	7.152382899	5.780532364	5.326738068	5.405925008	5.251912229	5.753006202	5.187905211	5.342737741	5.552995908	5.453870983	5.277723165	5.823953235
hsa-miR-3198	5.139847779	4.66592219	5.481274141	5.22855862	4.889573631	5.360914734	4.705580605	4.773993984	5.358927097	4.702585804	5.44900603	5.250195838	5.225889162	5.177481482	5.276553023	5.394328614	5.057491554	5.533231866	5.336831349	4.825994787
hsa-miR-320a	6.095165787	6.401046874	6.018122532	5.981754714	6.217784185	6.082984577	6.786491433	6.388352383	4.919863338	5.894555316	6.423972749	6.251781166	6.392015834	6.040463607	5.783410304	6.102647667	6.308486615	5.730105456	6.246417113	5.787123782
hsa-miR-320b	6.351531863	6.647739498	6.423075114	6.285308007	6.524831683	6.212112682	7.005654802	6.678341979	5.207592928	6.206443836	6.757359948	6.524270989	6.652559462	6.381656418	6.211602238	6.555735809	6.629933419	6.243065096	6.472323068	6.408613804
hsa-miR-320c	5.450999046	5.431064171	5.258429568	5.289569123	5.323972465	5.277755915	5.871801165	5.5354813	4.834478819	4.889214697	5.531369884	5.402902065	5.596733674	5.367399258	5.193149152	5.473099233	5.539408365	5.229083765	5.537296835	5.954296709
hsa-miR-320d	6.40681496	6.625741761	6.112624771	6.295794306	6.579512108	6.272677874	7.03972763	6.640516624	5.387434319	5.76563142	6.630685802	6.493714116	6.727140899	6.300486847	6.010620804	6.439835361	6.555238619	6.219298923	6.449912832	6.795524179
hsa-miR-320e	5.917165863	6.14624116	5.730735591	5.885612978	6.047990965	5.75345455	6.50385944	6.134313022	4.805050619	5.239230814	6.145238835	6.036460353	6.218874722	5.899623456	5.558840361	5.993655058	6.039734885	5.73422859	6.084022633	6.463906498
hsa-miR-324-3p	7.864688594	7.541077727	7.572295065	7.902087877	7.514136968	7.773470664	7.470477935	7.654141476	6.877144032	7.714076639	7.717005004	7.886042921	7.846192397	7.672057611	7.355796608	7.576577052	7.494568723	7.296021728	7.791131089	6.448612389
hsa-miR-324-5p	4.943711921	5.503858313	4.816752783	5.019822752	5.4080127	4.579218925	5.465756187	5.789356394	3.919175071	5.444585719	5.805195264	5.524432145	5.704538579	5.153839859	4.725478524	5.424304953	5.728349483	4.617940363	5.529233649	5.628743174

hsa-miR-32-5p	4.038576919	3.765901346	3.925654856	3.923803782	4.440243187	3.536392121	4.819190038	4.178989207	2.85365267	4.101443676	4.331137181	4.249963234	4.550223059	4.073592818	3.848485228	4.21283907	4.49732522	3.905988904	3.803292566	4.348704323
hsa-miR-328-5p	3.036748844	3.016871264	3.372400277	2.855634698	2.632908577	3.636334934	2.862591393	2.901233933	7.991136835	3.854805649	3.226480102	3.090935473	2.995467619	4.239322776	3.557796645	3.129783508	2.865354887	3.276652835	2.78254042	3.254944727
hsa-miR-331-3p	7.044360126	7.512447424	7.088293401	7.217200474	7.533628471	6.97406833	7.353977792	7.574916294	5.651506074	6.849207651	7.451772687	7.33578708	7.412665073	7.094361093	7.112231606	7.180823475	7.473794948	7.061962932	7.248818035	8.456327421
hsa-miR-335-5p	4.944954676	4.932286323	4.36327219	5.291558517	4.816673405	4.700683245	5.471992318	5.115724577	3.832101526	3.370029755	5.470698941	5.483267007	5.537743591	5.449589032	5.094252303	5.215448201	4.71822208	4.520409833	5.394237919	4.663481613
hsa-miR-337-5p	3.332414411	3.378443508	3.048101372	3.21321469	3.453292216	3.159785722	3.548943323	3.435616732	2.79679588	3.539152258	4.261476636	3.508671769	3.493390456	3.205291999	3.419847823	3.214626055	3.887739814	3.131065794	3.388751875	4.399107882
hsa-miR-338-3p	4.773514721	4.396117403	4.068201989	4.37087837	4.234743245	3.637171376	5.233257531	4.670872007	3.394967951	4.172124862	5.180361001	5.574162549	4.849440659	4.854359752	5.278414527	4.773604087	4.578640326	4.540222808	4.770183821	5.993811013
hsa-miR-338-5p	2.548537024	2.487908459	2.455645715	2.654775183	2.657925182	2.491708995	2.46801352	2.449112844	2.572144895	2.492590502	2.545517307	2.552255734	2.557923727	2.578021685	2.685442914	2.508921873	2.519863894	2.610347893	2.636843096	2.338742861
hsa-miR-33b-3p	3.708605378	3.942419848	3.811310762	3.634735948	4.196340925	3.541170915	4.133057228	4.061233149	3.490304942	3.933103605	3.964435103	3.988093385	3.866571065	3.915604467	3.6089357	3.780058177	3.923052627	3.755327167	4.12271081	3.749061089
hsa-miR-340-3p	4.182071951	4.279490407	3.999195741	4.122994978	4.557869071	3.797617998	4.17780806	4.750644973	2.842662882	3.194682549	3.686333054	4.083949206	4.154483956	4.040454584	3.665813704	4.050223015	3.969316137	3.842049227	4.086604884	4.407811188
hsa-miR-340-5p	6.676558873	6.636093985	5.965294478	6.510434601	6.723483444	6.47999981	6.549097059	6.935046215	5.012667464	4.604674377	6.091796083	6.540459892	6.757334381	6.359253804	6.059658879	6.292476988	6.284854037	6.123847871	6.375125574	5.949401133
hsa-miR-342-3p	7.60935607	7.722140067	7.455866667	7.66342652	7.426997319	7.383995787	7.829103165	7.981293196	6.680310724	7.603422217	8.545286731	8.479670234	8.075400194	7.888241977	8.058297813	8.280262169	8.023323079	7.699722373	8.009738107	7.16751937
hsa-miR-342-5p	3.487567872	3.334024322	2.913456508	3.394547866	3.068951884	3.061651885	3.411706958	3.522529894	2.874909082	2.683393581	4.063459942	4.347478702	3.925850797	3.587779387	3.638198084	4.148380773	3.948778302	3.562229637	3.783645185	3.677571214
hsa-miR-345-5p	2.659448651	2.797883275	3.251991211	2.848674739	2.763558574	3.005069797	2.897144063	2.934922915	3.14567766	2.980159669	3.097337874	2.913708669	2.870871206	3.136580494	3.133768069	3.014917824	2.996013635	3.303688075	2.906110147	2.831465234
hsa-miR-34a-3p	2.968988306	2.792670905	2.923424226	2.961510887	2.729720197	2.84664368	3.03423295	3.130327442	2.603884567	2.715061005	3.830910537	3.580214018	3.621288308	3.198503431	3.292314298	3.301434725	3.74891101	2.842651729	3.48366124	3.073238167
hsa-miR-34a-5p	8.044137554	8.843022008	9.108493558	8.551795234	7.867472825	8.05727081	8.674321291	9.08851653	7.335319595	8.163945509	10.36475607	10.00017667	9.98749103	8.878107207	9.634632455	9.678571961	10.26473368	8.505641578	9.664345442	8.032476286
hsa-miR-34b-5p	4.125826893	4.831200255	4.887698117	4.66563671	4.31494423	4.061891819	4.862309325	5.2404717	3.095602874	3.82432336	6.907960112	6.515006116	6.503784322	4.984091543	5.922121539	5.800322024	6.656156868	4.394844846	5.909424921	4.84967003
hsa-miR-3607-3p	4.477066263	4.193387891	4.070946516	4.477637544	4.77856494	4.113997384	4.768739967	4.98080391	3.261698361	4.475106669	4.33299933	4.764126145	4.585969629	4.08575244	3.965361265	4.057136538	4.094165371	3.846488047	4.397781447	3.436788356
hsa-miR-3613-3p	3.598252767	3.443104575	3.416707947	3.397461681	3.83652324	3.082695595	3.732117961	3.774365835	2.905213726	3.653579175	3.591371847	3.529367161	3.585041441	3.348867423	3.266418001	3.206569145	3.552084923	3.403989327	3.632498078	3.452255986
hsa-miR-361-3p	4.814809992	4.807771619	4.436635565	4.745314506	4.834960516	4.525381593	5.129201832	4.961395332	3.659400108	3.953582762	5.379970384	5.393733025	5.490720259	4.695486751	4.784231204	5.222062992	5.340378469	4.66724284	4.868129209	5.207097645
hsa-miR-3614-5p	3.451197879	3.57539417	3.350224299	3.250273159	3.565505733	3.261389221	3.683789732	3.350218713	3.852942714	3.619547308	3.522179292	3.437441994	3.406241253	3.584868566	3.327917575	3.154665363	3.395285043	3.196094768	3.595612948	3.399878675
hsa-miR-361-5p	6.598897922	6.605285586	6.202757996	6.385713087	6.679680643	6.315916731	6.981152102	7.052855855	5.155464227	6.634248464	6.384174385	6.60422813	6.753574261	6.458601042	6.053901437	6.423899998	6.50022747	6.109621359	6.719542869	6.841032717
hsa-miR-3620-5p	3.775068815	4.238021311	3.241396547	2.806144808	3.123911581	4.013925562	2.871736987	3.236274401	5.599602647	4.853247789	3.290215024	3.447312009	3.121290538	3.887607344	3.675466736	2.970177552	2.947636778	3.16559778	3.32423924	2.741460449
hsa-miR-362-3p	4.850012774	4.791824252	4.260723512	4.90261928	5.191188918	4.593010023	5.370304712	5.331371154	3.364877084	5.175281515	5.176405019	5.332452081	5.382570836	5.010726471	5.034239133	4.970286277	5.448809747	4.596882382	5.193320213	6.15518146
hsa-miR-362-5p	3.780590747	3.686191765	3.498867456	3.868938941	4.059463752	3.489666845	4.136980466	4.39687387	3.017497459	3.447120984	4.235213934	4.296650344	4.335382417	3.990694521	4.102139777	4.088211584	4.599160403	3.591103654	4.197994842	5.314706101
hsa-miR-363-3p	4.682593773	4.465425717	4.122448811	4.883916575	4.126344414	4.235994148	4.234656604	4.588691878	3.293260148	3.956663359	5.03197605	5.130134526	4.915400489	4.387084876	4.590379697	4.427069519	4.901957827	4.005716961	4.332748855	5.44979782
hsa-miR-3646	3.555472917	3.664996566	4.74977524	3.539000313	3.69694744	3.865746063	3.727140369	3.699828708	3.493444116	4.253720142	3.741954406	3.930310228	3.738737492	4.142608112	4.507998296	4.391899579	3.838164381	4.633960327	3.903064549	3.31775688
hsa-miR-3648	3.085996069	3.119531064	4.554109148	3.130090251	3.067340656	3.225207714	3.041147476	3.225840088	3.040855304	4.220042778	3.363654793	3.288464742	3.469667703	3.789374226	4.175663322	4.050201232	3.688919344	4.219250481	3.300048709	2.882908185
hsa-miR-3651	6.083663619	6.277027402	5.735321946	6.541925898	6.311359423	5.865428552	5.983553858	6.366617238	4.898603188	5.568084044	6.858442524	6.467917093	6.70231477	5.870642118	5.641328576	6.337644622	6.408487343	5.350037705	6.407399202	4.640770499
hsa-miR-3652	3.118008345	2.954675939	4.048961557	3.14937896	2.809205154	3.703724498	3.177044463	3.166189531	4.254555445	3.963285163	3.307612688	3.220054523	3.212854933	3.587657894	3.906579804	3.510647802	3.128925653	3.928649361	3.263190916	2.712366605
hsa-miR-3653-3p	7.436360921	7.801678363	7.69319913	7.583663048	7.75660966	7.5531066	7.728957913	8.002238197	6.439322983	8.16290704	7.39228788	7.690010622	7.482489636	7.358852859	7.028642533	7.311400957	7.061960845	7.220532154	7.733006062	5.634123443
hsa-miR-3656	4.722342655	4.946084369	4.807729289	4.543332607	4.766057597	5.019766006	4.821897212	4.766667462	5.123235892	5.129427333	4.308680957	4.437375932	4.135684843	4.652676307	4.744244119	4.422700876	4.511739898	4.715995773	4.506336024	5.463705101
hsa-miR-365a-3p	8.954459557	9.329098237	9.033565291	9.340946295	9.650584923	9.109091753	9.117041468	9.394492546	8.398439768	9.158269764	8.339571575	8.74047656	8.929274083	8.979251105	8.73499193	8.548668551	8.717610128	8.90207146	9.147559175	9.872714167
hsa-miR-3663-3p	4.526804827	4.759558141	4.887158717	3.933910911	4.732439559	4.282453879	4.846171251	4.687811117	6.814088151	4.873749298	4.57958796	4.824568866	4.473538641	5.912629056	4.677762751	4.6463938	4.527808824	4.414418565	3.933338654	3.732573703
hsa-miR-3665	5.633052614	5.903756349	5.725058574	5.498308565	5.979909229	5.858409803	5.872694808	6.155943753	6.934297662	5.799535836	6.298116167	5.990917678	5.920670723	6.056142076	5.714006341	5.930833202	6.307009143	5.546165646	5.565766032	5.795803207
hsa-miR-3679-3p	3.281712402	3.578262126	3.383773677	3.176754784	3.380852737	3.07593266	3.512222372	3.326122182	2.940245932	3.478731191	3.293020522	3.081062415	3.183592077	3.477308407	3.116814113	3.119710309	3.089103236	3.199583645	3.205398688	3.295223765
hsa-miR-3679-5p	4.109613935	4.123724586	4.860541032	3.824205619	3.938542264	5.220609606	3.924063428	4.19245942	6.751112708	4.383605478	4.339318319	4.61771923	4.310036744	4.867978019	4.869716652	4.786922863	4.015851048	4.817405211	4.321209403	4.455648467
hsa-miR-374a-5p	7.974180881	8.167522629	7.462198835	7.961785961	8.050702088	7.93511245	8.186693725	8.348057315	6.819997027	7.730012061	8.09725322	8.163615859	8.36937651	7.919382379	7.789515579	8.019516617	8.088875569	7.927460038	8.08025407	8.365369133
hsa-miR-374b-5p	6.855504043	6.775195175	6.208847788	6.866616048	6.943238434	6.772284139	7.047810199	7.150708898	5.501391461	5.947365261	7.155729631	7.102669681	7.284979487	7.047748616	6.792106629	7.065171482	7.079218025	6.33457905	7.149801075	7.18477429
hsa-miR-374c-5p	3.487233035	3.546571381	3.064315657	3.515283753	3.379785537	3.392539023	3.540105478	3.668646151	2.865358543	2.971943549	3.598102543	3.748440632	3.819646292	3.56283368	3.505584339	3.592724834	3.800261291	3.262034967	3.737485655	3.736916561
hsa-miR-375	4.93954885	4.632112098	4.860988699	5.388243207	5.017487083	5.607976294	6.069029723	4.932634394	3.809272785	3.669741943	3.375399337	3.727003136	4.846899052	5.049506582	3.968754855	4.139399952	4.10048766	4.520944947	4.378252952	3.017812041
hsa-miR-376a-3p	5.967084809	6.232734408	5.329232446	5.850141388	5.969000491	5.773409953	6.283525196	6.320538572	4.294634709	5.977555149	6.994930655	6.213364307	6.252128446	6.090390542	5.818602945	5.889653986	6.121610285	5.527020685	6.269598135	6.670445731
hsa-miR-376c-3p	6.207948391	6.439854306	5.703620648	6.256005239	6.337182839	6.13092728	6.599634554	6.568902745	4.383970598	6.045473523	7.087057031	6.574164138	6.617320317	6.347525055	6.239520778	6.054293616	6.42920467	5.732895562	6.403280059	6.840213426
hsa-miR-377-3p	4.370983325	4.600923038	4.029212853	4.152587906	4.660501723	4.166527622	5.001112038	4.687222278	3.115039459	4.355168952	5.572699574	4.67866076	4.770841256	4.424623616	4.322515705	4.469034746	4.844608714	4.224970293	4.664868397	5.669348375
hsa-miR-378a-3p	8.136319977	8.628059156	7.712647444	8.937685008	9.321834459	8.127814967	8.524700612	8.38475119	7.406596616	7.845576298	7.396855822	7.913008576	8.045007626	8.11026203	9.357915217	7.906532195	9.433163608	8.221734031	8.055134083	10.80687329
hsa-miR-378a-5p	4.815075892	5.240222505	3.999390365	5.491209153	5.857488232	4.656949759	5.184836006	4.921699674	3.550549091	4.135488071	4.126621938	4.582985387	4.775885831	4.727816367	5.814998759	4.566217019	6.276447638	4.78425274	4.941839433	8.114103594
hsa-miR-378d	4.713156951	5.032534237	4.408747903	5.289526333	5.873977723	4.711756188	4.966814853	4.881756467	3.884183044	4.466619176	4.4866723	4.584965878	4.735614119	4.655181599	5.77210912	4.602695226	5.996224865	4.833403996	4.553048187	7.833708778
hsa-miR-378e	3.090262086	3.161314015	2.747631472	3.327306469	3.25808746	2.882600573	3.137280391	3.129352968	2.879836744	2.591244715	2.745147969	2.911777945	2.983324926	3.033899267	3.592075701	3.073908979	3.628568156	2.957716659	2.913499135	4.838819561
hsa-miR-378f	3.013614635	3.296320847	3.053547844	3.601669552	3.904443442	3.463480802	3.363456365	3.238193965	3.439802595	3.257873644	3.184152021	3.17054693	3.196999474	3.327571995	4.093048997	3.209524544	4.088154909	3.406600765	3.16322074	5.581755778
hsa-miR-378g	3.6900908	3.838725651	3.360150018	4.406636763	4.830428424	3.498133532	4.076259438	3.844120284	3.07400267	3.738174827	3.684472912	3.764905576	3.590791407	3.803357429	4.831492904	3.689211403	5.340239326	3.711132708	3.684429168	6.733619505
hsa-miR-378i	8.383115985	8.923986828	7.936883828	9.153194072	9.530043847	8.417949922	8.656805301	8.63894363	7.79777346	8.156297981	7.693785377	8.099053021	8.294489135	8.323136541	9.63468456	8.102767005	9.698215001	8.53407905	8.361350904	11.18697662
hsa-miR-379-5p	3.234401428	3.46476936	3.119072588	3.117925118	3.189418461	3.202760373	3.344885487	3.615795029	2.699720338	3.169024915	4.035377003	3.422819437	3.420397373	3.368473947	3.238499191	3.344628664	3.484628159	3.207146919	3.366344555	3.745281751
hsa-miR-381-3p	3.787503188	4.246211948	3.504103273	3.931166542	4.64699433	3.58836373	4.287376397	4.643374459	2.98532229	3.781846462	4.738177853	3.953220367	4.139039168	3.93000917	4.520421561	3.873648659	4.501655062	3.724868913	4.073169014	5.189433415
hsa-miR-3907	4.352563955	4.0251983	3.730439611	3.905909947	3.277918021	4.298480319	3.385354089	4.18726403	5.736539979	4.118522707	3.69066838	3.754443022	3.710158078	4.541882336	3.515282909	3.595300108	3.579808218	4.001439721	3.805066148	4.160694536
hsa-miR-3911	6.373613792	3.732971958	3.790998123	2.994092601	2.862100947	4.437254501	3.125408382	2.882712294	6.474009274	4.00673024	3.259810034	3.615749193	3.26108138	4.129044329	3.896225336	3.390765016	3.053774828	3.535690721	3.126039062	3.115295263
hsa-miR-3940-5p	3.902867433	4.087947987	3.868985486	3.684515853	3.897991683	3.974510521	4.355524202	4.269542447	3.590910104	4.112653289	3.928116224	3.705591803	3.730937943	4.258648054	3.791866112	3.838714062	3.886676869	3.994425873	3.814954312	3.661032055
hsa-miR-3960	9.136044181	9.267875056	9.31012466	8.649185813	9.575191389	9.483262796	9.404702594	9.421085875	13.07458136	9.671014348	9.654503543	9.346354744	9.083270516	10.07186027	9.204464579	8.99684065	9.316881403	8.451212771	8.66402077	8.816085357
hsa-miR-409-3p	3.438349044	3.698918054	3.3840003	3.40889923	3.392127559	3.354659029	3.646742742	3.895789389	2.950183773	3.265683286	4.732046643	3.767002714	3.96153887	3.505418004	3.52749305	3.49736686	3.825819448	3.321132137	3.607688309	3.64098019
hsa-miR-423-3p	2.716600562	2.844324416	3.013389373	2.760193191	3.115961709	2.906189068	3.170855894	2.987152186	2.736344796	3.133421881	3.010618683	2.920999516	3.098419053	2.971016283	3.001229493	2.880340222	3.04128458	3.112016059	3.263604214	3.054822168
hsa-miR-423-5p	5.533900073	5.61659123	5.348512564	5.616103941	5.601354028	5.353341691	5.704952575	5.781793929	4.375491069	5.495973372	5.766939348	5.931965835	6.005468257	5.527746635	5.267343805	5.744251483	5.691734099	5.186627295	5.861490369	4.849613145
hsa-miR-424-3p	2.617474591	2.634041375	3.160890507	2.689087376	2.838539569	2.661150025	2.919053064	2.642535766	2.906434761	4.002017503	3.091528288	2.821481385	2.899955923	3.002008649	3.125601184	3.048600325	2.838199532	3.219935213	2.75767142	2.642571644
hsa-miR-424-5p	8.406487951	8.544820348	8.626045415	7.824124259	8.502342359	8.399485461	9.268125337	8.360775028	7.38999799	6.973404568	9.591853829	8.685834117	9.112023253	8.808920871	8.280515786	8.668093106	8.676610883	8.61806163	8.89133274	6.905445771
hsa-miR-425-3p	4.166509882	4.210145021	4.160859936	3.927612846	4.490513028	3.753376454	4.490380569	4.376105037	3.171350737	4.447449603	4.279298065	4.046559364	4.163701262	4.203704907	3.883225823	3.951288864	4.056090421	3.829510337	4.39240879	3.976083446
hsa-miR-425-5p	6.242951226	6.3940893	6.029556782	6.341701866	6.20574478	6.145392323	6.385146249	6.515580624	4.667524257	5.749475573	6.742636976	6.70650152	6.806191527	6.365162705	6.202549064	6.526671212	6.603631366	5.76673814	6.446930927	5.469914951
hsa-miR-4257	3.537291826	3.261788752	4.19229856	3.264461279	3.293657443	4.072967077	3.272648858	3.514378646	4.05411872	4.253323968	3.578781259	3.854172363	3.588056988	3.657957967	4.011187721	3.968567961	3.517123688	4.522244058	3.462324731	4.35885721
hsa-miR-4261	5.166611596	4.903435692	4.693249112	4.940012507	4.610715366	4.818204568	4.614332049	4.863416313	4.211124708	4.885138317	4.712159695	4.777351699	4.881035395	4.695161369	4.066164407	4.563251732	4.634823749	4.710471337	4.934979165	4.920010836
hsa-miR-4270	7.457064461	7.66845009	6.610210313	6.589010758	7.248119941	7.08182171	7.55288141	7.255147261	7.067678409	8.256745555	4.738937786	5.705947464	4.993909847	5.628687183	6.000456799	4.913456698	5.320524902	7.018840847	6.06016483	4.264917966
hsa-miR-4271	4.165088625	4.563405958	3.756934275	3.650910638	4.236123362	4.515957179	4.697365347	4.464612663	4.763754722	4.880291424	3.802134077	4.447009699	3.793311135	4.181654972	3.934964966	3.601760045	3.798135693	4.630306554	3.827333762	4.168051269
hsa-miR-4281	7.804607568	8.199166813	7.560066603	7.399526963	7.978287495	8.019866322	8.085626915	7.911898431	8.217741712	8.788362601	7.106639201	7.424028573	7.011220623	7.816995607	7.173299184	6.974674302	7.060793796	7.580169649	7.274356266	6.841225428
hsa-miR-4284	11.1885218	11.36163394	11.47488315	11.22125502	11.73095606	10.96283113	11.49876077	12.09339884	8.781159854	11.97982801	11.13110199	11.31228745	11.41722563	11.39483651	10.98499458	11.38062578	11.49476107	10.94769443	11.16797696	11.04536023
hsa-miR-4286	10.36902821	10.4132082	10.02468207	9.697232193	9.943887014	10.23397343	10.15545755	10.41896796	12.91099578	11.41736494	9.46611487	9.959370165	9.611516287	10.43434211	9.285693858	9.485664136	9.797070949	10.1915248	9.680049901	10.22720775
hsa-miR-429	4.151949741	3.803135447	3.196731465	3.725336357	3.712198653	3.405358072	4.950308244	3.606712336	3.209696233	3.604908479	6.001788639	4.190697379	4.859303151	4.484065389	3.393933789	4.709670077	4.195331559	4.033989738	5.891094731	2.878586521
hsa-miR-4291	5.366084861	5.780667061	5.259765683	5.369501956	5.511802552	5.072590674	5.625138761	5.81743256	4.007416601	5.955293296	5.924114329	5.809415205	5.791741008	5.480846931	5.46231032	5.50628094	5.790054734	5.134813125	5.800830452	5.464800286

hsa-miR-4298	3.932461045	3.657403591	3.872886218	3.258800104	3.304251009	4.736366751	3.81720563	3.277310594	6.129114147	4.245603064	3.536629433	3.859686734	3.294327765	4.060270111	4.359227684	3.785131547	3.025128604	4.298308998	3.445476717	4.480326582
hsa-miR-4299	9.078575685	9.031660091	10.07532026	9.161796419	8.987549056	9.271553248	8.904277276	8.874886067	9.001092039	9.59273357	8.520337569	8.920369194	8.772737962	9.128979737	9.503044335	9.523165081	8.472646865	9.769386936	8.996103487	6.790793859
hsa-miR-4306	6.080323112	6.13555285	5.710718105	6.044830451	6.08527574	6.202386533	6.165968012	6.19961912	5.407238736	3.358302221	6.409853283	6.663439299	6.454443188	5.983935857	6.257319784	6.223305328	6.542130531	5.866580864	6.167908972	6.064653116
hsa-miR-4310	4.070632935	4.32533946	3.965624796	3.848461068	4.430574232	3.600331081	4.333580064	4.22869136	3.062549439	4.246908531	4.122038887	4.083458484	4.218204007	4.062403473	3.731437721	3.985703396	4.153181142	3.867918689	4.415998138	3.902780927
hsa-miR-4312	3.413026772	3.281885228	3.233215178	3.219664106	3.71408058	3.067534933	3.776725564	3.628631039	2.886215996	3.333716938	3.434278499	3.308096457	3.458633207	3.422060006	3.252276812	3.133650461	3.092019702	3.262500584	3.550359238	3.34900288
hsa-miR-4313	4.711223024	4.890018826	4.807852216	4.648824629	5.116241528	4.166978807	5.07388534	4.998633603	4.366351341	5.121417789	4.882216966	4.913715501	4.924824413	4.836159478	4.515488522	4.731387351	4.719459896	4.62730506	4.881621005	4.58414573
hsa-miR-4317	2.915240738	2.629199733	2.845964216	2.951078002	2.913699745	2.827921561	2.919217613	2.993363482	2.834150027	2.57009761	3.234487586	3.065637148	2.98280276	2.976292214	2.905387513	2.844432866	2.934799214	2.864708128	2.988911261	3.84774215
hsa-miR-4323	3.274880468	3.153811029	3.19356809	3.168842646	3.303913333	3.000951937	3.251723333	3.14466417	2.999174888	3.303883446	3.235010749	3.186914385	3.191152503	3.329278882	3.151787798	3.219031291	3.314821565	3.042234399	3.345191434	3.183604259
hsa-miR-4324	3.182252035	3.15765109	3.212000353	3.228785652	3.218616921	3.168502687	3.488343024	3.133692823	2.859622656	2.646421393	3.126605934	3.236292567	3.115027104	3.312408308	3.08150511	3.231269007	3.124114087	2.977974737	3.198076464	4.679650896
hsa-miR-4428	5.250387113	5.415973013	6.922072039	5.27432273	4.620900767	5.599884688	5.331470036	5.243866734	5.112917894	6.133917387	5.553874727	5.455566944	5.486333208	5.772750538	6.364120332	6.262568203	5.664025364	6.44663371	5.532439257	3.469039783
hsa-miR-4430	4.069419459	4.456772632	6.090479632	4.403244238	4.17325191	4.586427795	4.238613828	4.605784257	3.920657289	6.989083363	4.565278807	4.830888581	4.86416763	4.953576823	5.461492754	5.412597938	4.727107994	5.408569215	4.652529017	3.042966366
hsa-miR-4433a-3p	3.66765334	4.249869028	3.279635651	3.849621982	4.032295753	4.02476001	4.288049122	4.158676619	3.835086862	4.205280559	3.106555338	3.367710765	2.881342449	3.184941922	3.477403502	2.978161357	2.952080214	3.831846617	3.414941502	3.720166457
hsa-miR-4433a-5p	3.959705554	4.016949155	3.609125169	3.901220847	3.971108072	3.467522385	4.131362441	3.991762274	3.27583506	3.984617085	3.928964683	3.794654226	3.984486952	3.753668976	3.589311007	3.697434083	3.855249315	3.593394869	4.067832558	3.648041644
hsa-miR-4436b-5p	3.788862467	4.08475349	3.723703548	3.609733471	4.314263303	3.760794794	4.039104575	4.102508187	3.909418289	4.238159747	4.001607306	3.895369829	3.755456602	3.995807206	3.712520393	3.859732368	3.832169893	3.714547928	4.115880456	3.865262131
hsa-miR-4442	5.077913661	4.783823168	5.142252278	4.920631388	4.51157459	5.183641426	4.672408671	4.710412923	5.942465342	4.91906504	4.962019342	5.195122701	4.922646712	5.066955228	4.972262706	5.121288516	4.671146056	5.184082529	5.056331775	4.67607785
hsa-miR-4443	5.137829562	5.152563681	5.855112228	4.543804969	4.384700172	4.997529102	4.694430409	4.75127483	5.406554533	5.623529185	4.935886964	4.831670473	4.679372851	6.181882104	5.721264067	5.458457788	5.136023761	6.370059116	4.509461275	4.698955866
hsa-miR-4449	3.104405156	2.958274763	3.084719097	3.231954513	3.169521022	3.05427214	3.238319296	3.123564398	2.906206933	2.997843283	3.495407239	3.167912847	3.320028001	3.079811855	2.926219479	3.286988806	3.041117457	2.898447998	3.317003666	2.704874254
hsa-miR-4455	4.087833546	3.517694673	3.491504915	4.140232675	3.907049824	3.75073038	3.854559356	3.331387891	7.074450428	3.892618019	3.7585632	4.02907082	3.899086859	4.087515494	3.764203888	3.706291644	3.533951791	3.053770503	3.589923573	5.000485172
hsa-miR-4459	9.486275306	10.1286365	10.73832496	8.92366576	8.888568543	9.881732644	8.973606869	9.833609215	9.63762532	10.99759456	9.026668822	9.180900132	8.870267964	9.338691324	10.11834444	9.883685004	8.731535099	10.41427615	9.545086845	7.127706299
hsa-miR-4462	3.240071793	3.34005949	4.186771431	3.455753637	2.942436769	3.719951807	3.251035843	3.266846331	4.047265427	3.620640836	3.303375544	3.405432372	3.404944024	3.706117682	4.188129108	4.017684088	3.481898408	4.083191674	3.237078581	2.760019001
hsa-miR-4465	5.306991424	5.801258303	7.200648236	5.52907543	4.981676726	6.106997918	5.534879984	5.716525245	5.186317488	6.19487818	6.267255756	5.943697167	6.072939444	6.19940974	7.000313023	6.813279818	6.349977186	6.610157484	5.970139682	3.647435946
hsa-miR-4466	4.996804472	5.401764364	5.664225043	4.82554286	5.267698199	5.642278244	5.328411929	5.464020498	5.253120233	5.92370003	5.614255748	5.314940254	5.312760091	5.725272298	5.524478924	5.472941355	5.594889259	5.410101723	5.128146694	5.516023797
hsa-miR-4478	4.387577769	4.61144694	6.078847921	4.577726384	3.60672459	4.901567896	4.44908344	4.51876851	4.312746336	5.224490888	4.837880053	4.827855451	4.831520343	5.138585627	5.866064285	5.636977475	4.796370414	5.931272509	4.66681244	3.406947817
hsa-miR-4484	4.54770265	4.717957192	4.630632228	4.354696681	4.663777883	4.44107239	4.784659658	4.594003921	6.325758877	4.896506651	4.410440206	4.699147053	4.519555085	4.727123196	4.570257117	4.606113964	4.389589455	4.697418374	4.692285588	4.049917762
hsa-miR-4485-3p	6.060417265	6.494282372	8.148934809	6.205898641	5.903896433	6.370579676	5.810064395	6.435314143	9.731457047	7.691500641	6.668956394	6.558779337	6.637380838	7.10227182	8.035453265	7.883575561	6.647442645	8.224493571	6.298731026	4.969642493
hsa-miR-4485-5p	9.365458786	9.35374439	9.981581062	9.16485875	9.319142484	8.908944823	8.514610649	9.367000349	11.64996161	10.28054822	9.245308699	9.264282435	8.917514505	9.122885039	9.470262614	9.605952068	9.037390744	9.674492416	9.18825177	7.970868492
hsa-miR-4497	4.647186381	4.382454668	4.99138224	4.406785175	4.28587274	4.607797405	4.341718975	4.507767778	4.979037875	5.282331013	4.587588576	4.716327392	4.683743165	5.074960449	4.916005019	4.748634073	4.512432333	4.463112168	4.556492661	3.890665046
hsa-miR-4499	3.673699656	4.110140714	4.744836073	3.862604064	3.504088689	4.43925286	3.813644417	3.78565873	5.363597072	4.239693507	3.621885347	3.962249235	3.774553731	4.109554204	4.700360991	4.55946083	3.777800479	4.852294055	3.617364439	3.416626472
hsa-miR-4505	5.173667584	5.021157489	6.177630835	5.279714392	5.215611895	6.313170891	4.947187123	5.163404869	6.057984787	5.652976629	5.302006592	5.444614867	5.328501324	5.797096802	5.921938341	5.82601863	5.331312921	6.106515556	5.201759326	4.772313184
hsa-miR-4507	5.304198676	5.440743565	6.045062291	5.097655664	5.306373766	6.310398497	5.294614129	5.382699911	6.853900087	6.264445589	5.36137929	5.531185449	5.465830202	5.921507912	5.823875324	5.76868342	5.446876741	5.764015658	5.257860386	4.875416219
hsa-miR-450a-5p	4.252069617	4.662749065	3.896774595	4.130129076	4.731263336	4.219230654	5.122883947	4.806986873	3.146426046	3.675140618	5.277505953	4.804497057	5.453774785	4.519141313	4.171960733	4.632036349	4.952295029	4.255058783	5.125028085	4.239794244
hsa-miR-4515	4.757085281	5.044101067	5.987148625	4.59064194	4.83146088	4.731247932	4.966866328	5.021184891	5.672334568	5.458205165	4.96706856	4.958319253	4.933895435	5.394832322	5.732466204	5.700975475	5.132982325	5.707047965	4.95534401	4.419418202
hsa-miR-4516	9.232440453	9.435646951	9.564020138	8.543711423	9.643778739	9.837133295	9.110416884	9.812309411	10.94265039	9.852064496	9.857461066	9.620448136	9.203852739	9.894754033	9.246253888	9.139108896	9.289031402	8.823604677	8.705228852	8.824625619
hsa-miR-451a	12.90808913	12.84580485	12.7265799	12.8235208	12.74264421	12.50565516	11.53212718	12.31020864	11.84360735	12.78054754	11.61569526	12.08732773	12.0701786	11.70938969	12.11577473	10.66115253	12.28598538	11.60481983	12.05180418	14.32399297
hsa-miR-452-5p	2.826287991	2.719591952	2.901750796	2.783365477	2.633364723	2.733169815	2.774266582	3.032064535	2.796974844	2.609424876	3.441400777	2.878453525	3.12590885	2.851802191	3.297865414	3.316223612	3.474272676	3.288918139	3.014712948	3.985062188
hsa-miR-4530	6.397691671	6.452741359	7.696690683	6.317832039	6.142590619	7.179918613	6.308317557	6.39922579	8.850579986	7.222932564	6.89148406	6.72541418	6.644555327	7.09068467	7.244483809	7.143360133	6.873874046	7.074925815	6.552814934	5.728586122
hsa-miR-4532	3.591914712	3.744792493	4.606728326	3.654231979	3.76555224	3.770670466	3.45836656	3.8209587	4.51055175	3.951936841	3.94482859	3.919981364	3.942765698	4.108445671	4.199949022	4.403407929	4.0002209	4.291241959	4.122361013	3.845525176
hsa-miR-454-3p	3.689733365	3.747301058	3.240465125	3.709877683	3.46573913	3.495827543	3.833285012	3.753499683	2.85527219	2.708701202	4.110780746	3.939059309	4.181299648	3.694534045	3.672135596	3.992527821	4.242218463	3.493902822	3.874487943	4.271830117
hsa-miR-455-3p	7.632243274	8.476415918	7.850617742	7.641107448	8.183462554	7.760912136	8.117023486	8.454493671	6.09762182	8.650884635	7.535502191	7.432737037	7.830702264	7.795040636	6.705252997	7.264138135	6.986551961	7.49136384	7.945407942	6.011437311
hsa-miR-455-5p	6.048813528	6.213909788	5.474564335	5.998816837	6.320446501	5.880137009	6.389201404	6.351026987	4.329189031	6.275998295	5.968224388	5.878695018	6.367828904	5.938502187	5.053985404	5.572322461	5.547289804	5.61330551	6.26024216	4.89816682
hsa-miR-4633-5p	3.276275181	2.985304238	2.886871511	2.965136064	2.959840886	3.35694124	2.731028964	2.903182914	4.529505103	2.829581905	2.912887706	2.925281677	2.961502375	3.483819253	3.137851615	2.868265216	2.891703531	3.181517398	2.939190723	2.932720335
hsa-miR-4634	4.358129507	4.320209332	4.285091687	3.18932195	4.452938564	4.735105441	3.765489896	3.634967037	10.08791752	4.636073667	4.354444726	4.658348559	3.884604057	5.559559345	4.881677126	3.921763555	4.132090815	3.469458915	3.966872155	5.285715423
hsa-miR-4646-3p	3.270355623	3.562615934	3.418735412	3.476110676	3.564540305	3.154262753	3.513594961	3.407833582	2.816100082	3.687849509	3.547266003	3.418559058	3.551395027	3.549548505	3.297041184	3.316722484	3.683361207	3.334038541	3.564882076	3.350602643
hsa-miR-4649-3p	4.419439969	4.812659657	4.423826847	4.279152887	4.761950766	4.042732087	4.626258897	4.643698152	3.311879095	4.693782908	4.499149883	4.485231063	4.624519964	4.413739072	4.070142254	4.482598975	4.490067116	4.221194993	4.809969216	4.373791734
hsa-miR-4651	2.988141057	3.013845895	4.187190724	2.867503934	2.99942069	3.02484337	2.877116322	3.068686929	3.382240872	3.421004038	3.519166385	3.59498004	3.288015166	3.713417324	3.932687662	4.213838711	3.749774311	4.071088213	3.006544634	2.906744368
hsa-miR-4652-3p	3.272815035	3.502337264	3.444116058	3.220254475	3.936102035	3.176138013	3.602896285	3.508766741	2.895659823	3.54226557	3.482892193	3.290161002	3.356360731	3.415766778	3.248962884	3.277264954	3.401466702	3.37272356	3.730468827	3.519581663
hsa-miR-4653-3p	5.514168172	5.469211616	6.319407959	5.641863707	5.374004315	5.480951796	5.403054543	5.573219054	4.331279471	5.889332767	5.610019858	5.622997257	5.683923865	5.595460088	5.952508009	5.946755927	5.618134833	5.918915908	5.603068177	4.437530632
hsa-miR-4656	3.770520138	3.729326811	4.508926898	3.757374453	3.331558266	4.049700867	3.716185109	3.656872128	4.228959655	4.249741117	3.815579817	3.805633847	3.819635412	4.24837701	4.331990737	4.252127644	3.823357225	4.285785117	3.781902068	2.636368701
hsa-miR-4664-3p	4.108178589	4.009714164	4.000613496	3.896852842	4.450962637	3.663335173	4.482687139	4.290807654	3.340328489	4.207129091	4.183998669	3.996378797	4.257697217	4.131642756	3.903154259	3.849171569	3.974814785	3.772081511	4.321931634	4.032995999
hsa-miR-4665-3p	4.935241056	5.245777551	5.02436671	4.704504278	5.33853786	4.544662015	5.360824683	5.309387281	3.956737845	5.514446249	5.062786507	4.972401231	5.025535045	5.032670582	4.659912825	4.824965754	4.986317573	4.8833848	5.234186729	4.743059753
hsa-miR-4666b	3.604049968	3.683457441	3.462050876	3.541060399	3.876846519	3.178052148	3.802581821	3.609849295	2.93429342	3.844711652	3.575372906	3.548149368	3.756629887	3.622349701	3.355565156	3.386493036	3.680539769	3.280903443	3.718849855	3.362200623
hsa-miR-4667-5p	3.238740735	3.425259478	3.849148366	2.969125578	2.792513808	4.05725115	2.838434452	2.9142348	6.686280657	3.410405219	3.410370032	3.793720191	3.387942294	3.893186529	4.291489502	4.090479513	3.073449794	4.044149654	3.108684297	3.110421586
hsa-miR-4669	4.308697841	4.629720841	5.527846703	4.547432685	4.223904311	4.992057295	4.693392653	4.638698514	6.350836105	6.277061286	4.549459871	4.708234816	4.434518686	5.041714993	5.377781463	5.124002274	4.392001331	5.223873628	4.568397449	3.883799082
hsa-miR-4672	4.962077661	5.431586934	7.23371469	5.127738259	4.727104452	5.753287186	5.017537366	5.329513053	5.59652211	5.679749315	5.501163277	5.357471817	5.413980898	5.885197425	6.90369905	6.691215716	5.692827396	6.893570365	5.343199609	3.709835856
hsa-miR-4687-3p	5.352400255	5.203452059	5.57234479	4.89050963	5.06224065	5.35566668	5.13891961	5.150432672	6.292889054	5.321864918	5.378055106	5.395060072	5.140476549	5.43316978	5.597880036	5.435147141	5.543208144	5.346495009	5.132353875	5.93829574
hsa-miR-4695-5p	3.389200673	3.10529256	4.056606286	3.149874092	3.305832778	3.570951193	3.223793414	3.071470137	3.294008334	6.238557378	3.130230213	3.559206868	3.345087491	3.494812542	3.938728966	3.695630867	2.999676093	3.854654237	3.101183627	3.195831035
hsa-miR-4697-5p	2.715292247	2.793848104	3.824208733	2.841503849	2.733162068	2.94442817	2.704274952	2.807361518	3.614789468	3.2761479	2.910752871	2.98010294	2.627162802	3.182944485	3.572067495	3.564894906	2.943618446	3.649860918	2.829233603	2.463012268
hsa-miR-4698	3.786189768	4.048130027	4.200723611	3.582186988	3.563793963	4.030739756	3.824710108	3.739638785	4.689264352	3.835639085	3.89311141	3.861287322	3.703589278	4.029680182	4.141840638	3.911456831	3.762038878	4.085806457	3.94503712	3.399045256
hsa-miR-4701-5p	3.375180035	3.637454691	3.409488953	3.222522804	3.706294254	3.181870089	3.625496972	3.561126914	2.893360523	3.536478724	3.485936604	3.63732637	3.427132208	3.479460533	3.134056971	3.470721164	3.568419107	3.29188075	3.590663827	3.41719695
hsa-miR-4713-3p	5.904294883	5.519766338	6.080704108	5.816763849	5.635474571	5.580879873	5.451511758	5.568859484	4.826461219	5.620715509	5.965388299	5.86604576	5.872531389	5.691320479	5.986846189	6.090241422	5.845322636	6.074809758	5.79596124	5.238259665
hsa-miR-4716-3p	4.538848098	3.702241918	4.871266888	4.506612962	3.921067892	4.299387655	4.106248433	4.034916216	4.08967752	3.991317338	4.584012465	4.505987982	4.633521097	4.475319543	4.501942749	4.734529362	4.422132975	4.833972069	4.324819304	4.262068446
hsa-miR-4721	5.490882218	5.865459543	7.017346742	5.652103689	5.4328893	5.91468278	6.239910332	5.636369574	4.984092422	7.948826646	5.765695017	5.808025642	5.861571553	5.934113887	6.541429165	6.486229199	5.925811334	6.527564575	5.716558098	4.303132123
hsa-miR-4725-5p	4.316185415	4.551699511	4.292163891	4.19105455	4.653149654	3.837413843	4.595188615	4.678968947	3.461536562	4.697943174	4.43014716	4.373250925	4.432040449	4.379826984	4.00878169	4.193049912	4.311298905	4.002169931	4.629984103	4.194961786
hsa-miR-4728-3p	3.614122392	3.585350909	3.31372876	3.389893155	3.827540689	3.088986226	3.60230668	3.324040781	2.993547175	3.562674417	3.509834538	3.353023003	3.324069044	3.437498119	3.165982106	3.238669216	3.221364175	3.3522852	3.296922094	3.471082062
hsa-miR-4728-5p	5.158817109	5.271528402	6.569392182	5.186680414	4.515044511	5.648782253	4.928153532	5.025647104	5.028917833	5.935777103	5.306374999	5.44016054	5.491107261	5.694392618	6.300243307	6.243166606	5.428733611	6.477461158	5.265151231	3.623429502
hsa-miR-4739	4.411430572	4.656577874	4.877730522	4.247832274	4.533808112	4.622002092	5.14735981	5.205522933	4.319340179	4.529516322	5.622942942	5.311243338	5.347246641	5.149286945	5.235516425	5.267286315	5.728468474	4.52532425	4.606381518	4.335751265
hsa-miR-4741	5.862099444	4.74099853	5.300842721	4.148993887	4.090025016	5.515885007	4.170546114	4.523739698	8.87165237	5.311286139	4.961663179	5.231612767	4.661694634	5.928618159	5.770499149	4.860564872	4.539787131	4.678048446	4.376233207	4.453992609
hsa-miR-4745-5p	3.181171622	3.335021264	3.784321395	3.325885416	3.480444183	3.207766342	3.164745259	3.649896434	3.059591	3.43721261	3.567228422	3.480127441	3.517777157	3.625380586	3.587243131	3.790384236	3.57430226	3.897976099	3.558281794	3.577632581
hsa-miR-4746-3p	4.027268249	4.135227468	5.596450634	4.032391262	3.837140556	4.476631019	4.09574605	4.036841937	3.424499834	4.455292128	4.604118827	4.307292231	4.337962741	4.591198613	5.368273544	5.134566796	4.646719362	5.471848474	4.156432275	3.372531175
hsa-miR-4749-3p	3.806811181	3.927376152	3.783200661	3.669043114	4.004483876	3.499499693	3.908872738	3.886825403	3.10371329	4.078694819	3.776197823	3.847926706	3.882602648	3.8269385	3.588617382	3.657130887	3.762298011	3.608944075	3.932386515	3.640634196
hsa-miR-4758-3p	4.070884867	4.108882218	4.090040551	3.828972372	4.240730969	3.707180284	4.249841131	4.304273936	3.565988672	4.286960848	4.239576792	4.111104878	4.169286126	4.132121672	3.754883266	3.944081843	4.05403965	3.854150494	4.408292207	3.921892294
hsa-miR-4763-3p	7.122656312	7.812056904	6.671161508	6.661744511	6.93153785	7.39674139	7.707985122	7.792042964	6.239433448	7.984377281	6.793963207	6.616045405	6.603755352	6.598348896	6.461137786	6.372332905	6.643451718	7.118872597	6.674969468	5.458669377

hsa-miR-4767	3.39620849	3.555213526	3.601200649	2.997968418	3.447907694	3.056643068	3.252669034	3.272284785	3.800510609	3.335487717	3.258517037	3.292245646	3.329636036	3.645263951	3.58073569	3.612685574	3.426126136	3.609185839	3.323874885	3.362032256
hsa-miR-4769-3p	3.618491726	3.578526428	3.500414833	3.418567082	3.76463869	3.305193432	3.811502795	3.719751436	3.008366848	3.809474578	3.752589391	3.624036212	3.344931758	3.543820636	3.263277114	3.353972854	3.486825462	3.401681954	3.630267863	3.593007427
hsa-miR-4787-3p	4.401647263	4.560160889	4.755616939	4.197613583	4.726544533	4.172635082	4.718353576	4.623136849	3.461741472	4.980953381	4.435256715	4.377118059	4.568481133	4.415922486	4.394886691	4.413437404	4.460828532	4.479390709	4.600749893	4.249961356
hsa-miR-4787-5p	4.843707207	5.004717123	5.214285148	4.559133552	4.965177682	4.611343363	4.833723554	4.974650305	7.204909977	5.152079949	4.765286426	4.955410745	4.731380655	5.230100536	5.257468981	5.034928248	4.748851196	5.091206066	4.933680725	4.82187939
hsa-miR-4788	4.392075267	4.297314035	4.96467456	4.290582134	4.0847898	4.46485803	4.082728902	4.334489982	5.268236988	4.702545329	4.200047647	4.678417888	4.317843524	4.738551859	4.742209802	4.748519158	4.298253624	4.65755085	4.230032344	3.307072175
hsa-miR-4793-5p	3.563763525	3.009571833	3.858699946	3.221967066	2.992454854	3.981959199	3.239282777	3.183562265	5.065677269	4.155717598	3.331672807	3.851615251	3.544650582	3.657218364	4.113961446	3.888960638	3.134820773	3.955350564	3.434628214	3.515963809
hsa-miR-4800-5p	3.743127147	3.627695845	4.468883556	3.404699093	3.232450016	4.534400478	3.3301433	3.7764383	6.528082296	4.166337457	3.805537684	4.130269162	3.704273956	4.364120664	4.479954284	4.471851499	3.051998104	4.553499638	3.62682638	3.840027866
hsa-miR-483-3p	5.00065715	5.206764071	4.835124668	4.81738768	5.163248123	4.841773474	5.264006548	5.341674061	3.674132788	5.474304518	5.257807178	4.698246078	4.896505154	4.892370941	4.054965459	5.581818148	5.109031969	4.821853211	4.964109936	3.995243115
hsa-miR-483-5p	5.156276394	5.253956701	5.223154835	4.709849061	4.996598924	5.452205711	5.258461655	5.44040545	6.583451086	5.450266188	5.210582612	5.583954345	5.158680886	5.330267622	4.735518825	5.338824728	4.881296915	5.016122605	5.028946936	4.736088404
hsa-miR-484	4.433004116	4.908854465	4.258435899	4.513210636	4.621530556	4.267668704	4.85130512	4.857378784	3.310824105	4.839059268	4.964146761	4.773054509	4.661105343	4.549464654	4.192902535	4.645966483	4.915751375	4.208407988	4.803082077	4.34822818
hsa-miR-486-5p	5.363000122	5.557867922	5.050291142	6.224831924	6.455450424	5.262602057	4.962784082	5.275427173	3.736281929	5.110347588	4.323072135	4.639959319	5.155000755	4.545557363	6.838297654	4.092243472	6.664599533	4.525319343	5.03138345	8.278639839
hsa-miR-487b-3p	4.740724879	4.888738997	4.207501687	4.858091705	4.641437351	4.656101629	4.55841742	5.050935375	3.298069822	4.479027566	5.428412072	4.892190266	4.931367834	4.811203847	4.264482289	4.483529133	4.950882313	4.521180713	4.771877151	4.877914911
hsa-miR-494-3p	10.08957029	10.67217507	12.91425306	10.3679301	9.447351648	11.06810741	10.08048562	10.30877946	10.93131024	11.82998449	10.91401294	10.52193825	10.36361032	11.47943414	12.6282058	12.42449123	11.0894282	12.81697843	10.60174043	6.610769333
hsa-miR-495-3p	3.49535404	3.809844437	2.966169411	3.341160251	2.982971368	3.39889455	3.454584162	3.387177617	2.781834277	2.658479056	4.20394155	3.719634838	3.767214101	3.318636587	3.400332856	3.624681868	3.832592583	3.217559665	3.501629273	3.931209062
hsa-miR-497-5p	7.336045935	7.258535213	7.19875277	7.260795227	7.099810735	7.00012941	8.114716867	7.4736625	6.391044471	7.460659496	7.859689447	7.536252363	7.877026756	7.366022011	7.066853797	7.778750747	7.561788603	6.993101628	7.804981862	8.868225459
hsa-miR-499a-5p	3.539232054	3.667142894	3.190004508	6.866096338	7.621198693	3.356414482	3.668195035	3.894418563	2.909764439	2.766057415	2.906760568	3.418959335	3.603033762	3.600070143	8.468006219	3.135107687	7.809670168	3.338053069	3.215392181	10.11431712
hsa-miR-5001-5p	3.783904351	3.85306564	4.450878813	3.65561564	3.618310577	4.014084528	3.689666926	3.833136402	5.875716669	4.326260916	3.790583358	4.016252041	3.781613219	4.298262113	4.409561469	4.137635524	3.985782319	5.633937836	3.881574383	5.038630874
hsa-miR-5006-5p	5.360641243	5.584986939	6.545986787	5.209673824	4.566839699	6.092124921	5.134406522	5.420274332	6.255866191	6.007516863	5.367524827	5.494859679	5.39905922	5.731098848	6.33870438	6.230876416	5.380671454	6.336496434	5.426075195	3.54351052
hsa-miR-500a-3p	3.235398987	3.343808616	3.251566794	3.202085111	3.190820847	3.16402151	3.612992668	3.572099153	2.888797662	3.108255697	3.688287738	3.576072547	3.595931693	3.372991226	3.549373225	3.454715127	4.064021323	3.193387488	3.383516836	4.607267704
hsa-miR-500a-5p	2.942603845	2.703686071	3.161920447	2.956558236	2.839703738	2.978678252	2.800376097	2.685684911	2.916521957	2.780233735	3.278080831	3.120732569	3.09844464	2.979712096	3.065770366	3.068682551	3.223935311	3.207930179	3.051628075	2.955384829
hsa-miR-5010-3p	3.40874689	3.437770347	3.397764544	3.414471514	3.79321734	3.144741787	3.879961773	3.568271941	2.868950388	3.48152544	3.542649564	3.332969061	3.550883448	3.468080519	3.179348303	3.251396389	3.415338171	3.295501483	3.755003096	3.398041049
hsa-miR-502-3p	3.242427633	3.379825281	3.14335913	3.294274915	3.302073575	3.239537957	3.713996901	3.741538914	2.95124954	3.222418083	3.803315426	3.676144622	3.573275566	3.805983227	3.607244751	3.732894755	4.082528202	3.303244017	3.581944216	4.935904068
hsa-miR-503-5p	2.800794097	2.744425294	2.969333776	2.522632224	2.992890206	2.842626328	3.33412418	2.794048835	2.574636615	2.974814409	4.178894835	3.350536207	3.46563522	3.066248182	2.919287291	3.003170675	3.761502671	2.748977523	3.073924138	2.680197372
hsa-miR-505-3p	5.946450355	6.250272353	5.594334591	5.880734894	6.44617343	5.831931394	5.821527064	6.618416067	4.65672104	6.054243004	5.501713651	5.933246477	5.93431534	5.838178589	5.107423074	5.484828111	5.66201016	5.509289221	6.114762342	4.727105328
hsa-miR-505-5p	3.867414611	4.129739805	3.472521128	3.833584268	4.265184264	3.574300565	3.717014154	4.413766006	3.160858007	3.234513415	3.745393955	3.970679787	4.028241219	3.921980761	3.438536941	3.774879381	3.974633314	3.59631862	4.026065664	3.544643728
hsa-miR-5088-5p	4.080597854	3.416366189	3.971470344	4.238650807	3.503295405	4.057306662	3.507683079	3.35618883	3.760520474	3.205238695	4.391341682	3.992013685	3.97347182	3.807099538	3.749768044	3.857824333	3.778302399	4.116553293	3.886265039	4.062184013
hsa-miR-5100	12.18623826	12.51520867	12.38169516	11.96094341	12.28906785	12.02844301	12.33041662	12.34415111	12.7985476	13.00709247	11.52732102	11.80706076	11.67475508	12.22524421	11.00560959	11.54984273	12.06734858	12.41565328	11.90850438	12.47829426
hsa-miR-511-3p	3.471096067	2.928837265	3.363439469	3.346921245	3.281381601	3.148734693	4.180881832	4.068889911	2.735005343	3.328365993	3.639700659	4.475335316	3.974672437	3.6467817	3.383943252	3.778953434	4.346315301	3.274327893	3.550758254	4.177831071
hsa-miR-513a-5p	5.257243351	5.348988689	6.684880183	5.228569155	4.78564443	5.533945193	5.018896087	5.210190707	5.590987044	5.997769976	5.381756498	5.498955665	5.483607189	5.723152279	6.407387628	6.347753107	5.628039963	6.369076063	5.316249744	3.192695531
hsa-miR-513b-5p	4.102942677	4.003777362	5.490543425	4.452070942	3.74031136	4.466392175	3.856880037	4.180983533	3.337129141	4.990300454	4.401525943	4.38791273	4.548391827	4.824108199	5.39625148	5.265990362	4.468784484	5.423172646	4.470307473	2.649639625
hsa-miR-513c-5p	3.433626586	3.086648425	4.388562147	3.585049679	2.960240437	3.469458263	3.269987623	3.20048606	3.013790245	3.720732162	3.390171625	3.350533292	3.579502174	3.902781349	4.381672396	4.370562989	3.601247175	4.219428257	3.353923513	2.790760446
hsa-miR-5194	4.012957233	3.66835624	4.084318387	3.871271193	3.946178293	3.879500946	3.651224851	3.529672097	3.49363328	3.721142062	4.054512772	3.970581465	4.105937975	4.095026583	4.175776759	4.184422466	3.993745666	4.156116082	3.822585555	3.546112298
hsa-miR-532-3p	3.625788981	3.941995497	3.686348622	3.924362762	3.815165247	3.699002626	4.221458152	4.405670093	3.012738849	3.67835533	4.408468072	4.219695274	4.374129326	4.024952377	4.024157944	4.256029412	4.499573068	3.622741381	4.225446999	4.883601921
hsa-miR-532-5p	4.453297903	4.968376643	4.183895292	4.774585248	4.832540695	4.313862619	5.14674278	5.293439854	3.531542647	3.347345177	5.210252266	5.310664674	5.435251522	4.950332839	4.96868914	5.103900649	5.462849035	4.395574026	5.361825405	5.694358627
hsa-miR-542-3p	4.187647219	4.169825779	3.98118438	4.003456991	4.296859412	3.991204287	5.02103296	4.378609063	3.078778581	2.65290815	5.048724066	4.41802894	5.132498726	4.513424448	4.214941321	4.514126255	4.650972212	4.311803341	4.776741986	4.076455294
hsa-miR-542-5p	3.158558098	3.28139922	3.370740261	3.127688136	3.441655309	3.095464149	3.963478434	3.584783231	2.809136985	3.788305276	4.191350289	3.713530697	3.83653587	3.557589249	3.259915505	3.611936689	3.839848249	3.380653446	3.666627778	3.733907349
hsa-miR-550a-5p	3.722982591	4.148264002	3.832474433	3.663432924	4.202350563	3.35186004	4.152900773	4.059535026	3.348489202	4.028797254	3.908488183	3.936722061	3.814790028	3.987072339	3.65817452	3.844848262	3.968487513	3.730418424	4.176543992	3.688689074
hsa-miR-551b-3p	2.852184156	2.866595489	2.797732362	3.240064663	2.854554752	3.095454866	3.208551672	2.878085212	2.823101593	2.9479164	4.24097698	3.151701313	3.609974043	2.925679911	3.078635775	3.9478579	4.741398394	2.9224655	2.860351695	5.06624896
hsa-miR-557	3.344722451	4.214585184	2.987424152	3.578755738	3.392524859	3.837722186	4.226376922	4.034720142	3.699705119	4.488671545	2.749973909	2.855703231	2.7578959	3.099144426	3.171587571	2.726263019	2.805691016	3.639813164	3.032841979	2.895729248
hsa-miR-5571-5p	3.510646411	3.739103549	3.494024143	3.369101117	3.973973432	3.105369197	3.839966412	3.847840661	3.184511139	3.98602084	3.573928122	3.688506523	3.4638531	3.617818428	3.370672865	3.481215736	3.264873893	3.341554681	3.654884217	3.64472239
hsa-miR-5581-5p	4.427508442	4.059731046	4.805225553	4.403808909	4.075488859	4.128827444	3.901049083	4.314019326	3.992692399	4.077861637	4.479234959	4.508782525	4.464293487	4.504509268	4.605468905	4.798796317	4.471597351	4.820401497	4.400841291	4.278839271
hsa-miR-5585-3p	3.828829608	3.707663206	4.808541612	3.189066219	3.121668583	4.031944447	3.283904657	3.390371631	6.689381704	4.659926116	3.872177141	4.268011021	3.876697583	4.100666366	4.944077732	4.842939316	2.977345593	5.063475845	3.730754877	3.780658363
hsa-miR-563	3.495283171	3.561325966	3.383651022	3.418806947	3.858046651	3.239502332	3.772376207	3.619483527	2.956616415	3.7299119	3.660065432	3.418057425	3.698649473	3.678244096	3.333402965	3.317948485	3.358891726	3.245684392	3.636084854	3.41821334
hsa-miR-564	4.261814481	4.203566772	4.713381025	4.557371025	4.388656605	4.204649533	4.453233866	4.294390906	4.054784547	4.490891626	4.199882727	4.528513484	4.450868904	4.526241665	4.205515113	4.580857418	4.248045984	4.426224774	4.528037325	3.259433954
hsa-miR-5684	3.262968017	3.481887812	4.154122854	3.262547464	3.146812998	3.852804655	3.11477915	3.440313038	3.232851692	4.33036873	3.765043074	3.421008471	3.417365676	3.931392291	4.069122638	4.142319225	4.14505965	4.364736341	3.617710825	3.50606649
hsa-miR-5699-5p	3.26777582	3.370270591	4.724134879	3.236811452	2.752608297	3.84018562	3.083056143	3.077348424	3.485837411	4.666993456	3.445275973	3.46114285	3.912473568	3.875631852	4.316715066	4.420110919	3.681219341	4.599251015	3.460033644	2.882058604
hsa-miR-5701	4.003231952	3.723954337	3.664624471	3.854158414	4.175027694	3.61596638	3.794737928	4.318164323	3.100580545	4.181146088	3.919107533	4.13147403	4.162289999	3.612190838	3.353052283	3.784430094	3.579975928	3.396770975	4.058794841	2.948532086
hsa-miR-572	2.907759953	2.989655934	2.963875941	2.813259004	2.854523816	3.045087465	3.558113315	3.237568338	3.872532468	3.13137009	2.782719725	2.692512849	2.829402076	3.041336821	3.142074278	2.830813947	2.959260598	3.564959327	2.88674862	3.41578514
hsa-miR-5739	9.745712926	9.813899007	9.643700992	9.311769277	9.321717097	9.896651148	9.208243996	9.461785398	12.11480892	10.33882757	9.069520118	9.481478963	9.189343751	9.858150651	9.201661178	8.922762091	8.865894509	9.250641988	9.366595754	7.934168203
hsa-miR-574-3p	6.557435594	6.78680658	6.421259069	6.607046625	6.594000572	6.493236391	6.882459314	7.163718566	5.358599871	7.39614103	6.467606703	6.615871555	6.728010967	6.720019003	6.050753406	6.523818461	6.408324553	6.177102986	6.855209927	6.332386864
hsa-miR-574-5p	6.784547135	6.459328342	6.246141963	6.748563124	6.402007778	6.596478785	6.541946671	6.371178931	9.924803419	6.363759983	6.592138401	6.820805472	6.383696719	6.56773062	6.357665133	6.168299738	6.231855102	5.640965881	6.504817597	7.087443223
hsa-miR-575	4.557263123	4.547976311	5.293965579	4.092937646	4.242815392	4.678501706	4.166337324	4.211228833	5.947164334	4.727099312	4.694093156	4.677797349	4.542039577	4.893528026	5.154485371	5.160189504	4.609757651	5.020938262	4.569464058	4.294841588
hsa-miR-5787	5.079155243	5.461389906	5.825718457	4.754299031	4.510553575	5.931940852	4.553177992	5.149750676	7.085694189	5.502745865	4.88730828	5.222745204	4.711320034	5.618687044	5.701788918	5.370833353	4.995757067	5.594747765	5.215828641	4.980542985
hsa-miR-582-5p	4.365843456	3.765946087	3.590114095	3.871635356	3.5972158	3.756667472	4.276979167	4.247778478	3.094142659	3.844583991	4.946338447	4.257363259	4.864920309	4.289948577	3.944657	4.578202269	4.243325281	3.926890123	4.452547366	4.55105655
hsa-miR-590-5p	5.19783548	4.996692612	4.819931798	5.120833474	5.66045874	4.514704486	5.828411646	5.589057773	3.694926762	5.340334944	5.167211155	5.380175537	5.520316977	5.109041315	5.246032564	5.18823943	5.480189884	4.997952475	5.217583426	6.450789697
hsa-miR-602	3.533972734	3.688113385	3.905556636	3.467106292	3.836538954	3.384198918	3.632763373	4.019387907	3.442623133	3.86521959	3.822508288	3.670312628	3.532777071	3.848996388	3.639744981	3.550435989	3.62846156	3.632299442	3.634634981	3.532043497
hsa-miR-6068	4.319539069	5.27745239	4.57195602	4.530053408	4.842597309	4.582905994	6.06330667	4.977191154	5.81394497	4.954102927	3.756111426	4.206575676	3.686682456	4.492335098	4.549166897	4.115096873	4.70669909	5.33261156	4.320192504	5.482681797
hsa-miR-6069	4.512605314	4.870083616	4.617548318	4.406336971	5.097299401	4.25367625	4.973641723	4.927705666	4.035796132	5.017293598	4.671188991	4.581501045	4.60065143	4.623239766	4.276626444	4.42310051	4.510934706	4.35472763	4.854636036	4.311943242
hsa-miR-6075	2.929795279	2.774669985	3.440914921	2.880319868	2.659441595	3.110174669	2.914515654	2.763199834	2.988513485	3.195503595	2.894928088	2.78208848	2.642506382	3.131770792	3.379094143	3.245703213	2.97083132	3.35961903	2.669207786	2.670415029
hsa-miR-6076	5.908869574	4.78960241	4.433220159	4.330154371	5.768085709	5.308250117	4.389525882	4.428198851	5.413867654	4.411415936	4.534340772	5.071481463	4.712309198	4.923032333	5.151043799	4.509478862	4.331962148	4.610343065	5.025517534	6.059552703
hsa-miR-6085	9.754602979	9.842928428	9.730552727	9.70793731	9.717107901	9.602678362	9.59046976	9.82961768	7.335665863	10.37331111	9.251862708	9.424102603	9.425924117	9.532977872	8.773639876	9.314001646	9.303584124	9.612467553	9.601772087	8.375657025
hsa-miR-6086	3.258610496	2.976832096	4.35092084	3.289590822	2.995454035	4.486093305	3.117210473	3.096523317	5.141658043	4.057620137	3.502196245	3.712472614	3.360490663	3.97024503	4.288361711	4.084858122	3.183965657	4.061222147	3.287561622	2.967575265
hsa-miR-6087	7.914119943	8.408318338	9.848950257	7.902466173	8.030878223	8.498366756	7.837890276	8.041924946	11.40789873	9.539915061	8.069508723	8.195885048	7.860181362	8.874045404	9.503213689	9.002962196	7.964581142	9.280292848	8.105620547	6.914527774
hsa-miR-6088	6.918181598	7.253622741	6.472301941	6.416239739	6.787680027	7.203708342	7.141654909	6.923019371	7.534614716	7.746369365	6.049314422	6.252942146	5.924003036	6.506713603	6.542076372	5.947706447	6.176616479	6.66545681	6.199328623	5.95439009
hsa-miR-6089	9.096546185	9.518234867	9.418917239	8.614478806	9.110949935	10.06306271	9.158946685	9.084645899	13.43269133	9.641409871	8.845700418	9.058713941	8.457310956	9.942549725	9.457290903	8.743921154	8.844578234	9.086920916	8.800324532	9.58572219
hsa-miR-6090	7.847632064	8.112182638	7.892850173	7.172112263	8.316428094	8.566400412	8.062844004	8.313368646	8.18937787	8.364349767	8.337008892	7.817567172	7.712231477	8.483131093	7.583231868	7.679333028	7.986557794	7.379456546	7.373969261	7.717653459
hsa-miR-6124	5.529597485	5.689625719	5.487543426	5.300533171	5.399999445	6.122707981	5.490786012	5.447859683	6.233986784	5.869927601	5.143090268	5.491392492	5.106212712	5.524027146	5.506430093	5.209492964	5.181671439	5.62525804	5.307360147	4.945408143
hsa-miR-6125	6.91568765	7.262392489	8.10243254	6.824652464	6.961063858	7.404487156	6.889985844	6.976001488	9.897119809	7.545250639	6.83491081	7.104984429	6.824638317	7.453254395	7.546844855	7.266708354	6.942742123	7.52074485	6.966340501	7.436016663
hsa-miR-6126	4.305096634	4.411737114	5.073858924	4.170442709	4.252527833	4.675206498	4.058106011	4.554127513	3.866445411	5.704059132	4.356336652	4.392456762	4.456446846	5.025481526	4.844746126	4.869716696	4.619006268	5.032091978	4.491633475	4.402271067
hsa-miR-6127	8.671966739	8.183423629	8.901264386	8.595570772	8.352190293	8.639277068	8.076953228	8.172180894	8.326131688	8.6935845	8.44055345	8.556252314	8.128506531	8.254708688	8.095207064	8.316961963	7.942535131	8.738748333	8.440982242	7.876292883
hsa-miR-6131	5.713726624	5.088575336	5.564851599	5.520987272	5.299886504	5.434722299	5.217673838	5.170893606	4.705506876	5.121302232	5.517303085	5.460297171	5.577260417	5.33486483	5.376594635	5.492512189	5.332302162	5.589474425	5.516440663	5.079358606
hsa-miR-6132	5.751474355	5.908267146	7.074730553	5.750809426	5.817304985	6.026713819	5.890523616	5.71462002	4.678820446	6.573015676	5.664042173	5.752665504	5.902791855	5.879593849	6.572922967	6.600989155	5.943738741	6.650851714	5.606091542	4.22638043

hsa-miR-6165	7.692845819	7.621331537	7.316412158	7.47969097	7.536017715	7.744123447	7.385417123	7.6618867	7.738253652	8.000431414	7.109487385	7.985225058	7.317302969	7.37315878	6.891303178	7.076122727	7.131370186	7.389777074	7.443878835	6.240977746
hsa-miR-619-5p	3.989253469	4.065235801	4.197262766	3.058442239	3.232348039	3.705360791	3.286741683	3.1021964	8.190325018	4.567869326	4.007738238	4.502887648	3.447864275	3.936642931	4.434209629	4.197027707	2.758041492	4.266409706	3.51460203	3.089062917
hsa-miR-625-5p	2.786052284	2.781951931	2.790085762	2.836903104	2.724512985	2.886991413	3.288501535	3.02665501	2.66172016	2.674232666	3.378954448	3.533372381	3.21270617	3.041065297	3.275659265	3.407254689	3.431316642	2.998495779	3.016916058	3.789650701
hsa-miR-628-3p	3.185673084	2.930471335	3.477359569	3.570364597	3.206565142	3.293157321	3.325965086	3.051851531	3.050023626	2.907982438	3.115558103	3.549858134	3.432215812	3.446761609	3.248239105	3.410544538	3.252951467	3.282303644	3.586317943	3.225239388
hsa-miR-634	3.282318966	3.322046313	3.442566203	3.310760767	3.583574969	2.982074006	3.569771187	3.425703941	3.0820353	3.596169989	3.467688768	3.318164277	3.30926605	3.337577094	3.201604069	3.379046699	3.28616261	3.264537564	3.419383984	3.430142929
hsa-miR-638	5.693388864	6.240028061	5.975355294	5.372394777	5.69400327	5.964565702	6.788258586	5.853248582	6.837517839	6.294829704	5.094445774	5.426380432	5.172952038	5.607245927	5.814003852	5.568840525	5.681424413	5.929805759	5.487200772	5.934238139
hsa-miR-642a-3p	6.764342185	7.254371586	9.16041441	6.788260824	6.055871463	7.247135056	6.747280218	6.874245796	7.736290694	7.532316825	7.216736616	7.195067908	7.037701331	7.600475381	8.431223171	8.321430819	7.366847134	8.838639038	7.046014604	5.294019211
hsa-miR-642b-3p	4.435815192	5.123078982	6.205400839	4.447214855	4.191576077	4.914570767	4.467330415	4.989360195	5.928742471	5.143303556	4.990044827	5.05488131	4.835325769	5.35322041	6.033152949	5.769221742	4.950141157	5.967126602	4.962085109	3.424436462
hsa-miR-6507-3p	3.432232381	3.760978565	3.207948643	3.307186264	3.767844158	3.211735545	3.74737766	3.451457536	2.875647534	3.466875534	3.46582954	3.263343292	3.359226553	3.400969864	3.073954811	3.21964897	3.274644048	3.22407393	3.601329698	3.345472256
hsa-miR-6508-5p	4.550992148	4.669213342	4.332801724	4.312069721	4.827153895	3.892848538	4.791901098	4.717075755	3.410322853	4.767435915	4.462021235	4.485863023	4.532999809	4.372041913	4.040898402	4.230596573	4.389337641	4.117326032	4.609480953	4.237520266
hsa-miR-6510-5p	2.870119449	2.821758406	3.906721186	2.721625029	2.534931932	3.169733582	2.723602773	2.804649903	6.430107773	3.197933685	3.348040438	3.634702834	2.995190507	3.670454935	3.976225773	3.756420593	2.76432771	3.582363462	2.81308594	3.445134838
hsa-miR-6512-5p	3.779921848	3.204470657	3.72018606	3.842979669	3.520636643	4.019576859	3.435494701	3.392284132	4.178161716	3.318239111	3.983477177	3.777161456	3.854420616	3.7919162	3.927233196	3.986887231	3.578702705	4.053498383	3.729815293	3.751115589
hsa-miR-6515-3p	4.29811832	4.723127778	4.442173551	4.194622027	4.59238456	3.909486669	4.740584227	4.678779857	3.599709849	4.806378814	4.325275461	4.364617509	4.248645286	4.370859618	4.040014526	4.33209877	4.358881094	4.266878401	4.555424141	4.119330934
hsa-miR-6515-5p	4.246140626	4.26565842	2.559182717	4.203713349	5.076122358	5.851668201	3.333247505	3.254932826	3.443209533	3.33812171	3.685613804	3.958504326	3.55389079	2.653026078	2.821873414	3.025020239	3.28978116	2.561930034	4.164608479	5.176450142
hsa-miR-6516-3p	3.088938761	2.850155555	2.917661974	2.948368994	2.979317083	2.834138248	2.880731059	3.161137544	2.680783241	2.904558387	3.362147958	3.250528375	3.40738253	3.077135371	2.994984795	3.35996481	3.272617943	2.964450784	3.37980989	3.053930454
hsa-miR-652-3p	3.13311048	2.925581732	3.014800297	3.304708132	3.301928311	3.012335154	3.478989914	3.206871042	2.730884559	3.02555994	3.614937474	3.567963532	3.626058999	3.338086052	3.487632269	3.481179136	3.780582165	2.895994967	3.368445094	4.467892339
hsa-miR-654-3p	3.730789311	4.010810331	3.364893733	3.692422749	3.942778614	3.54844801	3.828644657	4.322316911	2.9889299	3.708372571	4.560196482	3.767452868	3.957428366	3.961361337	3.645794856	3.755767331	4.012725923	3.483797822	4.037925011	4.392054675
hsa-miR-660-5p	5.563724425	5.560386381	4.962978744	5.668498825	5.603856689	5.406186162	5.83973477	5.927363289	4.077041961	5.279483583	5.988745383	6.075302461	6.055602848	5.73776285	5.683161678	5.881955703	6.073540713	5.212449769	5.954117147	6.418297265
hsa-miR-663a	2.995930677	2.801525172	3.792406153	2.655212375	2.974467522	3.751533994	2.990979328	2.787947109	5.851270424	5.197345962	3.166991308	3.349500916	3.024441757	3.794101742	3.784110818	3.554157466	3.062938367	3.597174937	3.004915269	3.576503239
hsa-miR-664a-3p	4.838584582	5.01319777	4.595872007	4.700800207	4.971504255	4.541299725	5.047171708	5.240564852	3.387809994	4.91488899	4.810271629	4.772162247	4.95365391	4.816851969	4.269131013	4.572849803	4.789159813	4.469992954	5.137598215	4.615087156
hsa-miR-664a-5p	3.660108012	2.980625106	3.006150454	2.935788579	3.004094587	2.921647491	2.978665597	3.07104513	6.164177485	2.794968327	3.171666408	3.238967876	3.167488717	3.498963287	3.22107769	2.906065208	3.158936289	3.108498205	3.018283036	3.155991165
hsa-miR-664b-3p	4.76325789	4.933071989	4.641867916	4.546921435	5.202104274	4.368395805	5.04628579	4.845400951	4.025882289	5.137222923	4.980940881	4.834291282	4.880405293	4.721462637	4.066184497	4.860867799	4.72487285	4.609518767	4.926340195	4.527397352
hsa-miR-665	3.362486077	3.165112023	3.382262656	3.287349079	3.118482226	3.058191375	3.285290463	3.298913345	3.476860083	3.281360022	3.383780165	3.205893824	3.036244694	3.240698817	3.067062258	2.957864015	3.105160477	2.900211661	3.374145349	3.014700806
hsa-miR-6717-5p	5.551109142	5.112684012	5.541191137	5.357015851	5.27355351	5.316459784	5.061959724	5.063584128	4.27397721	5.004131718	5.261751285	5.361664002	5.417747671	5.195870451	5.341974159	5.540360211	5.327224107	5.610479365	5.455909224	5.002954906
hsa-miR-6723-5p	4.224157677	4.122017735	3.893612617	3.898976942	3.849241075	4.351403339	3.729730305	4.388944322	4.211047997	4.221265192	4.39162917	4.139514794	4.072383864	4.733481222	3.805062271	3.849484889	4.247926449	4.033914578	3.665630175	3.880009268
hsa-miR-6724-5p	4.391957676	4.444879227	5.149840772	4.143301788	4.439085266	6.288355249	4.350416801	4.383454987	5.320443626	4.518745553	4.663739042	4.725476741	4.565221735	5.547296449	5.073775086	5.036699066	4.715707886	5.159824199	4.293262836	4.519227823
hsa-miR-6727-5p	3.793135631	3.227959927	4.313408199	3.460706168	3.668424459	4.304311882	3.627280065	3.586372756	4.247351094	3.721995048	3.938884808	3.685781228	3.695869024	4.611781367	4.203680022	4.157418536	4.026416931	4.024594019	3.680230653	3.942875079
hsa-miR-6728-5p	3.351608533	2.988095698	3.79582711	3.198756756	2.917286745	3.602925874	3.299236905	3.188800299	4.563479692	3.431367728	3.620173945	3.470621885	3.196533971	3.748135117	3.940451153	3.710681367	3.137478039	3.530938617	3.306401279	3.580930148
hsa-miR-6731-3p	3.564838901	3.740506566	3.604538932	3.50482133	3.969650423	3.204021744	3.664495531	3.761037609	2.95494125	3.837710918	3.759441339	3.603054638	3.727657201	3.66898767	3.320902737	3.580238293	3.623460425	3.331284603	3.910603656	3.558806373
hsa-miR-6734-5p	4.878795078	4.606907854	5.183356733	4.749503576	4.404973217	4.907716333	4.299140643	4.539387406	5.423722279	4.501329943	5.097650933	5.007779112	4.889339652	5.066801308	5.189866385	5.173674064	4.752889861	5.2595957	4.929550898	4.529651566
hsa-miR-6737-3p	4.239050236	4.736018628	4.441041079	4.307972059	4.778046147	3.924014184	4.651902025	4.630443779	3.307997474	4.662118987	4.409582259	4.413214979	4.481078309	4.418304416	3.989877364	4.375342389	4.469021127	4.228740819	4.677666489	4.223509464
hsa-miR-6740-5p	6.038392089	5.61883044	6.403388483	5.979378841	5.639883106	6.575092718	5.576378876	5.600095377	7.306992352	5.84312985	6.092639108	6.184939313	6.107394195	6.075719084	6.254277115	6.229982819	5.874978561	6.18995366	5.935895779	5.453714525
hsa-miR-6749-5p	8.929666427	8.953413573	8.633214018	8.39630096	8.335891144	9.183093567	8.302256584	8.660045567	11.5000841	9.363255298	8.125579493	8.564030826	8.116558658	9.107465791	8.31894365	8.074136298	8.085232996	8.309798295	8.355829794	7.428964534
hsa-miR-6752-3p	3.843681353	4.014178848	4.01722754	3.653843538	4.014498441	3.31028408	4.100589418	4.031308762	3.113043714	4.136985939	3.88994826	3.931552611	3.85885433	3.890667627	3.490260458	3.689169431	3.674614143	3.561334949	4.105855434	3.768909816
hsa-miR-6752-5p	3.498485172	3.815380286	4.235778473	3.257609671	3.240393783	3.880952344	3.494099037	3.590607376	5.554185692	4.034337191	3.999237193	4.02687838	3.707285031	4.122944294	4.253534929	4.063342498	3.746996044	4.210889465	3.705531554	3.741821322
hsa-miR-6756-3p	3.110681251	3.284168217	3.279001136	3.098482182	3.419400731	3.07823437	3.333056096	3.149272513	2.945851512	3.379556149	3.331083586	3.230351188	3.084943471	3.339201511	3.176871826	3.054809701	3.09699545	3.137318355	3.462806038	3.220547421
hsa-miR-6756-5p	4.16110047	4.487112019	4.041476021	3.794920674	3.738001946	4.446538691	4.566692048	4.257700869	6.082726343	4.656986676	3.795757714	4.505543445	3.58232864	4.270471639	4.241572285	4.005539872	3.822408678	4.582909972	3.803073594	3.495614616
hsa-miR-6757-3p	3.522237328	3.79020555	3.624329293	3.487640614	3.844380331	3.235214503	3.898396116	3.823937986	2.936425906	3.758817953	3.607942272	3.578937193	3.527079009	3.542430921	3.249442911	3.370840288	3.597479074	3.489883492	3.948253733	3.436349889
hsa-miR-6757-5p	3.609078832	3.189236315	3.47620296	2.934028376	4.141714372	4.478654356	3.178146425	2.884568176	5.280749666	3.748614973	3.337432237	3.965166363	3.498934049	3.664218558	3.874125382	3.483165161	2.917384082	3.632826542	3.447694953	4.581611411
hsa-miR-6759-3p	3.424490262	3.624508496	3.561378595	3.312228997	3.94081174	3.213294315	3.628126454	3.606545286	2.892135087	3.628685501	3.632327294	3.696692714	3.536376245	3.530549137	3.296815342	3.606201953	3.654982955	3.490688416	3.804206402	3.584720795
hsa-miR-6760-3p	3.662264996	3.920832033	3.642602551	3.605209824	4.096193292	3.291924008	3.854134444	3.845113871	3.042104605	3.79821885	3.649870733	3.560151639	3.598290198	3.799216098	3.432970672	3.661556531	3.656991572	3.579679015	3.904034732	3.620481011
hsa-miR-6763-3p	3.864974692	3.907527247	3.774775263	3.562128453	4.120544942	3.382650942	4.010517234	3.848011109	3.187934566	3.958759212	3.834194727	3.887153049	3.874996464	3.863113201	3.528725054	3.671627046	3.869464039	3.562983321	4.047629607	3.712532812
hsa-miR-6763-5p	5.713386225	5.634483128	5.407661402	5.332478171	5.406681029	6.072145116	5.408676134	5.61544668	5.742360531	5.815960294	5.253444794	6.351877356	5.465997936	5.523974667	5.055303548	5.279676563	5.305283308	5.280092226	5.423070348	4.758989463
hsa-miR-6765-3p	3.463878308	3.68037613	3.901859447	3.437552812	3.857611563	3.469456286	3.612850203	3.974241379	3.041711799	3.907664939	3.640795256	3.770687852	3.752817673	3.719098722	3.644340794	3.67325327	3.701859834	3.651812085	3.841037835	3.498010291
hsa-miR-6766-3p	3.653843337	4.03195628	3.873199115	3.567146341	3.685393295	3.728333619	4.072728918	4.064885757	3.500811433	4.12315262	3.636278951	3.535599474	3.575375162	3.742852942	3.564039463	3.609154617	3.586503396	3.884037052	3.846356753	3.296754392
hsa-miR-6767-5p	4.214612008	3.947374976	4.470557548	4.212862067	3.891012708	4.204746944	3.824605641	3.986797994	3.638477181	3.824790307	4.425529898	4.492603765	4.363971761	4.2939674	4.468794554	4.669955988	4.411906004	4.692014013	4.260291627	4.078953067
hsa-miR-6769b-5p	4.747954579	5.14393219	5.252785588	4.390388617	2.944478748	4.809048369	3.851816293	4.235906581	6.310180101	5.224435997	4.598133129	4.658120428	5.034568772	4.641534211	5.653504767	5.502618123	4.405312458	4.694992741	4.723590469	3.44044313
hsa-miR-6775-5p	3.598356447	3.406120732	4.169644879	3.352453495	3.295402307	4.455615019	3.620577893	3.362069729	5.811422244	5.483061666	3.573132242	3.711236283	3.333853383	4.070554349	4.189283579	3.574596886	3.422423758	4.094889085	3.157194955	3.587462292
hsa-miR-6777-3p	3.753340047	3.902426744	3.687934758	3.631251023	4.17214575	3.41758962	3.950769913	3.858653842	3.13484131	4.030421375	3.816019436	3.588985722	3.63069349	3.805259892	3.472996055	3.67222045	3.703049084	3.579305117	3.967826499	3.56749611
hsa-miR-6780a-5p	3.767152972	3.092078017	4.025404725	3.705712691	3.166199332	3.833479398	3.228169121	3.305840878	3.794704289	3.102780902	3.784624425	3.634854917	3.598798222	3.783917817	3.953407986	3.921500276	3.527634641	4.075015943	3.343813331	3.499359893
hsa-miR-6780b-5p	5.934293731	5.654551968	6.766349311	5.76724914	5.576923085	6.267527808	5.436625952	5.6159148	5.717630289	5.988324964	5.995249775	5.901610858	5.775017955	5.932417944	6.564208688	6.469058714	5.934833892	6.727759558	5.754579974	5.236801907
hsa-miR-6785-3p	3.557807037	3.908446801	3.565906493	3.437119654	3.932659036	3.194821521	3.714441777	3.49608969	2.924994498	3.749451014	3.534596542	3.527963112	3.453011797	3.537502833	3.329422014	3.364326037	3.405013218	3.469050159	3.435262362	3.46359997
hsa-miR-6785-5p	5.696791319	6.036704237	7.597788725	5.765931731	5.499857637	6.248408843	5.835329168	5.795265267	5.297453929	6.590866914	6.093307566	6.057347237	6.124954089	6.327111185	7.158888701	7.125949948	6.244931386	7.455975832	6.052225238	3.956271691
hsa-miR-6786-5p	3.397061551	3.57725297	3.47115764	3.28585632	3.391116351	3.620931002	3.453842294	3.752726922	3.784770226	3.496102612	3.577255672	3.561469691	3.345557612	3.533115054	3.500729019	3.310767191	3.535664274	3.434340866	3.35993849	3.495505977
hsa-miR-6789-5p	2.904398185	3.01695963	3.48140971	2.931987634	2.887686055	2.983913838	3.340668029	3.11678977	2.967673304	2.951639435	3.553851318	3.288486079	3.161489587	3.58304673	3.367258567	3.344875834	3.597539439	3.141671734	2.95506706	3.596700167
hsa-miR-6791-5p	4.066319282	4.647428355	5.386578948	4.077478202	3.749449267	5.412101058	4.195041639	4.519072265	5.055634052	4.551861893	4.468447281	4.61602893	4.508447894	4.970503981	5.214961759	4.940902803	4.359400455	5.071730566	4.436048626	3.702334084
hsa-miR-6794-5p	3.992412859	3.605770107	3.784508786	3.522437275	3.189001754	4.339189182	3.490826315	3.314213829	6.1527411	3.83162848	3.599927704	3.89982492	3.188083248	4.200260095	3.995648413	3.732997758	3.451842552	4.027813837	3.375045569	3.589207281
hsa-miR-6795-3p	3.550859491	3.743379188	3.457331804	3.66480752	4.10739131	3.31954442	3.926140142	3.81697528	2.971664696	3.917259518	3.645512042	3.508456329	3.564427227	3.499503318	3.371068212	3.414871357	3.433459056	3.37201033	3.776947759	3.478733612
hsa-miR-6797-3p	4.404393894	4.585871075	4.522268595	4.173794368	4.837389158	3.994179472	4.682145712	4.700614279	3.40190743	4.814812279	4.410335991	4.264472617	4.466039631	4.393947689	4.281278803	4.479711685	4.412998649	4.315144234	4.746593069	4.191461176
hsa-miR-6798-3p	3.14013302	3.28308933	3.161482584	3.088932791	3.282004542	2.949308995	3.426131706	3.206288847	2.809604678	3.452353968	3.257567036	3.15567103	3.122898435	3.253029272	3.156883233	3.087822612	3.22611197	3.111504838	3.315871259	3.152040135
hsa-miR-6798-5p	3.009536299	3.06709998	3.777800513	3.226872723	2.815958056	3.238016606	3.168664665	3.173348301	2.920082696	4.506860596	3.356615846	3.306132343	3.241965117	3.193675782	3.678369756	3.577600596	3.295356318	3.532256362	3.058994822	2.547693326
hsa-miR-6800-3p	4.424160374	4.70320671	4.380675169	4.316314765	4.738401439	4.012066379	4.797468581	4.695313656	3.642964353	4.811817124	4.510357152	4.44209601	4.564571095	4.44975296	4.108835155	4.201915645	4.383764676	4.048244601	4.664942764	4.266866772
hsa-miR-6800-5p	6.274943175	6.202547067	6.095800407	5.559605197	5.945967524	6.728577771	5.976940382	6.081407132	10.3994657	6.159179015	6.275459081	6.346325241	5.841588422	7.11535889	6.317349	5.793956017	6.175015461	5.723109311	5.762378121	6.291880349
hsa-miR-6803-5p	5.822247905	5.844347476	6.991295356	5.898969791	5.783867956	6.096547652	5.867701605	5.794337247	6.68330135	6.853314469	5.981090037	6.17577125	6.028187603	6.222911111	6.513395895	6.597101602	6.010176173	6.591193494	5.879078127	5.274554191
hsa-miR-6807-5p	3.661690906	3.210489094	3.868223329	3.364812219	3.455864628	3.641322075	3.17173305	3.512225876	4.568022789	3.319772074	3.878040284	4.462350078	3.389016162	3.774792914	4.185090208	3.844220164	3.290615506	3.651016839	3.607854649	3.693137462
hsa-miR-6808-5p	3.963123693	3.891283348	4.287277088	3.730596206	3.155579797	4.131258899	3.466601091	3.843730748	5.145115811	4.117928515	3.723700017	3.756935928	3.739537018	4.153583137	3.89991774	3.943702627	3.717463758	4.198060455	3.562800406	3.678549794
hsa-miR-6812-3p	3.564820915	3.829652053	3.77196497	3.718104457	3.960756187	3.264073386	3.928969318	3.887976861	3.441186827	3.902631129	3.754748233	3.620572998	3.642220981	3.713667239	3.540762097	3.444108632	3.7274938	3.606030279	3.876244465	3.558337503
hsa-miR-6812-5p	4.952032866	4.822177944	6.665224975	4.275851827	4.876976199	5.502777362	4.328268864	4.449199995	6.582500908	10.51169407	4.73742417	5.702677563	4.581334686	5.294205581	5.841773456	5.417320553	4.58742326	5.50700814	4.657501859	4.479392368
hsa-miR-6813-3p	3.860003963	4.089549799	4.080816347	3.802040089	4.32891686	3.532161979	4.289736747	4.323759736	3.113413204	4.366301497	4.201648621	4.016150584	4.179325504	4.141557998	3.67125587	3.925892407	3.929323305	3.839208206	4.390827906	3.838770586
hsa-miR-6819-3p	4.450343184	4.735552987	4.357296629	4.141021778	4.660372629	4.253112086	4.759198159	4.593880586	3.499451502	4.86940936	4.446286131	4.372660227	4.463272539	4.516481253	4.116315417	4.229526164	4.345672137	4.218253114	4.701822273	4.158907268
hsa-miR-6820-5p	4.608332894	4.641304231	4.371895841	4.392608015	4.342963865	4.74687165	3.911866848	4.454120982	4.472308205	4.577286413	4.488201936	4.680705499	4.562545854	4.700312366	4.143120349	4.321936257	4.418755342	4.511538365	4.470000191	4.179676281
hsa-miR-6821-5p	6.659550908	6.844894017	6.677318636	6.377940516	7.060623395	8.564358337	6.861413638	7.025301387	6.581411719	7.000039008	6.976343247	6.793725424	6.664729471	7.629951543	6.457228808	6.668144085	6.801021514	6.093417071	6.536840736	6.208731548

hsa-miR-6824-3p	3.554874425	3.924076422	3.802233164	3.407253749	3.994532448	3.360447043	3.993834544	3.728382125	3.007382558	3.987947814	3.752028855	3.692007633	3.777965845	3.645085611	3.444528622	3.628741959	3.630277661	3.470263443	3.905489281	3.553301642
hsa-miR-6826-5p	8.077649355	8.472629201	8.091753534	7.62975476	7.751663372	7.727783783	7.927804164	8.139268519	9.262719212	9.080900286	7.521125288	7.651828832	7.516645789	8.102214848	7.144497637	7.357664691	7.719943174	7.873420402	7.669416626	7.415407141
hsa-miR-6829-5p	4.108033452	4.113671341	3.984585926	3.72863589	3.551586006	4.396733076	3.740742788	3.791738233	5.142845769	4.306711628	4.062365241	4.261139183	4.105245079	4.223500144	3.904288346	4.178192343	4.124531273	4.202252218	3.943834035	4.085750309
hsa-miR-6840-3p	3.888081453	3.457395242	3.385044062	3.452864459	3.503018113	3.679844963	3.435375675	3.77530546	3.569521765	3.774429097	3.397508304	3.509854048	3.68699242	3.762881823	3.57497006	3.381605048	3.663171519	3.795888057	3.547539708	3.611126489
hsa-miR-6845-5p	2.998977881	2.988352173	4.095365956	2.97585571	2.787896635	3.72755572	2.760102516	2.89952715	5.399828038	3.18596433	3.19036034	3.649185351	3.272023848	3.574306771	3.883009395	3.47076966	2.800166317	3.661328985	3.087225006	2.783968132
hsa-miR-6848-3p	3.834954108	3.872888518	3.658517824	3.695767773	4.007076523	3.354644666	4.053566751	3.723996701	3.063684766	3.953795921	3.799443378	3.849345436	3.643712903	3.643189639	3.409957706	3.502458907	3.692548417	3.528935637	4.039690963	3.570035552
hsa-miR-6850-5p	3.438763306	3.662229651	3.969404	3.381656108	3.782766074	3.831198541	3.853888295	3.617556618	3.954203919	3.991094521	3.938032236	3.994615965	3.766838577	4.789813817	4.006700892	4.055053248	3.980233483	3.576855651	3.40533981	3.659995531
hsa-miR-6851-3p	3.781887183	3.818959081	3.780143426	3.648679241	4.380717487	3.471883395	4.070139174	3.976822996	2.992768238	3.871935638	3.741254464	3.703659789	3.782632585	3.789038999	3.443513551	3.657329913	3.892896731	3.699638515	4.087194971	3.810717697
hsa-miR-6855-3p	3.346422219	3.303155013	3.517576159	3.260579047	3.908185372	3.085970686	3.774484137	3.529729487	2.939756333	3.736391016	3.519774902	3.57879499	3.509712531	3.456448534	3.245709704	3.436625069	3.429235414	3.254445347	3.414953826	3.450591735
hsa-miR-6858-3p	3.713158402	4.018906806	3.68180128	3.544804095	4.076821206	3.40550407	4.018006807	3.887501015	3.115489109	3.861624959	3.808454231	3.921243196	3.953548045	3.789436966	3.46415557	3.827269593	3.810714272	3.632398802	4.124832065	3.695548637
hsa-miR-6858-5p	3.994144445	4.065059179	4.407373954	3.321640916	3.362103138	5.472242605	3.300936731	3.615042766	8.192853083	4.293398485	3.710168279	4.691167366	3.560315172	4.852462929	4.962097675	4.306348275	3.596640209	4.284553249	3.44043946	3.34226358
hsa-miR-6861-3p	3.781897154	3.234030533	3.52599696	3.398297284	3.742464691	3.160995718	3.662958857	3.865557185	2.911009491	3.789724817	3.660632652	3.478711303	3.608631904	3.524027529	3.278552545	3.466940724	3.63573257	3.315070809	3.691524448	3.557696069
hsa-miR-6865-3p	4.126070608	4.316778962	4.134622748	3.868797744	4.574849	3.79733317	4.452165355	4.496040739	3.191140142	4.396811334	4.158706374	4.110731845	4.213243	4.114026837	3.786181878	4.04988819	4.155053533	3.862616646	4.443673313	4.146477969
hsa-miR-6867-5p	3.364605299	3.438537187	3.68368983	3.166340963	2.857515628	4.416199826	3.247460508	3.127112081	6.199928204	3.468673841	3.545241865	3.993390517	3.403094827	4.056672474	4.029919362	3.546798404	3.032231355	3.868668489	3.445156584	3.704985343
hsa-miR-6869-5p	7.937823153	7.887081425	7.996825453	7.051209514	6.795854134	8.232142095	7.398092265	7.394905618	11.7622521	7.755094645	7.440961479	7.509381186	7.083460722	8.562013926	8.17969707	7.138549545	7.100036022	7.354208774	7.309578694	8.216678531
hsa-miR-6870-3p	3.22891303	3.52681227	3.176066049	3.291589277	3.520319454	3.095447188	3.525872777	3.510261742	2.807821611	3.462097322	3.310021406	3.421186473	3.476044817	3.349513505	3.158978931	3.282165672	3.347216212	3.184321674	3.435134359	3.212798663
hsa-miR-6872-3p	3.232062127	3.330685367	3.233400107	2.7216144	2.822076813	3.106753093	3.050728248	3.206215912	3.205471642	3.748461039	2.918399708	3.061736897	3.092306647	3.590295738	2.997584666	3.190739754	3.306770992	3.428782056	3.213583386	3.689672914
hsa-miR-6875-5p	7.959344977	7.871981579	8.776988842	7.939760103	7.943321669	8.041404723	7.669783959	7.765602981	7.350073132	8.300203375	8.004701644	7.957618505	7.919266315	7.819668122	8.076278051	8.279077446	7.775516285	8.706461369	7.926983841	7.176237428
hsa-miR-6879-5p	6.983085707	6.24220599	6.935480976	6.569013299	6.459127496	6.797453526	6.246765695	6.15345766	6.543276973	6.369192364	6.711122952	6.963613919	6.608196185	6.6055645	6.642565435	6.672656291	6.433052436	6.802855004	6.565952407	6.260624222
hsa-miR-6880-3p	3.58730667	3.993567708	3.716083861	3.41914145	4.197694092	3.422571162	3.922680125	4.121737148	3.029433	3.880343572	3.842009563	3.749205752	3.666542479	3.715479816	3.496977225	3.463188048	3.669093191	3.454459471	4.091392222	3.591126797
hsa-miR-6889-3p	4.334938233	4.596164591	4.35269348	4.296646119	4.762036473	3.886039057	4.598959253	4.622674439	3.229326903	4.768295945	4.377056281	4.360657128	4.360439095	4.433966909	4.008281008	4.268568672	4.426673597	4.183697524	4.538884019	4.248434012
hsa-miR-6891-5p	3.960285699	3.898885465	4.024091359	3.776521122	3.824126685	4.11446255	3.929719351	3.987381952	5.921183346	4.368439728	3.922376766	4.032028517	3.793467511	4.201667024	4.127694733	3.855047833	3.941923157	3.910891227	3.649403828	4.042430602
hsa-miR-6893-5p	5.745073286	6.091417282	7.706888998	5.59389321	5.718927594	6.310591146	5.777823094	5.943599297	6.555686566	7.197973812	6.197065449	6.424305221	6.003214081	6.496027516	7.220624795	7.057147496	6.155950389	7.329138429	6.147175468	4.975676996
hsa-miR-7107-5p	4.727021842	4.734437309	4.789367206	4.460049643	4.096655145	5.379439857	4.49425252	4.507653677	6.958003439	5.194159322	4.451935601	5.3747266	4.287251794	5.057655215	4.956970215	4.606952461	4.148962217	5.04897702	4.100475432	4.967183203
hsa-miR-7108-5p	4.008665559	4.320137937	4.223243793	3.653393574	3.858864418	4.144922904	4.168801432	4.226121538	5.380861928	5.112491125	3.997085935	3.94316547	3.849623643	4.312479238	4.211443775	4.076008457	4.107279541	4.169023921	3.891857975	4.066166614
hsa-miR-7110-5p	4.060975485	4.423933093	5.245188991	4.000910115	3.739044433	4.575355554	3.901912951	4.196871815	5.553084696	4.869718362	4.270972447	4.49942728	4.295540095	4.738616916	4.924416433	5.055075781	4.287376572	5.039328398	4.134354271	3.968698375
hsa-miR-7111-3p	3.371842976	3.5470132	3.822292385	3.256484523	3.629843333	3.14971933	3.591272142	3.626074045	2.957155578	3.576518052	3.421184056	3.438149481	3.402033827	3.524044219	3.447540204	3.533950831	3.374915094	3.485471852	3.562586669	3.36869448
hsa-miR-7114-5p	7.142875799	7.385294987	6.873670693	6.895339466	6.97104697	7.010241041	6.980838961	7.383667258	6.78343281	7.936608746	6.672915207	7.029910739	6.916287489	7.075469185	6.292553552	6.752874061	6.812382042	6.941197325	6.936856748	6.266669025
hsa-miR-7-1-3p	3.401081972	3.021200632	3.134220466	3.488026392	3.312955984	3.28661679	3.663254764	3.712319035	2.833399556	3.00545203	3.399008261	3.649662374	3.750230312	3.624260001	3.381316085	3.589765437	3.691451122	3.25661046	3.424301305	4.175494102
hsa-miR-7150	4.346191894	4.413914665	5.135771257	4.185030566	4.188298096	4.452082489	4.16774139	4.265262074	4.617036747	4.8833734	4.39632328	4.544503562	4.540450986	4.81496843	4.823887694	4.996312862	4.58345587	4.985230064	4.294695625	4.159785388
hsa-miR-7152-3p	3.450895782	3.176943819	3.717768477	3.295802464	3.230453777	3.733677764	3.108436728	2.961859604	4.832346836	3.264065277	3.459936358	3.390655042	3.382767693	3.737162505	3.665116916	3.776805062	3.595555559	3.888910118	3.270670606	3.243288929
hsa-miR-7152-5p	4.906981961	3.879669017	2.682369494	3.775610793	5.979363343	4.999787144	3.50684977	3.150927278	3.04356951	4.019621678	3.783432467	4.1250086	3.142882259	2.890386537	2.704735868	3.263402858	3.561684408	2.656438799	4.67505187	6.273347712
hsa-miR-7-5p	3.420863998	3.138959853	2.972573768	3.423501035	3.188113736	3.230401888	3.228379796	3.167240685	2.769074642	2.623030126	3.531257808	3.52942232	3.513212925	3.491735789	3.580370794	3.469525995	3.518256538	3.221991594	3.369323763	3.927699757
hsa-miR-762	5.010851435	5.14413082	6.271595083	4.99268568	5.225524741	5.412509942	5.073523007	5.227908821	6.186604599	6.316841574	5.150828347	5.359550043	5.222920683	5.923955597	5.89001936	5.787895344	5.367835971	5.737027997	5.345071018	5.395373628
hsa-miR-7641	10.80605044	11.23700532	13.49553399	10.86982023	10.81089049	11.2901231	11.0323293	10.95297273	10.58217495	12.29920791	11.36832647	11.15338984	10.958968	12.10876372	12.96910517	12.65223413	11.6162656	13.29748704	11.06304227	8.968678647
hsa-miR-766-3p	4.181621015	4.376668471	4.165963271	4.102052806	4.328327361	3.898936085	4.417559288	4.292792995	3.695810017	4.387090397	4.43199994	4.361385675	4.417449115	4.289292732	4.068778496	4.299710678	4.346305771	4.080402628	4.452722415	4.177840598
hsa-miR-7704	5.36716794	5.63904628	6.098360986	5.228032283	5.29813834	5.412760213	5.501328711	5.245840069	8.314249777	5.75830938	5.103083444	5.581538619	5.195146695	5.812486508	5.88015123	5.574379023	5.405378794	5.68296355	5.34210508	5.749176047
hsa-miR-7847-3p	3.665066769	3.825134638	4.092702313	3.433794304	3.511670354	4.34323483	3.886100715	3.476938747	6.240298085	3.911920829	3.722754649	4.46177177	3.480159146	4.38831278	4.416212733	3.777797711	3.537009982	4.315864901	3.667868169	5.496382184
hsa-miR-7974	3.902774895	3.966395017	3.736303637	3.782265774	4.104076065	3.404921325	4.1157559	3.921672997	3.163095599	3.880250834	3.856143695	3.758945647	3.896212554	3.864992489	3.56058434	3.528433483	3.826922176	3.560112742	3.99165483	3.763582645
hsa-miR-7975	17.02296558	17.03693182	17.03693182	16.91408683	16.81063377	16.83895898	16.93009622	17.03693182	16.06450307	17.02001036	16.8838989	16.9416494	16.96212482	17.03693182	16.79274771	16.8507053	17.03693182	17.03866239	16.98800026	17.29601411
hsa-miR-7977	15.33905191	15.4143163	15.37636866	15.33521773	15.31624734	15.17577448	15.34243705	15.38018459	13.32863988	15.76706648	15.64883804	15.61329182	15.31624734	15.34243705	14.99443675	15.34243705	15.76706648	15.61329182	15.36933237	16.13860677
hsa-miR-8060	3.162164079	3.194512484	4.334015286	3.408492987	2.913011467	3.541205767	3.215507092	3.513382131	4.572096839	3.44205712	3.378026236	3.495081608	3.233890959	3.907285619	4.315587961	4.196933765	3.422502417	4.196982813	3.402722716	2.95013218
hsa-miR-8063	4.168526471	4.230677467	4.993341557	3.986957574	4.220399691	4.378520344	3.958402976	4.332331464	5.079606947	5.075312836	4.42465339	4.4594342	4.434056358	4.925139786	5.037591323	5.007446755	4.508942308	4.994109358	4.226657791	4.143794098
hsa-miR-8069	13.82488459	14.22064024	14.25007596	13.88722551	13.77802628	13.87101082	14.21318181	13.82902836	15.39406097	14.65694876	13.70464967	13.84909124	13.64549638	14.05062296	13.82280556	13.93583807	13.508311	13.67733429	13.81684233	12.03340637
hsa-miR-8072	4.047767734	4.255669569	4.54841857	3.85851535	3.665636093	4.243544506	3.840269959	4.064327905	4.138049614	5.091587575	3.938122964	4.067576621	3.965052506	4.074142949	4.296154773	4.188210145	4.000116458	4.392511408	4.095047986	3.039367212
hsa-miR-8485	5.041245615	5.048378652	5.386985898	4.834531841	5.053095416	5.236069644	4.204598867	4.383674376	5.550929786	6.085939918	4.655603138	4.805332004	4.523913906	4.84355411	5.366449784	5.442383515	4.572482335	5.661552012	4.737325813	4.305028491
hsa-miR-874-3p	4.999882546	5.21700924	5.407052354	4.948640689	5.338605528	5.219732928	5.393940307	5.270797123	5.061726104	5.595492958	4.930073041	5.532762679	5.351003186	5.315581547	4.857330249	5.38401238	5.23158343	5.015222648	5.480015878	4.811809032
hsa-miR-885-5p	8.790444837	8.946444244	9.292036391	8.725562897	8.904917222	8.677749472	9.033946296	9.130297922	8.028334706	9.11260549	8.083144211	8.482826072	8.560811853	8.588815915	7.949399598	8.522468713	8.191085774	8.670938223	8.537343988	6.421238792
hsa-miR-92a-3p	8.571343637	9.199235167	8.99933655	8.5060166	8.696817417	8.804604859	9.051749233	9.089177811	8.545337206	9.999812275	8.639893637	8.592594051	9.058129952	8.730138973	8.12900233	8.993052733	8.551700178	8.730957451	8.652968577	7.557012057
hsa-miR-92b-3p	3.519412943	3.462955804	3.457271587	3.347131526	3.56507698	3.109084599	3.830721249	3.492975135	3.053711367	3.954018022	3.623133947	3.508210523	3.650304103	3.648518077	3.246826175	3.31430153	3.683684473	3.259206659	4.007874591	3.61346928
hsa-miR-933	3.254978432	3.269814443	3.279046071	3.212730903	3.464222706	2.958040476	3.624312677	3.354735384	2.919355817	3.49732452	3.403092033	3.18568241	3.390199504	3.388724119	3.035206983	3.237294425	3.316975199	3.153189894	3.646577071	3.16111761
hsa-miR-93-5p	6.907229443	7.140106468	6.881974251	6.844501723	6.726930554	6.574521025	7.205760264	7.356917616	5.724007555	7.430893166	7.425904546	7.515639475	7.580681583	6.984162419	6.921468146	7.398489904	7.457192098	6.716826134	7.217201577	5.927618607
hsa-miR-937-5p	4.826662343	5.109696385	4.514141495	4.441960111	4.574748731	5.199716551	5.142001367	5.053506774	5.592239911	5.504621709	4.25120809	4.445362519	4.343387833	4.878083353	4.485389461	4.243087348	4.451802769	5.301098612	4.311068622	4.14694885
hsa-miR-939-5p	5.540106108	5.79327705	5.14532075	5.140213304	5.434832992	5.558468061	5.342462107	5.772388563	6.829119345	6.020636661	5.022765013	5.385929723	5.352681709	5.464633677	4.775909924	5.16449453	5.317436747	5.261749811	5.255782715	5.198354686
hsa-miR-940	5.553613615	5.873919398	6.304424527	5.185913969	5.875681002	5.868355839	6.025302117	5.853889276	6.173410038	6.170174946	5.849580903	5.650116866	5.68185263	6.092142326	6.134885779	6.103369292	5.762144344	6.031565352	5.714093476	5.542026581
hsa-miR-95-3p	4.101082211	4.403843301	3.622196339	4.998055172	5.627840133	3.797820693	4.627923113	4.783821494	3.243586566	3.710774902	4.941131645	4.279551554	4.701611398	4.258959015	6.231996464	4.524770991	6.013981504	4.01771764	4.675129802	8.149906506
hsa-miR-96-5p	3.182523202	3.121408814	2.766936002	3.383458097	3.288220025	2.75969535	3.087884854	2.87245905	2.716826467	2.751019377	4.302472233	3.150666695	3.558591492	3.014399561	3.339302011	3.029056925	3.9594993	2.714050077	3.71799283	4.856813085
hsa-miR-98-3p	3.461324047	3.747117332	3.307305652	3.503125476	3.758757017	3.211761375	3.890313123	3.806894514	2.945962875	3.649414988	3.619907338	3.740586235	3.69990565	3.627993663	3.325652403	3.495425038	3.527174905	3.330610792	3.696744161	3.474606217
hsa-miR-98-5p	5.61698854	5.65277976	4.71155922	5.664431723	5.63458658	5.468239355	5.511688368	5.751329234	4.422341905	3.628781687	5.830778693	5.788094261	5.997206762	5.725706698	5.416632102	5.718005486	5.858380139	5.169725471	5.903643766	6.429444989
hsa-miR-99a-5p	9.749353423	9.99583984	9.799691007	9.813828875	9.892599162	9.758734216	9.998203079	10.1759056	9.240717011	10.03609083	9.367068671	9.872464128	9.774965733	9.673190741	9.345215839	9.575950881	9.453625587	9.705807417	9.797664636	10.28057549
hsa-miR-99b-5p	5.228882767	5.059962857	4.446559697	4.815243248	4.88717444	4.621874388	5.357652671	5.241212447	3.849369723	3.576821451	6.315126278	5.385252275	5.986915632	5.164488348	5.000608352	5.633926199	5.54701869	4.683395758	5.772355288	6.163618955

Abbreviations: IT, immune tolerant; IA, immune active. Values represent normalized signal values for individual samples.

Table S10. Normalized expression values of 559 hepatic miRNAs in liver tissue from chronic hepatitis B patients (IC and ENH phases).

Sample ID	IC_1	IC_2	IC_3	IC_4	IC_5	IC_6	IC_7	IC_8	IC_9	IC_10	IC_11	IC_12	IC_13	ENH_1	ENH_2	ENH_3	ENH_4	ENH_5	ENH_6	ENH_7	ENH_8	ENH_9	ENH_10
	IC-LYM_S13	IC-CZY_S4	IC-YSR_S18	IC-YFL_S16	IC-WCY_S14	IC-WJY_S2	IC-WL_S3	IC-WZH_S15	IC-ZBL_S17	IC-LXL_S5	IC-CX_S10	IC-WJQ_S1	IC-XYJ_S19	ENH-LJQ_S15	ENH-CJH_S3	ENH-LBL_S4	ENH-ZZS_S9	ENH-XXY_S2	ENH-CRF_S8	ENH-ZMM_S5	ENH-ZXD_S1	ENH-DW_S12	ENH-GCF_S17
hsa-let-7a-5p	12.771331	12.912695	12.932426	12.917935	12.859395	12.764246	12.925898	12.87678	12.739883	12.949737	12.398336	12.637104	13.004331	12.8220637	12.7428019	13.0051216	12.7985307	12.9142124	12.9612648	12.8470523	13.0716069	13.0075188	12.5011378
hsa-let-7b-3p	4.0520033	3.9343823	4.3288885	4.0262719	4.2590871	4.1811179	4.0559501	4.0024924	4.2389297	4.2685993	3.6370358	3.8131257	4.1446346	4.09371084	4.44805327	3.90219143	3.8436056	4.05485354	4.35636227	3.96872277	4.20928274	4.10745457	3.62969102
hsa-let-7b-5p	11.789686	11.88965	12.042684	12.000581	11.944134	11.908132	12.014898	12.095789	11.885732	11.863117	11.61583	11.918334	11.921455	11.9343852	11.8780258	12.1884678	11.9498333	12.0593192	11.8873931	11.7214961	12.2034302	12.0449202	11.5380159
hsa-let-7c-5p	10.958586	10.951739	10.841786	10.921957	10.939499	10.840013	10.873958	11.096816	10.939675	10.902368	10.72852	10.863739	10.936847	10.8924127	10.880292	11.000896	10.8128169	11.0412024	10.9265786	10.9172115	11.1275725	10.961602	10.5355993
hsa-let-7d-5p	8.9460053	9.0695369	9.1095939	8.9429813	9.0842113	9.0389826	9.0761579	9.0181614	9.08732	9.1024439	8.7884416	8.8861328	9.1505027	9.14347716	9.11859122	8.99207007	8.81175506	9.06321441	8.97229042	8.9480778	9.20600083	9.0184543	9.7680451
hsa-let-7e-5p	7.8290439	8.1220265	8.130583	8.1139754	8.1100355	8.0230458	7.9231599	8.0531311	8.0856455	7.9481923	7.3804578	7.9637662	8.1953286	8.20563116	8.2768665	8.3188327	7.98498309	8.33551874	8.02889576	7.64428352	7.92700308	7.98437114	8.24217714
hsa-let-7f-1-3p	4.1408323	3.9072993	4.1693267	3.855841	4.0337524	4.0010855	3.9165527	3.8821042	4.2209886	4.1832105	3.5100991	3.8264435	4.0633396	4.14890702	4.18649271	3.79852259	3.69922225	4.15076614	4.07682311	3.97758261	3.95141407	4.12966202	3.65787337
hsa-let-7f-5p	12.110849	12.120225	12.121305	12.193403	12.195592	12.116608	12.155098	12.193059	12.090816	12.159734	11.794223	11.989952	12.238998	12.2797736	12.221349	12.34645	12.1845468	12.3255847	12.1734072	12.1995127	12.3224649	12.1691446	12.2904266
hsa-let-7g-5p	11.05272	11.123123	11.162965	11.209104	11.121889	11.155112	11.251981	11.177491	11.199703	11.188105	10.718885	11.071869	11.054202	11.2690929	11.335259	11.1822351	11.3374841	11.3167684	11.2341525	11.1675749	11.2544464	11.2596055	10.6459637
hsa-let-7i-5p	9.2928226	9.5483986	9.5930419	9.3571859	9.3882149	9.3585319	9.339771	9.536236	9.6694274	9.4066983	9.2370762	9.3250329	9.4889886	9.84311358	9.8632361	9.53690714	9.4417353	9.71775243	9.3156227	9.21942666	9.50909256	9.5340353	9.94987483
hsa-miR-100-5p	9.4528616	9.5603502	9.5471555	9.5563596	9.453945	9.3457456	9.3339436	9.320988	9.4390396	9.5323126	8.6070699	9.1164809	9.5340889	9.58826505	9.71302225	9.33953457	9.46282605	9.62677497	9.64401861	9.32820014	9.49381853	9.52854353	8.95843941
hsa-miR-101-3p	9.5971391	9.318688	9.180901	9.1739747	9.3435567	9.2359435	9.3432567	9.015793	9.3155169	9.4690503	9.1926806	9.1727033	9.5401414	8.89083211	8.95833816	8.83733323	8.69083965	8.77599336	9.24778518	9.3133052	9.01666322	9.34127056	9.57627857
hsa-miR-103a-3p	9.7678152	10.100627	10.043637	10.094009	10.216682	9.9967167	10.266136	9.9906959	10.223435	10.0551	9.4567825	9.8294889	10.047345	10.1567047	10.1114889	9.5941926	9.78440048	10.0003798	9.94973872	9.94729433	10.1105053	10.0537129	8.13556124
hsa-miR-106b-5p	8.1955734	8.3059886	8.2518885	7.8587836	7.959762	8.1228101	8.2075909	8.0416999	8.3782008	8.2014687	7.3419556	7.7341566	8.1688978	8.32419106	8.3757738	7.93221688	8.06926907	8.20314955	8.20339199	7.92516503	8.10827315	8.14643612	7.30094586
hsa-miR-107	9.8415396	10.032148	10.020103	9.8775198	10.085347	9.8641359	10.188844	9.8964674	10.107184	10.138492	9.2739939	9.8212655	9.9303872	9.9021588	9.89163103	9.54538385	9.8435195	9.89981173	9.89131941	9.87497558	10.1527333	9.97291426	8.08354512
hsa-miR-10a-5p	7.4984358	7.622774	7.7063486	7.7679844	7.5559272	7.5845742	7.3412048	7.5834942	7.4692225	7.5829682	7.0297034	7.3345635	8.0586592	7.80900431	7.96785212	7.88325823	7.77122133	7.82963143	7.55736198	7.25225491	7.52737239	7.82989267	7.56254467
hsa-miR-10b-5p	4.754295	4.8020549	4.896555	4.5738038	4.6529153	4.9430715	4.1207963	5.2852078	4.2752841	4.7862148	5.6731808	4.6399947	5.5077341	5.81157346	5.59208777	6.65260468	5.0247032	5.31455208	4.81411347	4.65451387	4.72035391	5.28559337	7.93391891
hsa-miR-1181	4.1931996	3.7744855	4.6109233	3.488472	4.1542115	3.8875217	3.922109	4.1450648	3.8555058	3.7063387	5.2902142	5.2854168	3.8693881	4.46517656	4.55331904	3.94975204	4.06610891	4.3993154	4.21380512	5.23737116	4.66529577	4.44317369	4.59361639
hsa-miR-1185-1-3	3.027692	3.3152691	3.4913208	3.2410172	3.4178028	3.3534241	3.1248529	3.378826	3.1193002	3.2703347	4.3323313	4.4515712	3.377699	3.60910274	3.41768474	3.90053961	3.93540408	3.58902338	3.40393551	4.16608471	3.79211384	3.56434177	4.14227078
hsa-miR-1202	7.7350112	6.9909463	6.963325	7.1268472	7.345462	6.9284265	6.9209625	7.1320024	7.0164378	6.8756398	7.3918082	7.1703828	6.9332274	7.03425309	6.85800222	8.15708328	7.62702073	7.43459881	7.25211845	7.03272083	6.99312285	7.08538493	7.82534904
hsa-miR-1207-5p	6.348982	5.9359495	6.0145075	6.7810149	6.3791031	6.7859096	6.7679404	6.7553716	6.5436684	6.183928	7.0315726	6.2877055	6.8207165	6.20451417	5.98212853	6.77350384	6.26842547	6.00941188	5.96791411	6.51231815	6.70546631	6.17092087	6.37995288
hsa-miR-122-3p	11.337023	11.385647	11.153448	11.119763	11.252739	11.261251	11.29555	10.959681	11.352106	11.422774	10.904425	10.97996	11.600367	10.8212154	10.9122925	10.8783815	10.8892699	10.777344	11.2282568	11.4378622	10.9589039	11.112179	6.81720314
hsa-miR-1224-5p	4.0221654	3.7843362	4.1399021	4.3618844	4.0219067	3.7469146	4.7053359	4.426371	4.1541902	4.104562	5.0382074	4.3723631	4.4841156	4.10948768	4.09291205	4.56386479	5.08261615	4.32124926	4.16249673	4.80126981	4.60605313	4.69278349	4.61691084
hsa-miR-1225-5p	6.8014214	6.5625076	7.094211	7.072018	6.9901045	7.3677976	7.3022209	6.8551526	7.2039521	6.7993734	7.5295278	7.360183	7.1551541	6.55523481	6.49739756	6.81897231	6.53139823	6.4085497	6.55859813	6.81428389	7.11725562	6.67021826	6.48864335
hsa-miR-122-5p	15.407259	15.742746	15.613292	15.342437	15.342437	15.613292	15.613292	15.188662	15.613292	15.622566	15.484781	15.342437	15.632082	15.3424371	15.3424371	15.6132918	15.3424371	15.1886624	15.3424371	15.7749631	15.5555814	15.3424371	11.0139234
hsa-miR-1227-3p	3.1682574	2.976781	3.0779215	3.2330111	3.3691481	3.2955659	2.9868419	3.0565115	3.1756729	2.9747419	2.9350174	3.2545453	2.941822	3.20499036	3.34902054	3.07965142	3.07394843	3.36882096	3.37319321	3.12657581	3.33645378	3.23614502	3.06438349
hsa-miR-1227-5p	3.5974109	3.256891	3.0675627	3.7026972	3.5764836	3.7926269	4.0209366	3.4107892	3.7098326	5.5951622	4.7839609	4.0932641	4.9034773	3.46706985	2.75645783	4.01805708	3.45208779	3.23126183	3.14497145	4.41900566	3.63255099	3.95229699	4.63547038
hsa-miR-1228-3p	4.9825543	4.9714385	5.1871221	4.9598479	5.1656697	5.0118366	4.9422629	4.8926422	5.338425	5.0790004	4.7052485	5.1187625	5.3414184	4.90681856	5.01161845	4.55525039	4.64920072	4.94619	5.09972228	4.79091255	5.04712595	5.04861298	4.40787426
hsa-miR-1229-3p	3.5945243	3.3544945	3.621484	3.2185755	3.467157	3.4929067	3.3824401	3.2413128	3.4046278	3.4500443	3.1773908	3.4604909	3.1882094	3.60126845	3.68766858	3.2862585	3.39369549	3.5277512	3.33356626	3.46466923	3.37091539	3.26862549	3.51104611
hsa-miR-1229-5p	3.9373575	3.2440756	4.1018228	3.5513485	3.8850442	3.9935453	3.782906	3.4057396	3.5471941	3.4675756	4.3486172	4.2008979	4.1225449	4.49888882	3.74323127	5.39657659	5.11041299	3.81950125	3.75023327	4.61807693	3.9502328	3.8515777	5.48193017
hsa-miR-1233-5p	3.8920187	3.4958972	3.3601242	4.0334651	3.9574456	2.9670481	3.5247286	4.1839135	3.2403656	3.2730536	3.9705273	3.9785538	3.3392489	4.21080185	3.77829326	3.62020379	4.22158611	4.13787406	4.25934534	3.80739089	3.99898596	4.1790015	4.16937534
hsa-miR-1234-3p	5.1399084	4.55236	5.1109095	4.7814424	5.0738345	4.9938209	4.7680361	4.8964963	5.0887014	5.0894432	4.739594	4.9841203	5.0195479	4.70835473	4.80748832	4.53494791	4.53001924	4.93148017	4.91770133	4.84948003	4.96049187	4.89565248	4.44743447
hsa-miR-1237-3p	3.9959745	3.5197864	4.1454536	3.7488958	4.0596429	3.7258764	3.7533169	3.6212911	3.8893455	4.0316641	3.6265692	3.807573	3.9251737	3.67224957	3.89960951	3.52140611	3.7114081	3.93911163	3.92634808	3.79435287	3.88479603	3.91057811	3.48091622
hsa-miR-1238-3p	4.6886119	4.318943	4.9149398	4.5832453	4.898541	4.6727813	4.4670175	4.5326373	4.9495678	4.841747	4.05852	4.5806985	4.755132	4.61834307	4.75237305	4.25583109	4.2339975	4.80383296	4.69325505	4.54782246	4.60596976	4.70237533	4.12436204
hsa-miR-1246	4.6114529	4.4196854	4.7924004	4.5722421	4.5748899	4.4373008	4.3337448	4.4224297	4.4392806	4.4708264	5.0708976	5.1384146	4.5301893	4.90560815	4.84138663	4.99089549	5.09313871	4.57490221	4.83113695	4.95281885	4.71968805	4.89424851	5.75562463
hsa-miR-1249-3p	4.9249997	5.0657774	4.9531708	5.3234476	5.161017	5.2463475	4.9672459	5.2827251	5.3548168	5.2085493	5.5015193	5.5902091	5.7522142	4.6130482	4.66646729	4.82009808	4.79244171	4.92339123	5.066451	4.88373096	5.02168909	4.86227894	4.53604806
hsa-miR-1249-5p	3.2743697	3.4499154	3.7043839	3.0738401	3.5328822	3.484539	3.3086407	3.4518942	3.247958	3.5287895	3.7631751	3.9951721	3.4742172	3.47090617	3.36987084	3.26711467	3.42900777	3.47322985	3.47620041	3.44498949	3.5415539	3.43084308	3.52739083
hsa-miR-125a-3p	4.2896738	4.068775	4.3223378	3.6758712	4.0700045	3.9507756	3.8416456	4.110928	3.7518463	3.759104	4.8517959	5.0004703	3.7636105	4.60232045	4.35876196	5.26772987	6.24465048	4.44475144	4.1423563	5.01966451	4.44933868	4.77525837	4.65836854
hsa-miR-125a-5p	5.7458169	5.9812761	6.1672898	6.1239412	6.2152635	5.9962218	5.5925805	6.0305266	6.0394218	5.9590657	5.2808896	5.6489964	6.1489005	6.44239303	6.49359865	6.24929526	6.00953072	6.37035571	6.11979971	5.47101885	6.03964113	6.26985268	6.14341358
hsa-miR-125b-2-3	3.7252261	3.8180912	3.4161074	3.4926407	3.8401517	3.4752092	3.3871109	3.7699625	3.9421301	3.8082397	3.57524	3.5246513	3.6006582	3.76956556	3.84726356	3.8122209	3.84052857	3.96188639	3.9771634	3.92681052	4.08801068	3.98871663	4.09863342
hsa-miR-125b-5p	11.054076	11.069176	10.941174	10.826906	10.907345	10.926439	10.944147	10.930092	10.959894	11.171946	10.49095	10.776194	11.23023	10.7272777	10.6936592	10.7523306	10.6323935	10.6700273	10.9691814	10.7231579	10.8689426	10.9990065	10.8528057
hsa-miR-1260a	11.378245	10.958114	10.669629	11.309299	11.425405	10.900169	10.909018	11.391534	10.904457	10.833477	10.778322	11.314531	10.877788	10.8359625	10.8352335	10.5508073	10.9204885	11.1613569	11.2677943	10.3894059	10.8299133	11.0984815	10.3122622
hsa-miR-1260b	9.5773625	9.2819089	9.0949065	9.5014623	9.7078158	9.1800516	9.1814694	9.8061076	9.267646	9.1429341	9.0335582	9.6979936	9.1349162	9.19196256	9.30862603	8.88593758	9.22101697	9.66666377	9.46440929	8.74203419	9.14746705	9.36520082	9.20005256
hsa-miR-126-3p	10.781887	11.017608	10.717663	11.290352	10.906848	10.809734	10.813061	10.794374	10.769818	10.777739	10.715193	10.675189	11.059272	10.9394862	10.9469513	11.0614854	10.9412148	10.8224324	10.7473467	10.8379295	10.7670362	10.7221802	10.3705053
hsa-miR-126-5p	5.542933	5.6965635	5.2018981	5.7904818	5.7967872	5.0610472	5.2491693	5.6045371	5.413732	5.4672319	5.0917793	4.9851187	5.8372607	5.76706802	5.91198055	5.84656827	5.66222474	5.77843239	5.76204619	5.70975007	5.70388909	5.81013237	5.67199801
hsa-miR-1268a	5.3567586	4.9353613	4.8868838	5.7220728	5.2807445	5.6349971	5.5300046	4.9846771	5.3344505	4.8248834	6.0827205	5.4193835	6.2337862	4.9075684	4.91806523	5.10643063	4.82936088	4.97365949	4.9132203	5.9558928	5.05299785	4.78191299	5.28639822
hsa-miR-1268b	4.9380977	4.5737745	4.4734147	4.7546345	4.7381301	4.3707028	4.5122922	4.8639695	4.3605419	4.2746269	4.8346088	4.8891508	4.985804	4.9572286	4.68703897	5.71907464	5.64975633	4.96491397	4.81296681	5.01493988	4.675889	4.90624151	5.39122049
hsa-miR-1271-5p	3.3280718	3.1356385	3.1274704	2.9297817	3.2435041	3.1758195	3.0391622	2.9847167	3.3617463	3.2978359	3.3393296	2.9954308	3.0239245	3.24070572	3.52194781	3.46843865	3.21430828	3.25842376	3.4660741	3.25536111	3.28247677	3.38113067	5.14682579
hsa-miR-1273e	3.3913187	3.3174518	3.8815782	3.2137077	3.5540418	3.5670983	3.52093	3.3133851	3.0009901	3.4989393	4.7288548	4.6243755	3.6509553	3.60691273	3.56216152	3.78777405	3.68656209	3.7151176	3.50123399	4.34673612	3.85681094	3.57716635	3.90704423
hsa-miR-1273f	3.1055667	2.9674171	2.7445789	2.7840767	3.0194866	2.7632859	2.6782844	2.7669231	2.5954969	2.7896861	3.4921246	3.5779737	2.5975266	3.11740945	2.73546438	3.90418201	3.93695487	3.02317625	2.72640862	3.87724551	2.93725275	2.8400715	3.45089175
hsa-miR-1273g-3	10.662317	10.66802	11.011613	10.554472	10.645409	10.654476	10.605903	10.552952	11.147362	10.722306	10.585237	10.78804	10.415103	10.847091	11.1555262	10.4959625	10.3804252	10.9767813	10.7870812	10.5367671	10.5721614	10.5080257	10.5857693
hsa-miR-127-3p	3.8809584	3.7818233	3.5591909	3.8335012	3.8344793	3.7418999	3.7413811	4.0444669	3.5724421	3.569738	3.6821119	4.2012571	3.8051521	4.20030915	4.8163599	4.36977458	3.91350743	4.64900236	4.460917	3.60467208	4.32247047	4.21661087	5.51550192
hsa-miR-1275	4.9511113	5.1080204	4.8708758	4.9914745	4.9445895	4.7911562	4.6908097	5.0639634	4.8850347	4.6920334	4.607636	4.7958152	5.0627344	4.88107723	4.85609145	5.03870729	5.18098926	4.92961046	4.53616188	5.07054229	4.67927741	4.75633668	4.49703287
hsa-miR-1281	4.6316448	4.3550086	4.7875456	4.3706707	4.6143076	4.4857668	4.2695539	4.4044447	4.5577754	4.6690098	4.0463926	4.3300194	4.581434	4.36043692	4.60477686	4.10869264	4.16524096	4.61848393	4.61004783	4.34718225	4.47065043	4.63684895	4.08049062
hsa-miR-128-3p	4.9374967	5.0383912	4.223613	4.5889704	4.5864189	4.8822086	4.6609423	4.7840497	5.2433347	4.6072851	5.2110296	4.2437018	4.8630867	5.56428463	5.52918156	5.13617086	5.01931655	5.63830102	5.2798617	4.95765866	5.47502823	5.36827845	6.88019634
hsa-miR-1288-3p	3.5331866	3.2754427	3.5022406	3.2092166	3.44109	3.3561496	3.1237936	3.4136421	3.5937059	3.2382243	3.5424233	3.4120043	2.9353962	3.92366499	3.82098237	3.45274594	3.5300483	3.76448117	3.66021519	3.80707303	3.66375293	3.91450911	3.66038983
hsa-miR-1290	3.6334356	3.3979376	3.2672609	3.300038	3.600785	3.1187661	3.0810496	3.2612823	3.2101365	3.4274817	3.6255377	3.3945044	3.0802884	3.84298652	3.63737251	3.45834618	3.575661	3.43539109	3.57430951	3.57531564	3.5144689	3.55887518	3.41238062
hsa-miR-129-1-3	3.4696851	3.6333171	3.4885945	3.1453022	3.5818797	3.267753	3.4077123	3.4812941	3.365704	3.4597687	3.1152054	3.5515199	3.2639881	3.32779716	3.38196215	3.16806569	3.24387125	3.62682524	3.35393427	3.37652487	3.5367898	3.50231321	3.20955531
hsa-miR-129-2-3	3.6098435	3.6074566	3.5676886	3.3135338	3.6355518	3.3560496	3.3541323	3.3708166	3.628915	3.4022084	3.3058604	3.7026083	3.5471941	3.39364286	3.57245352	3.2643133	3.26679427	3.56620786	3.49462879	3.51221909	3.50696345	3.50421367	3.43989289
hsa-miR-1304-3p	4.7853516	4.4203634	5.0772869	4.6155519	4.8532112	4.7008132	4.5276966	4.6821847	4.9806307	4.9068568	4.0417117	4.600601	4.6587957	4.61454791	4.74052827	4.2528177	4.25378612	4.70585329	4.7812016	4.50785793	4.74276311	4.71591743	4.12082666
hsa-miR-1305	4.8828274	4.9886343	5.0654119	4.6087572	4.9156789	4.5756542	4.7055149	4.6523617	5.0177147	4.6638677	4.7451477	5.028829	4.5750719	5.28079502	5.14531723	4.63133594	4.86114948	5.05232517	5.06367968	5.15370077	4.98164291	5.16434463	4.7466752
hsa-miR-1307-5p	2.9884891	2.7394346	2.7005472	2.9674783	3.1654458	2.9719498	2.8165635	3.1029308	2.6581672	2.869228	3.4132481	3.268795	2.9122261	3.08421033	3.14471506	2.94855475	2.99124946	3.48061678	3.40021221	3.1375323	3.28216948	3.087323	3.21445439
hsa-miR-130a-3p	8.9804621	8.9978358	9.1948247	8.8813369	8.8035733	9.0912338	8.9003841	8.788433	9.0402397	8.9081772	8.3612295	9.1071481	9.0578069	9.17738031	9.11748913	9.00947961	8.76627778	9.00527575	8.87003898	8.40759341	8.83288306	8.90177891	8.21853138
hsa-miR-130b-3p	5.2479146	5.4721216	5.2527291	5.1650402	5.3565323	4.7947163	5.00114	5.3579562	5.5292864	5.4643297	5.0370044	5.3861216	5.097578	5.35802866	5.65097559	5.59259507	5.21971422	6.14798627	6.68329174	4.93852971	5.80600499	5.36774828	3.72004928
hsa-miR-132-3p	3.9025206	3.7685434	3.5335018	3.4762923	3.247598	3.2952071	3.1258174	3.6669648	3.4122014	3.4881371	3.1786264	3.2205008	3.3970912	4.42810786	4.73025331	4.07733466	4.17410764	4.42081392	3.80151653	3.21976899	3.95965888	4.05525998	3.87975412
hsa-miR-133b	3.8008355	3.8315547	3.9759067	3.0422094	3.608638	3.355626	3.6702697	3.6817672	2.9487387	3.194317	12.452797	3.3404712	4.2600242	3.98882757	3.65669756	9.81674329	3.71895601	3.66429905	3.39210506	3.1942017	3.21790235	4.0273507	15.530754

hsa-miR-135a-3p	3.5769262	3.2780209	3.3326888	3.0813205	3.4259833	3.2990531	3.288885	3.3044126	3.173382	3.1806803	4.2656611	4.1808194	3.2468927	3.88272788	3.51562046	3.47180837	3.62247242	3.58830859	3.50403653	4.13116347	3.53700427	3.54439064	3.96780013
hsa-miR-135b-5p	3.0622726	3.1129872	3.351268	2.9288431	3.1884975	2.8982359	2.962447	3.2566764	3.1551419	3.1910956	2.8323199	3.0484834	3.1733091	3.40377039	3.64777231	3.88571361	3.27357785	3.18654466	3.57220445	2.90044958	3.26318731	3.21910009	3.24708267
hsa-miR-136-3p	3.3063185	3.2997916	2.8983895	3.3518654	3.5691939	3.0625817	3.0413948	3.365632	3.1538897	3.1030195	3.4224683	3.0853192	3.3276752	3.55111894	3.76737219	3.82277322	3.2579594	3.82012699	3.74592198	3.5032787	3.57138565	3.54382073	4.66603436
hsa-miR-136-5p	4.2762811	4.1087529	4.046111	4.1334314	3.9538617	4.0689857	3.8031405	3.9568051	3.9051629	3.6783646	4.0501394	4.1718837	4.1947124	4.42339361	4.86609345	4.46958388	3.6397486	4.29163023	4.4833579	3.92299836	3.93257737	4.05541835	5.63447036
hsa-miR-139-3p	4.3191953	4.1269098	4.0695577	3.9729655	4.1060733	3.8875433	3.9705185	4.1253574	4.0713784	4.2266397	3.6972364	3.8399275	4.3413744	4.00407794	4.07978906	3.89788432	3.91410292	4.133277	4.05625489	4.10752606	4.15867961	4.13300321	3.61196636
hsa-miR-139-5p	4.280951	4.1295497	3.929111	4.2530316	4.1744689	3.898391	4.0510299	4.0629644	4.0792576	4.1991167	3.7835082	3.8077502	4.2154999	3.96585683	4.16999505	4.0294805	3.92943817	4.0046162	4.06429821	4.04214035	4.08809566	4.14069823	3.50328064
hsa-miR-1-3p	4.5227419	4.441801	4.5413073	2.9376707	4.1328914	3.8896707	4.2832672	4.4913066	3.1554775	3.8852463	13.174607	4.0415568	5.4389016	4.69916928	4.27486478	10.7186857	4.22292985	4.30320335	3.80298733	3.64868337	3.63685295	4.61687072	15.9584356
hsa-miR-140-3p	6.538307	6.5127155	6.7861575	6.2960136	6.3464288	6.4880177	5.9813528	6.7005121	6.6225752	6.3192002	5.7041921	6.1549495	6.9924119	6.86406708	6.81531747	6.48357692	6.467378	6.60374998	6.68169867	5.79086629	6.45120441	6.67220151	6.52975736
hsa-miR-140-5p	6.3010324	6.523189	6.7161051	6.5016885	6.4973597	6.5122222	6.2010894	6.6600885	6.6675266	6.4656123	6.0355928	6.2040176	6.8483458	6.76237044	6.8328526	6.55358592	6.33033371	6.68244617	6.64091004	6.0211744	6.51292329	6.69818554	6.49297919
hsa-miR-142-3p	9.5589855	9.3654146	9.824103	9.9042873	9.0821266	9.6083921	9.1539647	9.7128508	9.7345912	9.1027676	8.9624742	8.7979155	9.6215821	10.744257	10.4829307	9.79526797	9.97303963	10.2606414	9.67755313	8.8981787	9.52352628	9.83468425	7.90240188
hsa-miR-142-5p	6.2798456	6.1576099	6.9377119	6.6875392	5.8566981	6.4871652	5.7690945	6.5058139	6.7913221	5.9910098	5.4727483	5.0668671	6.4540607	7.67789866	7.48417448	6.71436858	7.13081608	7.28808751	6.82932738	5.69421806	6.6423549	6.97970285	5.13635911
hsa-miR-143-3p	5.4331245	5.2902611	5.4137708	5.0818862	5.4828361	5.5260761	4.8488596	5.7251726	4.8653735	4.8917022	4.6198928	5.3050784	6.7542454	6.2692176	6.05059681	5.70903646	5.24419887	5.77474436	5.20241254	4.98684392	5.09998471	5.47124459	5.30995006
hsa-miR-144-3p	7.7103152	7.1817343	6.9519022	6.4953107	6.9949333	7.4655702	6.2059898	6.8451077	8.2224178	7.3027455	7.0206271	6.365102	6.9795828	6.90203127	7.58085392	6.94093525	6.0717615	6.56423111	7.04298225	6.82262788	6.5396119	6.62361064	9.23955926
hsa-miR-144-5p	3.8875762	4.1209397	3.414854	3.3488276	3.9802984	3.9804159	3.5868684	3.5780098	4.4150345	4.1326256	3.3344961	3.1884364	3.62511	3.51485985	4.2332675	4.02514675	3.41739263	3.42805867	3.88516763	4.1921933	3.71969974	3.52115509	5.11419893
hsa-miR-145-3p	3.6355901	3.4740602	3.7371285	3.4170665	3.5824956	3.5731224	3.2508252	3.8126479	3.3615509	3.2277354	3.1226108	3.6300635	4.3361839	4.41911607	4.18775785	4.04579148	3.58169919	4.02718761	3.56960313	3.67296829	3.69078013	3.76140466	3.75807576
hsa-miR-145-5p	8.5456117	8.7538988	8.9574392	8.7254509	8.8347056	9.0384892	8.4319957	8.9741326	8.3741318	8.5371282	7.6822508	8.8864778	9.5882148	9.19441298	9.08052709	8.77193386	8.3231474	8.69478458	8.38854776	8.42041676	8.39467051	8.69884646	8.18284888
hsa-miR-146a-5p	6.5611651	6.434317	6.9233184	6.709148	6.2664264	6.6596046	6.2472944	6.7747626	7.053899	6.2562922	5.5799776	6.00607	6.5346716	7.72835626	7.70630919	6.66606983	7.02054501	7.17056023	7.18194278	5.72708957	6.89920773	6.68032685	4.13440131
hsa-miR-146b-5p	7.4334754	7.7538921	7.4417388	6.7854614	7.1546904	6.7759461	6.9526177	6.920261	7.9481591	7.2472546	5.7230098	5.4843193	7.471368	8.01897338	7.7783175	7.08133733	7.97450423	8.46155841	7.78974902	6.74378894	7.59317205	7.77864261	4.54097615
hsa-miR-148a-3p	11.677573	11.837528	11.546094	11.732643	11.966418	11.338273	11.738693	11.401346	11.374852	11.941866	11.423814	11.607586	11.759451	11.1489351	11.0876321	11.4607041	11.2796766	11.0828163	11.6911594	11.7873968	11.5566848	11.7821729	8.25969134
hsa-miR-148a-5p	2.8086361	2.8237749	2.8370282	2.6554718	2.928418	2.7846749	2.6914668	2.7443745	2.7601556	2.757895	2.7295844	2.6509387	2.7422366	2.81078929	2.88860049	2.77726279	2.54652548	2.73115241	3.01290344	2.95974295	2.89385678	3.09432519	2.60611285
hsa-miR-148b-3p	5.3299073	5.4026606	5.1837844	5.1218721	5.2483456	4.9176681	5.0613743	5.4533258	5.2810767	5.3292942	4.9741191	4.9281163	5.5306791	5.37763798	5.61966422	5.40947112	5.22260907	5.59303388	5.89630226	5.36491514	5.79188837	5.69561295	4.72192781
hsa-miR-149-5p	4.1263792	3.8796668	4.2326954	4.1247675	4.3249441	4.1733055	3.8435698	4.0872719	4.2464918	4.2875803	3.6940248	4.0146197	4.1216296	4.02858019	4.27462974	3.80820132	3.88026875	4.30455695	4.42774987	4.04005915	4.22565903	4.14362708	4.03347648
hsa-miR-150-3p	3.2034854	2.9040832	3.1070377	3.0187395	3.2478921	2.9557261	2.9402195	2.8758977	2.9739873	2.8920053	3.8124144	3.8560667	3.2744344	3.34917622	3.21700631	3.59775502	3.89469063	3.10573517	3.1313617	3.89185986	3.29949715	3.02708231	3.70405315
hsa-miR-150-5p	7.6730864	7.9122274	8.2793267	8.6802641	7.4727127	8.2110324	7.8341297	8.4628324	8.1506202	7.5222285	6.8185701	7.1141372	8.1103757	9.00763516	8.74467186	8.46804092	8.83005638	8.99766701	8.40627018	7.22681014	8.49239687	8.83867027	7.34595287
hsa-miR-151a-3p	5.3046665	5.4995187	5.3576951	5.4933619	5.3568952	5.1245045	5.1458639	5.3194722	5.3025978	5.483362	4.7125858	4.9325082	5.4590762	5.48447688	5.53924371	5.3048726	5.22211687	5.45494764	5.54885126	5.11275288	5.44579142	5.55492283	4.80546158
hsa-miR-151a-5p	8.3871823	8.5382648	8.2528499	8.2851314	8.4007609	8.1053822	8.4068954	8.3209226	8.0539451	8.638759	7.7220165	7.9958303	8.5403871	8.05640558	8.12312942	7.9664062	8.01564852	8.15976715	8.3630129	8.11753606	8.20022487	8.28327609	7.45383291
hsa-miR-151b	6.6697042	6.6069053	6.1632458	6.3658405	6.5166812	6.1373869	6.2986937	6.3574924	6.2184725	6.739242	5.8253624	6.0389069	6.7261316	6.44779742	6.60202857	6.29379854	6.36364711	6.56881638	6.69682209	6.48826679	6.52250154	6.609452	5.86776253
hsa-miR-152-3p	5.1017109	5.159205	4.7698901	4.9949009	5.2855005	4.9489688	5.3522593	5.4092151	4.8914591	5.1047331	4.8461836	4.4510152	5.1879239	5.18958432	5.17242694	5.24267187	5.12815698	5.30988873	5.28037257	5.19120677	5.45020018	5.35303641	5.25818206
hsa-miR-1539	4.2463233	4.1503151	4.5173578	4.1087208	4.2679823	4.3328195	4.022564	4.0247042	4.319386	4.4650145	3.7125484	4.080342	4.134873	4.08432769	4.29553393	3.80413886	3.89939237	4.21297664	4.10377624	4.01715435	4.2321851	4.19290604	3.67446689
hsa-miR-154-5p	3.3823001	3.4109104	3.2995438	3.4821044	3.6033079	3.1642782	3.1803453	3.3733184	3.4378518	3.1004533	3.0961325	3.3226793	3.4629219	3.63985156	4.00468226	3.8570702	3.46448482	3.87006914	3.84768967	3.46797395	3.64498498	3.55306751	4.08522929
hsa-miR-155-5p	5.014973	5.0265817	6.1261661	5.3802293	4.731308	4.8309233	4.5105147	5.5334704	5.2958982	4.5150801	3.992794	4.1626849	4.9206915	7.18972349	6.78869741	5.54201946	6.16661421	6.32519265	5.627749	4.53448958	5.45965711	5.76627155	3.67828971
hsa-miR-1587	4.106627	3.7974084	3.814506	3.7124569	3.9630912	3.8475505	3.9433148	3.7428111	3.7267668	3.9174356	4.2482756	4.2664622	3.685782	3.97563299	3.7567191	4.44098929	4.5112047	3.76528254	3.74004161	4.22504683	3.86745637	3.76277211	4.31740694
hsa-miR-15a-5p	9.204469	9.4868777	9.2628348	9.2448287	9.2699298	9.2370575	9.3677725	9.150763	9.5203907	9.4898829	8.8217022	9.0395806	9.2928561	9.31390274	9.40457222	8.9440098	9.1615791	8.94906474	9.45421691	9.08310401	9.13418249	9.28585839	9.00328839
hsa-miR-15b-5p	9.2464766	9.3636039	9.264941	9.268054	9.2282628	9.3242337	9.1981614	9.2491559	9.4493334	9.2944137	8.3838874	8.8345154	9.1156948	9.46560203	9.53934815	8.83741521	9.22368263	9.06625016	9.13185283	9.02404572	9.21929854	9.09990895	8.46801435
hsa-miR-16-2-3p	2.5313536	2.6944928	2.5688511	2.7032318	2.7300429	2.7866101	2.6126058	2.5111259	2.6660134	2.5403318	2.5057644	2.8297809	2.6011786	2.82417569	2.86010394	2.71138778	2.66295214	2.82025535	2.64584523	2.67931585	2.88081612	2.7806259	2.65838785
hsa-miR-16-5p	10.915354	11.041772	10.875071	11.017255	10.933174	10.84349	10.91962	10.960162	10.967022	11.00057	10.532806	10.592125	10.710657	11.3920968	11.427313	11.0893243	11.3169511	11.0455283	11.0787237	10.8848485	11.0575777	10.9720008	10.8872597
hsa-miR-17-3p	5.1152017	5.0162778	5.1877575	4.3406031	4.7388003	4.5549718	5.1206679	5.3644528	5.207919	4.8966723	4.0403113	4.5842572	5.1379555	5.01464887	4.68544702	4.69728431	4.86178005	4.80318853	5.39790257	4.84797944	4.99424591	5.23130636	3.77926812
hsa-miR-17-5p	9.3505978	9.2936742	9.0736402	9.0359697	9.2462539	9.0385663	9.2382939	9.2279244	9.3926383	9.0926085	8.6562475	8.9694968	9.2131119	8.79316743	8.85049638	8.75558231	8.968517	8.78576418	9.0925493	9.40713859	8.99417589	9.11395219	7.02034142
hsa-miR-181a-2-3	2.8642765	2.9319321	3.0081175	2.7544137	2.8090447	2.9536514	2.8961551	2.9006187	3.0401124	2.8913039	2.7854063	2.7959194	2.9123382	2.94161171	3.02083234	3.09188954	2.97720603	3.05510261	3.1607521	2.93494089	3.20194356	3.1091069	3.85060777
hsa-miR-181a-5p	7.1863416	7.5458978	7.8734749	7.6960278	7.4216766	7.4236245	7.0815921	7.5012159	7.4813453	7.4183787	6.6809678	7.0498231	7.5441072	7.72801604	7.83638399	7.23471291	7.62676835	7.48283352	7.47009046	6.72889461	7.56183723	7.55358584	6.99454248
hsa-miR-181b-5p	4.0021191	3.9493203	4.4023421	3.9158228	3.8290505	3.7318528	3.6369999	4.6086867	3.9240259	3.9073205	3.5425301	3.5443411	4.111256	5.05627093	4.93476535	4.43871024	4.68614473	4.88739523	4.31948559	3.85742099	4.68001019	4.53728695	4.29446149
hsa-miR-181c-5p	2.9037839	2.9911406	3.1334183	2.8578065	2.9791638	2.8718498	2.8260702	3.238218	2.8404159	3.085657	2.7860617	2.8252013	3.3377294	3.52568359	3.48675679	3.43830837	3.28117364	3.40645536	3.11613866	2.7951287	3.15384802	3.32329482	3.53301895
hsa-miR-1825	4.4994056	4.3581026	4.595293	4.1959178	4.5272748	4.3690422	4.2236903	4.2373338	4.5844078	4.5874926	3.8421659	4.1930426	4.2509431	4.33020481	4.64194544	4.10186652	4.02238532	4.48934195	4.44772152	4.2341232	4.35328606	4.35102763	3.83010363
hsa-miR-185-5p	5.8186692	5.9737009	5.7912286	5.6497277	6.0071461	5.6051417	5.7334226	6.1966107	6.4010433	5.9698263	5.1182357	5.1176256	5.9287571	6.43921615	6.61501161	5.95382193	5.84736557	6.37597185	6.30820412	5.89877245	6.30673648	6.19281638	5.57563331
hsa-miR-186-5p	5.3536697	5.212492	5.2093813	4.8861169	4.8867968	4.9534929	4.6957157	5.292244	5.3177283	4.9672556	4.3562465	4.5284423	5.1805508	5.64016517	5.69880217	5.15270257	5.30378423	5.62142953	5.5498516	5.18063226	5.46523032	5.50677044	4.99751447
hsa-miR-188-5p	4.2161632	3.621402	3.9275591	4.1936192	4.1475292	4.2361045	4.2038627	4.0591281	3.894651	3.7602536	4.9790698	4.419187	4.3475697	4.10244215	3.55199866	4.39807177	4.18722364	3.80456918	3.98869288	4.6724872	4.51770024	4.3708945	4.21841198
hsa-miR-18a-5p	4.7404139	4.4474412	4.3686422	3.9853746	4.5264054	4.1181863	4.5445739	4.726004	4.7236173	4.1923307	4.0488995	3.9461954	4.3940609	4.84836793	5.2038869	4.69679996	4.58266281	4.86067417	4.77720051	4.92682284	4.90782722	4.9262084	3.47663808
hsa-miR-18b-5p	2.8664628	2.6202293	2.6987086	3.0468973	3.025308	2.8491378	2.7668018	2.8956088	3.1419604	2.580001	2.9516865	2.7345134	2.8872454	3.05173077	3.19629593	2.94382023	3.02362564	2.99085086	3.07247322	3.06094381	3.06482216	3.22581113	2.6508723
hsa-miR-1908-3p	4.0561454	3.4764236	4.0661988	3.7374266	4.0094153	3.7004058	3.5968045	3.7456616	3.7243913	3.9207661	3.2697323	3.8520038	3.8133576	3.8872274	4.04717143	3.58894551	3.73414805	3.98498207	3.94427471	3.86425884	3.95254014	3.88966306	3.58140382
hsa-miR-191-3p	4.6848062	4.2612741	4.7429934	4.4504233	4.5837805	4.573283	4.4356159	4.3311572	4.7531868	4.6043067	3.9341963	4.2976041	4.3998187	4.35942997	4.72396236	4.14077004	4.18246369	4.5671771	4.51160683	4.28125426	4.46805377	4.61180431	3.91642914
hsa-miR-1914-3p	4.6571241	4.2520676	4.3863261	4.2259812	4.3984079	4.2715414	4.2405346	3.9981556	4.6497447	4.1264826	4.2379715	4.5309064	3.7528835	4.79195819	4.39447547	4.36997156	4.64085684	4.45400953	4.04921878	4.53257444	4.2740557	4.44178894	4.33773722
hsa-miR-1915-3p	5.9782198	5.6909879	5.8434919	6.0485861	6.064808	5.8171434	5.9981964	6.0618142	5.936269	7.0392471	6.4157018	6.1291924	6.2791875	6.04483129	5.8438212	6.33655071	6.23603241	5.64757077	5.55508562	6.0901372	6.01630047	6.13069084	6.78760635
hsa-miR-192-3p	6.096348	6.2588052	5.799204	5.5700058	6.127777	5.8241489	5.6980676	5.8782851	6.2894606	6.1818453	5.1635824	5.3864758	6.1066074	5.82410525	6.03066116	5.4297356	5.69376369	5.8117496	6.17847376	5.94034517	6.08164869	6.02951347	2.88905344
hsa-miR-192-5p	12.087864	12.20659	12.011477	11.848513	12.051031	12.036965	12.058272	12.046646	12.036837	12.247858	11.613637	11.962449	12.243638	11.7523494	11.7858159	11.6385039	11.9473306	11.7617521	12.1538527	12.2405597	12.0309542	11.9595789	7.40399912
hsa-miR-193a-3p	7.1873212	7.4967557	7.3590411	7.3526361	7.3566151	7.4391018	7.6240667	7.2892291	7.7364315	7.5807077	6.4208308	6.8956121	7.4299236	7.39595256	7.11145283	6.88666892	7.03876633	7.17496486	7.54915649	6.9395102	7.41876733	7.56202283	5.8033823
hsa-miR-193a-5p	4.8755093	4.8790964	4.4664179	4.8373337	5.1974639	4.6331174	4.8779812	4.9683654	5.2586266	5.1461255	4.3537517	4.1525599	4.8379452	4.73932053	4.67855256	4.7388961	4.5835086	4.78125744	5.17865195	5.26655059	5.15615278	5.15033874	3.66126303
hsa-miR-193b-3p	7.5886193	7.9082099	7.7579962	7.7232918	7.767848	7.4749828	7.5802538	7.6041222	7.680543	7.941222	7.718383	7.3681887	7.4924234	7.72133772	7.76380758	7.32437934	7.20848596	7.79935133	7.90970827	7.49195377	7.93811742	7.853657	8.68861118
hsa-miR-193b-5p	3.920488	3.9078317	3.6072618	3.7946886	4.1182722	3.6157874	3.8607353	3.9580857	3.8383899	4.3460664	3.7934917	3.5069161	3.9068252	3.8003397	3.69158731	3.74609164	3.7501685	3.88280102	4.23975045	4.23773811	4.18557997	4.28610158	4.32190144
hsa-miR-194-3p	3.3304523	3.5110587	3.0237412	3.2142125	3.3402664	3.3508933	3.3498901	3.4081968	3.3367284	3.4401109	2.9894402	3.2171503	3.3173771	3.09227509	3.37718999	2.98935176	3.21195963	3.41699491	3.39696955	3.3912537	3.35649232	3.23767163	2.41448435
hsa-miR-194-5p	11.015109	11.144047	10.949731	10.893011	11.023175	11.169944	11.153939	10.802179	11.092934	11.12108	10.493046	10.952335	10.96203	10.5698707	10.6882795	10.4331361	10.72205	10.7490794	10.9199664	11.0132034	10.9790139	10.8565283	6.32427261
hsa-miR-195-5p	9.2527164	9.3700216	9.3891333	9.2884446	9.2259274	9.2595841	9.2398934	9.4790266	9.2454486	9.5101477	8.8184531	9.5570999	9.7831183	9.35595004	9.41353507	9.67754556	9.34081852	9.52552132	9.58556635	8.88232884	9.35362671	9.58238938	9.33382546
hsa-miR-1972	3.0092507	2.7430411	3.2291108	2.9502754	2.9595338	3.1624375	2.9457654	3.1886342	2.543586	2.996157	3.8008283	4.0716038	2.954943	3.35261492	2.85835428	3.63789371	3.70864563	3.30561055	3.1630889	4.08576488	3.26704557	3.44084712	3.45601328
hsa-miR-1973	6.219861	6.0549509	7.1912928	5.9704805	6.0749928	6.63788	6.1911219	5.8896347	5.8364923	5.952893	8.3274135	7.8893934	5.7326632	6.57770379	6.74223328	6.2572965	6.53641909	6.49780574	6.50013471	7.63183638	6.62993636	6.92307396	8.92460659
hsa-miR-197-3p	4.5412572	4.3401829	4.4077028	4.1569151	4.4659899	4.3544613	4.0882946	4.4711604	4.5174182	4.4924238	4.0237342	4.1764025	4.2629673	4.45608159	4.84698827	4.1942966	4.17376919	4.62955477	4.46096602	4.34619065	4.53745119	4.54044215	4.6139004
hsa-miR-197-5p	7.5423249	7.572496	7.7772151	7.3793806	7.8914445	7.6766716	7.4980518	7.264336	7.5755165	7.6590826	8.0450771	8.3253331	7.4111967	7.41693645	7.27644447	6.97554726	7.18615677	7.42256017	7.38662894	7.70894627	7.39199442	7.524048	6.8701893
hsa-miR-199a-3p	10.036024	10.173731	10.405686	10.395062	10.090768	10.227	10.088457	10.392365	10.106905	10.172313	9.6975729	10.378457	10.519448	10.5212156	10.6685879	11.0036634	10.4838676	10.7275763	10.1395912	9.91185967	10.2426882	10.3031739	9.8710159
hsa-miR-199a-5p	8.382465	8.6469112	8.6853552	8.8239064	8.6120537	8.4744724	8.4860615	8.7894257	8.6534806	8.6680118	7.8340791	8.7477137	8.9867418	8.89164764	9.0546707	9.25632355	8.8408436	9.18255886	8.62709878	8.33422447	8.67874683	8.87780601	7.9397998
hsa-miR-199b-5p	3.7042587	3.4526237	4.1512908	3.1494871	3.6860825	4.2589719	2.9521532	4.1862786	3.2204755	3.2310402	5.515534	4.1265641	5.0719025	5.77435189	5.87243364	6.73555062	3.32021975	4.38803395	3.53573805	2.83002227	3.60064042	4.20419862	8.19381373
hsa-miR-19a-3p	9.555597	9.2460182	9.0039429	9.0335153	9.1000693	9.120628	9.2224815	9.1270767	9.4565136	9.1276159	8.9123074	8.9847241	9.4190665	8.91643365	9.01472934	8.81196079	8.82897469	8.65584675	9.09463169	9.36021721	8.67970147	9.15862993	7.4398776
hsa-miR-19b-1-5p	2.8663701	2.8389562	2.9607539	2.6455689	2.7815252	2.7362524	2.7146982	2.8355879	2.7313472	2.7374157	2.5690559	2.7382429	2.8689662	2.62622242	2.78902151	2.75956681	2.67819764	2.83647359	2.91314786	2.95060812	2.80616184	2.68257483	2.52263069
hsa-miR-19b-3p	11.193612	10.958586	10.856979	10.721537	10.785043	10.90637	10.92262	10.808868	11.188339	10.714306	10.642219	10.784344	11.081017	10.7639422	10.8280208	10.6413187	10.7941648	10.4438471	10.7684843	11.1337566	10.5055663	10.817878	9.31692964
hsa-miR-200a-3p	5.3362934	4.7412115	5.8699023	4.8573914	5.1294014	4.5127275	4.8444683	6.0045891	4.8931221	5.3423415	3.7658815	5.1245873	6.0958881	6.38783165	6.13778789	6.22116937	4.96021602	7.16726312	6.20220425	4.58716695	5.71383149	6.01357098	3.20060236

hsa-miR-200b-3p	6.6210259	6.3399109	7.5291948	6.4583852	6.6020675	6.1212172	6.2236879	7.3283746	6.5143701	6.8831042	4.8425624	6.6647955	7.403352	7.44671483	7.1669435	7.30732008	6.24140177	8.17277503	7.35743421	5.52071946	6.91290436	7.14255066	3.6668208
hsa-miR-203a-3p	4.1117487	4.4821897	4.3167077	3.7393066	3.8385104	4.1799322	4.0952816	4.3639148	4.1224827	4.2392689	3.6270968	3.7427497	4.0135999	4.52317759	4.25771739	4.0739484	4.2976408	4.31922154	4.82390733	4.58943628	4.48568058	4.62695192	3.03773703
hsa-miR-204-5p	5.4191112	5.4483134	4.7787175	4.4305325	5.2926014	5.0567812	4.8909599	5.5233369	5.2444397	5.1511598	4.8583681	5.2520233	5.5784885	5.37308113	5.74361022	5.40313825	4.97491112	5.23154986	5.32398003	5.14839627	5.24763083	5.24986395	4.72259454
hsa-miR-20a-3p	4.2826732	3.6999836	3.5797532	3.4920649	3.9315769	3.4096192	3.6797332	3.7355895	3.9607146	3.8376993	3.3974044	3.1642981	4.0299771	4.03359223	4.00699806	3.85567184	3.7005402	3.75370676	4.28769103	4.20401493	3.84008268	4.13179785	3.36332265
hsa-miR-20a-5p	10.772754	10.702001	10.525809	10.520137	10.561579	10.510757	10.681424	10.695008	10.793353	10.537809	10.148051	10.5314	10.571982	10.2499294	10.3632482	10.3703646	10.4885914	10.1206946	10.5625165	10.8223647	10.3407374	10.5637039	8.77971059
hsa-miR-20b-5p	8.6326083	8.4828515	8.0966339	8.1414067	8.4329529	8.08253	8.2152579	8.2488081	8.5829205	8.2835067	7.6358495	7.9598396	8.5255702	8.14722659	8.14595519	8.00213098	8.14405413	7.97378905	8.41217074	8.60550142	8.08699199	8.384539	6.48849382
hsa-miR-210-3p	5.9888443	6.1978938	6.0126486	6.2668851	5.4622174	5.9755806	5.9864398	6.5527487	5.497568	5.067217	5.7221955	5.1881602	5.8223705	5.63704397	5.79806696	5.42917204	5.44356863	5.964514	5.58148076	5.5225121	5.92722789	5.98019755	5.03789051
hsa-miR-211-3p	4.8133693	4.7722657	4.5214274	5.0372922	4.9927579	4.3345891	4.7616494	5.0547362	4.6098189	4.6003043	4.4958441	4.6914973	4.4718091	4.94826833	5.04724797	5.29056739	6.01342488	5.26623542	5.15224393	5.04816964	5.1311074	5.49603724	5.00169199
hsa-miR-2116-3p	4.3253779	3.874179	4.3756493	4.0575709	4.3152271	3.9696258	4.0800645	3.9648042	4.2229614	4.3124015	3.7523831	3.9826921	4.0198772	4.04318466	4.21119089	3.8340003	3.89551115	4.23771326	4.31472978	3.82835832	4.18651246	4.21253264	3.56701799
hsa-miR-212-3p	3.9752004	3.2142256	3.292933	3.7935435	3.7699377	3.1242316	3.2758775	3.9754968	3.1567435	3.2619431	3.5142976	3.4548197	3.3222638	4.09701644	3.81487172	3.65599809	4.30911856	4.0626222	4.02013964	3.47886473	3.95139133	4.04224174	3.7123876
hsa-miR-21-3p	2.9000234	2.9953352	2.8941056	2.9274898	3.3158224	2.8412597	2.8395678	3.1130749	3.158004	2.9685847	2.7203708	2.6631807	2.9277234	4.1587535	3.95464205	3.58372346	3.38015464	3.81675217	3.33672314	2.69800451	3.5408692	3.73396034	2.83745963
hsa-miR-214-3p	6.7384611	6.769656	6.8730605	6.8534029	6.6250494	6.7942815	6.5465197	6.9923699	7.1429642	6.8378892	6.0771944	6.7724291	7.187462	7.11073444	7.33234288	7.14135884	6.9518719	7.36712834	6.94095941	6.25535659	6.81841446	7.02323134	6.49117614
hsa-miR-214-5p	3.4433924	3.1661269	3.4761017	3.5695362	3.4216819	3.2438103	3.2340458	3.5519291	3.4747746	3.3508715	3.0624233	3.1957058	3.6043504	3.96277673	4.09627042	4.09573301	3.91830898	4.24270064	3.48890812	3.26444617	3.76961843	3.91288802	3.34209356
hsa-miR-215-5p	11.051649	11.048706	10.842305	10.679577	10.985094	10.93954	10.878494	10.883387	10.963517	11.029907	10.598105	10.832638	11.044281	10.6652493	10.6784832	10.5280674	10.8214276	10.6850124	10.9386101	10.9662921	10.9691966	10.8602172	6.21853526
hsa-miR-21-5p	12.349917	12.52968	12.42786	12.156953	12.119773	12.267941	12.343545	12.327943	12.093777	12.467662	11.414463	11.67307	12.472946	13.0981588	13.1159609	12.5842595	12.7659633	12.8677236	12.5007956	12.1128511	12.4311598	12.6416056	10.1875077
hsa-miR-218-5p	3.5568816	3.6409998	4.071367	3.5768522	3.6071328	3.7527539	3.306626	4.3304462	3.6547423	3.627302	4.2251125	3.6942437	4.4714539	5.16662782	5.13724649	5.74649498	4.35426553	4.47269437	4.38272729	3.38252065	3.87580305	4.43624651	5.89342222
hsa-miR-219a-5p	4.048685	3.4421135	3.2128907	3.0346479	3.5087153	3.486658	3.2123597	3.7141146	3.5285236	3.6226156	3.2698648	3.1054016	3.4435793	3.7410203	3.70523191	3.58878365	3.33580862	3.62183202	4.0575395	3.53418183	3.61807753	3.78274896	2.85861101
hsa-miR-221-3p	3.8874223	3.9551201	4.0589853	3.8608933	3.9844922	3.8654252	3.9860734	4.0059479	4.1836601	3.9723483	3.7041834	3.6592846	4.0429032	4.48052829	4.39478366	4.16746354	4.22730042	4.29421288	4.4635843	3.61913854	4.16574069	4.27503299	4.6549452
hsa-miR-222-3p	3.1187107	3.2201694	3.3733276	3.12086	3.109982	3.3970227	2.9143438	3.4816503	3.4650726	3.2334639	3.0620502	2.8912394	3.3435271	4.26348506	3.82799851	3.55895712	3.59858352	3.94030144	3.50964614	2.86688456	3.50382037	3.52545127	3.72728408
hsa-miR-223-3p	8.4837762	8.6647243	8.6156841	8.7688767	8.39622	8.3609016	8.4619619	8.6229395	8.339817	8.1632657	7.5998291	7.9654512	8.6642133	9.04258218	8.85571465	8.55573496	8.81890064	9.24213134	8.45063858	8.34599058	8.77826121	8.64338826	6.67511601
hsa-miR-22-3p	10.58246	10.603617	10.687702	10.643289	10.539492	10.580567	10.703471	10.585483	10.574236	10.815091	10.903711	10.495265	10.491903	10.4916605	10.7051572	10.403586	10.5370449	10.8458266	10.7087174	10.6160257	10.7444118	10.547151	11.7584824
hsa-miR-224-5p	2.74615	2.7286004	3.6805942	3.4593239	2.9066323	3.6909449	3.3253907	4.7183189	4.5579963	2.9542559	2.8739904	2.8981655	3.5912266	4.13005307	4.67620254	4.35143919	4.4629295	6.21869986	4.55108959	3.04074845	4.48704913	3.31701208	3.18948958
hsa-miR-22-5p	4.2408994	4.0017737	4.0700292	4.1796635	4.2907549	3.899536	3.9557785	4.4148169	3.944115	4.1777695	4.7048647	3.6397678	4.0176174	4.42355404	4.57091301	4.63162232	4.25658986	4.94389073	4.56392484	4.68923666	5.1461978	4.34286318	6.76042562
hsa-miR-23a-3p	9.5557974	9.683531	9.9186105	9.7966588	9.7342491	9.8705539	9.741826	9.8906107	9.6942488	9.6689592	9.5458623	9.8234081	10.119934	10.0638481	10.0970258	10.1850609	9.86971958	9.99691905	9.65726952	9.48037585	9.71501752	9.8114595	10.9818878
hsa-miR-23b-3p	10.823344	10.859568	10.65835	10.797695	10.939878	10.835883	11.163703	11.049658	10.860619	11.027807	10.502316	10.866078	10.985596	10.350663	10.4254613	10.4967171	10.4250538	10.7786381	10.7560175	10.9302735	10.9435119	10.7914932	10.2011928
hsa-miR-23b-5p	2.5411807	2.7431696	2.4892803	2.560934	2.7682951	2.5970434	2.6352648	2.7511524	2.7741075	2.720311	2.5755653	2.6876971	2.6314388	2.72080167	2.98960214	2.82792394	2.57095439	2.80874851	2.79170881	2.78936972	2.76425837	2.9258124	2.82616728
hsa-miR-23c	3.8193614	3.8406894	4.202093	3.6653563	3.8222897	3.9437267	3.9081615	3.7894347	4.1085156	4.0984587	3.510176	3.7771583	3.8707847	3.93892613	4.0738095	3.71799043	3.69012006	3.95891054	4.03132267	3.88069486	4.06729723	3.97181627	3.57371469
hsa-miR-24-1-5p	3.6731351	3.5963717	3.370963	3.550833	3.9088289	3.4478173	3.6401266	3.990241	3.7201823	3.8026256	3.5369055	3.4001478	3.945646	3.47481674	3.76876746	3.45551377	3.37997115	4.00603801	3.85565214	3.83297471	3.91037385	3.7757382	4.7271234
hsa-miR-24-3p	10.217725	10.254005	9.9887161	10.208478	10.316183	10.008528	10.361738	10.364506	10.053166	10.244291	10.119451	10.052708	10.310867	10.1106099	10.1344693	10.1200517	10.0262697	10.2767649	10.1694515	10.193873	10.3207876	10.143638	10.8682105
hsa-miR-25-3p	8.2911263	8.4829355	8.1614582	8.1484701	8.3509893	8.0336374	8.2422595	8.1439027	8.3058333	8.2766318	7.3910147	7.7813124	8.0644365	8.26201456	8.42833634	7.9251024	8.23299439	8.15140517	8.24786294	8.13560608	8.18546852	8.23830758	7.11526334
hsa-miR-26a-5p	11.085993	11.085719	11.173155	11.006237	11.063702	11.100527	10.862145	10.982252	10.987587	11.082058	10.6769	11.073569	11.259638	10.9465004	10.8977273	10.8758855	10.791046	10.8278172	10.8063237	10.7188284	10.8843885	10.9748279	10.9339374
hsa-miR-26b-5p	11.097449	11.091304	10.923376	11.024241	10.991271	11.008305	10.978614	10.928411	10.988722	11.074328	10.555109	11.029292	11.180439	10.9204243	10.934675	10.9246398	11.0312829	11.0268117	11.0803317	10.9246688	11.0302216	11.0257182	9.96415462
hsa-miR-27a-3p	8.5694593	8.7285743	8.900005	8.7550789	8.7509846	8.6841434	8.7835797	8.9446724	8.5627235	8.5971377	9.03021	8.624653	9.1078642	9.07273685	9.1262955	9.25828542	8.75499272	9.08474278	8.76164204	8.52469845	8.69567836	8.9313156	10.8662985
hsa-miR-27b-3p	10.715626	10.638138	10.25406	10.770482	10.819461	10.478927	10.985964	11.127404	10.522069	10.801514	10.71955	10.557257	10.771552	10.2148125	10.4048184	10.449074	10.3203008	10.7148298	10.6509959	10.8988686	10.9461275	10.7640507	10.3902052
hsa-miR-28-3p	2.6690659	2.5704952	2.5947313	2.5253785	2.6324465	2.6425823	2.6371196	2.5640143	2.7405424	2.7382073	2.5629287	2.6144097	2.6543532	2.56945475	2.73521673	2.65964506	2.54595086	2.60512807	2.65545856	2.64737177	2.60282207	2.72762083	2.48727688
hsa-miR-28-5p	6.7375789	6.9724549	6.6396372	6.64537	6.696402	6.371951	6.585028	6.6355087	6.899117	7.0584442	5.8679618	6.294233	6.898108	6.6846429	6.67287171	6.50149959	6.58893109	6.56513119	7.03399682	6.64224716	6.74741254	6.9565033	5.41527409
hsa-miR-2861	5.4970457	5.0096016	5.1065632	5.7872953	5.5455412	5.7379761	5.9888812	5.625377	5.7299636	7.1163952	6.5407171	5.8302006	6.0340666	5.28630486	5.00647016	6.11359759	6.17084374	5.10796525	5.2952628	6.21421187	5.88511621	5.55634253	6.4949729
hsa-miR-296-5p	3.7316159	3.6426921	3.5712525	3.8362295	3.7549105	3.8497854	3.813114	3.9065213	3.5671599	3.6746669	3.928377	4.0485654	4.0159102	3.55775394	3.43589315	3.71112433	3.66541769	3.67800151	3.77443194	3.7662377	3.78703684	3.57825287	3.67915001
hsa-miR-299-5p	3.4554842	3.7628832	3.4674395	3.3248216	3.2802942	3.4542742	3.2576241	3.2951244	3.4179976	3.2807603	3.5652088	3.4691103	3.3765991	3.53346473	3.91192764	3.60887948	3.36809353	3.63142045	3.85217732	3.4964326	3.63160994	3.62957834	6.02716101
hsa-miR-29a-3p	11.077542	11.127323	11.157238	11.087886	11.024644	11.206686	11.144719	10.973618	11.135661	11.123591	10.976034	11.147883	11.218378	11.3337625	11.1497205	10.9778685	11.2063631	11.2020493	11.1675785	10.8862605	10.8406314	11.1278718	11.6568581
hsa-miR-29b-3p	9.3027264	9.1174012	9.2269674	9.100395	8.9791959	9.3632301	9.0639695	9.0958372	9.1757038	8.9537236	9.2397354	8.9044023	9.3207236	9.67849777	9.48355606	9.12807139	9.13217715	9.24661688	9.14395838	9.01324843	8.85255897	9.11883967	9.99754895
hsa-miR-29c-3p	11.097137	11.070181	10.996777	11.035882	11.044898	11.081679	10.981741	10.911415	11.200437	10.98314	11.005142	10.976475	11.141292	11.1826731	11.158274	11.0733271	11.00172	10.8874686	11.1123031	10.9133985	10.8925945	11.0857102	12.1897052
hsa-miR-29c-5p	4.8719959	4.8149963	4.730555	4.6903179	4.9949782	4.7334595	4.6227711	4.9834727	5.2343694	4.7576426	4.6779683	4.5002435	5.0100307	5.12367607	5.16130305	5.06137513	4.86199415	5.04732917	5.19907539	4.82400497	4.93166344	5.0756643	6.58849747
hsa-miR-301a-3p	3.7796545	3.4170243	3.5424369	3.2091938	3.442197	3.2884936	3.3576437	3.6896223	3.3904782	3.3642262	3.4000682	3.2943608	3.7187714	4.26203223	4.46561176	3.94685413	3.68590302	4.23999638	3.84337993	3.40117395	3.7305114	3.86698444	4.1089244
hsa-miR-30a-3p	5.8387092	6.0617432	5.9144537	6.0931035	6.1641136	5.6700822	5.502484	6.1162513	6.1110334	6.315824	5.5555431	5.2356596	6.1229588	5.52158171	5.63145735	5.99181467	5.49474625	5.90616536	6.11895881	5.51134562	5.92047299	5.89560218	7.37687076
hsa-miR-30a-5p	8.9287878	9.0372572	8.9238983	9.0779997	9.2859973	8.8677882	8.8316881	8.9856234	8.9025986	9.2409424	8.7989992	8.5937618	9.1364651	8.46402736	8.4728866	8.88630265	8.59300993	8.89127274	8.92842538	8.6111858	8.88800902	8.69590522	10.0420777
hsa-miR-30b-5p	10.949374	10.854058	10.885645	10.879689	10.841928	10.853294	10.871095	10.648422	10.914679	10.869759	10.654126	10.631929	10.836277	10.5739935	10.6007097	10.5754598	10.582175	10.3659595	10.7559523	10.6291439	10.5505341	10.7988336	10.980193
hsa-miR-30c-1-3p	2.6267851	2.7331067	2.5671956	2.6399295	2.8328213	2.6984667	2.6135888	2.6881537	2.7758227	2.7715383	2.5027789	2.5392008	2.5173105	2.73305527	2.71823544	2.76279345	2.54180454	2.61745852	2.74368463	2.77284064	2.74708314	2.67518542	2.66967604
hsa-miR-30c-2-3p	3.1921806	3.0370594	3.0522833	3.2016379	3.369325	3.0034185	2.7978796	3.3879135	3.0237765	3.1421242	2.9546833	2.9698506	3.0303762	3.15191865	2.95845522	3.08935972	3.17735988	3.29619163	3.41790964	3.03713303	3.19933683	2.98673222	3.2801126
hsa-miR-30c-5p	9.2560228	9.3221589	9.0407803	9.3291063	9.5491115	9.2249943	9.2838376	9.1932607	9.3297663	9.4323159	8.807837	8.9848407	9.3126324	8.70385076	8.81845535	8.75645869	8.67886763	8.77812326	9.11440197	9.18247827	9.15311624	9.19599246	9.25210471
hsa-miR-30d-5p	8.7014913	8.527731	8.4234584	8.540643	8.5883981	8.4617	8.463441	8.3648038	8.5941243	8.5998368	8.0702146	8.2166807	8.59623	7.95546151	8.08437647	7.9422268	8.02631288	7.87840783	8.26984331	8.33160767	8.19374219	8.34411569	8.84328263
hsa-miR-30e-3p	6.6854793	7.0024283	6.6696725	6.8315449	7.0834168	6.543631	6.6312046	6.7142867	6.8508386	7.0441044	6.4517906	6.2669328	6.8741493	6.44557902	6.70580543	6.47525266	6.38889479	6.47028527	7.00100837	6.5920342	6.80122102	6.97951558	7.44485758
hsa-miR-30e-5p	9.3016398	9.2763636	9.1022801	9.1760442	9.2736228	9.2561939	9.1691821	8.9120519	9.4135138	9.2725461	8.8946853	9.0504414	9.2863357	8.84154489	8.94641055	8.73870226	8.76128478	8.77344515	9.16700416	9.0636141	8.96116619	9.190407	9.399259
hsa-miR-3125	3.4065982	3.3276127	3.7086809	3.5186834	3.633704	3.3633895	3.1853166	3.4683572	3.6911032	3.5715488	3.6192067	3.6333084	3.3412563	4.35214454	4.03137944	3.8847749	4.0464342	3.94253302	3.78425734	4.09419255	3.77436172	4.00693026	3.87669651
hsa-miR-3135b	4.9996122	4.4859431	4.7226775	4.9091034	4.6926573	4.4080431	4.6225529	5.0220886	4.2168416	4.1153501	5.3337991	5.2560801	4.7693448	5.2377841	5.00679334	6.95767717	7.12175179	5.22896918	4.90658383	6.02335359	5.17751476	5.58907277	6.33202391
hsa-miR-3141	3.9635061	3.5772518	3.5055293	4.0656196	3.6795861	4.0833294	4.0677457	3.897187	3.6422951	3.6625382	4.5162425	4.0669773	4.3276015	3.92165526	3.47953086	4.58248569	4.81126208	3.96859344	3.77349236	4.61113081	4.02660335	4.09260716	4.28556032
hsa-miR-3151-3p	3.7830651	3.4856632	3.8937036	3.4384887	3.6630928	3.7086969	3.5168235	3.6280071	3.4775998	3.7443145	3.7711674	3.8362027	3.6176321	3.87103306	3.81652763	3.41554096	3.65479475	3.84559136	3.77483039	3.89897753	3.87669857	3.97139749	3.79945485
hsa-miR-3156-5p	3.2073863	3.035832	3.3207671	3.3452498	3.1649545	3.0684795	2.9563408	3.241633	3.1650971	3.0226954	3.8058785	3.4371296	3.0666031	3.84457723	3.46064331	3.64625456	3.8624332	3.44072146	3.41319984	3.78734324	3.55124816	3.56598053	3.77034543
hsa-miR-3162-3p	4.726256	4.6091032	4.9347122	4.3932496	4.8344881	4.7406352	4.5260016	4.4344859	4.7780723	4.7987438	4.0877478	4.6425899	4.6451011	4.49967748	4.73854829	4.27013497	4.34236364	4.70907168	4.69368663	4.45862667	4.53434879	4.70104316	4.13362125
hsa-miR-3162-5p	8.4607851	7.9944247	7.7273726	8.1011311	8.2534281	7.6558064	7.8529356	8.1528257	7.7542259	7.9086235	7.9872131	8.0647453	7.7135127	7.79901147	7.74668028	7.6416292	7.96015567	8.07546902	7.91367949	7.93943288	7.8131382	7.90853764	7.45229624
hsa-miR-3180-5p	3.4875066	3.837381	3.6788075	3.5124749	3.5514599	3.4522168	3.3890601	3.609717	3.6584416	3.8591197	3.5590354	3.4474807	3.4546697	3.60578386	3.77693795	3.37613	3.3691195	3.50339219	3.47471196	3.38077334	3.53918894	3.53297556	3.32623795
hsa-miR-3190-5p	3.5936049	3.2645468	3.8502532	3.5709988	3.7675049	3.5624632	3.6244083	3.5032581	3.7153166	4.0655246	3.3150586	3.5763121	3.6214723	3.46253609	3.71631278	3.4345529	3.37419682	3.61813242	3.72661852	3.54784914	3.80870767	3.62446961	3.41623097
hsa-miR-3195	5.5663458	5.0581676	5.6155973	5.4851692	5.6153333	5.3246189	5.097327	5.5446236	5.7182659	5.0588434	6.2514624	6.6860744	5.2492861	5.50055755	5.59046783	5.4827297	5.96139245	5.71130874	5.8115324	5.98429737	5.664978	5.89845079	6.16657622
hsa-miR-3196	5.4584162	4.9655965	5.2336069	5.2374869	5.5134837	5.0914432	4.8974305	5.6475443	5.4515892	4.9803347	5.2990625	5.6647948	5.062871	5.52246337	5.26068039	5.78670289	5.95740959	5.60171765	5.59715504	5.45881063	5.39385777	5.60766415	5.70565407
hsa-miR-3198	5.0977701	5.0114182	5.2924035	4.762522	5.2174772	4.6289293	4.8563399	4.8049166	5.1795654	4.8430882	5.0431093	5.1941851	4.6206875	5.53317097	5.26331863	4.98327123	5.26174905	5.13986725	4.99462988	5.24197529	5.08914174	5.31759474	4.99735759
hsa-miR-320a	6.0897101	6.4055229	6.2701587	6.1546229	6.3891151	6.1495106	5.9217832	6.5654958	6.3838434	6.3113424	5.6915548	6.0363447	6.3993223	6.40220749	6.15491232	6.07093096	5.93149942	6.33175976	6.34149648	5.9179545	6.40602264	6.38920178	5.3002926
hsa-miR-320b	6.3931744	6.6599144	6.5898904	6.3043209	6.5654976	6.4079444	6.2665886	6.76536	6.7304803	6.553645	6.0613241	6.4016621	6.6929557	6.57456936	6.43565995	6.31632552	6.23723024	6.64053075	6.70373862	6.2404543	6.63848586	6.58636907	5.61798759
hsa-miR-320c	5.282174	5.5463914	5.521456	5.0780578	5.4084809	5.2559621	5.0871652	5.4794709	5.4683931	5.3741887	5.0768414	5.2296731	5.6420637	5.61478057	5.32506774	5.4183097	5.42163542	5.56479241	5.6177558	5.31290795	5.61047742	5.49161874	4.98280342
hsa-miR-320d	6.4622289	6.5993004	6.4767577	6.1744503	6.5524319	6.4359557	6.2144756	6.6322314	6.7913355	6.5419332	5.8909776	6.3285755	6.757208	6.64181512	6.42839904	6.52765188	6.31494279	6.57983714	6.72455603	6.2494943	6.59199797	6.60706904	5.69571543
hsa-miR-320e	5.914863	6.1016822	5.9192608	5.5924501	6.0502199	5.8300372	5.5909513	6.0605657	6.1975531	5.9914878	5.3914299	5.7406819	6.2205369	6.20130419	5.9448063	5.9460086	5.86633911	6.20580364	6.1609309	5.83971822	6.25086648	6.15664651	5.36672469
hsa-miR-324-3p	7.8029512	7.6288479	7.4124076	7.6347251	7.9984437	7.2425839	7.5972417	7.6581345	7.5107021	7.4170501	7.1000965	7.0706935	7.4062975	7.83306356	7.70671556	7.114588	7.70924268	7.80523903	7.58227086	7.46367852	7.89077695	7.78443913	6.5561904
hsa-miR-324-5p	4.9087697	5.0760061	5.0342111	4.8222983	5.1506792	5.1110129	5.1587115	5.4467831	5.2285043	4.8396585	4.4906304	4.6415585	5.1183068	5.39676998	5.49273508	5.0313733	5.19070941	5.49203339	5.25751408	5.14030514	5.64895829	5.42347736	4.86158217

hsa-miR-32-5p	4.3458902	4.0360277	3.8886363	3.5018301	4.0186258	3.843195	3.5366617	3.9295814	4.16087	3.7537088	3.6569091	3.470533	4.1548613	4.51885091	4.49818802	3.94558384	3.87154091	4.27150151	4.10194434	3.73569091	3.99623185	4.0505242	3.95134037
hsa-miR-328-5p	2.9676967	2.7688404	3.0548086	2.7592313	2.8718762	2.9038417	2.6720987	2.8383916	2.6073259	2.7428447	3.4296244	3.1636924	2.6976727	3.17460564	2.9521105	4.38883301	4.62022203	2.9690087	2.8312291	3.8289748	3.0520739	3.04573981	3.60737624
hsa-miR-331-3p	7.3088159	7.3297041	7.0767871	6.9671832	7.0648565	7.2271131	7.3785556	7.4936263	7.343062	7.2206929	7.0019648	7.0074487	7.3848446	7.20185463	7.28291517	6.8989518	7.03018545	7.58205018	7.35013856	6.9821494	7.4320396	7.32316985	7.87590983
hsa-miR-335-5p	5.5816485	5.2916109	5.2240549	5.1994947	5.4501678	4.7035694	4.5451444	4.5066813	5.2677321	5.5033499	4.1688314	4.237302	5.6695409	5.22822342	4.68920811	5.19558519	4.92549691	4.69551189	5.46327717	5.22183346	4.97756534	4.97010432	4.16914907
hsa-miR-337-5p	2.8897057	3.0923079	2.9332686	3.1541388	3.0395007	2.8853845	3.094634	3.1970442	2.9216799	2.9194837	3.1153137	2.9443552	3.3106787	3.23002545	3.49365246	3.59469086	3.01611598	3.45873485	3.6307008	3.01347741	3.31124437	3.19626107	4.6304137
hsa-miR-338-3p	4.5896774	5.1448238	6.0529064	4.128107	5.3400515	5.6574686	4.3378871	4.1911753	5.8376417	5.1483371	3.4691745	4.9151023	5.3913739	5.72366262	5.15224294	5.09339507	4.58872628	4.75797801	5.20841792	4.42016493	5.11010116	6.34279105	6.16346734
hsa-miR-338-5p	2.574238	2.4986991	2.5407486	2.5213403	2.4471824	2.4262057	2.4696134	2.4934884	2.5416787	2.5056359	2.5775047	2.692601	2.479419	2.49380344	2.67625635	2.58428324	2.49849098	2.49352346	2.51700073	2.49373776	2.59429926	2.6262983	2.62739223
hsa-miR-33b-3p	4.0453992	3.8513798	4.1718512	4.1465093	4.1326417	3.7739128	3.98203	3.6784951	4.0001462	4.1113966	3.6240814	3.9643074	3.8727332	3.96693715	3.99043019	3.74363695	3.82414457	4.08861629	4.11280393	3.96429058	4.0054003	4.12978227	3.74517138
hsa-miR-340-3p	4.319465	4.0823792	3.8343945	3.9470109	4.4623083	3.9861874	3.8842936	4.2667946	4.2459759	4.2224516	3.7305175	3.7852365	4.4473027	4.08430908	4.37033662	4.10925142	3.87807722	4.17683245	4.5589994	4.40215734	4.41743588	4.27938721	3.82424621
hsa-miR-340-5p	6.4131578	6.4351992	6.4007393	6.5245342	6.5177449	6.5212115	6.3428651	6.5735872	6.7750451	6.6224469	6.0603394	6.1369248	6.7608255	6.59970588	6.68795945	6.4095889	6.10456967	6.73340605	7.11890018	6.5400541	6.81426675	6.84750851	5.52441484
hsa-miR-342-3p	7.3498252	7.8107171	8.041639	7.8713861	7.6614753	7.70143	7.6085799	7.951967	7.8886799	7.5413374	7.0530343	7.0255349	7.7613595	8.37982333	8.17836567	7.63610103	7.81402953	8.19787921	7.8695854	7.26535903	7.90156435	7.55147995	6.09507758
hsa-miR-342-5p	3.2378765	3.3501712	3.691788	3.4031313	3.2570858	3.2955961	3.1340139	3.4005393	3.4879755	3.2253841	3.0189571	2.8597451	3.3732021	4.14811758	3.91027757	3.60584546	3.69833388	3.95531484	3.79229862	3.26398012	3.67058735	3.65009605	2.71939705
hsa-miR-345-5p	2.6943106	2.7720659	2.6459181	2.7902492	2.8072895	2.8181423	2.8218142	2.7989468	2.8419552	2.9009988	2.9806315	3.0010188	2.8913689	2.84406224	2.83435217	2.90941581	3.01082263	3.02333445	2.8685346	3.0035759	3.02998595	2.84767487	3.07839258
hsa-miR-34a-3p	2.8145705	2.771322	2.9721167	2.6892304	2.7494897	2.9062337	2.9455255	3.0327777	3.1619025	2.6996032	2.683462	2.7858207	2.8009584	3.60164677	3.57691683	3.07152197	3.08637657	3.6269213	3.73423419	2.75514328	3.25605348	2.91143069	2.77285899
hsa-miR-34a-5p	8.3006245	9.0363669	9.3895519	8.6323234	8.2819832	8.8444571	9.579315	9.3858314	9.6299241	8.4855319	7.659923	8.0918038	8.8563969	9.79676804	9.93644259	8.72090339	9.25320316	10.0888673	9.89313109	7.69610828	8.95599202	8.4229596	6.78979094
hsa-miR-34b-5p	4.2572141	4.7017103	5.1442871	4.2328503	4.2649503	4.289803	5.0162409	5.1473911	5.4093123	4.3093909	3.7209289	3.5760017	4.6260254	6.43712335	6.45138521	4.9208761	5.42070605	6.62088572	6.2038214	3.87765616	5.18212112	4.55048089	3.70964216
hsa-miR-3607-3p	4.4186035	4.3049374	4.207061	4.4145362	4.1216546	4.2030016	4.1386952	4.2316859	4.5755163	4.2659003	3.8489502	3.6879281	4.3229996	4.57498638	4.9067499	3.37240407	4.04759536	4.3948911	4.50958788	4.36481017	4.19332895	4.42226022	3.80236746
hsa-miR-3613-3p	3.6404853	3.6168842	3.6668711	3.3546385	3.6252531	3.5014275	3.5044008	3.3507576	3.664438	3.7011119	3.1861243	3.4653637	3.4580528	3.60308285	3.78081768	3.30983867	3.37187941	3.6376021	3.64296382	3.58656938	3.60269189	3.67933328	3.32246314
hsa-miR-361-3p	4.896672	4.6412873	4.6085185	4.2478419	4.5022038	4.5175401	4.2734936	4.9501335	4.9376464	4.5387648	3.9574121	4.2693972	4.655739	5.33674492	5.22986828	4.77078797	4.92553629	5.38816341	5.10533051	4.61043181	4.60628142	4.8424204	4.29222989
hsa-miR-3614-5p	3.5275475	3.186471	3.5241619	3.3826408	3.4634921	3.3984418	3.4214715	3.4256752	3.5159843	3.666637	3.1453989	3.4142814	3.5098998	3.37857909	3.63830954	3.63699475	3.70167963	3.48029085	3.58821374	3.46665598	3.67661008	3.73003786	3.3352511
hsa-miR-361-5p	6.7065421	7.019277	6.6705106	6.8217788	6.9562439	6.6956891	6.6843943	6.8640124	7.0953399	6.912244	6.2195116	6.6328215	7.0121823	6.45378122	6.79473255	6.54442481	6.37807079	6.69578998	6.95476143	6.55891691	6.51133873	6.8938413	6.44872941
hsa-miR-3620-5p	2.8397648	2.9251798	2.9578273	3.0922355	2.8326909	2.6869645	3.1448919	3.2932331	3.0101988	2.8329254	3.2929419	2.9367766	2.9960234	3.62315332	3.31529851	3.82397027	4.97581044	3.46087742	3.00729528	3.73837207	3.66305793	4.21116447	3.61961256
hsa-miR-362-3p	4.9111681	4.9173942	4.8023531	4.7324926	4.5237174	4.4550646	4.5330736	4.8352976	5.1294344	4.9029545	4.3972418	4.3514649	5.1619088	5.29660501	5.44636324	4.95704555	4.90001646	5.20538733	5.33082821	4.8199631	5.09467313	5.21668366	5.68405254
hsa-miR-362-5p	3.8171259	4.1549229	4.0919741	3.8640915	3.7624911	3.7618534	3.6969686	4.0710274	4.1758086	3.846996	3.6498542	3.5296091	4.0398639	4.29350294	4.35121074	3.94980739	4.0343606	4.30595358	3.93922774	3.84383294	4.21943336	4.02080324	4.94340452
hsa-miR-363-3p	4.1138411	4.6263555	4.0161311	3.9906278	4.2628289	4.7751943	3.2400142	3.9229162	4.6363216	4.2345345	3.7021424	3.455185	4.179645	4.76097384	4.95024168	4.32571508	4.67191453	4.38633508	3.67332142	3.96422411	4.5713406	4.26878263	4.66852545
hsa-miR-3646	3.6552509	3.496989	3.8237139	3.6065469	3.6433205	3.5795133	3.4669873	3.6351957	3.4680108	3.5597706	4.4221684	4.280527	3.4317364	3.90045418	3.84730607	3.57956181	3.78115837	3.76978697	3.63652591	4.17655847	4.08842631	4.04722502	4.04894364
hsa-miR-3648	3.2411993	2.9539787	3.5202235	2.9512928	3.2302702	3.1430379	3.0004892	3.180493	2.958609	2.91099	4.1923296	4.0184717	2.9427547	3.48894505	3.34430836	3.23177499	3.42114313	3.27261767	3.42268271	3.73594509	3.55886534	3.29326933	3.80750641
hsa-miR-3651	6.108847	6.4132485	6.2980689	6.2725193	6.2026131	6.0129403	6.071278	6.336896	6.249418	6.1458359	5.4700857	5.1923328	5.9223976	6.37780847	6.75512248	5.92952737	6.06348123	6.35919727	6.35381656	5.76857054	6.21561474	6.46305311	4.58813852
hsa-miR-3652	3.1163548	3.1498139	3.2758078	2.9376261	3.2134644	3.0108162	2.9498698	3.1321536	2.8523655	2.9480196	3.7115308	3.4412355	3.0196567	3.40961373	3.10654823	3.56208863	3.5353597	3.23666969	3.05143611	3.54269379	3.18311041	3.26168913	3.61274468
hsa-miR-3653-3p	7.8377339	8.0144864	7.8592817	8.1024589	7.8650249	7.8531898	7.9498529	7.8566229	7.9673871	8.0955967	7.3243803	7.3102762	7.8875186	7.5323763	7.7592748	7.14642301	7.40787174	7.47609908	7.65208317	7.5948304	7.47210564	7.54519319	5.73701548
hsa-miR-3656	4.550399	3.9682467	4.5027938	4.5179551	4.4048524	4.6492826	4.6927577	4.7396785	4.8306276	4.4019329	5.2059462	4.7874931	4.9814611	4.7202855	4.19132186	5.07921479	4.78853037	4.16400985	4.48787575	5.07382073	5.01232854	4.5848697	5.49146124
hsa-miR-365a-3p	9.2965788	9.2062665	8.7418201	9.2139913	9.3738776	8.6663847	9.2647724	9.0807647	8.8337939	9.488889	9.1288659	8.7061427	9.2220622	8.70930371	8.78643677	8.86900936	8.65208556	8.91534586	9.36514883	9.38178241	9.28597912	9.21589823	9.87191376
hsa-miR-3663-3p	5.2311723	3.8000746	4.5990673	3.8998787	4.979657	4.4559963	4.0252613	4.1192562	4.4919525	4.1055691	4.9564409	4.7993791	4.4111219	4.64734923	4.65723386	4.88166112	5.37851241	4.16022248	4.01721781	5.09335058	5.06545589	3.95286215	4.3990114
hsa-miR-3665	5.8689761	5.283056	5.5450073	5.8069251	5.6303199	5.6705075	5.5618483	6.2041376	5.9221004	5.2978876	6.0874666	5.8897798	5.7596009	6.25756693	6.27228654	6.0455349	6.15372966	5.78798226	5.80941162	5.93096516	6.06797648	5.58243127	6.13533809
hsa-miR-3679-3p	3.2261845	3.266902	3.3506827	3.1029576	3.4607949	3.4045783	3.2413883	3.3604892	3.6226515	3.3441719	3.1855505	3.2983257	3.2879336	3.34487579	3.15887038	3.20094856	3.19605908	3.33835475	3.11928181	3.37545952	3.46645756	3.33980779	3.27960319
hsa-miR-3679-5p	4.327479	4.0394433	3.9675485	3.8175326	4.0128504	3.9824953	3.7801542	4.1879477	3.6160911	3.8934422	4.4854523	4.5743001	3.9339084	4.4668415	3.97770487	5.16863887	5.26864638	4.32331532	4.16596634	4.64807972	4.33151825	4.48582749	4.79303455
hsa-miR-374a-5p	8.0463287	8.2392701	7.9693002	8.0608218	7.9275716	8.0148796	8.1122335	8.0504384	8.1423519	8.1456642	7.3504099	7.7179509	8.2568502	8.10990189	8.21253472	7.96485429	8.04266609	8.11167587	8.16006725	8.03632228	8.05977308	8.13874377	7.58867951
hsa-miR-374b-5p	6.834616	7.0567278	6.878754	6.8461803	6.8014728	6.7479119	6.5615497	6.8226542	6.9444891	6.8669283	6.2359476	6.3773786	6.8064564	7.11863205	7.16835516	6.80032562	6.93435327	7.05738597	7.20591027	6.45210044	6.99014943	7.13723504	6.63497067
hsa-miR-374c-5p	3.5577911	3.4631437	3.3967821	3.2843101	3.4225595	3.359507	3.3712916	3.2734483	3.4117111	3.5160814	2.9273934	3.1766387	3.4527314	3.75448297	3.84567999	3.45401346	3.52954619	3.77277914	3.73215567	3.60312619	3.77385493	3.68862306	3.5761821
hsa-miR-375	5.2672001	5.2991457	4.6439238	3.8967557	5.6455185	4.3038763	4.4436446	4.0509606	5.086764	4.6818442	3.8279336	4.3790848	5.7084445	4.45797993	3.77417776	5.21574178	4.33312492	4.83238014	4.4803845	5.72568606	4.49039173	5.24383474	2.59918081
hsa-miR-376a-3p	5.6773673	5.9203432	5.6742445	5.9550782	5.8811975	5.7920587	5.5727559	5.7935335	5.8040898	5.6174336	5.6412778	5.8337093	6.1627598	5.99473141	6.51402336	6.4484689	5.49323307	6.23687328	6.26604737	5.61168598	6.14367055	6.04582484	7.05090773
hsa-miR-376c-3p	5.8649177	6.2761481	5.9619086	6.4417855	6.2015338	6.1214874	5.9324057	6.1326928	6.0269677	5.913081	5.9814611	5.9711651	6.3540309	6.04905334	6.71065374	6.54140067	5.64931795	6.21437396	6.60855465	5.95376561	6.28251508	6.38976805	7.84648889
hsa-miR-377-3p	4.341223	4.401524	4.0004401	4.1826842	4.3722872	4.0455549	4.1218162	4.100461	3.9589385	3.8688158	4.3705374	4.2014337	4.4388029	4.65215808	4.94816986	4.5885356	3.94355882	4.7021124	4.60069473	3.98600689	4.36581225	4.54279189	6.30368258
hsa-miR-378a-3p	8.7862175	9.1004434	8.6721763	8.7040233	9.0965829	8.8537658	8.3270081	8.3767826	8.446093	9.3655309	9.4965484	8.511086	8.4566766	8.21606208	7.9716301	8.39728693	7.63409968	7.65736922	8.74120728	7.89618459	8.51222923	8.63534707	11.3255754
hsa-miR-378a-5p	5.2253272	5.5312314	5.0424621	4.8018237	5.4366858	5.0356097	4.4754468	4.7802528	5.1748245	5.8756941	5.554403	4.7026263	4.7877936	4.92174369	4.91261664	5.05497641	4.23961407	4.39542738	5.63383088	4.36684368	5.18289984	5.40562656	8.19721284
hsa-miR-378d	5.3043316	5.4096057	4.7290017	4.8329216	5.2538375	4.9035514	4.4575223	4.8969127	4.6927103	5.635918	5.630201	4.6444925	5.2241587	5.04261861	4.80302673	4.91709311	4.51403775	4.50468273	5.59451846	4.5047419	5.09655503	5.18003351	8.15682369
hsa-miR-378e	3.0968988	3.2299912	3.0126534	2.8119557	3.0324651	2.7740983	2.8000491	3.0447853	2.9153045	3.3655368	3.3133214	2.7400496	3.0457281	3.19953017	2.9995853	3.26099068	2.75585026	2.7702734	3.38728949	2.9649677	3.20486271	3.29101931	5.03706354
hsa-miR-378f	3.5150244	3.4253258	3.1425611	2.9765277	3.5498821	3.2671488	2.9049752	3.0996344	3.1070775	3.6418165	3.7214317	3.2948891	3.3724043	3.46145815	3.18793957	3.5175005	3.29241303	3.0374837	3.51366065	3.14657612	3.50512944	3.41370254	5.88745676
hsa-miR-378g	4.1205099	4.0714772	3.667341	3.5462035	4.1952619	3.6158791	3.4062379	3.6908335	3.3736043	4.5081115	4.6147442	3.4810416	3.8212976	4.0479655	3.73238448	4.1308227	3.49765241	3.63599465	4.27571413	3.37901382	4.17249043	4.10389034	7.28275965
hsa-miR-378i	9.0584092	9.339206	8.7481742	8.8398909	9.3122417	9.0374593	8.4838127	8.6072675	8.6121169	9.5574644	9.7159044	8.6886549	8.8311359	8.51282734	8.25560517	8.67682668	7.95187126	7.91741096	9.03057324	8.17398755	8.74957756	8.89527453	11.6815827
hsa-miR-379-5p	3.0063565	3.1085133	2.7471443	3.1073162	3.1843097	2.8856499	3.1302682	3.2102031	2.9400511	2.9397282	3.0940232	3.1580576	3.1712188	3.23163344	3.44554417	3.43859527	3.06679641	3.12343019	3.31662539	2.97611189	3.43817853	3.32691744	4.44009162
hsa-miR-381-3p	3.6989056	3.9122148	3.4445306	4.0251073	3.8290375	3.5815665	3.5763754	3.6198846	3.6087879	3.5937786	4.526056	3.5193469	3.9007905	3.84154536	4.29079441	4.36585115	3.63129387	3.86589527	4.14749215	3.67253743	3.99053351	3.80061047	7.38205923
hsa-miR-3907	3.9381048	3.4396846	3.4641191	3.6908158	3.9180429	3.3944798	3.590021	3.7759109	3.4432549	3.3860153	3.6266021	3.7073397	3.3105326	3.84552261	3.58479501	3.96609638	4.94096478	4.01740919	3.86036025	3.77902369	3.93427343	4.09866939	4.17746894
hsa-miR-3911	3.1155681	3.0683065	3.0369403	3.0132215	3.1376471	3.0121088	3.0458791	3.2164794	2.70441	2.7842327	3.4036484	5.8217638	2.9751675	3.34479104	5.86926174	4.03012582	4.78820575	3.36391654	3.02950071	3.76787128	3.25906723	3.38583231	3.76090482
hsa-miR-3940-5p	4.1563524	3.3959139	3.6003282	3.9545786	3.9945542	3.8109765	4.011902	4.1433831	3.9211533	3.6776959	4.464366	4.0099277	4.0212327	4.02301023	3.83456702	4.08176432	4.03142517	3.75952992	3.70931034	4.18234909	4.12716653	3.6539702	4.13085005
hsa-miR-3960	9.6680699	8.8872185	9.2928099	9.2482846	9.337107	9.6910437	9.3514908	9.436393	9.5053561	9.084007	9.6263838	9.5060047	9.4479756	9.54452501	9.24274635	10.4238005	10.4943725	8.83411853	8.72977395	9.75931479	9.39134721	8.55666753	9.87197262
hsa-miR-409-3p	3.34957	3.3092161	3.3774761	3.3160606	3.4739127	3.3526391	3.2846594	3.2982951	3.4337305	3.1917511	3.2517257	3.3593895	3.0296683	3.59234159	4.01855927	3.54252048	3.34602662	3.8612139	3.68711649	3.29316383	3.57452209	3.51420372	3.79135595
hsa-miR-423-3p	2.9288715	2.9555372	2.9046008	3.0394688	2.910394	2.8785669	2.7206985	2.8321713	2.969876	2.907422	3.1326646	2.8836666	2.8080888	3.10327203	3.16589939	2.87874147	2.79929637	2.94653557	2.9354534	2.93321053	2.99042188	3.0558311	3.0617668
hsa-miR-423-5p	5.6523949	5.631381	5.307746	5.5554266	5.8456347	5.1469835	5.2657061	5.6623452	5.4462843	5.7098254	4.9440988	4.9631262	5.664727	5.84117817	5.84342178	5.45422326	5.39043192	5.76476984	5.81553693	5.41210996	5.80161285	5.85907102	4.87230733
hsa-miR-424-3p	2.9167494	2.6820223	2.5806813	2.6509654	2.7770412	2.7597842	2.605137	2.7384106	2.5641751	2.647039	3.0935681	3.0159365	2.7061938	2.89995242	2.8876014	2.87865486	2.85868589	2.91305934	2.71637158	3.02556816	2.91128198	2.77235951	2.90305701
hsa-miR-424-5p	8.6723361	8.72214	8.3444752	8.1941086	8.8617153	8.5274583	8.3814477	8.3410834	8.2529863	8.8078575	8.1300327	8.6116233	8.6146878	8.54665293	8.96906041	8.65127042	8.21443005	8.74693582	8.25740038	8.31207331	8.67576867	8.69014005	5.87452511
hsa-miR-425-3p	4.3575335	4.0658228	4.6501606	4.2434597	4.3726017	4.3016228	4.0685792	4.0458557	4.4260176	4.5649272	3.7479555	4.1292564	4.3012025	4.2193029	4.37193505	4.03903079	3.99327678	4.30710014	4.40279818	4.17553699	4.40568815	4.31249635	3.81006315
hsa-miR-425-5p	6.1112201	6.355129	6.1545718	6.2492223	6.362837	6.1447135	6.1056481	6.2884144	6.632053	6.3733014	5.4668954	5.5399509	6.2003933	6.65307652	6.76043433	6.14403239	6.1618441	6.65001273	6.4233358	6.131489	6.51641432	6.4155317	4.79660235
hsa-miR-4257	3.8977909	3.10979	3.5297909	3.2297021	3.8123811	3.1394322	3.4534275	3.4608402	3.0800487	3.2102522	4.0716039	3.9261719	3.3469966	3.63586815	3.4304459	3.8033349	3.92129675	3.74531027	3.29343149	3.98690263	3.73508635	3.29588063	3.76905294
hsa-miR-4261	4.8879551	4.6310347	4.2853951	4.6944106	5.0177589	3.9537131	4.1083705	5.0148591	4.156708	4.4025688	4.3295314	4.4354672	4.4258997	4.95732015	4.52200969	4.51265465	4.58132443	5.03041399	5.05290203	4.56751708	4.64479666	5.02184403	4.68805449
hsa-miR-4270	7.2366821	6.979208	6.8150081	7.8551567	7.4141053	7.9183819	7.953348	7.2902476	7.6877912	6.8473173	7.8448462	7.1515487	7.9775958	6.41589789	5.59184711	6.73400194	6.17655014	5.99714473	6.5075397	7.09096112	7.20739625	6.79810031	5.24554793
hsa-miR-4271	4.7933241	3.3898058	3.2495867	4.1619765	4.230694	3.9769211	4.2217858	4.2963437	3.7076229	3.7521471	4.8303994	3.9778458	4.2430304	3.92813706	3.76032737	4.67243751	4.41932337	4.27148812	3.90387187	4.59274597	4.38344908	3.57021803	3.6815545
hsa-miR-4281	8.1766319	7.5966854	7.9589185	7.9356492	8.0712536	8.867063	8.3602672	7.8542463	8.1993289	7.571568	8.2580951	7.9788472	8.1016327	7.52285613	7.34987216	7.81765875	7.56491249	7.32489439	7.27694048	8.0286878	8.18288635	7.15003202	7.57875808
hsa-miR-4284	11.597128	11.37938	11.402474	11.606424	11.519663	11.419928	11.92008	11.510426	11.443133	11.744653	11.12476	11.428737	11.391137	11.4736101	11.5096727	10.8830404	11.5104153	11.2955223	11.5947489	11.2806706	11.2006483	11.3171817	11.673135
hsa-miR-4286	10.47009	9.6302705	9.7721978	10.715083	10.494559	9.9655055	9.6726824	10.667808	10.203407	9.87676	9.7883182	10.82078	9.5896015	9.9042691	9.76247203	10.06415	10.8674825	10.2710864	10.5371545	9.98523618	9.91268658	10.5285568	9.84605434
hsa-miR-429	4.1372276	3.8262111	4.6372392	3.979248	4.1106832	3.4025281	3.8742407	4.9063737	3.7265184	4.4301625	3.0504002	4.0591102	5.0255735	5.08528196	4.86701402	5.0331307	3.89040905	5.93673174	5.16118645	3.54395297	4.7783793	4.93819437	2.86781082
hsa-miR-4291	5.5054511	5.5463611	5.3256464	5.5316455	5.7016491	5.0522613	5.2691041	5.7305606	5.4536667	5.5423882	5.1381878	4.9982112	5.8533651	5.69065466	5.64435076	5.40027504	5.35420199	5.78014305	5.70622499	5.41975719	5.7429277	5.85618682	5.95599172

hsa-miR-4298	4.1550841	3.3530387	3.1263276	3.3860799	3.2864506	3.5743255	3.4201525	3.1584715	2.9692583	3.1941376	3.9757328	3.7723643	3.2867041	3.54636751	3.32475717	4.67878844	4.72302829	3.81190222	3.19523131	4.40910218	3.6138323	3.6938872	4.63444182
hsa-miR-4299	9.153586	9.1467746	9.2264916	9.1157614	9.4578975	9.2278346	9.1720835	8.8540493	9.0763237	9.1750722	9.6727867	9.7191714	9.1312544	8.91953943	8.91676057	8.70339206	8.86432198	9.05286611	8.88820669	9.52519234	9.01981028	9.09250698	8.9787102
hsa-miR-4306	5.8750194	5.9116827	5.8951449	5.5615063	6.0256295	5.6719921	5.6922957	6.1194136	6.5174377	6.1394019	5.1067459	5.3263478	6.0675946	6.55170337	6.67263431	6.13589926	6.00016505	6.36383408	6.40222283	6.08622573	6.27533903	6.24000906	5.79307759
hsa-miR-4310	4.3306079	4.1234927	4.4957483	4.0913504	4.3891903	4.3476784	4.1025004	4.0731213	4.2372213	4.3903207	3.6872576	4.2309862	4.2126358	3.98391081	4.33659441	3.89474607	3.83920052	4.36734269	4.31769624	4.12613656	4.1585766	4.25561722	3.74935107
hsa-miR-4312	3.4478234	3.1577016	3.7309272	3.4348646	3.4598047	3.4184819	3.3261228	3.5067248	3.6143459	3.6851286	3.1218063	3.3468182	3.3751587	3.46116999	3.82331219	3.32274734	3.37127607	3.40885402	3.53821682	3.40024401	3.591997	3.60647526	3.23175986
hsa-miR-4313	5.2411773	4.7825386	5.1464712	4.9022623	5.1327278	4.8853393	4.8749927	4.9458922	4.9924309	5.03933	4.4664477	4.733862	4.8163164	4.79867915	5.0940318	4.74758626	4.63744494	4.92740792	5.00890499	4.76102641	4.96145348	5.0052098	4.57773756
hsa-miR-4317	2.7775435	2.8076523	2.8350335	2.7160538	2.8018109	2.5962105	2.7161541	2.7219487	2.6941307	2.7398185	2.7998893	2.6509178	2.9249936	3.17239295	3.1488043	3.19773482	3.01882243	3.04232818	2.86792804	2.88097423	3.02918777	2.84844067	3.68756585
hsa-miR-4323	3.4483964	3.266214	3.3427384	3.1644808	3.3792668	3.3616483	3.1681018	3.1364775	3.3626698	3.3376997	3.0214003	3.2229477	3.1632768	3.31418403	3.28276074	3.18556192	3.13657478	3.33490862	3.2331791	3.16673549	3.33001259	3.38548837	2.95942079
hsa-miR-4324	3.3165998	3.2330821	2.8767489	3.276615	3.2311818	2.9067423	2.9689711	3.1434732	3.3582896	3.3257781	3.143819	2.9452886	3.3733828	3.33480541	3.40927313	3.28605914	3.15061839	3.19912079	3.36889922	3.13226697	3.22713636	3.54952354	3.72429885
hsa-miR-4428	5.4129422	5.2896267	5.5046681	5.2977063	5.6225925	5.222695	5.3196471	5.2440901	4.9397361	5.2050083	6.3763048	5.9597816	5.192473	5.72485484	5.40631626	5.26213316	5.31555098	5.55877439	5.36879867	5.88459061	5.56509925	5.45475816	5.38149014
hsa-miR-4430	4.4365125	4.6152715	4.7563487	4.2815308	4.7959406	4.113798	4.4076915	4.7003983	4.0316468	4.5905598	5.381211	5.4678947	4.414813	4.66989095	4.46561009	4.3055201	4.300562	4.61216008	4.59189203	4.95511613	4.71904597	4.67789884	4.69573756
hsa-miR-4433a-3p	3.4691776	3.0360149	3.0109294	3.9153901	3.5054363	3.7488455	3.8298458	4.0870076	3.6658347	3.2255687	4.4311662	3.4662633	4.5102913	3.36494663	3.0244789	3.86318523	3.63287569	3.39060233	3.55946906	4.20605889	3.94133057	3.40915452	3.68293726
hsa-miR-4433a-5p	3.9284622	4.0004523	4.0501643	3.7744228	3.9903871	3.8273617	3.6818138	4.1204278	3.9382702	3.9821995	3.7066822	3.839797	3.989497	3.88811165	4.01534309	3.66075241	3.59262227	3.89918533	3.90933857	3.94859328	4.01650528	4.00854211	3.42274109
hsa-miR-4436b-5p	4.0911747	3.8316818	4.2188202	3.7528457	4.2505623	4.0611064	3.9104274	3.8588021	4.2605858	4.1292027	3.6370891	3.8425371	3.8285676	3.94647062	4.15452702	3.85222284	3.98816676	4.10316493	4.04614058	4.01300837	4.08645764	4.12609106	3.64535069
hsa-miR-4442	5.0247266	4.6312066	4.8545229	4.6983227	5.065653	4.7358223	4.7423645	4.563717	4.8965411	4.6078603	4.9132939	5.1346916	4.6955843	5.14041098	4.796749	5.05422872	5.31587325	4.94860912	4.78919614	5.17049044	5.03859988	5.02198364	4.80826322
hsa-miR-4443	4.8048218	4.2817289	5.0059379	4.7693334	4.8787596	4.75389	4.6931341	5.0233581	5.03147	4.5123989	5.7294499	6.3478283	4.5682445	5.02580884	5.06659952	5.11647205	5.51850387	5.27349754	5.36499644	5.4634064	5.18717762	5.4457883	5.74391491
hsa-miR-4449	3.0426999	2.8499222	2.9333936	3.0099978	3.1874961	2.7579143	2.9439185	2.9922147	3.0351304	2.9888675	3.0144724	2.9036275	2.9206236	3.35089382	3.37284952	3.07368344	3.15808789	3.47639046	3.26881346	2.97766223	3.04438173	3.02839684	3.06280186
hsa-miR-4455	3.544745	3.5500926	3.0041017	3.3536704	3.5378024	3.7245941	3.0104852	3.9115671	3.367226	3.4110888	3.3526865	3.1280958	3.8003001	4.01004726	4.17343432	4.29142384	4.18081049	3.6483188	3.58176317	5.34327551	3.775331	4.13817533	4.1549068
hsa-miR-4459	9.4615001	9.2685283	9.9210091	10.022895	9.3245617	9.9411092	10.389354	10.054535	9.4663793	9.6216496	10.749999	10.584253	9.7008744	8.97709088	8.8744604	9.30199276	9.70171532	9.0349111	9.25667733	10.199119	9.98684394	9.27071709	9.1849522
hsa-miR-4462	3.1154482	3.0657272	3.3454011	3.1411955	3.3245482	3.1102168	2.9937308	3.1398043	2.9272138	3.0289917	4.0010758	3.8445061	3.1532002	3.52926914	3.28695627	3.50045889	3.645051	3.47777244	3.23676973	3.65353134	3.42372517	3.53631514	3.70502487
hsa-miR-4465	5.7335653	5.8105766	6.5728959	5.5838244	5.8863407	5.9734457	5.7396589	5.7006117	5.7012511	5.838515	6.8866383	7.2493856	5.643455	6.04819182	5.92041693	5.36853512	5.7035146	5.84338014	5.65330599	6.08845785	5.86307502	5.96491668	5.51028361
hsa-miR-4466	5.5812372	4.81127	5.4223291	4.9893222	5.3033891	5.359861	5.1792723	5.6045263	5.3090292	4.9366047	5.8426605	5.6335282	5.2982452	5.68685979	5.45105942	5.64201735	5.55277991	5.1744448	5.19400206	5.72678127	5.6053521	4.83872631	5.99594358
hsa-miR-4478	4.4817154	4.614965	4.7548216	4.3346466	4.7474464	4.5142124	4.4760742	4.474274	4.0924242	4.5205639	5.7434744	5.707865	4.4551664	4.88880835	4.39046818	4.44081164	4.60573314	4.77527761	4.59204833	5.2241072	4.77684526	4.90222378	4.7231676
hsa-miR-4484	4.7584071	4.3071056	4.5827261	4.4790061	4.6908812	4.4255487	4.3318932	4.5933265	4.3967823	4.7422527	4.3505532	4.6530285	4.6046618	4.47099059	4.6511195	4.62705509	4.76258782	4.55180784	4.61192326	4.88337165	4.76537997	4.7370492	4.58661846
hsa-miR-4485-3p	6.2764298	6.0204465	7.1329394	5.9773527	6.0197439	6.7645103	6.1100721	5.9651189	5.8669931	6.0143846	7.9764604	7.7564089	5.7092189	6.43829777	6.73161908	6.13972849	6.50384086	6.33977157	6.36627883	7.46262798	6.58059687	6.68997461	8.55495506
hsa-miR-4485-5p	9.0515555	9.1010966	9.7108789	9.1092085	8.797909	9.7920782	9.1816301	9.5201229	9.1556766	9.1958197	9.9546604	9.6286699	8.431116	8.69573344	9.05714882	8.74244662	9.01324956	8.99572273	8.87747854	9.54618662	9.22569311	8.90221227	10.1053154
hsa-miR-4497	4.6533944	4.4891708	4.7438445	4.660983	4.6347699	4.4431803	4.3856269	4.5448442	4.5175558	4.5806618	4.718185	4.7860572	4.4925581	4.80469019	4.57904144	4.52890318	5.03540564	4.70567415	4.53362897	4.619969	4.54310253	4.82367524	4.81983961
hsa-miR-4499	3.7999369	3.3967103	3.7160108	3.7179216	3.9189036	3.7268076	3.7667449	3.7413645	3.2748154	3.6227834	4.7627972	4.5714939	3.937569	3.90622798	3.28996211	4.47629264	4.42250983	3.76827066	3.53050716	4.60118809	4.13332791	4.03234021	4.36220855
hsa-miR-4505	5.2591558	5.2912489	5.5089476	5.086263	5.6232159	5.2968381	5.2865536	5.2109023	5.1514554	5.2690583	6.0170353	5.9906596	5.0837806	5.48103047	5.2843142	5.52258566	5.42488581	5.36095072	5.25681241	5.60766898	5.28040086	5.20531732	5.85736288
hsa-miR-4507	5.4313691	5.3227832	5.7216336	5.3124335	5.6280903	5.4539534	5.2620479	5.2686329	5.5478357	5.4339784	5.7978362	6.0162837	5.11267	5.55064851	5.37389677	5.63129924	5.82715566	5.36830407	5.52409281	5.65012326	5.45247271	5.42606597	5.61910145
hsa-miR-450a-5p	4.7346836	4.7345164	3.9721996	3.7798839	4.9761512	4.0887932	3.8522808	4.0806663	4.3253384	4.5389629	4.0372276	4.2188652	4.6716263	4.78609954	5.1812693	4.60661326	4.28984577	4.92100098	4.66172765	4.49527199	4.93268266	4.83187178	3.2855978
hsa-miR-4515	5.0044667	4.4511015	5.179969	4.6319639	5.1438276	4.793352	4.6394496	4.7378175	4.8913194	4.7662335	5.6406302	5.7245227	4.6392799	4.99101921	4.9583151	4.83124169	5.0642518	5.06475195	4.94638129	5.41456879	5.11537954	5.29584466	5.13595713
hsa-miR-4516	9.7219538	9.2317299	9.9624575	9.4151424	9.0569326	10.096115	9.6039824	9.7436342	9.851223	9.3265264	9.7833756	9.3966894	9.4365168	9.64155088	9.30974862	9.62798572	9.70573534	8.94625845	9.1792861	9.73529074	9.67392414	8.50454296	10.0662911
hsa-miR-451a	12.533367	12.717829	11.999031	11.965002	12.333922	12.743128	11.795396	11.854341	13.400331	12.663823	12.036089	11.467765	12.118999	11.7621063	12.4218772	12.5656865	11.9458066	11.4557056	12.0005515	12.684656	12.0823949	11.9023796	14.2290932
hsa-miR-452-5p	2.5369992	2.7608971	2.7771894	2.7918332	2.6311196	2.709552	2.8172018	3.2638383	2.7413762	2.614735	2.9449168	2.7690151	2.867473	3.01286299	2.98939089	3.09163071	3.0300358	4.02254108	3.04232621	2.83887049	2.86503763	2.79992922	3.39820145
hsa-miR-4530	6.3179766	6.4808819	6.8136873	6.4348644	6.6794553	6.5214103	6.2046719	6.5017202	6.3866577	6.4461708	7.3914697	7.5470771	6.3626913	6.86882774	6.75182665	6.83144074	7.17812343	6.54294846	6.45184949	6.53670349	6.61007472	6.56343341	7.23406189
hsa-miR-4532	3.7810242	3.4051571	3.8147586	3.6913727	3.9390777	3.6239401	3.4861811	4.074035	3.5624194	3.4417878	4.3411149	4.260395	3.4072919	4.00153896	3.81781773	3.90357453	4.00199981	3.88997661	3.78865334	4.11999654	4.00936382	3.83137516	4.42183197
hsa-miR-454-3p	3.6438425	3.4641965	3.4988042	3.3725455	3.5137586	3.1823506	3.4437441	3.5240012	3.6824972	3.4520861	3.2521937	3.28996	3.5416138	3.97131054	4.18040389	3.74388682	3.59810321	3.95706468	3.54703833	3.55484423	3.66863237	3.75278154	3.55310475
hsa-miR-455-3p	7.9529583	8.3930507	7.9205813	7.9844054	8.6056686	8.2291591	8.4451032	8.5412522	8.5576555	8.3526634	7.6472063	7.8349482	8.3747535	7.26229009	7.44211555	7.70507179	7.44397447	7.70362334	8.17598245	8.30815077	8.13862347	8.1984503	5.21699091
hsa-miR-455-5p	5.9632554	6.0324916	5.7364261	5.9017718	6.2456991	5.7620536	5.9167624	6.2242259	6.4714866	6.0852106	5.4067782	5.4985542	6.0067796	6.02371974	6.01016571	5.93177046	5.56857828	6.14541944	6.48666562	5.32673094	6.10084958	6.51372362	3.97820436
hsa-miR-4633-5p	2.8567273	2.6431375	2.6636227	2.7872855	3.1123594	2.7074275	2.8077397	2.8943755	2.7612169	2.7402337	2.955504	2.771134	2.718043	2.94392056	2.9575913	3.30237237	3.85840716	3.06945108	2.97599658	3.14528776	3.05494996	3.10483135	3.27181448
hsa-miR-4634	3.8060083	3.2966962	3.6387841	3.5611552	4.1161729	3.5721791	3.636909	3.3142165	3.703675	3.0799815	4.5363825	4.2359616	3.7651284	4.37210644	4.0096546	6.33971761	6.59187315	3.82049263	3.49760278	5.14834252	4.32249102	4.38115308	5.47638096
hsa-miR-4646-3p	3.6854318	3.4360353	3.7131568	3.2662853	3.5166933	3.4672373	3.2961866	3.2329338	3.5867926	3.7060057	3.2446888	3.3589576	3.3427861	3.41936958	3.60409586	3.34152276	3.3213626	3.60252856	3.63576729	3.42513861	3.47375268	3.55433272	3.23361253
hsa-miR-4649-3p	4.8745869	4.8563337	4.7842371	4.5319214	4.7359178	4.6198758	4.4138415	4.6187888	4.8564928	4.8106239	4.1297772	4.5082776	4.5472474	4.39827127	4.68702447	4.26358144	4.13473786	4.73915389	4.74118499	4.49795653	4.44155126	4.70905354	4.03496918
hsa-miR-4651	2.8542102	2.9929579	3.02963	2.8939369	3.1233769	3.0844506	3.1062738	3.0070482	2.9033052	2.8848584	3.9911832	3.7326803	2.9257634	3.60418631	3.55079097	3.24175269	3.47433143	3.22482148	3.13431654	3.73758665	3.40608407	3.44091871	3.60398531
hsa-miR-4652-3p	3.7368529	3.4831665	3.7617275	3.336521	3.6421407	3.304659	3.404275	3.4019844	3.7615709	3.5424214	3.2921202	3.5758353	3.7682325	3.3475442	3.59573786	3.18123752	3.2736574	3.40382846	3.45063084	3.41532408	3.52606362	3.40868382	3.43654611
hsa-miR-4653-3p	5.6158759	5.5601549	5.8337896	5.4864079	5.7906652	5.4257566	5.4739025	5.4249925	5.6852163	5.5730537	5.9484428	5.9080249	5.5044964	5.82301124	5.64825972	4.98975325	5.30211815	5.53872571	5.51146572	5.54783536	5.53530391	5.69584133	5.07033128
hsa-miR-4656	3.6831689	3.6251698	3.9914186	3.4270786	3.8297763	3.683759	3.6006835	3.6657492	3.481432	3.8510075	4.1652972	4.3129686	3.6913469	3.91182719	3.70921808	3.7482071	3.94100856	3.74813149	3.81295874	4.12202702	3.77280361	3.82300178	3.77640021
hsa-miR-4664-3p	4.1908679	4.0267557	4.4972847	4.1951522	4.4198672	4.3139149	4.1288194	4.0145713	4.402055	4.3357772	3.7249524	3.8619174	4.1541846	4.1017341	4.35425204	3.90349173	3.91617329	4.33004932	4.37518032	4.18268002	4.2440773	4.32680404	3.75967344
hsa-miR-4665-3p	5.2738619	4.8209721	5.4709691	5.1823717	5.3240481	5.2258415	5.0358147	5.1550539	5.5304476	5.2321133	4.8214203	5.2386073	5.3011485	5.13803812	5.24404293	4.74661949	4.80612682	5.09945684	5.10584331	5.03903455	5.12915116	5.06658563	4.72030511
hsa-miR-4666b	3.672076	3.6018818	3.9817516	3.5891196	3.6802248	3.7541815	3.6111758	3.5150132	3.6798626	3.9115639	3.1677759	3.6107252	3.5683956	3.57903163	3.83520172	3.57443854	3.44420471	3.76704076	3.92751066	3.5309422	3.81387004	3.75538692	3.26667354
hsa-miR-4667-5p	3.0381929	3.0222958	3.043054	3.1817449	3.0713253	3.0975362	2.9615343	2.9545333	2.813404	2.9527251	3.7438483	3.6776733	2.9302998	3.40146017	3.00927346	4.30376743	4.82511509	3.45538666	3.21217186	4.09099446	3.34049033	3.54254552	3.93120647
hsa-miR-4669	4.6170726	4.5521494	4.5261453	4.6233899	4.8290995	4.5372191	4.7697732	4.4684527	4.069964	4.5364384	5.2139169	5.2015683	4.5854089	4.69556224	4.25408364	4.88373101	5.17139873	4.44640545	4.37466811	5.10750827	4.58879071	4.64912712	4.92015657
hsa-miR-4672	5.1476801	5.284235	5.7205744	5.0405425	5.5253335	5.2342221	5.155072	5.3988913	4.7315227	5.2785904	6.898161	6.8589494	5.1820353	5.53273014	5.07823069	5.16601306	5.3158877	5.47453342	5.45900107	6.01512527	5.53461419	5.76968614	5.93316538
hsa-miR-4687-3p	5.2585087	4.7438847	5.1665753	4.9733815	5.1947972	4.8902793	5.0172329	5.328727	4.9939335	4.658005	6.1373341	5.6262002	5.0197681	5.63239813	5.18349477	6.86574936	5.69361982	5.09434958	5.03616356	5.53276124	5.57518872	5.12291991	7.09213221
hsa-miR-4695-5p	3.3913111	2.8661094	2.9690759	2.9415674	3.3442359	2.9010186	3.2504266	3.0735384	2.7945691	2.9027993	3.7586748	3.4745329	2.9724417	3.44695401	3.01754005	3.59275824	3.27200202	3.53134584	2.94012449	3.57040382	3.46075734	3.35848802	3.59978419
hsa-miR-4697-5p	2.7347599	2.8112287	2.8377112	2.6693023	2.8793881	2.6707413	2.6224636	2.7109836	2.560272	2.7924243	3.5599113	3.3486465	2.7061981	3.14647944	2.77586606	2.91072525	3.12957516	3.05108975	2.87045536	3.31079099	3.03320245	2.96047474	3.38832926
hsa-miR-4698	3.7938486	3.6697335	4.0727626	3.7078135	3.8696004	3.8611388	3.6562986	3.7837368	3.8083423	3.7505127	3.9026518	4.0486558	3.7707428	3.90315247	3.83432842	4.01368429	4.21794215	3.9002391	3.9052086	4.18277511	3.88566001	3.90364442	4.08085337
hsa-miR-4701-5p	3.5468893	3.5939785	3.5854547	3.2298622	3.5900528	3.7184167	3.3657577	3.524973	3.4562795	3.3629858	3.3390141	3.4314884	3.5843694	3.53474172	3.59418148	3.2978263	3.40980814	3.77446402	3.53173613	3.52873046	3.73164886	3.71556551	3.26892013
hsa-miR-4713-3p	5.6271159	5.4397537	5.8805328	5.4984045	5.7432776	5.5326227	5.4072563	5.6588816	5.9583754	5.4373221	5.7677668	5.7226863	5.1469604	6.07695912	6.07500424	5.35535125	5.57559077	5.76995453	5.71215674	5.87531269	5.68683492	6.03036495	5.41281125
hsa-miR-4716-3p	4.1221171	3.9756079	4.297587	3.862726	4.2926536	3.7594109	3.7957149	3.9794193	4.0122985	3.8060736	4.3724057	4.4115259	3.5800535	4.78355513	4.47265952	4.2150338	4.42083444	4.39944134	4.3694124	4.61102942	4.34029999	4.63997841	4.40106242
hsa-miR-4721	5.7786528	5.6299728	6.2432081	5.7215341	5.9534249	5.8962151	5.7990365	5.69509	5.6707201	6.1888772	6.7330134	6.6971254	6.0336727	5.94644208	5.7425391	5.40178094	5.42425238	5.77113342	5.85650839	6.11994419	5.84478211	5.87838016	5.5606248
hsa-miR-4725-5p	4.5002259	4.2544541	4.682469	4.3307837	4.7339077	4.5002951	4.2839062	4.2909193	4.6451021	4.5581826	3.8524478	4.2998581	4.3930421	4.44228968	4.56031138	4.04274003	4.182079	4.44133678	4.61717271	4.38275847	4.4440339	4.53420797	3.99246897
hsa-miR-4728-3p	3.2823254	3.3007028	3.62115	3.2077257	3.4167936	3.3774353	3.3857078	3.2098463	3.3620633	3.3496429	3.1754841	3.3102369	3.2356235	3.47549176	3.60801205	3.30126739	3.50535875	3.54316029	3.5433828	3.64638579	3.40600936	3.43191923	3.37800854
hsa-miR-4728-5p	5.322011	5.1133883	5.4441495	4.8859704	5.3443146	4.9912901	4.9402086	5.0597649	5.0691614	5.1284315	6.1390258	6.0974541	5.1892776	5.50833365	5.28458762	5.27545589	5.19818239	5.46462093	5.372759	5.74593364	5.47349083	5.58729329	5.26895935
hsa-miR-4739	4.8662682	4.1462058	4.5962684	4.4658388	4.5668521	4.8049626	4.6765655	5.3091915	5.0099975	4.4455643	5.1125049	4.6450111	4.6337189	5.50665194	5.31578811	5.19467743	5.10744009	4.53566589	4.56418742	5.14099472	5.12596093	4.33799554	4.88286939
hsa-miR-4741	4.3816021	4.6194763	3.95954	4.3592154	4.7938032	4.3477507	4.4590432	4.206676	4.0664231	4.1029078	5.1275657	4.8116068	4.5221	4.9738916	4.57569839	6.62181536	6.99207841	4.82745081	4.28497878	5.68201321	4.73602234	5.39082141	6.02497387
hsa-miR-4745-5p	3.3793879	3.3107923	3.2286705	3.3792355	3.5989849	3.1878019	3.3205677	3.5355507	3.267008	3.24291	3.6591346	3.4394514	3.1411545	3.80578559	3.63743942	3.38151269	3.48401344	3.42708093	3.5145315	3.69829348	3.59629063	3.44633907	3.74746544
hsa-miR-4746-3p	4.1587324	4.0471966	4.2657066	3.9253394	4.2012404	3.996093	4.1303229	4.1032899	3.8515305	4.0723741	5.2665418	5.0529317	3.9752791	4.69354564	4.44385399	4.21269219	4.29614801	4.2215369	4.14357215	4.84281092	4.25575468	4.27279138	4.6516741
hsa-miR-4749-3p	4.0308578	3.843962	3.9850665	3.7570199	3.8830883	3.9539263	3.7943297	3.7046696	3.9431049	3.9252908	3.5917897	3.9381389	4.043644	3.87447084	3.96369206	3.64945047	3.61439311	3.96804103	3.89768929	3.85141903	3.88548502	3.88687892	3.58735832
hsa-miR-4758-3p	4.3258401	4.1027854	4.3637797	3.9982371	4.1307574	4.2487231	3.9408357	3.8244623	4.2480861	4.4578845	3.6655019	4.0651972	3.9823526	4.13629228	4.30880699	3.89131565	4.0200208	4.23904655	4.34372582	4.1032336	4.21381088	4.16324376	3.74782711
hsa-miR-4763-3p	7.294696	6.2451244	6.5200968	7.5060997	6.9076663	7.256245	7.9756053	7.756026	7.1790067	7.016797	8.2454323	6.9621228	7.6280126	6.74251396	6.74718428	6.97612729	6.9764308	6.69521477	6.79923456	7.53910492	7.49614024	6.69479395	6.09055789

hsa-miR-4767	3.3315339	3.0801919	3.2015768	3.1439817	3.4029294	3.1622474	3.1994596	3.0561466	3.2943685	3.1519318	3.4367572	3.4952255	3.0688479	3.31440017	3.45660471	3.37419872	3.49992031	3.42087566	3.39878842	3.44920276	3.74552331	3.25770703	3.53724395
hsa-miR-4769-3p	3.6116348	3.5867197	3.8150183	3.6100482	3.8015747	3.5443922	3.4924715	3.6583675	3.6182717	3.6027195	3.3835699	3.5221858	3.6210354	3.75690032	3.78442082	3.45958733	3.43300382	3.77919805	3.74193179	3.54367917	3.78279991	3.61181765	3.41186474
hsa-miR-4787-3p	4.7619567	4.3429746	4.9108761	4.4386237	4.6596246	4.449403	4.3692505	4.417057	4.6406546	4.5907365	4.3424482	4.5373668	4.4681025	4.59257097	4.77194919	4.30017913	4.34386684	4.63262098	4.54092901	4.61268349	4.58871205	4.71767002	4.61932693
hsa-miR-4787-5p	4.6640795	4.5347978	4.7694532	4.5509514	4.9286286	4.4318392	4.7744756	4.6933659	4.6256625	4.8758912	5.1639986	5.0151517	4.5637447	4.81601703	4.59603589	5.13698437	5.45846818	4.93181696	4.89481851	5.20080129	4.95479716	5.1856818	5.17397707
hsa-miR-4788	4.3084185	4.0245113	4.1220588	3.9466421	4.4218814	4.050649	3.9908037	4.1441495	3.9353961	4.2562419	4.6748366	4.7402258	3.9970928	4.45546179	4.16947413	4.46977672	4.68007194	4.19008024	4.1256513	4.7096804	4.36069175	4.40978062	4.33076623
hsa-miR-4793-5p	3.5993745	3.1542318	3.4195348	2.8726317	3.3756742	2.9772068	2.9991207	2.9148636	2.8559663	3.1124347	3.6678466	3.6300558	3.0699715	3.44272859	3.18374573	3.70641227	3.9784785	3.36770382	3.38954566	4.02736582	3.61957348	3.54407971	3.63296717
hsa-miR-4800-5p	3.7361256	3.2799703	3.650891	3.1730455	3.5211066	3.5300555	3.4379794	3.4404549	2.7252601	3.3341298	4.1137726	4.2465143	3.3749185	3.68662713	3.17171696	4.81831153	4.86643841	3.65120048	3.61439249	4.55010268	3.7539084	3.88034792	4.25059887
hsa-miR-483-3p	5.9537994	5.2736852	5.0206221	4.9243121	5.0761729	4.7991379	4.7160299	5.1709507	5.0733021	4.9743492	4.5507819	4.8509577	5.0732448	4.7264304	5.0217102	4.58366512	4.70555523	5.31288653	5.40889471	5.15875369	5.15187388	5.30157386	3.65214067
hsa-miR-483-5p	5.9644543	5.0030093	5.0555351	4.8732614	5.1341635	4.8907344	4.9290505	5.172982	5.3211122	5.0448292	4.7956227	4.9210903	5.0323642	4.9392663	4.76524635	5.40806091	5.64538104	5.506738	5.44150171	5.28925145	5.39567531	5.38580463	4.29733353
hsa-miR-484	4.5998761	4.7784428	4.6932523	4.4079131	4.4928842	4.3352232	4.2361125	4.8179282	4.6482409	4.4056945	4.0570063	4.3015933	4.4015291	4.62697422	4.69560563	4.31947788	4.17132004	5.00730605	4.55107313	4.42269286	4.89658697	4.68070534	3.96523239
hsa-miR-486-5p	5.4319136	5.7039773	5.1077718	4.7384929	5.2839199	5.36567	4.3736656	4.6983295	6.2671541	5.8584368	6.4036149	4.4654547	4.8543902	4.70088731	5.91010986	5.22561173	4.29521067	4.6005888	5.15061846	5.20533073	5.09362519	5.15421433	8.90612806
hsa-miR-487b-3p	4.4775941	4.6674798	4.2843221	4.5936801	4.6405548	4.0564797	4.4350427	4.7554922	4.272239	4.246054	4.1992843	4.1193663	4.6332419	4.92995581	4.85847486	4.49970574	4.63264249	4.89801705	4.88063579	4.35244335	4.80078376	4.81884319	5.10988311
hsa-miR-494-3p	10.206044	10.471217	11.366088	10.484562	10.595859	11.010672	10.651545	10.358942	10.180916	10.474548	12.803204	12.736856	10.320798	10.8771276	10.2418184	10.633278	10.5722589	10.7338675	10.4601803	11.842794	10.7851238	10.9957625	11.1617831
hsa-miR-495-3p	3.1000596	3.3048239	2.9339532	2.9928619	3.1370515	3.0510342	2.9472026	3.1869729	3.0187784	3.2433006	3.0967991	2.9961962	3.2108808	3.5194134	3.80071697	3.54553426	3.16528664	3.60558466	3.36567065	3.20876817	3.42047767	3.33813381	4.36283368
hsa-miR-497-5p	7.3066638	7.5622194	7.7950256	7.4358498	7.4989786	7.5991855	7.3678574	7.5807912	7.5605442	7.6384996	6.8109419	7.6902409	8.0799413	7.74072042	7.81509668	7.84289942	7.59513983	7.84896372	7.8966944	6.85890288	7.6131513	7.88690444	7.67210458
hsa-miR-499a-5p	3.7146275	3.7192749	3.4009316	3.1940417	3.5331717	3.0963319	3.3097689	3.5793474	3.6928072	3.9076561	7.855804	3.3095483	3.8429175	3.50900163	3.5501251	5.47884713	3.32144095	3.58892784	4.09331823	4.03921679	3.93150287	3.77189499	10.5853358
hsa-miR-5001-5p	3.7240864	3.8446368	3.5217577	3.6690465	4.0969188	3.6183721	3.5973284	3.6286825	3.6022707	4.3559401	4.4561309	4.2399623	3.6115463	3.70848438	3.51089135	4.26503568	4.35077533	3.87248824	3.59386716	4.43370619	3.99829178	4.20932793	4.63652382
hsa-miR-5006-5p	5.3717271	5.1860735	5.5557967	5.1949626	5.494477	5.1311123	5.444472	5.2985495	4.7372579	5.2685436	6.3777026	6.1351867	5.3288294	5.5719599	5.25765985	5.40001436	5.61838307	5.40369286	5.43320792	6.01111031	5.64573323	5.57879729	5.18693531
hsa-miR-500a-3p	3.1765925	3.3320493	3.1488752	3.0382917	3.0849758	3.2802787	3.1388568	3.6142562	3.2839877	3.3621625	3.1362734	3.1014823	3.4861715	3.62166557	3.56464728	3.43399585	3.48828951	3.61481647	3.48050067	3.29654482	3.62803618	3.41525261	3.96952633
hsa-miR-500a-5p	2.9022035	2.6680587	2.7490176	2.6069054	2.8664721	2.7664335	2.728789	2.7806414	3.0491823	2.7529365	2.944987	2.7450509	2.7783278	3.40686909	2.94081014	2.99313864	2.99714123	2.94183135	2.9152212	2.91105721	2.94378507	2.93849725	3.16599244
hsa-miR-5010-3p	3.823363	3.1200701	3.9747424	3.4697149	3.5865248	3.5545358	3.412006	3.5063129	3.4883446	3.3707025	3.3168054	3.3751786	3.4763615	3.63903016	3.73937147	3.37793325	3.40639103	3.69803188	3.60103901	3.3482362	3.60398287	3.65545244	3.31683063
hsa-miR-502-3p	3.1135774	3.1327937	3.3333827	2.9732873	3.2109923	2.9999851	3.1276707	3.7145333	3.2341168	3.0742364	3.1102625	3.1669914	3.3979811	3.91704165	3.73415614	3.52303421	3.61758262	3.72314108	3.39523742	3.25619052	3.77713907	3.5194054	4.19523574
hsa-miR-503-5p	2.9816971	2.8115206	2.8174763	2.5959765	3.128311	2.8671955	2.6328677	2.9338293	3.024208	2.9515104	2.8986223	2.8014685	2.8033446	3.29483717	3.27764758	2.94907842	3.00520885	3.200298	2.85172313	2.79002281	3.09715026	3.12309196	2.67664111
hsa-miR-505-3p	5.5968767	6.0560753	5.9095416	5.7071305	5.9446897	5.769143	5.9728528	6.175594	5.9997213	6.082131	5.497212	5.343702	6.0607726	5.61278714	5.82895438	5.68240473	5.79992997	6.1340886	6.33885216	6.12004715	6.4976193	6.10936668	3.88595834
hsa-miR-505-5p	3.5420051	3.8986833	3.7908817	3.5121516	3.8449333	3.7258258	3.6945078	4.0197326	3.9714723	3.8458511	3.5025174	3.3296436	3.7486455	3.94840827	4.02917474	3.77486587	3.77749622	4.07171145	4.23884941	4.00756379	4.51023543	3.98698758	2.99590074
hsa-miR-5088-5p	3.6992644	3.6527664	3.6511011	3.4814922	3.6683387	3.6491014	3.7273635	3.744766	4.1533857	3.6162211	3.7349781	3.6201241	3.0451745	4.39073368	3.67988113	3.586542	3.83676634	3.81213946	3.63834556	3.84340172	3.85719715	3.72953726	4.02599053
hsa-miR-5100	12.489744	11.894857	12.018605	12.661153	12.529901	12.282798	11.787527	12.675492	12.546255	12.078306	12.232419	12.936989	11.968261	12.016381	11.7787325	11.9497172	12.3421512	12.3426695	12.4877261	11.8063441	11.9504957	12.3477996	12.1849891
hsa-miR-511-3p	3.5013652	3.3337154	3.210737	3.266548	3.1545292	2.9878185	3.2133109	3.0876522	3.788476	3.0710418	3.3109205	3.1165007	3.3840824	4.11022999	4.21988383	3.32511916	3.65758833	3.55311144	3.74318396	3.25929504	3.39594979	3.5512242	3.01611962
hsa-miR-513a-5p	5.4682319	5.3142273	5.6567902	5.0551465	5.6184497	5.3743948	5.290168	5.2808919	4.9797592	5.4434951	6.2663695	6.2356295	5.3169861	5.56982008	5.39850676	5.02712565	5.26307489	5.50534631	5.48157571	5.91565749	5.46516553	5.58113374	5.22676522
hsa-miR-513b-5p	4.0437053	4.1613791	4.5386454	3.75423	4.2973445	4.0585772	4.1288091	4.1387919	3.828992	4.2871169	5.0727725	5.1107396	3.958621	4.57808765	4.42828361	3.90756707	4.16067648	4.44473179	4.39632906	4.89023002	4.45978012	4.58593175	4.31440167
hsa-miR-513c-5p	3.4960696	3.4600962	3.5714485	3.0138822	3.3382634	3.1038276	3.1009258	3.2474853	3.0885262	3.2809916	3.9770524	3.9742808	3.2732189	3.77431512	3.40762654	3.34339559	3.41496162	3.4777311	3.4778233	3.90588583	3.84570875	3.53340853	3.58052479
hsa-miR-5194	3.8607466	3.6662483	3.8730817	3.6463891	3.5879527	3.413686	3.4558853	3.6895233	4.0350933	3.7970335	3.8844583	3.9493718	3.4180064	4.13800375	3.99996041	3.66853669	3.88600323	3.90571075	3.99864299	4.14104392	3.83733186	4.11548167	3.99028464
hsa-miR-532-3p	3.7036334	3.874532	4.0619781	3.7902806	3.8795718	3.642308	3.6554936	3.9653115	4.1052216	3.6308543	3.5048284	3.6313929	3.8758415	4.56091904	4.54690628	3.967083	4.06931612	4.30593605	3.99211132	3.86406181	4.14990212	3.9241598	4.57177396
hsa-miR-532-5p	4.6682267	5.0323652	4.9343435	4.7558179	5.0184288	4.4276692	4.6280762	4.9786882	5.1676179	4.7943905	4.2449737	4.3396923	4.9041477	5.22514811	5.38750376	4.93303035	4.92621753	5.25597555	5.05681141	4.58206104	5.1441303	4.94571115	5.45156511
hsa-miR-542-3p	4.2407369	4.3566904	3.9620659	3.389569	4.5099709	4.0659789	4.0215438	3.9328512	4.0040652	4.2890613	3.9725329	4.0249662	4.4779571	4.36694518	4.86759188	4.37532448	3.93745406	4.42128897	4.42941345	4.37276045	4.59462661	4.59048192	3.28521858
hsa-miR-542-5p	3.3654217	3.4031405	3.1125676	2.9872233	3.7645439	3.2860717	3.1412763	2.9793749	2.9708724	3.2835446	3.1547232	3.2905438	3.29821	3.77631886	3.95852218	3.31038318	3.30943173	3.48355483	3.45396786	3.02471672	3.71600972	3.42075928	3.1041259
hsa-miR-550a-5p	3.9788933	3.9537224	4.3410754	3.9278148	4.1285226	3.9269842	3.9658666	3.7036636	4.0693233	4.1200619	3.533635	3.9750975	4.028552	4.0976399	4.1633628	3.80313507	3.82939134	3.96458691	4.22996314	3.94356336	4.10673195	4.17750954	3.59785038
hsa-miR-551b-3p	2.9894058	2.8154592	2.9086288	2.7134436	3.0763939	2.989309	2.9467202	2.9478912	2.8952654	2.7512386	3.1012042	3.1640707	2.9958764	3.09246803	2.9760392	3.28437091	3.33997087	3.33289817	3.03651335	2.96738216	2.77290772	3.2049449	4.05347157
hsa-miR-557	3.4801924	2.8465876	2.7594868	4.0222176	3.5198258	3.7619592	3.8643658	3.9100709	3.3472808	3.2862793	4.7944284	3.5206636	4.2994858	3.1388876	2.67466787	3.47338302	3.21496509	3.322145	3.31493034	3.83414031	3.58735848	3.19129108	2.93532069
hsa-miR-5571-5p	3.7866778	3.3088002	3.8783156	3.4782341	3.8391746	3.5791614	3.535545	3.6418281	3.7267663	3.7356433	3.3095386	3.5683828	3.4333681	3.7714915	3.79722937	3.39197415	3.55542941	3.77031581	3.68980819	3.51489273	3.70638664	3.72497103	3.39894472
hsa-miR-5581-5p	4.1286216	3.9424313	4.1556959	3.815029	4.3528925	4.0149503	3.7680271	3.9974209	4.196782	3.9934964	4.3858524	4.4348573	3.3866507	4.83452324	4.5311147	4.30927591	4.39474723	4.50308531	4.20869356	4.6339349	4.37898532	4.51699815	4.39859956
hsa-miR-5585-3p	3.5270657	3.2541594	3.8201075	3.3397837	3.2455714	3.69023	3.4580614	3.5205084	2.6985325	3.3430078	4.549252	4.9906913	3.4689983	3.90583011	3.29699583	4.95817625	5.05880431	3.76460417	3.81052927	5.07577748	4.0184072	4.32864242	4.69882704
hsa-miR-563	3.5361725	3.4753613	3.8630419	3.6967353	3.7977923	3.7034887	3.8434847	3.5676257	3.8208491	3.7380838	3.1281356	3.5755956	3.6122718	3.58830142	3.89328742	3.56378552	3.40267329	3.73981126	3.67696218	3.41867362	3.82559565	3.7707456	3.34887203
hsa-miR-564	4.4060397	4.3522051	4.2899426	4.0946412	4.6921533	3.8000227	3.9497265	4.232076	4.0690398	4.4681392	4.3795833	4.2170308	4.2031299	4.50162632	4.28144766	4.12907993	4.32645758	4.33796911	4.36499528	4.30507686	4.29840393	4.52458815	4.16240169
hsa-miR-5684	3.490778	3.2131693	3.2232356	3.0777115	3.4120791	3.2169845	3.1739519	3.316874	3.3772046	3.4188477	3.62939	3.6031627	2.8972091	3.94352022	3.690313	3.51781238	3.44067567	3.86970209	3.24807786	3.57353557	3.40723428	3.33789083	3.97905861
hsa-miR-5699-5p	3.1265926	3.246124	3.5974812	3.0929304	3.4062818	3.2539521	3.3229982	3.2781065	3.0401176	3.2752456	4.215661	4.2457784	3.3850742	3.58125551	3.6458653	3.54487475	3.46590535	3.60669504	3.56017892	4.1320779	3.55844749	3.66287086	3.71855757
hsa-miR-5701	3.6071702	3.741693	3.6816733	3.5407133	3.8264601	3.4079362	3.3688958	3.889566	3.8592661	3.896101	3.3076843	3.1917382	3.821204	3.97426998	4.07825845	3.43029431	3.7428953	3.84936168	3.9934458	3.94819169	3.99489851	4.07661951	2.90888703
hsa-miR-572	2.9597074	2.7673363	2.7411262	2.9390593	2.9573152	2.8549703	3.1204073	2.8339786	2.982986	3.8218879	3.5793648	3.0577707	3.0992783	2.83213486	2.74236939	3.32417913	3.26813198	2.69800106	2.81941777	3.3063367	2.97815519	3.00418274	3.57469047
hsa-miR-5739	9.4250248	9.485355	9.096798	9.584288	9.6078736	9.2097673	9.4838743	9.5638814	9.2155872	9.2324049	9.3762628	9.5116607	9.3335702	9.25809885	9.13791427	9.62551885	10.364999	9.49464365	9.44204368	9.58312764	9.34996983	9.62093119	8.83212044
hsa-miR-574-3p	6.8243464	6.8961002	6.8834984	6.9082551	7.1198247	6.83401	6.7531858	6.8045112	7.0794179	7.0835239	6.2137096	6.6079639	6.7077278	6.46541824	6.59674489	6.44128769	6.49350207	6.67587627	6.9198654	6.51206792	6.85612553	6.91475404	5.56776935
hsa-miR-574-5p	6.4400287	6.3012577	6.3868552	6.4774182	6.4793316	6.7056909	5.857213	6.7686887	6.710431	6.1848554	6.1742396	6.3075946	6.5829306	6.56605706	6.86602401	6.82266547	6.73908066	6.45607755	6.28708298	6.20977377	6.34328071	6.97417033	6.12154226
hsa-miR-575	4.3339745	4.2091211	4.3253283	4.0267989	4.3380848	4.1132125	4.1060823	4.0597454	3.9886468	3.9116352	5.3388365	4.8421701	3.852686	4.81997546	4.43939509	5.32924641	5.54510967	4.52125583	4.42035152	4.9636966	4.64941519	4.62008102	5.6510479
hsa-miR-5787	5.0931817	4.5210456	4.8987072	4.7589427	4.8062657	4.9671519	5.0560555	4.890549	4.7455968	4.7260192	6.054926	5.6032304	4.7285015	4.96239238	5.01198032	6.69898215	5.83875484	4.74230409	4.78031312	6.03958342	5.42528368	4.93783966	6.42578405
hsa-miR-582-5p	3.9023735	4.1437275	4.1930867	4.0598926	4.0258168	3.7595223	3.7855948	3.9449901	4.3224545	4.3323766	3.254379	3.8041383	4.0611483	4.11861442	4.45669498	4.11498498	4.42835368	4.75255124	4.32462537	3.89664521	4.37853033	4.21902043	3.04433033
hsa-miR-590-5p	5.3397931	5.194989	4.9538942	4.957545	5.1266456	4.7499854	4.7038584	5.050194	5.4277286	5.231457	4.9806955	4.6925938	5.4461322	5.31295491	5.50825019	5.16533109	5.07240677	5.28119624	5.61335168	5.03274633	5.20395202	5.50568999	6.23090438
hsa-miR-602	3.8152103	3.561729	3.8950304	3.5134743	3.7167732	3.4993253	3.5050201	3.4701147	3.5403772	3.605392	3.3831508	3.8997213	3.4694832	3.68999034	3.5936503	3.41800229	3.64240961	3.78334615	3.82300565	3.9255334	3.96656689	3.61355354	3.50445204
hsa-miR-6068	4.58286	4.1432995	4.1009069	4.6261711	4.6402616	4.580576	4.9049319	4.469567	4.8173046	6.6157875	5.7870272	5.0779364	5.5451587	4.14127506	3.81391028	5.10868616	4.65792089	4.308604	3.99885225	5.31281196	4.73413445	4.76635042	5.64464911
hsa-miR-6069	4.9807566	4.54484	5.0600237	4.7512889	4.872947	4.8613061	4.6608004	4.7081441	4.9067981	5.0197773	4.3015105	4.6920051	4.952002	4.766	4.87153159	4.40191817	4.54282783	4.77908874	4.82461224	4.65135165	4.7499809	4.78388433	4.29283216
hsa-miR-6075	2.6975658	2.6857882	2.716757	2.7148122	2.8614286	2.5532171	2.6772012	2.9354133	2.7967141	2.8363413	3.2970781	3.0382992	2.8351167	2.90804705	2.83107017	2.79904379	3.00338856	2.79389286	2.7970863	3.13504978	3.02592362	2.93930651	3.12321695
hsa-miR-6076	5.0753094	3.8049703	4.651586	4.3999952	4.9379426	4.001941	4.5346693	4.3686196	4.9963018	3.8710748	5.1476224	4.6386228	4.0852249	4.76814547	4.27886098	5.69734797	5.73460623	5.28918203	4.02328337	5.88481787	5.00567686	5.49854936	4.82517829
hsa-miR-6085	9.8330654	9.8033298	9.4914033	9.8373345	9.9591364	9.4923125	9.644818	9.907803	9.4551153	9.5331359	9.5987788	9.7481568	9.6069165	9.44763865	9.42876462	9.23855602	9.25060523	9.82345946	9.80149006	9.36016229	9.58949928	9.59224865	9.11485004
hsa-miR-6086	3.3344987	3.300121	2.9521611	3.0880178	3.3328285	3.0568083	3.3064864	3.1138114	2.8271224	3.1071875	3.9067329	3.8903623	3.1509869	3.46482419	3.07850104	3.98983335	4.02889487	3.30865633	3.2175527	4.03153584	3.40846097	3.36709354	3.93471623
hsa-miR-6087	8.2400767	8.1491637	8.676951	8.1394874	8.3609734	8.5547633	8.3806561	8.1886694	8.0953895	8.1405396	9.5194041	9.8169158	8.1511638	8.09983974	7.88759921	8.61181916	9.25622701	8.02840553	8.01573957	9.00686977	8.31537245	8.34210757	8.78425839
hsa-miR-6088	6.6308836	6.3304042	6.5653709	6.9967626	6.7803509	7.2735767	7.2211933	6.8260114	7.1886764	6.6917917	7.4943676	7.0405091	7.2185719	6.61016856	6.1728193	6.89101517	6.68521113	6.0257371	6.36078375	6.81908829	6.99709706	6.51616876	6.79113419
hsa-miR-6089	9.2016638	8.7456366	9.0439143	9.1753955	8.9994405	9.2645477	9.44581	9.2537581	9.0331384	9.5440903	9.8707608	9.7919977	9.2516525	8.96334908	8.71075535	10.4559537	10.6534222	8.79072431	8.61181103	9.64236892	9.27007388	8.88538558	10.3050009
hsa-miR-6090	8.315146	7.2355633	8.1056658	7.9451203	7.884319	8.2955532	8.109269	8.6136325	8.1876986	7.5920542	8.5985382	8.178869	8.1297664	8.02700898	8.08492974	8.21002948	8.17070997	7.42397537	7.61931874	8.4096431	8.34371341	6.964558	8.35276979
hsa-miR-6124	5.4194319	4.9184555	5.0778378	5.444117	5.4350381	5.3724728	5.3033552	5.3582262	5.6230215	4.9352953	5.5135132	5.3245603	5.3846979	5.47647532	5.25768114	5.89912069	5.7151144	5.2854391	5.16494934	5.75307187	5.47944539	5.4158392	5.28505953
hsa-miR-6125	6.8736188	6.9483825	7.0066575	6.8870534	7.1251313	6.8367171	7.1014268	6.999032	6.8120452	7.4521653	7.8142865	7.9665858	7.0330606	6.94448839	6.75116326	7.52767927	7.87027817	6.97250857	6.84443881	7.52326719	7.14702386	7.21269501	8.04467613
hsa-miR-6126	4.5628974	4.1021759	4.3436683	4.0309789	4.5607679	4.0672682	3.9475926	4.2669733	4.0465666	3.9488484	4.9424998	4.8596937	4.0744274	4.70647822	4.54534958	4.22071573	4.64907802	4.56214203	4.56128074	4.74382955	4.60950229	4.6249421	4.90197801
hsa-miR-6127	8.612221	8.394572	8.799327	8.4125235	8.4832665	8.7033016	8.6922797	8.2371161	8.811164	8.4052278	8.5787063	8.9512734	8.1972257	8.56504937	8.25180367	8.02529526	8.15319228	8.37418299	8.07802419	8.54626106	8.05980512	8.22369349	8.07059821
hsa-miR-6131	5.3478165	5.2058586	5.5538627	5.1479043	5.3384271	5.2073889	5.0223254	5.1244164	5.8043737	5.2126835	5.0803317	5.438101	5.0687915	5.93385075	5.53411206	4.88002109	5.2484332	5.334969	5.33271424	5.42476527	5.34502512	5.58755806	5.00932933
hsa-miR-6132	5.8815116	5.8623956	6.3695889	5.6322715	5.9340866	5.9204612	5.7017235	5.6487619	6.050807	5.9210765	6.866055	6.763303	5.7162366	5.88428485	5.798321	5.32761654	5.42307789	5.76899054	5.7229029	6.13993431	5.82729731	5.94509444	5.48774955

hsa-miR-6165	8.1791766	7.5290012	7.2609154	7.6680733	7.7256979	7.1414114	7.3126427	7.773761	7.3737371	7.2324985	7.178264	7.3271978	7.2835838	7.33799355	7.26063877	7.28917673	7.45638898	7.92547774	7.66138144	7.14712064	7.30408121	7.48641863	6.8311719
hsa-miR-619-5p	3.541283	3.190225	3.1834803	3.4235338	3.3531981	3.3331835	3.4027054	3.3160256	2.5821867	3.0879056	3.7301513	4.2469142	3.4913141	3.81170862	3.26343281	4.70923946	5.16972739	3.64776393	3.22617986	4.50296662	3.71204061	4.00955294	3.39128771
hsa-miR-625-5p	2.7871935	2.684247	3.1402751	2.8387684	2.7847446	2.6302255	2.723638	2.8937127	2.8034671	2.947508	2.8969114	2.8473188	2.9220713	3.63815983	3.32844279	3.16855891	3.22827312	3.41352374	3.18856974	2.85295679	3.13610737	2.97122704	3.22137498
hsa-miR-628-3p	3.3810053	3.4831649	3.2669154	3.1079039	3.4563218	2.998369	3.2921267	3.3093995	2.9474786	3.6033989	3.2401849	3.2127539	3.5018059	3.47252591	3.07269111	2.99355129	3.02429273	3.36249332	3.1671754	3.36101543	3.38425795	3.63290097	3.65592479
hsa-miR-634	3.5796042	3.6124293	3.6099517	3.3183921	3.4223005	3.4003502	3.0983016	3.3788827	3.2990451	3.3391893	3.3393771	3.2373246	3.354639	3.30455249	3.50380766	3.25346878	3.24593726	3.45796606	3.39250156	3.35637779	3.15058769	3.37485486	3.24768014
hsa-miR-638	5.6213988	5.3613135	5.5278016	5.8868271	5.6906382	5.7727803	6.1741993	5.7136083	6.4673031	7.7249272	6.7848096	6.3286029	6.4183819	5.398749	5.05770917	6.18686347	5.76151606	5.29377837	5.35457712	6.23734382	5.95469787	5.87353363	6.31010443
hsa-miR-642a-3p	6.9377629	7.0519739	7.8093646	6.8673474	7.1702387	7.4642589	7.0699393	6.8762533	7.0298654	6.9890951	8.8255253	8.9517238	6.9408163	7.23634761	7.04254152	7.18034673	7.45112729	7.03776589	7.03786894	8.20877672	7.33136607	7.28650077	7.39065732
hsa-miR-642b-3p	4.6044161	4.4657608	5.2093456	4.665712	4.7742606	4.6668862	4.7849062	4.6241252	4.5336159	4.5835117	6.0889213	5.8932253	4.500992	4.82713267	4.77232147	5.00259385	5.40229794	4.78463412	4.79291123	5.60426844	5.18613737	5.06426053	4.99967797
hsa-miR-6507-3p	3.5306698	3.1863314	3.6979107	3.4431121	3.6299434	3.6530213	3.2469914	3.4916396	3.7055496	3.4412059	3.1458635	3.2831266	3.4748774	3.53586999	3.60795806	3.36800022	3.29429133	3.6601493	3.62591775	3.47741982	3.52253077	3.5035582	3.19935556
hsa-miR-6508-5p	4.6687932	4.3336002	4.8513642	4.3908931	4.7452087	4.4838771	4.4144011	4.5951593	4.7316125	4.8027975	4.0747532	4.4494566	4.6407567	4.45312975	4.77906052	4.24528437	4.17281893	4.66904399	4.73993459	4.41778006	4.61000588	4.63958487	4.08860592
hsa-miR-6510-5p	2.8654015	2.855277	2.7403039	2.7156392	2.8950304	2.7369521	2.8661015	2.7691555	2.6854068	2.7485816	3.4494982	3.179771	2.9655353	3.25311157	2.73094942	4.74972753	4.74585771	3.023326	2.95649897	4.01331306	2.99404578	3.17472869	4.4283786
hsa-miR-6512-5p	3.2205312	3.3304535	3.5132387	3.1594501	3.3550673	3.2996427	3.5169659	3.5427281	3.5548883	3.4226383	3.645597	3.6700424	3.1436961	4.16160051	3.74756143	3.85132375	3.92024636	3.91310931	3.66684458	3.84701498	3.63678405	3.81009081	3.81591029
hsa-miR-6515-3p	4.5717446	4.6743765	4.6325988	4.6184283	4.6315748	4.4908416	4.4532673	4.5247532	4.7283358	4.5671699	4.4192809	4.709331	4.8087396	4.41045945	4.46657875	4.21430086	4.21100539	4.45213882	4.65087877	4.34685486	4.41180183	4.56267622	4.11262821
hsa-miR-6515-5p	3.2816999	2.5906457	4.0945318	3.6414636	3.8828214	4.4421444	3.2359722	2.5427908	3.193193	2.5011787	3.4710853	3.3182886	2.5496623	3.68074487	2.71118081	3.64773403	2.92827079	2.48967748	2.47995455	3.53501437	4.028049	4.16371893	2.53395645
hsa-miR-6516-3p	2.9427909	2.8015605	2.8883199	2.6782603	2.834359	2.8301466	2.9506062	2.9697409	2.9709979	2.782944	2.903603	2.9300211	2.6841503	3.24851105	3.37731733	3.0280481	3.09953133	3.23617443	3.26386608	2.95222961	3.10297002	3.26191956	2.94189956
hsa-miR-652-3p	3.203676	3.0307058	2.9790213	3.1118434	3.0806647	3.1318184	3.1409436	3.0805215	3.1419404	3.1349631	3.1587705	2.9067292	3.3246167	3.6540683	3.65536738	3.31905237	3.18272421	3.53742231	3.38461021	3.13130945	3.20260261	3.18195332	4.37754958
hsa-miR-654-3p	3.560511	3.7372448	3.6252776	3.7178579	3.9816763	3.7282279	3.4835413	3.5221581	3.5586684	3.5203359	3.61307	3.7078067	3.911272	3.74391233	4.04939565	3.93722503	3.5279189	3.97406858	4.03867722	3.83640062	3.844135	3.99754078	4.40677811
hsa-miR-660-5p	5.4272533	5.7577648	5.5486754	5.4955573	5.4959921	5.0476382	5.3746771	5.6079342	5.7576959	5.4676763	5.0418435	4.9906449	5.6825141	6.10635819	6.19404629	5.56330972	5.53695731	5.92270308	5.76275977	5.35803821	5.78292074	5.71834473	5.86898749
hsa-miR-663a	2.9735104	2.7380432	2.9004076	2.9814612	2.8571734	2.82046	2.9050502	2.9407584	2.6522512	2.7228162	3.6022555	3.4967588	2.9768805	3.18339081	2.96058118	4.25782206	3.88873272	2.98015333	2.98298043	3.6038308	3.29764899	3.20081562	4.2841854
hsa-miR-664a-3p	5.0924808	4.9382702	4.7988178	4.6089775	4.8224487	4.7465669	4.5394335	4.9574458	5.1858895	4.9211959	4.2128988	4.5025471	4.9297735	4.87974472	4.91993941	4.43677613	4.62453247	5.14714963	5.25948501	4.53168717	4.98752967	4.89437856	4.40305615
hsa-miR-664a-5p	3.0745101	3.1199467	2.970023	2.9531387	2.8165259	2.8414646	3.016342	3.073769	2.9890449	2.8591759	2.9334257	3.0152698	2.8901782	3.047111	3.09335689	3.96419631	4.12971557	3.04196447	3.25378585	3.25381562	3.21264417	3.18372536	3.49265829
hsa-miR-664b-3p	5.0262682	4.3711793	4.5642148	4.7705408	4.7284716	4.7744217	4.4493659	4.8698256	4.7396051	4.3877318	4.3506705	4.5294185	4.5417824	5.01832339	5.22546438	4.61982439	4.72683273	5.03986693	4.90413126	4.62074121	4.6638295	4.85335791	4.12187614
hsa-miR-665	3.280999	2.9874721	3.2070119	2.7680263	3.1836634	3.0420764	3.0418881	3.1379973	3.1815008	3.0812466	3.1366071	3.1857667	3.3566889	3.25729787	3.25592766	3.20089596	3.27519735	3.11923665	3.29255962	3.14510983	3.23670123	3.24122427	3.15479499
hsa-miR-6717-5p	5.1837327	5.1040005	5.4093152	5.0630229	5.2638597	5.2023879	4.7231872	4.83468	5.5217705	4.8385323	5.0854602	5.2613668	4.6677067	5.7516798	5.53598615	4.9633368	5.0380012	5.39888795	5.32477898	5.41405519	5.33647351	5.40181645	5.04214026
hsa-miR-6723-5p	4.0820719	3.7783705	3.8120569	3.8607309	3.7857822	3.6890279	3.7072704	4.2982717	3.9435099	3.6127202	4.0453225	3.8754244	3.7568431	4.27975359	4.12375599	4.06116124	4.58007537	4.29061832	4.21627538	4.14705649	4.30015229	4.12148899	4.31451996
hsa-miR-6724-5p	4.8486103	4.1086262	4.3815586	4.0966723	4.4130939	4.3763013	4.3733368	4.4649872	4.2635079	4.3722584	5.1403181	5.0827295	4.2842917	4.74127809	4.5236129	4.97479948	4.78215324	4.53539086	4.39820067	4.9211868	4.72248024	4.58240764	5.16853805
hsa-miR-6727-5p	3.8826293	3.2208899	3.3706731	3.1967061	3.7450851	3.3617482	3.2418582	3.5113275	3.2008518	3.3269986	4.2966019	4.2401812	3.6576715	4.17749223	3.7789476	4.27045667	4.29624604	3.4917716	3.49710167	4.27254724	3.96124967	3.62222044	4.59529871
hsa-miR-6728-5p	3.4313677	3.0510315	3.4139566	3.0624006	3.3282971	3.4243153	2.9753572	3.6539211	3.1074064	3.0552126	3.5473256	3.4709515	3.0572248	3.54848317	3.17021702	3.93091013	3.78129285	3.36000192	3.14685854	3.91915925	3.81039439	3.25749083	4.14112841
hsa-miR-6731-3p	3.7274884	3.9322581	3.8254906	3.5330883	3.7487534	3.7175807	3.5983345	3.6410086	3.794325	3.8774442	3.504265	3.5846634	3.6604516	3.66767536	3.82835816	3.52507078	3.45836944	3.87879392	3.80146788	3.7815938	3.72338875	3.78470399	3.4496738
hsa-miR-6734-5p	4.5981067	4.4901641	4.7130441	4.5677775	4.7111679	4.1656189	4.4623881	4.7000502	4.5099819	4.4129196	4.7791394	4.8447373	4.1810537	5.14111762	4.95738592	4.72779734	5.03779143	4.86490089	4.76672421	5.12858173	4.86529217	4.98673606	4.73728722
hsa-miR-6737-3p	4.7342251	4.7744924	4.7846511	4.4670042	4.6962048	4.7383129	4.4175738	4.514731	4.7474838	4.7622924	4.0735239	4.4848989	4.7110302	4.35032192	4.72119434	4.13555659	4.14369209	4.64014705	4.4810972	4.55380283	4.38887829	4.45366413	4.06119395
hsa-miR-6740-5p	5.8125431	5.544637	5.9141232	5.8835899	5.8449694	5.6432187	5.6300676	5.623742	5.892051	5.5415829	6.051757	5.9610584	5.5427677	6.42098201	5.92998223	6.11114276	6.17930878	5.88012275	5.8359835	6.10805592	5.86036152	6.33730799	5.6416192
hsa-miR-6749-5p	8.6178414	8.4190316	8.1994876	8.7315039	8.7309333	8.3568158	8.6280273	8.6525675	8.457541	8.2585707	8.9272734	8.8247123	8.3853165	8.31636285	8.11285109	9.71956226	9.72763395	8.57808217	8.51253802	8.92773215	8.50927397	8.62672601	9.23966604
hsa-miR-6752-3p	4.1127614	4.2224064	3.9870873	3.7139362	4.0422218	3.8912834	3.8663283	3.78278	3.8937906	3.9663144	3.8133006	3.8324326	4.0277051	3.77055924	3.97988546	3.62246526	3.75329164	4.0349351	3.8286556	3.94624836	3.97785364	3.88202285	3.59545352
hsa-miR-6752-5p	3.7098698	3.6881487	3.8355421	3.4482971	3.6062654	3.6259492	3.4854205	3.3796519	3.6150562	3.4922703	4.2432936	4.0783408	3.7040568	3.96744576	3.49831777	4.30170795	4.29745576	3.54069216	3.66064851	4.23860056	3.93958951	3.79406404	4.28795274
hsa-miR-6756-3p	3.265278	3.2529212	3.2814369	2.9094663	3.1987103	3.2527648	3.1800609	3.1784419	3.1981764	3.1591682	3.1057278	3.1532139	3.1030556	3.20741775	3.32118466	3.12962646	3.21204869	3.34229024	3.32833597	3.2755168	3.41427533	3.2621286	3.14170553
hsa-miR-6756-5p	4.3918292	3.472285	3.5130656	3.8969733	4.0976411	3.9880875	4.1259599	3.9625123	3.7635805	3.7057019	4.6571657	4.0519526	4.3078322	3.93347472	3.71952125	4.69320861	4.97139521	4.13891624	3.87650792	4.59347291	4.27280048	3.85885588	4.1106387
hsa-miR-6757-3p	3.7693339	3.6951486	3.8323294	3.5739014	3.6557689	3.7374515	3.6165083	3.7919896	3.8061072	3.8801427	3.2082082	3.6096724	3.7518322	3.63670584	3.79268957	3.51629113	3.4072563	3.72293046	3.72559069	3.64264634	3.77803197	3.77263077	3.36242075
hsa-miR-6757-5p	4.6035931	2.8096833	3.485035	3.14835	3.3245369	2.9552842	3.0715286	2.7489556	2.885957	2.7626385	3.812723	3.7409387	2.8803105	3.56546118	2.75628373	4.37959418	3.99469718	3.27121011	2.92872073	4.09003703	3.47079058	3.68461072	3.52491679
hsa-miR-6759-3p	3.6937691	4.3105154	3.7296975	3.3818836	3.6007139	3.2683634	3.437157	3.6930869	3.5252785	3.7036238	3.6000708	3.4679526	3.5678514	3.5749852	3.7589392	3.35670523	3.45326678	3.47336912	3.64977174	3.64871942	3.61537453	3.54384127	3.34972108
hsa-miR-6760-3p	3.8991347	3.8939674	4.0536397	3.752831	3.814734	3.893402	3.6658489	3.7828687	3.8321567	3.9367226	3.6222342	3.8592849	3.930352	3.66404157	3.85176479	3.50325104	3.51658549	3.91364609	3.73162976	3.84658271	3.66952991	3.81297703	3.47896337
hsa-miR-6763-3p	4.0239755	3.6698806	4.0040102	3.69531	4.0330041	3.9469374	3.6165135	3.9896074	3.9499052	4.1517001	3.657717	3.9003535	3.8084453	3.89852718	3.96694753	3.58792161	3.75404977	3.94807037	3.90422508	3.91965773	3.94867941	3.92444945	3.52365223
hsa-miR-6763-5p	6.1247672	5.264671	4.9680406	5.3560231	6.5585908	4.7694158	5.0126906	5.6201888	5.4393043	5.1249714	5.181457	5.3593612	5.2377339	5.44296962	5.2444628	5.50086527	5.73484956	6.01247485	5.67460466	5.31405028	5.361509	5.568161	5.14324046
hsa-miR-6765-3p	3.8771218	3.4434488	3.6943406	3.5125496	4.0063821	3.5788886	3.5318959	3.5518625	3.7140496	3.7765514	3.5310016	3.859107	3.5101141	3.74349938	3.79918009	3.44349002	3.55546705	3.75314552	3.73754874	3.78790499	3.96711114	3.73733458	3.61166076
hsa-miR-6766-3p	3.754558	3.7686408	3.7464046	3.7748666	3.9154828	3.8047181	3.7535032	4.0200674	3.8069599	4.0257093	4.1463287	4.1707704	4.3027492	3.58520715	3.84362486	3.71691618	3.61842454	3.77007427	3.91786831	3.70680682	3.93771398	3.66760689	3.5201605
hsa-miR-6767-5p	3.9779001	4.0170043	4.1895928	3.6936612	4.1046412	3.8733257	3.8047903	3.9842305	4.1324181	3.9074169	4.1715849	4.191538	3.510981	4.47106993	4.3236637	3.89900913	4.27425533	4.18539427	4.32783991	4.42787354	4.270113	4.38074556	4.13073121
hsa-miR-6769b-5	4.8002761	2.78404	3.6223339	4.5991379	5.0916612	5.0911022	4.459306	3.9040481	4.1183236	4.7397249	4.9366084	4.7952367	4.4577054	4.56488111	4.48537805	5.06464292	5.31151588	4.46311427	4.52829638	5.48001827	4.59270558	5.32747792	4.2994855
hsa-miR-6775-5p	3.6560773	2.9918745	3.2190486	3.3669737	3.3248694	3.375869	3.1843985	3.3150009	3.1872931	3.2008093	4.1288796	3.6520524	3.4509881	3.66247431	3.08902028	4.68704113	4.58214661	3.37004593	3.37618776	4.12344263	3.76143242	3.63413092	4.17185627
hsa-miR-6777-3p	3.7669684	3.7293497	3.9342315	3.6305523	4.0492491	3.8876252	3.5904912	3.9442409	4.0484548	4.0807036	3.4209459	3.689235	3.8406548	3.89469368	3.96128578	3.66291715	3.72019791	3.88054268	3.85133452	3.82326638	4.02516513	3.78383302	3.51781559
hsa-miR-6780a-5	3.2066485	3.154192	3.4525566	3.1845859	3.2791213	3.0588956	3.0931274	3.217101	3.0387841	2.9464984	3.7207907	3.428307	2.8785298	3.86817759	3.52467988	3.46093484	3.73627479	3.45908569	3.38284454	3.79637457	3.42688764	3.54255823	3.97105797
hsa-miR-6780b-5	5.641336	5.4523812	5.9651785	5.4065965	5.6605014	5.5634303	5.3130906	5.5242074	5.9117473	5.4909215	6.3990983	6.393343	5.2967951	6.1845259	5.99172211	5.66621636	5.81272798	5.80929472	5.73094057	6.11379722	5.76873175	6.06035264	5.76363107
hsa-miR-6785-3p	3.786758	3.1914578	3.5954482	3.2992694	3.7720098	3.5012783	3.5610632	3.4306592	3.786273	3.5906116	3.237958	3.6866226	3.5552852	3.55293936	3.55712153	3.43346461	3.53600811	3.66245631	3.62109021	3.5749445	3.60312688	3.70087613	3.43384967
hsa-miR-6785-5p	5.9093357	5.9353694	6.6825319	5.9864994	6.0525093	6.3534196	5.8415892	5.7763333	6.1405181	6.0685665	7.1947794	7.392961	6.1148399	6.33449306	6.13124354	6.11826803	5.84395355	6.02601024	6.09764914	6.52844154	6.15594014	6.30812427	5.89819413
hsa-miR-6786-5p	3.8260197	2.9379403	3.1649358	3.4623671	3.4320546	3.4071669	3.575924	3.8716266	3.4044591	3.1600635	3.8847877	3.3258825	3.5768551	3.59484114	3.32442025	4.0322385	3.83541621	3.20876883	3.18181181	3.96609627	3.95820773	2.99088777	4.09556903
hsa-miR-6789-5p	3.308961	2.9149351	2.9306626	2.9418857	3.0084329	2.8856779	2.9304162	3.3294978	2.7203866	2.9720485	3.5359487	3.1666952	3.1134999	3.57283599	3.2266492	3.37255514	3.28874461	2.9238454	3.04639698	3.49700177	3.46692133	2.85361671	4.07472646
hsa-miR-6791-5p	4.467032	4.4188398	4.1308326	4.1881658	4.4026451	3.865189	4.3426962	4.4636231	3.6353994	4.401737	5.2372008	5.1175873	4.4044312	4.45393831	4.22155693	4.44026023	4.44003171	4.40956901	4.2784814	4.84954667	4.69980549	4.41225703	4.65970365
hsa-miR-6794-5p	3.5561335	3.0574334	3.0272213	3.5577098	3.2747323	3.1726087	3.1206022	3.3951383	3.1386764	3.00031	3.8251063	3.7730603	3.2473273	3.82830119	3.2346845	4.65716119	4.66493458	3.59515892	3.62068381	3.91091689	3.61067339	3.89134197	4.27875529
hsa-miR-6795-3p	3.8031435	3.5523525	3.9885243	3.6982529	3.9015472	3.4862177	3.5193038	3.7209646	3.7492896	3.9134762	3.2182882	3.5537489	3.7601277	3.66149221	3.9183448	3.46627467	3.54758616	3.76938904	3.70951405	3.47520806	3.8563809	3.75734913	3.37208262
hsa-miR-6797-3p	4.5586079	4.5133059	4.8680494	4.429877	4.7642882	4.6047417	4.5536162	4.5081462	4.7865754	4.8313803	4.2781564	4.5859289	4.5760384	4.4572448	4.54895587	4.13720235	4.21558499	4.70450297	4.60423848	4.62410331	4.54556288	4.63710595	4.09800173
hsa-miR-6798-3p	3.3598663	3.4632258	3.2663058	3.2187428	3.5080601	3.3322113	3.1340144	3.0894452	3.3110098	3.2421841	3.1887397	3.2737913	3.1155353	3.22853784	3.42876344	2.97915175	3.15920044	3.2442151	3.02337173	3.27962994	3.17842819	3.18127901	3.17832169
hsa-miR-6798-5p	3.1643167	3.1424166	3.1052597	3.0445644	3.2198221	2.9876865	3.0033512	3.2338598	2.9628837	3.162711	3.6119892	3.5724623	2.9276205	3.21181775	3.16990592	2.99709505	3.07851237	3.25422287	3.13764484	3.35421917	3.2024797	3.16377856	3.09498935
hsa-miR-6800-3p	4.7635751	4.4063892	4.8916508	4.4932389	4.769396	4.6256156	4.5099254	4.468138	4.7342155	4.6412797	4.0435028	4.3256966	4.7132332	4.52863419	4.72186721	4.24517027	4.22658606	4.69555153	4.71271154	4.46516769	4.56840057	4.63851082	4.02451636
hsa-miR-6800-5p	6.2280297	5.3346711	5.7708671	5.991335	6.1689942	6.0752831	5.9279943	6.1829365	6.0652323	5.6504984	6.5590304	6.4835907	6.056224	6.38959624	6.11582683	7.22276421	7.44687578	5.76448097	5.7561642	6.24790303	6.41310521	6.00120057	7.10781632
hsa-miR-6803-5p	5.9385372	6.0659447	6.481059	5.9141719	6.3586786	6.1634079	5.9856865	5.9375591	6.0963632	6.0589622	6.6046949	6.8819033	5.8617269	6.03159283	6.01752603	5.95258729	6.00989874	5.89557255	6.0265599	6.02431302	5.94951114	6.11484047	6.24508766
hsa-miR-6807-5p	3.5270029	3.2023282	3.354624	3.2229371	3.5982354	3.0682007	3.1840827	3.3786322	3.3787793	3.1313676	3.5898825	3.2527921	3.2644964	4.10226091	3.59898704	4.21924904	4.41821687	3.30366148	3.13152189	3.58266139	3.42460653	3.8111559	4.00226194
hsa-miR-6808-5p	3.6486221	3.3485562	3.5393682	3.6652459	3.3788969	3.4960045	3.6381357	3.7862685	3.564495	3.4643709	4.1156291	4.1177035	3.6997018	3.79672645	3.30404154	4.29959217	4.52526965	3.96304475	3.81145279	4.07851217	3.93805769	3.93002581	4.09928707
hsa-miR-6812-3p	3.8975571	3.8832914	3.9420217	3.6918154	4.1215354	3.7316004	3.7375513	3.5596234	3.6773964	3.9079644	3.4959114	3.6897221	3.6786641	3.72397128	3.93986118	3.59719831	3.58355745	3.94338861	3.65919944	3.73564109	3.86952707	3.69361592	3.46622076
hsa-miR-6812-5p	5.1129228	4.17884	4.5468643	4.4273666	4.5415201	4.2026045	4.2368641	4.2891425	3.7988154	4.1859139	5.3607631	5.1865829	4.4635412	5.04142229	4.44336774	5.34750925	5.45179604	5.25355678	4.32455707	5.50131197	4.71235449	4.72266693	5.23488261
hsa-miR-6813-3p	4.3922274	4.0980273	4.3568289	4.171651	4.2075884	4.097107	3.823971	4.2176562	4.2527339	4.1915606	3.7169889	3.9969391	4.1540698	4.02140245	4.24237169	3.89567822	3.9178154	4.1125097	4.16823173	4.05448173	4.17777261	4.08124144	3.75084447
hsa-miR-6819-3p	4.5465718	4.3498709	4.8601316	4.5904929	4.5917177	4.7196715	4.3210623	4.5911606	4.6925455	4.6047818	4.0948897	4.4121563	4.5202554	4.40045213	4.65024493	4.15865346	4.24755503	4.56065776	4.49600354	4.38388413	4.43932755	4.58394503	4.08764328
hsa-miR-6820-5p	4.6022133	4.628468	4.282602	4.6007499	4.6086674	3.9844185	4.3335805	4.8976541	4.186033	4.3548325	4.3344736	4.4660835	4.4785279	4.61009753	4.34119643	4.35163947	4.87595992	4.55059588	4.59902197	4.24158474	4.66261948	4.91676012	4.31189942
hsa-miR-6821-5p	7.1313895	6.2256388	6.9025925	6.7767143	6.7894436	7.0198472	6.7826262	7.1231094	6.8218812	6.5423076	7.1509506	6.7872462	6.8300429	7.01901717	6.94796688	7.03452335	6.92606214	6.48573611	6.6065405	6.82134043	6.99269257	6.37519606	7.17945967

hsa-miR-6824-3p	4.0173603	3.6198775	3.8998675	3.6666158	3.8210249	3.9589245	3.7167374	3.801607	3.840292	3.9668437	3.3846186	3.7143052	3.5700756	3.81180228	3.87414717	3.52911371	3.63103303	3.84199201	3.90379397	3.7747487	3.81986621	3.7950031	3.40528811
hsa-miR-6826-5p	8.0931697	7.6715054	7.7360316	8.3168268	8.1270508	7.8706424	7.6247369	8.3958523	8.062822	7.8215289	7.5828457	8.6366814	7.6958541	7.61201784	7.33308243	7.85587557	8.56721255	8.26158669	8.20593109	7.5421965	7.72751109	8.05576317	7.64191197
hsa-miR-6829-5p	3.9349934	3.3871607	3.4923254	4.0880019	4.1357769	3.6958152	3.502533	4.3635057	3.8032997	3.520952	3.8200786	4.2854349	3.5736601	4.1690212	3.67287781	4.25214169	4.53810097	4.40801433	4.32074028	4.0653445	4.10499112	4.32195689	4.00574133
hsa-miR-6840-3p	3.4260887	3.1196117	3.0032151	3.4125171	3.7021334	2.927524	3.2456086	3.5097891	3.279453	3.4197963	3.4057171	3.477386	3.292672	3.61774664	3.54309421	3.67438834	3.8319625	3.76944919	3.56952177	3.70830386	3.21982747	3.87473729	3.90768782
hsa-miR-6845-5p	2.8822056	2.8090459	3.0429286	2.9219384	2.9911051	2.7883937	2.8607546	3.3111413	2.8388004	2.7623192	3.4917763	3.3053284	2.8184649	3.32517289	3.28928122	3.91174808	4.15401142	3.11200712	3.13702191	4.66557295	3.20766596	3.13019904	3.9559589
hsa-miR-6848-3p	3.8908721	3.7930846	4.1538389	3.7810539	3.808351	3.9089247	3.8037974	3.7246418	3.950259	3.9644595	3.7245737	3.6744071	3.7349693	3.89211807	3.80954766	3.62437584	3.54313705	3.93910326	3.923571	3.81951378	3.87176564	4.02768807	3.50026044
hsa-miR-6850-5p	3.95581	3.0008739	3.4298677	3.2253347	3.8890003	3.6176519	3.2792865	3.6830652	3.3673008	3.4052013	4.0396025	3.9107945	3.3270402	3.98446555	3.97522181	3.88607762	4.11959916	3.46361263	3.50011128	4.16724121	4.16051326	3.20169544	4.16887096
hsa-miR-6851-3p	3.9193149	4.0068371	4.0783011	3.9320864	4.0886495	4.2876404	3.7171171	3.7229978	4.1424408	4.0760386	3.6440041	3.9052551	3.9316407	3.85275658	4.18955162	3.66370372	3.66591732	4.25692292	3.98028014	3.92636932	4.01636222	3.90263851	3.38735379
hsa-miR-6855-3p	3.5534702	3.5419257	3.5828135	3.3034943	3.5748604	3.2940859	3.392792	3.3266799	3.3873002	3.6966832	3.2746287	3.3608381	3.5706953	3.50305135	3.52099531	3.23221679	3.39595421	3.52411496	3.58911235	3.36018302	3.57315739	3.56482945	3.31187026
hsa-miR-6858-3p	4.0491672	3.9774943	4.1301397	3.7698701	4.0586832	3.9428611	3.6839392	3.9501288	4.0915594	4.0385822	3.5284924	3.7356778	3.9822707	3.84105831	3.97083254	3.65170821	3.6597539	3.95965525	3.97289951	3.84447408	3.86105193	4.02038539	3.49360889
hsa-miR-6858-5p	3.491313	3.4087	3.7476481	3.4229329	3.3197999	3.4977665	3.3267362	3.3314856	3.0074063	3.228322	4.0132137	4.0722459	3.4509808	3.80985217	3.25688692	4.77431512	5.79199353	3.91710208	3.49870657	4.57276068	3.70075997	4.06028243	4.15275318
hsa-miR-6861-3p	3.6604116	3.5838244	3.8638035	3.3653943	3.7988753	3.6176098	3.3239198	3.4868334	3.6879591	3.7040026	3.1996988	3.327742	3.517703	3.6935767	3.61091098	3.40620367	3.46490505	3.63990521	3.64917987	3.58977016	3.58952878	3.69060643	3.31548521
hsa-miR-6865-3p	4.4244111	4.1172125	4.7079048	4.166431	4.5270893	4.3577363	4.2739585	4.0004588	4.473381	4.4204804	3.6081955	4.2007809	4.2848527	4.33191907	4.53521267	3.8972058	3.92323886	4.49003022	4.34938716	4.1099855	4.28914096	4.35521005	3.79960109
hsa-miR-6867-5p	3.2292998	3.2209316	3.6184425	2.9409393	3.0580504	3.0841448	3.0182465	3.0101485	2.7795829	3.0634218	3.4324382	3.3414049	3.0773922	3.31388619	2.87743248	4.75215336	4.74218362	3.4331288	3.60413826	3.83700856	3.6903696	3.9165331	3.96690821
hsa-miR-6869-5p	7.4575182	7.1360436	6.8388993	7.4287867	7.3059484	7.1594726	7.6216338	7.321217	7.0429076	8.031104	8.5595609	7.8833229	7.647092	7.07000758	7.13760994	9.27336955	9.34259021	7.09323064	6.88433837	8.08343904	7.69658561	7.56535669	9.21312208
hsa-miR-6870-3p	3.6484066	3.5330056	3.6721437	3.4349384	3.5034269	3.5333484	3.2945554	3.3640974	3.5806596	3.5750948	3.2081423	3.4551183	3.4279633	3.4300268	3.48298205	3.21011145	3.21487959	3.4137158	3.34228337	3.25469548	3.48215216	3.38279185	3.1016772
hsa-miR-6872-3p	3.1250036	2.9000549	2.8046314	3.1553415	3.1371542	2.9542751	2.9045547	3.2621968	3.0615592	3.0395831	3.1477453	3.2456962	3.1087384	3.19084134	3.04917433	3.2495548	3.59239286	3.25954887	3.39559005	3.25790315	3.15002576	3.23893448	3.53712026
hsa-miR-6875-5p	7.9361063	7.9463875	8.2644409	7.8556963	7.9697547	8.0259492	7.9812621	7.7134345	8.1026495	7.8626917	8.3727492	8.6131263	7.4958135	8.14177667	7.99138814	7.58113618	8.74632821	8.05746973	7.87200999	8.37954315	7.8202236	7.94239728	7.74149725
hsa-miR-6879-5p	6.6662705	6.2246344	6.5655349	6.3959442	6.5628309	6.2765409	6.1834261	6.3325813	6.5340455	6.2321337	6.5528241	6.4059018	6.1719768	6.74181221	6.63063765	6.47696853	6.41420073	6.78683996	6.45236304	6.9723958	6.45110196	6.69925732	6.00709618
hsa-miR-6880-3p	4.0421963	3.6837987	3.984551	3.6328082	3.9196662	3.7566072	3.6388087	3.7865461	4.0743296	3.9949101	3.521363	3.699866	3.9340632	3.87016568	3.95083058	3.51488521	3.55472636	3.81660613	3.80260669	3.76007933	3.81513056	3.84986561	3.42795682
hsa-miR-6889-3p	4.5482115	4.519236	4.8023312	4.4095708	4.6074337	4.5283214	4.4333181	4.4577198	4.8654983	4.6966181	4.1319233	4.5133492	4.443547	4.43582997	4.64472273	4.0589791	4.22774521	4.63789453	4.54937667	4.47036083	4.55973394	4.64144891	3.99907079
hsa-miR-6891-5p	3.8358538	3.4854346	3.7093824	3.7105371	3.8385939	3.9227316	3.8745052	4.1404068	3.9189266	3.6043051	4.2014514	3.8981405	3.728088	4.19592961	3.72629006	4.42478675	4.65694855	3.79644375	3.76958411	4.3654743	4.35531861	3.84722139	4.34499875
hsa-miR-6893-5p	5.9769506	5.9254796	6.6264162	6.190009	6.1592996	6.0820741	6.0492156	6.1222469	5.9155365	5.974968	7.2259045	7.416771	6.0074155	6.19951358	5.7581011	6.25154739	6.0879962	5.95779696	6.0336308	6.33591579	6.25746653	6.50782902	5.96296407
hsa-miR-7107-5p	5.3846682	3.9551825	4.1389097	4.179513	4.3042425	4.6253749	4.2355187	4.2375051	3.5688598	3.814618	4.9013467	4.4539928	4.3592478	4.59326469	4.03774359	5.75538068	5.61217902	5.00574854	4.2607372	5.23611716	4.62867532	4.58216966	5.24693299
hsa-miR-7108-5p	4.1653496	3.6386649	3.6021898	3.8139652	3.9310187	3.8641147	4.2082949	3.8657311	3.6082274	3.9223882	4.7975508	4.2718277	4.1651244	4.12085916	3.69876181	4.6407452	4.42209639	3.63254255	3.97527651	4.55262201	4.24616812	3.76533631	4.54088583
hsa-miR-7110-5p	4.0470467	4.1671245	4.1766945	3.8650177	4.2538065	4.1555046	4.0923276	4.2656877	3.7215506	4.1725344	4.8358058	4.8001395	4.1291277	4.40017708	4.22262023	4.85098	4.82125541	4.28202644	4.34222552	5.05304313	4.49297421	4.39436743	4.71189471
hsa-miR-7111-3p	3.595901	3.4064447	3.4943725	3.2407561	3.6045427	3.4406277	3.2356536	3.3271414	3.363711	3.664207	3.4948207	3.6112368	3.3136446	3.48410419	3.60747681	3.33243238	3.4188652	3.60372401	3.34223296	3.60974758	3.33366639	3.65735365	3.53489593
hsa-miR-7114-5p	7.0774999	7.0705501	7.1048275	7.4078947	7.2981471	6.7530778	7.0299108	7.2793722	7.2262604	6.9531923	6.7737958	7.2529416	6.8742061	6.97592524	7.04613177	6.64021629	7.47157036	7.17587666	7.20313198	6.41776775	7.04258943	7.41443479	6.7768421
hsa-miR-7-1-3p	3.6707637	3.3295569	3.3983119	3.113127	3.4053986	3.3659675	3.1622955	3.3402115	3.1875256	3.1989262	3.1207181	3.1560661	3.3676694	3.75031189	3.66129956	3.3052207	3.46658758	3.46643704	3.5958319	3.35760145	3.40556809	3.74772945	3.60973158
hsa-miR-7150	4.571242	4.2198128	4.5058856	3.9428975	4.5686718	4.3705277	3.9787931	4.0914136	3.9623236	4.3220702	4.8578223	4.860332	4.2114983	4.60475832	4.31305874	4.57992037	4.65048922	4.59268963	4.45324704	4.96080253	4.62469895	4.50000517	4.82841811
hsa-miR-7152-3p	3.5025948	3.137967	3.1629631	3.1896563	3.3525753	2.8283515	3.1434358	3.3400337	3.0829072	3.0205594	3.6842126	3.4470211	2.8979217	3.79195961	3.48050414	3.76209044	4.65130055	3.52360424	3.29435848	3.86557643	3.44389638	3.38402853	3.6759826
hsa-miR-7152-5p	4.5396415	2.6516362	3.976379	3.5582758	4.1233788	4.113539	3.3796394	2.6769867	3.3236448	2.5456809	4.046859	3.5632415	2.6110592	4.00496094	2.67865739	4.65392448	2.99279973	4.98678272	2.68105131	3.34974561	3.96961999	4.28179836	2.83624816
hsa-miR-7-5p	2.9340171	2.9667593	3.0949907	3.1327147	2.9363097	2.9197277	3.1656835	3.1260563	3.1829022	3.1439798	2.9513788	2.7197184	3.2647659	3.54090671	3.641464	3.50160607	3.28052528	3.49400745	3.54542813	3.18382019	3.36064948	3.28092271	3.25108083
hsa-miR-762	5.3035042	4.8821154	5.3372067	5.1139075	5.4038002	5.1598713	4.7704522	5.2087446	5.039927	4.9132137	6.2344802	6.20043	5.0095986	5.33850975	5.37821882	5.42008198	5.67100463	5.04173488	5.15240565	5.87855774	5.44843538	5.37762117	5.98168575
hsa-miR-7641	10.959331	10.87339	11.808428	11.045083	11.098825	11.440508	11.159544	11.055637	10.783989	10.919131	13.187335	12.997953	10.75462	11.422805	11.0349441	11.07468	11.2247652	11.2854074	11.0988253	12.2097245	11.4027585	11.6404818	11.9552273
hsa-miR-766-3p	4.3043269	4.3104781	4.47034	4.2329362	4.2013899	4.2346186	4.18959	4.2501205	4.3388606	4.5130094	3.8164771	4.2634213	4.0383348	4.34786688	4.34787982	4.10820735	4.19675564	4.34671075	4.39023866	4.26870625	4.37676351	4.40789197	3.86281381
hsa-miR-7704	5.2213869	5.0760191	5.0196494	5.151511	5.4212269	4.98819	5.4391698	5.3550142	4.9058719	6.1511593	6.1252709	5.7605037	5.3742938	5.28166785	5.11443104	6.01986727	6.41823482	5.48496792	5.09405093	6.00523463	5.47942383	5.77549167	6.50574741
hsa-miR-7847-3p	4.5039265	2.9711461	3.1777095	3.3265366	3.4863266	3.5802613	3.5150762	3.4035207	3.0846149	2.948765	4.1903185	4.1329785	3.4915292	3.75765351	3.31137589	4.97637974	4.85742079	3.58160282	3.37041322	4.98159585	3.8437603	3.91308345	4.49113788
hsa-miR-7974	3.8121942	3.8477337	4.1237382	3.7459529	4.1694406	3.9452449	3.8312741	3.7391342	4.0884172	4.0084	3.3441176	3.6886419	3.7476451	3.92146364	3.97708825	3.77073215	3.71555486	4.00261564	4.03331346	3.64995677	4.0317428	3.96467585	3.47287486
hsa-miR-7975	17.036932	16.950602	16.942963	17.036932	17.036932	16.916959	17.028993	17.051696	16.983962	16.9184	17.036932	17.191915	17.013949	16.9451006	16.8035353	16.9786765	17.0369318	17.0369318	17.0446993	16.7713327	16.9866826	17.0369318	17.2837282
hsa-miR-7977	15.554358	15.191082	15.342437	15.613292	15.610919	15.342437	15.342437	15.756544	15.342437	15.336561	15.468939	15.613292	15.337828	15.6132918	15.5975689	15.348888	15.6132918	15.7611907	15.6304867	15.1886624	15.3015406	15.6132918	15.707292
hsa-miR-8060	3.2390273	3.4218253	3.3949771	3.2530318	3.5052158	3.2003664	3.1435021	3.1620599	3.1329867	3.4533423	4.1351499	3.9218477	3.256499	3.58609792	3.30894228	3.67427289	3.68902661	3.34791042	3.33435222	3.89953405	3.57985378	3.59975257	4.01417038
hsa-miR-8063	4.471862	4.1659758	4.050763	3.7421387	4.2412129	4.0780495	4.0594417	4.123822	3.6422736	3.6527657	4.9519912	4.6988208	3.9882018	4.71874839	4.34802004	4.66072192	4.61639016	4.45714616	4.32917808	4.84912309	4.54644179	4.2495422	5.1327873
hsa-miR-8069	14.222005	14.115367	14.069116	14.059956	14.198152	14.056333	14.188012	14.003714	13.893805	14.098939	14.199252	14.166279	14.208626	13.6633119	13.6192987	14.2418014	14.2468988	13.8489728	13.5786249	14.0430927	13.759086	13.9210445	13.645632
hsa-miR-8072	4.0107936	3.885375	4.1421439	3.9927552	3.9670443	3.9779918	4.1238666	4.016579	3.9124346	4.038077	4.4201644	4.1534827	3.9913043	3.98099774	3.88673487	3.8718121	4.16346411	3.97717365	4.18096436	4.37249512	4.17572301	4.06490949	3.76812738
hsa-miR-8485	5.3259015	4.4833513	4.834122	4.3471287	4.4388918	4.4036384	4.1754304	4.5856104	4.356231	4.219046	5.2885241	5.527964	4.1870316	4.69200872	4.9262766	5.1325131	5.182286	5.02789669	4.49648155	5.57945502	4.80363025	4.91707182	5.42633737
hsa-miR-874-3p	5.4047777	5.1718433	5.0641354	4.9447735	5.5947902	5.0307294	4.863423	5.1898598	5.1558782	5.1782738	5.0194443	5.2374953	5.5533374	5.2411153	5.27177776	4.91283928	5.25995725	5.45042678	5.37492759	5.09402176	5.33387068	5.56569628	4.90985282
hsa-miR-885-5p	9.2461494	9.2218229	9.051471	8.9310131	9.229087	9.2332494	9.0500918	8.6982408	9.2585888	9.1835095	8.1227144	9.023028	8.8705513	8.59376645	8.69955307	8.31920208	8.18599013	8.92790919	8.91695803	8.79692069	8.89943322	8.88492544	6.67800167
hsa-miR-92a-3p	9.2771784	9.2411007	9.1096608	8.8597352	9.1407013	9.0877417	9.1076331	9.1215792	9.4487299	9.1051729	8.3627162	8.8881347	9.0409472	8.74669139	8.90056612	8.55036662	8.73655392	8.55203222	8.94604078	9.19533979	8.69364138	8.95665551	7.59253396
hsa-miR-92b-3p	3.6445559	3.3060929	3.7178895	3.3009378	3.5763799	3.417965	3.514069	3.4895413	3.6571363	3.7043822	3.1134763	3.4648463	3.4298752	3.54417843	3.90839192	3.26559767	3.33865828	3.63987913	3.78573468	3.51278036	3.4577779	3.70734727	3.32715926
hsa-miR-933	3.4024601	3.5303826	3.6750477	3.1417184	3.2280681	3.3198136	3.284893	3.4101577	3.3755781	3.3896986	3.0265842	3.2733168	3.2300729	3.29558028	3.3376921	3.11205286	3.26443925	3.42134399	3.49988132	3.37089987	3.2491618	3.36919928	3.14982258
hsa-miR-93-5p	6.9297473	7.3128993	7.1188407	7.0348057	7.3286296	6.926158	7.1329169	7.0713178	7.3804751	7.2067962	6.0424116	6.4931764	7.1057128	7.39986597	7.488986	6.93164493	6.93920015	7.04334891	7.27937896	6.8558312	7.17718865	7.24413655	5.65186734
hsa-miR-937-5p	4.827174	4.1330275	4.2182538	4.9463519	4.8493768	4.8190167	4.9418805	4.8546909	4.605655	4.4344848	5.7694404	4.9275681	5.1507329	4.46542011	4.31962525	4.96362645	5.00371065	4.56689558	4.61565676	5.09681286	5.00809433	4.31252481	4.2567508
hsa-miR-939-5p	5.4834115	5.3086438	5.165061	5.8450189	5.5919658	5.1170787	5.146651	5.6760546	5.5018021	5.1180905	5.0412591	5.3932855	5.3306094	5.33995804	5.41926692	5.80277217	5.99037116	5.56978064	5.61825983	5.30451188	5.40694411	5.89702021	5.55584687
hsa-miR-940	6.0703776	5.4659732	5.9364404	5.8584223	5.8863238	5.7656334	5.5817577	5.9431602	6.017585	5.6601451	6.46782	6.3736405	5.7737023	5.9265689	5.91320591	5.89073726	5.95541529	5.7478235	5.72102264	6.10230549	5.84341081	5.87983035	6.20780775
hsa-miR-95-3p	4.1189539	4.1839883	4.0761081	4.4812324	4.217823	4.0235438	3.841535	4.4278735	4.3804269	4.5516183	5.727113	3.9119139	4.2675241	4.3545717	4.78962739	4.82700558	4.41323364	4.47139159	4.70081786	4.20243656	4.53439973	4.64500925	8.42956554
hsa-miR-96-5p	2.9513204	2.7772887	2.9019601	2.7046341	2.8953327	2.7078133	2.7813816	3.0821215	3.3847225	2.8711318	2.8266397	2.6189893	2.8007731	3.49084823	3.89809027	3.38736867	3.04681774	4.45668939	3.5286109	3.11358704	3.16535805	3.11344267	4.95380799
hsa-miR-98-3p	3.7784167	3.4666056	3.9015259	3.444535	3.7397894	3.6731852	3.5315099	3.4798899	3.7828511	3.7524265	3.3233423	3.4820727	3.4819233	3.56442068	3.75248292	3.4638983	3.49337558	3.77820901	3.86209137	3.42977248	3.74866759	3.81356291	3.37170609
hsa-miR-98-5p	5.3660527	5.4645673	5.3122411	5.2458136	5.6547886	4.8556159	5.2140664	5.5120743	5.3660384	5.397451	5.0672427	4.9418391	5.6603599	5.75689587	5.88681606	5.6281009	5.35005835	5.93777134	5.77853467	5.46625702	5.97754856	5.79520959	5.93413541
hsa-miR-99a-5p	9.9556056	10.023865	9.6604692	9.9269367	9.9331025	9.7192821	9.9459431	9.9664561	9.8484088	10.046809	9.5631252	9.7331197	9.9528226	9.6756547	9.67602183	9.75129396	9.7657294	9.74174242	9.99822987	9.87834224	9.93546318	9.98611984	9.74120121
hsa-miR-99b-5p	4.7447426	5.049309	5.156249	4.949322	5.1176144	5.017294	4.5570861	5.089924	5.0684223	5.021524	4.3235294	4.7483755	5.2266023	5.70975557	5.80918429	5.50725519	5.0778175	5.6588568	5.22171879	4.61141635	5.0929513	5.25542189	5.31297519

Abbreviations: IC, inactive carrier state; ENH, HBeAg-negative hepatitis. Values represent normalized signal values for individual samples.
