# Supplementary material for: The microRNA (miRNA) signatures in different phases of the natural history of chronic hepatitis B virus infection
Source: Sci Rep. 2026 May 29;16:24608. doi: 10.1038/s41598-026-53116-z (PMC13454168; doi:10.1038/s41598-026-53116-z)
Supplement: Supplementary file 2 [file 41598_2026_53116_MOESM2_ESM.docx]

**The microRNA (miRNA) signatures in different phases of the natural history of chronic hepatitis B virus infection**

**Qiong Wang,** ^†,1^ **Fahong Li,** ^†,2^ **Yurong Yao,** ^†,3^ **Jinyu Wang,** ^2^ **Jun Wang,** ^4^ **Yong Lin,** ^5^ **Ting Wang,** ^3^ **Jiming Zhang,** *^,2^ **Mengji Lu***^,1^

**Supplementary Materials and Methods**

**Sample** **exclusion.** We performed clustering dendrogram and principal component analysis (PCA) on all 43 samples (Fig. S1). Two samples (IA_10 and ENH_10) showed divergent patterns and were excluded, resulting in 41 samples: IT (n = 10), IA (n = 9), IC (n = 13), and ENH (n = 9) (Fig. S1a–c). Further analysis revealed that IA_10 and ENH_10 exhibited abnormal cell compositions with elevated expression of endothelial gene markers (Fig. S1d).

**Supplementary Results**

**Analysis of immune cell infiltration and the expression of 92 liver cell-type marker genes in the liver samples from CHB patients**.

Previous analyses by our group and others have shown that immune cell infiltration is one of the most critical events occurring in the liver during chronic HBV infection^1-3^. Immune cell infiltration, the expression of immune activation related markers, and induction of genes like interferon-stimulated genes (ISGs) resulted in significantly differential expression of thousands of host genes^1^. In this study, immune cell infiltration in liver samples from CHB patients was assessed using CIBERSORT (Fig. S2a). Consistent with prior reports, the proportions of CD8⁺ T cells and follicular helper T cells were elevated in liver samples from the IA and ENH groups, while resting mast cells were reduced in the IA group. In the IC group, M0 macrophage levels decreased, whereas resting CD4⁺ memory T cells and resting natural killer (NK) cells were increased. Overall, these findings align with previously published data^1,3,4^.

To further characterize hepatic cell-type composition, we analyzed the expression of a curated panel of 92 liver cell-type marker genes (Table S2). These selected markers were described in our previous publication^1^ and were derived from single-cell RNA sequencing analyses of hepatic cells^5,6^. A heatmap of marker gene expression sorted by cell type is shown in Fig. S2b. Using the 92-marker panel with a brief significance screen (BH FDR < 0.05; FC ≥ 1.5 or ≤ 0.67; lineage called increased/decreased if ≥ 50% markers passed; data not shown), significant differences were confined to IA vs IT/IC phase, whereas ENH phase showed no significant changes versus either group. In IA phase, the markers of B cells, plasma cells, αβ T cells, γδ T cells, and macrophages (iMac and nMac) were significantly increased; only a subset of the endothelial cell, cholangiocyte, and HSC markers showed limited increases. The hepatocyte markers were largely unchanged across phases, except for BCHE and CYP2A7, which were decreased in IA vs IC phase.

We also examined the expression of 110 immune-related genes, including immune regulatory molecules, cytokines, and chemokines, and compared them with previously published data^1^ (Table S3). Consistent with earlier findings, the expression of most negative immune regulators, pro-inflammatory cytokines and chemokines, and select chemokine receptors was positively correlated with serum ALT levels, but not with HBV DNA levels (Fig. S2c).

These findings suggest that hepatic gene expression profiles in CHB are shaped by two primary factors: immune cell infiltration and immune-mediated gene regulation.

**Phase-stratified analysis of correlations between hepatic cell type–enriched miRNAs and marker genes.**

Phase-stratified validation shows that the PHH-enriched hsa-miR-122-3p is significantly correlated with APOB (overall Spearman R = 0.425, P = 0.006; IA phase Spearman R = 0.700, P = 0.036), HSD11B1 (overall Spearman R = 0.642, P < 0.001; IA phase Spearman R = 0.783, P = 0.013) and HP (overall Spearman R = 0.389, P = 0.012; IA phase Pearson R = 0.718, P = 0.029) but not in IC or ENH phases (P > 0.05) (Fig. 5e). The KC-enriched hsa-miR-155-5p correlates positively with CD163 (overall Spearman R = 0.717, P < 0.001; ENH phase Pearson R = 0.878, P = 0.002), with no significant associations in other phases; the HSC-enriched hsa-miR-199a-3p correlates positively with ACTA2 (overall Pearson R = 0.627, P < 0.001; IT phase Pearson R = 0.663, P = 0.037, IC phase Pearson R = 0.676, P = 0.011); and the LSEC-enriched hsa-miR-126-3p fails to correlate significantly with CLEC4M in any phase (P > 0.05) (Fig. S3).

**Cell type–enriched patterns of top immune miRNAs.**

For the seven miRNAs most strongly correlated with αβ T-cell markers (Fig. 4a, right panel), we examined their expression across 196 sorted primary human cell types using the UCSC human cellular microRNAome [(UCSC Genome Browser](https://genome.ucsc.edu/)). Box‐and‐whisker plots show reads per million (RPM) of seven miRNAs—hsa-miR-155-5p, hsa-miR-142-3p, hsa-miR-146a-5p, hsa-miR-142-5p, hsa-miR-222-3p, hsa-miR-342-5p and hsa-miR-150-5p (Fig. S4). All except hsa-miR-222-3p are markedly enriched in immune lineages compared with epithelial, endothelial and other lineages. These plots were obtained from the UCSC Genome Browser “cellular microRNAome” tracks^7-12^.

**Supplementary Figures**

**Fig. S1. Sample exclusion and clustering analyses based on mRNA expression of 92** **liver cell-type marker genes.** (**a**) Hierarchical clustering dendrogram of all 43 CHB liver biopsy samples using mRNA levels of 92 intrahepatic cell-type marker genes. (**b**) Principal component analysis (PCA) of the 43 samples based on mRNA expression of these markers. (**c**) PCA of the remaining 41 samples after excluding outliers IA_10 and ENH_10. (**d**) Heatmap of Z-score-normalized mRNA expression of the 92 marker genes across all 43 samples; rows are grouped by cell type (left color bar) and columns by clinical phase (top color bar), with hierarchical clustering performed within each group (red = high expression; blue = low expression). Abbreviations: IT, immune tolerance; IA, immune active; IC, inactive carrier; ENH, HBeAg-negative hepatitis.

**Fig. S2. Immune cell infiltration and intrahepatic gene expression across CHB phases.** (**a**) Immune cell composition in liver biopsy samples was estimated using CIBERSORT based on bulk RNA-seq data. Relative proportions of 22 immune cell subsets across the four CHB phases (IT, IA, IC, and ENH) are shown. (**b**) Heatmap displaying the expression of 92 liver cell-type marker genes, grouped by lineage (e.g., hepatocytes, immune cells, stellate cells, endothelial cells), across all samples; rows were hierarchically clustered within each cell-type group, and columns were hierarchically clustered within each clinical phase. (**c**) Heatmap of 110 selected immune-related genes, including immune regulators, cytokines, and chemokines, showing expression levels across phases and their associations with ALT and HBV DNA levels, with clustering as in (**b**). Values are z-score-normalized (red = high; blue = low). Abbreviations: IT, immune tolerance; IA, immune active; IC, inactive carrier; ENH, HBeAg-negative hepatitis; ALT, alanine aminotransferase; HBV, hepatitis B virus.

**Fig. S3. Correlation of hepatic cell type–enriched miRNAs with their corresponding marker genes.** Scatter plots show expression of KC-enriched hsa-miR-155-5p versus CD163, HSC-enriched hsa-miR-199a-3p versus ACTA2, and LSEC-enriched hsa-miR-126-3p versus CLEC4M across all 41 liver biopsy samples (left panels), within each CHB phase (IT, IA, IC, ENH; middle panels with 95% CI shading), and phase-specific correlation coefficients with 95% CIs (right panels). Correlation coefficients were calculated using Pearson correlation when both variables passed the Shapiro–Wilk normality test and Spearman rank correlation otherwise. Raw p-values are shown in the scatter plots, and p < 0.05 is indicated in red. For phase-specific comparisons, 95% confidence intervals were estimated using Fisher’s Z-transformation. Abbreviations: IT, immune tolerance; IA, immune active; IC, inactive carrier; ENH, HBeAg-negative hepatitis; CI, confidence interval.

**Fig. S4. Expression of selected miRNAs across primary human cell types.** Box-and-whisker plots show reads per million (RPM) for hsa-miR-155-5p (a), hsa-miR-142-3p (b), hsa-miR-146a-5p (c), hsa-miR-142-5p (d), hsa-miR-222-3p (e), hsa-miR-342-5p (f), and hsa-miR-150-5p (g) across 196 sorted primary human cell types. The plots were generated from the UCSC Genome Browser “cellular microRNAome” tracks (hg38). To locate these plots in the [UCSC Genome Browser](https://genome.ucsc.edu/), open “My Data”, select “Track Hubs”, choose the “hg38” link associated with the “ABC of cellular microRNAome” hub, and query the respective miRNA names.

**Supplementary References**

1	Wang, J. *et al.* Cell-type-specific expression analysis of liver transcriptomics with clinical parameters to decipher the cause of intrahepatic inflammation in chronic hepatitis B. *Imeta* **3**, e221 (2024). https://doi.org:10.1002/imt2.221

2	Vanwolleghem, T. *et al.* Re-evaluation of hepatitis B virus clinical phases by systems biology identifies unappreciated roles for the innate immune response and B cells. *Hepatology* **62**, 87-100 (2015). https://doi.org:10.1002/hep.27805

3	Montanari, N. R. *et al.* Multi-parametric analysis of human livers reveals variation in intrahepatic inflammation across phases of chronic hepatitis B infection. *J Hepatol* **77**, 332-343 (2022). https://doi.org:10.1016/j.jhep.2022.02.016

4	Li, N. *et al.* Intrahepatic transcriptomics reveals gene signatures in chronic hepatitis B patients responded to interferon therapy. *Emerg Microbes Infect* **11**, 1876-1889 (2022). https://doi.org:10.1080/22221751.2022.2100831

5	MacParland, S. A. *et al.* Single cell RNA sequencing of human liver reveals distinct intrahepatic macrophage populations. *Nat Commun* **9**, 4383 (2018). https://doi.org:10.1038/s41467-018-06318-7

6	Aizarani, N. *et al.* A human liver cell atlas reveals heterogeneity and epithelial progenitors. *Nature* **572**, 199-204 (2019). https://doi.org:10.1038/s41586-019-1373-2

7	Patil, A. H., Baran, A., Brehm, Z. P., McCall, M. N. & Halushka, M. K. A curated human cellular microRNAome based on 196 primary cell types. *Gigascience* **11** (2022). https://doi.org:10.1093/gigascience/giac083

8	McCall, M. N. *et al.* Toward the human cellular microRNAome. *Genome Res* **27**, 1769-1781 (2017). https://doi.org:10.1101/gr.222067.117

9	de Rie, D. *et al.* An integrated expression atlas of miRNAs and their promoters in human and mouse. *Nature Biotechnology* **35**, 872-878 (2017). https://doi.org:10.1038/nbt.3947

10	Juzenas, S. *et al.* A comprehensive, cell specific microRNA catalogue of human peripheral blood. *Nucleic Acids Research* **45**, 9290-9301 (2017). https://doi.org:10.1093/nar/gkx706

11	Lorenzi, L. *et al.* The RNA Atlas expands the catalog of human non-coding RNAs. *Nature Biotechnology* **39**, 1453-1465 (2021). https://doi.org:10.1038/s41587-021-00936-1

12	Patil, A. H. & Halushka, M. K. miRge3.0: a comprehensive microRNA and tRF sequencing analysis pipeline. *Nar Genom Bioinform* **3** (2021). https://doi.org:10.1093/nargab/lqab068
